# Structure Elucidation of Prenyl- and Geranyl-Substituted Coumarins in *Gerbera piloselloides* by NMR Spectroscopy, Electronic Circular Dichroism Calculations, and Single Crystal X-ray Crystallography

**DOI:** 10.3390/molecules25071706

**Published:** 2020-04-08

**Authors:** Tuo Li, Xue Ma, Daniil Fedotov, Louise Kjaerulff, Karla Frydenvang, Sonia Coriani, Paul Robert Hansen, Kenneth T. Kongstad, Dan Staerk

**Affiliations:** 1 Department of Drug Design and Pharmacology, Faculty of Health and Medical Sciences, University of Copenhagen, Universitetsparken 2, DK-2100 Copenhagen, Denmark; tuo.li@siat.ac.cn (T.L.); louisek@sund.ku.dk (L.K.); karla.frydenvang@sund.ku.dk (K.F.); prh@sund.ku.dk (P.R.H.); kenneth.kongstad@sund.ku.dk (K.T.K.); 2 Engineering Research Center for the Development and Application of Ethnic Medicine and TCM, Guizhou Medical University, No.4 Beijing Road, Yunyan District, Guiyang 550004, China; xuema0111@163.com; 3 Department of Chemistry, Technical University of Denmark, Kemitorvet Building 207, DK-2800 Kgs. Lyngby, Denmark; feniil@kemi.dtu.dk (D.F.); soco@kemi.dtu.dk (S.C.)

**Keywords:** *Gerbera piloselloides*, coumaroyl derivative, protein-tyrosine phosphatase 1B, α-glucosidase, chiral separation, electronic circular dichroism, X-ray

## Abstract

Crude ethyl acetate extract of *Gerbera piloselloides* (L.) Cass. was investigated by dual high-resolution PTP1B/α-glucosidase inhibition profiling and LC-PDA-HRMS. This indicated the presence of a series of unprecedented prenyl- and geranyl-substituted coumarin derivatives correlated with both α-glucosidase and PTP1B inhibitory activity. Repeated chromatographic separation targeting these compounds led to the isolation of 13 new compounds, of which ten could be isolated as both enantiomers after chiral separation. The structures of all isolated compounds were characterized by HRMS and extensive 1D and 2D NMR analysis. The absolute configurations of the isolated compounds were determined by comparison of experimental and calculated electronic circular dichroism spectra. Compound **6** features a rare furan-oxepane 5/7 ring system, possibly formed through addition of a geranyl unit to C-3 of 5-methylcoumarin, representing a new type of geranyl-substituted coumarin skeleton. Compounds **19** and **24** are the first examples of dimeric natural products consisting of both coumarin and chromone moieties.

## 1. Introduction

Diabetes mellitus is a multifactorial disease characterized by insufficient regulation of glucose metabolism, with severe long-term micro- and macrovascular complications such as retinopathy, neuropathy, nephropathy and cardiovascular diseases. More than 463 million people were affected by diabetes worldwide in 2019, and this figure is estimated to increase to 700 million by 2045 [1]. Type 2 diabetes (T2D) comprises more than 90% of all diabetes cases and is characterized by decreased insulin sensitivity in target organs like muscle and adipose tissue as well as reduced pancreatic insulin secretion [2,3]. Thus, management of a relatively stable blood glucose in the interval 5–10 mmol/L by hypoglycemic drugs is of utmost importance for diabetics. One target for management of T2D is protein tyrosine phosphatase 1B (PTP1B) [4,5], which acts as a negative modulator of insulin by dephosphorylation of the insulin receptor and the insulin receptor substrate. α-Glucosidase constitutes another potential T2D target enzyme [6]. It is found in the brush border of the small intestines, where it hydrolyses terminal non-reducing 1,4-linked α-glucose residues to absorbable monosaccharides for managing a low and stable blood glucose level. There are still no clinically approved PTP1B inhibitors for diabetics, and the clinically approved drugs acarbose, miglitol and voglibose are associated with side effects such as flatulence, diarrhea and stomach ache [7]. Thus, there is an unmet need for new PTP1B and α-glucosidase inhibitors as potential T2D drug leads.

*Gerbera piloselloides* (L.) Cass. (Compositae) is a perennial herb commonly found in southwest China. It is mentioned in local traditional medicine books, where it is recommended as a remedy for treating fever, cough, and snake bites [8]. Previous studies have shown that *Gerbera* spp. are rich in coumarins [9,10,11,12,13,14,15,16], with substituents regularly occurring at C-3 and C-4 of the α-pyrone moiety, as seen for dibothrioclinin I and II [11], gerdelavin A [12], and gerberlin A-C [13]. Coumarins from *Gerbera* have been reported to possess anticancer [13,14,15], antioxidant [16], antimicrobial [15], anti-HIV [17], and hypoglycemic activity [18]. However, to date only a few studies of the phytochemistry and the pharmacological activity of *G. piloselloides* and its constituents have been published [11,16,19], and this species therefore holds promise for discovery of new natural products with pharmacological activity.

The ability to pinpoint the constituent(s) correlated with one or more bioactivities in a complex extract can significantly speed up the identification of bioactive natural products. One way of achieving this is by microfractionation of the eluate from analytical-scale HPLC separation of crude extracts followed by assaying of the material in each well with selected assay(s). The results expressed as percentage inhibition are then plotted at their respective retention time to provide a high-resolution inhibition profile (biochromatogram) with a typical resolution of 4 to 10 datapoints per min. The biochromatogram can be represented underneath the chromatographic traces, which allows direct pinpointing of HPLC peaks correlated with peaks in the biochromatogram. This approach has been successfully used to accelerate identification of α-glucosidase [20], α-amylase [21], aldose reductase [22], and PTP1B [23] inhibitors as well as radical scavengers [24], using either single [25], dual [26], triple [22], or quadruple [27] high-resolution inhibition profiling.

Here, we report the combined use of LC-PDA-HRMS and dual high-resolution PTP1B/α-glucosidase inhibition profiling, that for the first time directly pinpoints α-glucosidase and PTP1B inhibitors in *G. piloselloides*. This was used to guide isolation towards a series of unusual prenyl- and geranyl-substituted coumarin derivatives with hitherto unprecedented skeletons - including dimeric coumarin derivatives formed by Diels-Alder cycloadditions. Full structure elucidation by 2D NMR spectroscopy and assignment of absolute configuration by electronic circular dichroism spectroscopy of these complex natural products provide important reference data for future studies of related structures.

## 2. Results

**Figure 1:**
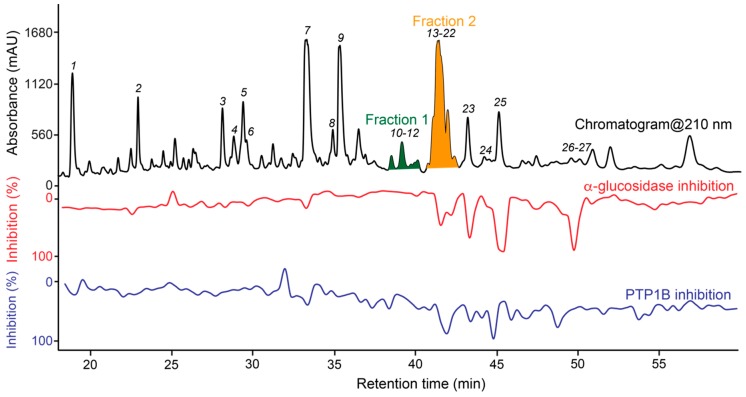
Dual high-resolution PTP1B/α-glucosidase inhibition profile of the crude ethyl acetate extract of *Gerbera piloselloides* shown with the UV chromatogram at 210 nm.

Crude ethyl acetate extract of *Gerbera piloselloides* showed moderate inhibition of PTP1B and α-glucosidase with IC_50_ values of 82.8 ± 0.96 μg/mL and 35.9 ± 0.03 μg/mL, respectively. Thus, two repeated analytical-scale HPLC separations were microfractionated into two 96-well microplates each, which after evaporation of the solvent and assaying using PTP1B and α-glucosidase, resulted in the dual high-resolution PTP1B/α-glucosidase inhibition profile (biochromatogram) shown in Figure 1.

The biochromatogram shows that constituents in peaks *7* and *13*–*27* are correlated with both PTP1B and α-glucosidase inhibitory activity. LC-HRMS analysis of the material eluted in the regions marked fraction F1 (dark green) and F2 (orange) implied that most of the constituents eluting in these regions have the same molecular formula C_20_H_22_O_3_. However, the material eluted with Fraction 1 did not show correlation with inhibitory activity in the biochromatogram. To compare active and non-active compounds with the same molecular formula, Fraction 1 and 2 were selected for further investigation together with the constituents eluted with peaks *1*–*27*.

**Figure 2:**
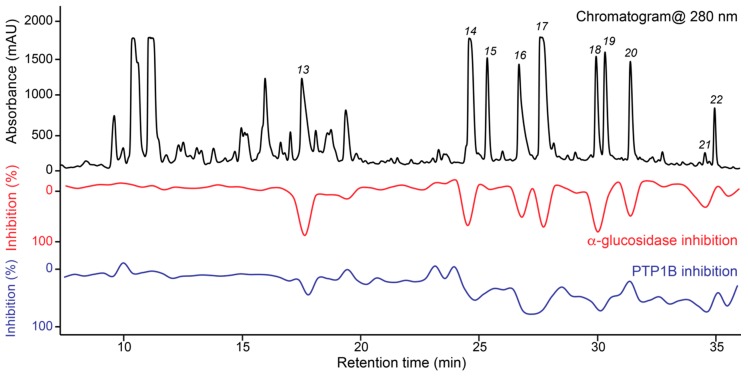
Dual high-resolution PTP1B/α-glucosidase inhibition profile of Fraction 2 of *Gerbera piloselloides* shown with the UV chromatogram at 280 nm.

Material eluted with peaks *1*-*27* and fractions 1 and 2 were collected manually by preparative-scale HPLC. Fraction 2 was subsequently separated using an analytical-scale pentafluorophenyl (PFP) HPLC column, and two successive separations were microfractionated. The dual high-resolution inhibition profile of F2 is shown in Figure 2. This shows that the constituents eluting as peaks *13*, *14* and *16*–*21* are correlated with α-glucosidase and/or PTP1B inhibitory activity. Thus, the extract seems to contain multiple new compounds correlated with inhibitory activity towards both PTP1B and α-glucosidase - one of many unique features of natural products.

### Structure Elucidation of Prenyl- and Geranyl Coumarin Derivatives

Based on information from the two biochromatograms shown in Figure 1 and Figure 2, material correlated with bioactivity as well as material suggested by LC-PDA-HRMS to be prenyl- or geranyl coumarin derivatives were isolated by preparative-scale HPLC followed by analytical-scale HPLC as detailed in the Materials and Methods section. This led to isolation of the new compounds **2**, **5**, **6**, **8**, **10**, **11**, **14**, **15**, **17**–**19**, and **23**–**25** as well as identification of the known compounds marmesin (**1**) [28], 7-demethylsuberosin (**3**) [29], apigravin (**4**) [30], bothrioclinin (**7**) [31], (+)-2-[(2*R*)-6-acetyl-2,3-dihydro-5-hydroxybenzofuran-2-yl]- prop-2-enyl 15-methylbutanoate (**8**) [29], 3,5-bis-(isopent-2-en-1-yl)-4-hydroxyacetophenone (**9**) [32], 6-acetyl- 2,2-dimethyl-8-(3-methylbut-2-en-1-yl)-2*H*-chromene (**12**) [33], and mutisicoumarin B (**16**) [34] by comparison of their ^1^H NMR data with data from the literature. Retention times, HRESIMS, and ^1^H NMR data for **1**, **3**, **4**, **7**–**9**, and **12** are given in Table S1, Supplementary Material.

Compound **2** showed a [M + H]^+^ ion with *m*/*z* 277.1083 (C_15_H_17_O_5_^+^, ΔM −4.5 ppm), which suggests the molecular formula C_15_H_16_O_5_. The ^1^H NMR spectrum shows characteristic signals for a 1,2,3-trisubstituted benzene ring δ 7.12 (d, 7.4 Hz, H-6), δ 7.45 (dd, 8.3, 7.4 Hz, H-7) and δ 7.19 (d, 8.3 Hz, H-8)), two methyl singlets (δ 1.52, CH_3_-13 and δ 1.57, CH_3_-14) with HMBC correlations to C-4 (δ 163.7) and C-11 (δ 83.1), a methyl singlet (δ 2.72 CH_3_-12) with HMBC correlations to C-4a (δ 115.5), C-5 (δ 139.0), and C-6 (δ 128.9), and two oxymethine signals at δ 4.58 (d, 4.1 Hz, H-9) and δ 3.75 (d, 4.1 Hz, H-10) with HMBC correlations to C-3 (δ 101.8) and C-2 (δ 164.7).

**Figure 3:**
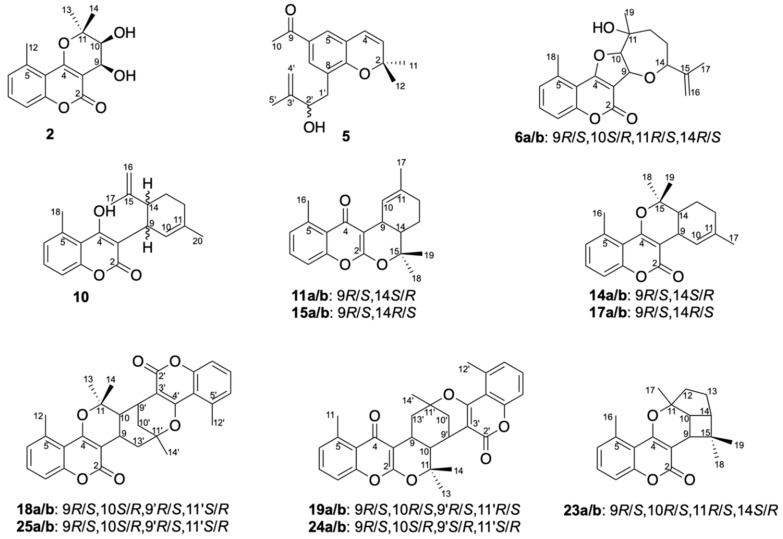
New prenyl- and geranyl coumarin derivatives **2**, **5**, **6**, **8**, **10**, **11**, **14**, **15**, **17**-**19** and **23**–**25** identified in the crude extract of *Gerbera piloselloides*.

**Figure 4:**
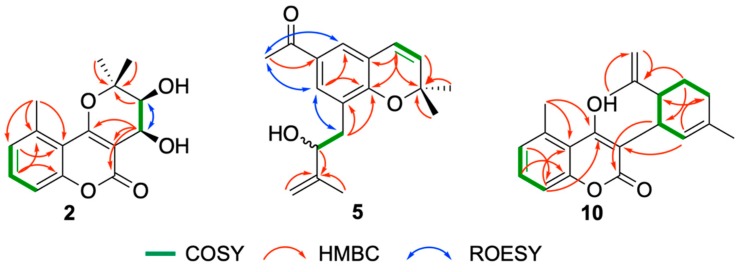
Selected COSY, ROESY and HMBC correlations of compounds **2**, **5**, and **10**.

This suggested **2** to be the 9,10-dihydroxylated analogue of bothrioclinin (**7**) (Figure 3), and COSY, ROESY, HSQC and HMBC correlations, of which selected correlations are shown in Figure 4, confirmed this and allowed the full assignment of ^1^H and ^13^C NMR signals provided in Table 1 and Table 2. The relative configuration of **2** was established to be 9S*,10S* based on the coupling constant (^3^*J* = 4.1 Hz) between H-9 and H-10, which suggests a *cis* configuration as also reported for *cis*-khellactone [35]. Finally, the absolute configuration of **2** was assigned 9*S*,10*S* by comparing the ECD spectrum of **2** (Figure S8, Supplementary Material) with ECD data of *cis*-khellactone [35]. Compound **2** is a new compound for which the name gerbeloid 1 is proposed.

Compound **5** showed a [M + H]^+^ ion with *m*/*z* 287.1637 (C_18_H_23_O_3_^+^, ΔM 1.6 ppm), which suggests the molecular formula C_18_H_22_O_3_. The ^1^H NMR spectrum of **5** displays characteristic signals for the *cis*-coupled H-3 (δ 5.74, d, 9.8 Hz) and H-4 (δ 6.42, d, 9.8 Hz), the *meta*-coupled H-5 (δ 7.56, d, 2.1 Hz) and H-7 (δ 7.70, d, 2.1 Hz), a methyl signal for CH_3_-10 (δ 2.52, s), and two methyl singlets for CH_3_-11 (δ 1.46, s) and CH_3_-12 (δ 1.47, s). This confirms that **5** possesses a 2,2-dimethyl-2*H*-chromene core skeleton with an acetyl at C-6 and a second substituent at C-8, which based on the residual molecular formulas, is in agreement with a hydroxyisoprenyl unit (C_5_H_9_O). The COSY spectrum revealed a CH_2_-CH-O spin system, and HMBC correlations from H-1′ to C-8′ and C-8a′ as well as from H-2′, H-4′ and H-5′ to C-3′ confirmed a 2-hydroxy-3-methylbut-3-en-1-yl unit at C-8. Compound **5** gradually degraded in solution, and it was therefore not possible to determine the absolute configuration at C-2′. The structure of **5** is shown in Figure 3, selected COSY, ROESY and HMBC correlations are shown in Figure 4, ^1^H NMR, COSY, ROESY, HSQC and HMBC spectra are provided in Figures S33–S38, Supplementary Material, and fully assigned ^1^H and ^13^C NMR data are provided in Table 1 and Table 2. Compound **5** is a new compound for which the name gerbeloid 2 is proposed.

Compound **6** showed a [M + H]^+^ ion with *m*/*z* 343.1527 (C_20_H_23_O_5_^+^, ΔM 3.8 ppm) and a [M + Na]^+^ ion with *m*/*z* 365.1359 (C_20_H_22_O_5_Na^+^, ΔM 0.1), which suggested the molecular formula C_20_H_22_O_5_ with a hydrogen deficiency index of 10. The ^1^H NMR spectrum of **6** shows characteristic signals of a 5-methylcoumarin moiety substituted at C-3 and C-4 (δ 7.26 (d, 8.4 Hz, H-6), δ 7.56 (dd, 8.4, 7.5 Hz, H-7), δ 7.20 (d, 7.5 Hz, H-8), and δ 2.71 (s, CH_3_-18)) as also seen for **2** and **7**, *vide supra*; leaving a residual molecular formula of C_10_H_16_O_3_ and three hydrogen deficiency index numbers for the substituents at C-3 and C-4. COSY correlations were observed between oxymethines H-9 ↔ H-10 and between H-12 ↔ H-13 ↔ H-14, and with HMBC correlations from H-9 to C-14 and from H-14 to C-9 as well as from H-10 and H-12 to the oxymethine C-11, this established the oxepane ring. 

Furthermore, the prop-1-en-2-yl unit at C-14 was established by HMBC correlations from H-14 to C-15, C-16 and C-17, and the methyl group at C-11 was established by HMBC correlations from H-19 to C-11. Finally, the ether linkage between C-4 and C-10 to form the 2,3-dihydrofuran moiety was inferred from the downfield shift of C-4 (δ 171.0) in **6** compared to **2** (δ 164.7) and **7** (δ 161.6), as also reported by Qiang and co-workers for gerbelin 3 [13], as well as the need to establish a ring closure as determined from the hydrogen deficiency index. Thus, the structure of **6** contains a rare furan-oxepane 5/7 ring system formed by a geranyl unit.

**Figure 5:**
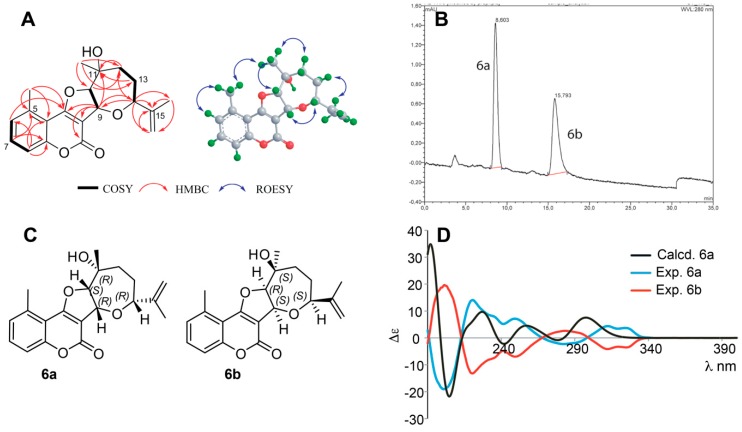
(**A**): Key 2D NMR correlations of **6**. (**B**): Chiral separation of 6 showing two enantiomers in a 1:1 ratio. (**C**): structures of **6a** and **6b** with the absolute configuration of stereocenters. (**D**): Calculated ECD spectrum of **6a** and experimental ECD spectra of **6a** and **6b**.

The relative configuration 9R*,10S*,11R*,14R* was established based on the *cis* coupling constants between H-9 and H-10 (^3^*J* = 7.6 Hz) as well as the ROESY correlations H-9 ↔ H-14, and between H-10 ↔ CH_3_-19 (Figure 5A). An ECD spectrum of **6** indicated the material to be an enantiomeric mixture, and subsequent chiral separation showed two enantiomers (**6a** and **6b**) in a 1:1 ratio (Figure 5B). The two enantiomers were isolated, and their experimental ECD spectra (Figure 5D) were as expected completely opposite. After a conformational search, the 10 lowest-energy conformations of **6a** were obtained by energy minimization at the B3LYP/6-311G(d,p) level in CH_3_CN (Figure S4 and Tables S2 and S3, Supplementary Material) and their ECD spectra calculated. The total Boltzmann-averaged ECD spectrum is shown in Figure 5D, confirming **6a** to be 9*R*,10*S*,11*R*,14*R* and **6b** to be 9*S*,10*R*,11*S*,14*S*. (Figure 5C), with an enantiomeric similarity index (Δ_ESI_) of 0.80. Selected COSY, ROESY and HMBC correlations are shown in Figure 5A, ^1^H NMR, COSY, ROESY, HSQC and HMBC spectra are provided in Figures S39–S44, Supplementary Material, and fully assigned ^1^H and ^13^C NMR data are provided in Table 1 and Table 2. Compounds **6a** and **6b** are new compounds for which the names gerbeloid 3a and gerbeloid 3b are proposed.

Compound **8** showed a [M + H]^+^ ion with *m*/*z* 319.1535 (C_18_H_23_O_5_^+^, ΔM 1.6 ppm), which suggested the molecular formula C_18_H_22_O_5_ with a hydrogen deficiency index of 8. The ^1^H NMR data of **8** are almost identical with data reported for 2-[(2S*)-6-acetyl-2,3-dihydro-5-hydroxybenzofuran-2-yl]prop-2-enyl 3-methylbutanoate [29,36]. There is no optical rotation reported for this compound, but the optical rotation [α]^25^_D_ + 60.8 (*c* 7.2 mM MeOH) of **8** has the opposite sign as the closely reported compounds (−)-2*S*-6,12-dihydroxytremetone and (−)-(*S*)-2-(5-acetyl-6-hydroxy-2,3-dihydrobenzofuran-2-yl)allyl isobutyrate [37]. Furthermore, the experimental ECD spectrum of **8** shows positive Cotton effects around 195 and 265 nm and a negative Cotton effect around 225 nm (Figure S8, Supplementary Material), whereas the experimental and calculated ECD spectra of (−)-2*S*-6,12-dihydroxytremetone show negative Cotton effects around 205 and 280 nm and a positive Cotton effect around 240 nm [37].

Thus, **8** is tentatively assigned the *R* configuration at C-2, and 2-[(2S*)-6-acetyl-2,3-dihydro-5-hydroxybenzofuran-2-yl]prop-2-enyl 3-methylbutanoate, previously isolated from the closely related *Leibnitzia anandria* Sch. Bip. [36] and *Gerbera saxatilis* [29], should most likely also be assigned the *R* configuration at C-2 based on biosynthetic arguments. Full assignment of the HRMS and ^1^H NMR data are included in Table S1, Supplementary Material, and ^1^H NMR, COSY, ROESY, HSQC and HMBC spectra are provided in Figures S48–S52, Supplementary Material.

HRESIMS data for the material eluted with peaks *10–11*, *14–15* and *17* showed [M + H]^+^ ions suggesting that these compounds all have the formula C_20_H_22_O_3_. The 1D and 2D NMR spectra of **14** and **17** (Figures S72–S77 and Figures S88–S93, Supplementary Material) suggest structures consisting of the same 5-methylcoumarin moiety substituted at C-3 and C-4 as seen in **2**, **6** and **7**. Furthermore, detailed analysis of the NMR spectra suggested **14** and **17** to contain the same pyrano[3,2-*c*]coumarin core skeleton that was obtained as product of the microwave-accelerated domino Knoevenagel hetero Diels-Alder reaction between 4-hydroxycoumarin and citronellal [38], but with a methyl group at C-5 and a double bond between C-10 and C-11 (Figure 3).

**Figure 6:**
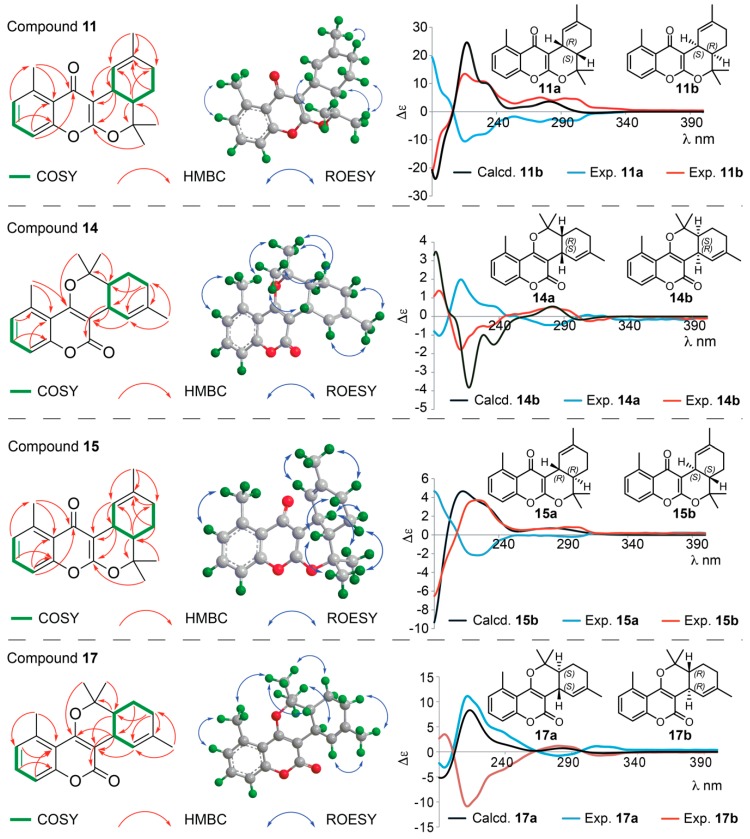
**Left**: Key COSY and HMBC correlations of **11**, **14**, **15** and **17**. **Middle**: Key ROESY correlations to establish relative configuration of one of the enantiomers of **11**, **14**, **15** and **17**. **Right**: Calculated ECD spectra of **11b**, **14b**, **15b** and **17a** and experimental ECD spectra of the enantiomers **11a/b**, **14a/b**, **15a/b** and **17a/b**. The enantiomeric structures of **11**, **14**, **15** and **17** shown with absolute configuration of the stereocenters determined based on the ECD data.

HMBC and COSY correlations support the structures of **14** and **17**, with HMBC correlations from H-18 to C-4, from H-9 to the carbonylic C-2 and the olefinic C-3, and from H-14 to the oxygenated C-15 confirming the pyrano[3,2-*c*]coumarin skeleton (Figure 6). Compounds **14** and **17** are diastereomers, and the vicinal coupling ^3^*J*_H9,H14_ = 6.8 Hz for **14** and a ROESY correlation between H-9 and H-14, support these two protons to be *cis* in **14**; whereas the vicinal coupling ^3^*J*_H9,H14_ = 10.9 Hz for **17** as well as ROESY correlations between H-9 and CH_3_-18 and between H-14 and CH_3_-19 support these two protons to be *trans* in **17**. Both **14** and **17** are racemic mixtures, as seen from the 1:1 peak ratio upon chiral separation (Figures S1 and S2, Supplementary Material), and separation of the two enantiomers allowed comparison of their experimental and calculated ECD spectra. The absolute configurations are therefore assigned 9*R*,14*S* for **14a** and 9*S*,14*R* for **14b** (with Δ_ESI_ of 0.94), and 9*R*,14*R* for **17a** and 9*S*,14*S* for **17b** (with Δ_ESI_ of 0.86), as depicted in Figure 6. ^1^H NMR, COSY, ROESY, HSQC and HMBC spectra are provided in Figures S72–S77 and Figures S88–S93, Supplementary Material, and fully assigned ^1^H and ^13^C NMR data are provided in Table 2 and Table 3. Compounds **14a**/**b** and **17a**/**b** are new compounds for which the names gerbeloid 6a/gerbeloid 6b and gerbeloid 8a/gerbeloid 8b, respectively, are proposed.

Synthesis of the pyrano[3,2-*c*]coumarin core skeleton via the microwave-accelerated domino Knoevenagel hetero Diels-Alder reaction between 4-hydroxycoumarin and citronellal [38], also provided products with the pyrano[2,3-*b*]chromen-4-one core skeleton. This is because the final cyclisation of the chromane-2,4-dione intermediate can occur via two different 4 + 2 cycloadditions, and one can predict that the potential geran-1-ylidene-chromane-2,4-dione intermediate for biosynthesis of **14** and **17** (lactones) could also lead to formation of the isomeric keto products with the pyrano[2,3-*b*]chromen-4-one core skeleton (Scheme S1, Supplementary Material). The ^13^C NMR carbonyl resonances for C-4 in both **11** and **15** in fact suggest that the two compounds are chromone (keto) products, where C-4 is at δ 182.2 in **11** and δ 181.8 in **15** (Table 2). Additionally, HMBC correlations from CH_3_-18 and H-9 to C-2, from H-9 and H-10 to C-3 and from H-8 and CH_3_-16 to C-4 in both compounds support the pyrano[2,3-*b*]chromen-4-one core skeleton (Figure 6). Key COSY and HMBC correlations used for structure elucidation of **11** and **15** are shown in Figure 6. The relative configurations of **11** (9R*,14S*) and **15** (9R*,14R*) were established based on the vicinal coupling between H-9 and H-14 (^3^*J*_H9,H14_ = 6.7 Hz *cis* in **11** and ^3^*J*_H9,H14_ = 10.8 Hz *trans* in **15**) (Table 2 and Table 3) and ROESY correlations (Figure 6), showing that **11** and **15** are diastereomers. By chiral resolution, two enantiomeric pairs **11a**/**b** of **11** and **15a**/**b** of **15** were obtained in a 1:1 ratio (Figure S1, Supplementary Material), and ECD spectra were measured of each of the separated enantiomers. Comparison of the experimental and calculated ECD spectra (Figure 6) allowed assignment of the absolute configurations to be 9*R*,14*S* for **11a** and 9*S*,14*R* for **11b** (with Δ_ESI_ of 0.98), and 9*R*,14*R* for **15a** and 9*S*,14*S* for **15b** (with Δ_ESI_ of 0.95). ^1^H NMR, COSY, ROESY, HSQC and HMBC spectra of **11** and **15** are provided in Figures S61–S66 and Figures S78–S83, Supplementary Material, and ^1^H and ^13^C NMR data are provided in Table 2 and Table 3. Compounds **11a**/**b** and **15a**/**b** are new compounds for which the names gerbeloid 5a/gerbeloid 5b and gerbeloid 7a/gerbeloid 7b, respectively, are proposed.

Analysis of the 1D and 2D NMR data of **10** shows the 5-methylcoumarin skeleton (δ 6.99, H-6, δ 7.29, H-7, δ 7.03, H-8 and δ 2.76, CH_3_-18) as seen in **14** and **17**, but with an additional double bond (δ 150.0, C-15 and δ 110.6, C-16) and olefinic methylene group (δ 4.52 and 4.46, H-16) appearing and one methyl group disappearing (Table 1 and Table 2). Thus, **10** was identified as 4-hydroxy-5-methyl-3-(3-methyl-6-(prop-1-en-2-yl)cyclohex-2-en-1-yl)-2*H*-chromen-2-one, the 10,11-dehydroanalog of an intramolecular domino Knoevenagel ene adduct previously reported [37]. Key COSY and HMBC correlations used for structure elucidation are shown in Figure 4, and **10** is tentatively assigned the 9S*, 14R* configuration based on the coupling constants between H-9 and H-14 (^3^*J*_H9,H14_ = 10.5 Hz *trans*) as also reported for the synthetic analog [38]. However, keto-enol tautomerism did neither allow chiral separation or acquisition of ECD spectra, and **10** was gradually degraded in solution. ^1^H NMR, COSY, ROESY, HSQC and HMBC spectra are provided in Figures S56–S60, Supplementary Material, and fully assigned ^1^H and ^13^C NMR data are provided in Table 1 and Table 2. Compound **10** is a new compound for which the name gerbeloid 4 is proposed.

**Figure 7:**
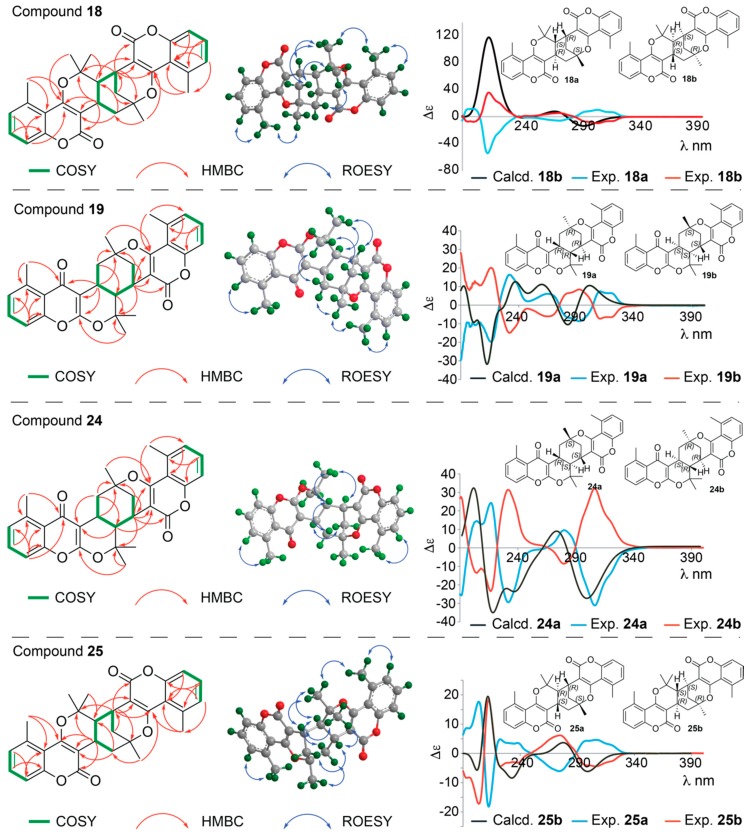
**Left**: Key COSY and HMBC correlations of **18**, **19**, **24** and **25**. **Middle**: Key ROESY correlations used to establish relative configuration of **18**, **19**, **24** and **25**. **Right**: Calculated ECD spectra of **18b**, **19a**, **24a** and **25b** and experimental ECD spectra of the enantiomers **18a/b**, **19a/b**, **24a/b** and **25a/b**. The enantiomeric structures of **18**, **19**, **24** and **25** are shown with absolute configuration of the stereocenters determined based on the ECD data.

HRESIMS data for the material eluted with peaks *18*-*19* and *24*-*25* provided [M + H]^+^ ions, showing that these compounds all have the formula C_30_H_28_O_6_ (see details in the Materials and Methods section), suggesting them to be dimers of prenyl-substituted coumarin derivatives. Furthermore, chemical shift values of C-4 (δ 181.7 and 181.9) (Table 4) in **19** and **25**, respectively, showed each of these to consist of a coumarin (lactone) unit and a chromen-4-one (keto) unit, whereas **18** and **25** consist of two coumarin units each. Detailed analyses of 1D and 2D NMR data for **18** and **25** show that they consist of two pyrano[3,2-*c*]coumarin units joined together by bonds between C-9 and C-13′ and between C-10 and C-9′, and comparison with NMR data reported for dibothrioclinin I and II [11], show that **18** and **25** are diastereomers of these. This was further supported by COSY and HMBC correlations (key correlations seen in Figure 7).

The core cyclohexane of **18** was identified as a chair conformation with axial-axial couplings between H-9 and H-10 and between H-9 and H-13′_ax_ (*J*_H9,H10_ = *J*_H9,H-13′ax_ = 12 Hz) and 1,3-diaxial ROEs between H-10, H-10′_ax_, and H-13′_ax_. With equatorial position of H-9′ and CH_3_-14′, as seen from coupling constants and ROEs to H-10, H-10′_ax_, and H-13′_ax_, the relative configuration of **18** was established as 9R*,10S*,9′R*,11′S*. Contrary to this, the core cyclohexane of **25** was identified as a boat conformation, with very strong ROE correlation between H-9 and H-10′_endo_ (and ROEs to H-9′, H-9′, H-13′_eq_ and CH_3_-14′ on the same side of the plane) and 1,3-diaxial ROEs between H-10 and H-13′_ax_. The relative configuration of **25** was therefore established as 9R*,10S*,9′S*,11′R* (Figure 7). Two pairs of enantiomers (**18a** and **18b**, **25a** and **25b**) with the peak area ratios 1:1 were separated by chiral HPLC chromatography (Figures S2 and S3, Supplementary Material). Comparison of the experimental and calculated ECD spectra (Figure 7) allowed assignment of the absolute configurations to be 9*R*,10*S*,9′*R*,11′*S* for **18a** and 9*S*,10*R*,9′*S*,11′*R* for **18b** (with Δ_ESI_ of 0.93), and 9*R*,10*S*,9′*R*,11′*S* for **25a** and 9*S*,10*R*,9′*S*,11′*R* for **25b** (with Δ_ESI_ of 0.82). ^1^H NMR, COSY, ROESY, HSQC and HMBC spectra are provided in Figures S94–S99 and S118–S123, Supplementary Material, and fully assigned ^1^H and ^13^C NMR data are provided in Table 4 and Table 5. Compounds **18a**/**b** and **25a**/**b** are new compounds for which the names gerbeloid 9a/gerbeloid 9b and gerbeloid 13a/gerbeloid 13b, respectively, are proposed.

**Figure 8:**
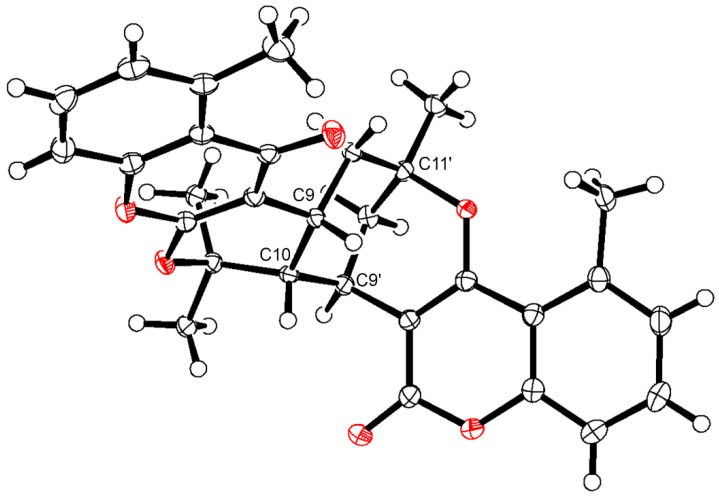
Perspective drawing (ORTEP-3) of **19**. Displacement ellipsoids of the non-hydrogen atoms are shown at the 50% probability level. Hydrogen atoms have been shown as spheres of arbitrary size. Oxygen atoms are red.

The ^1^H NMR data of **19** and **24** show similar patterns for H-6, H-7, H-8 and H-6′, H-7′, H-8′ in the aromatic region as seen for **18** and **25**, showing the methyl groups at C-5 and C-5′. However, as stated above, **19** and **24** each consist of a coumarin (seen from the chemical shift values of δ 163.8 and δ 163.5 for C-2′ and δ 166.3 and δ 163.1 for C-4′ in **19** and **24**, respectively) and a chromen-4-one unit (seen from the chemical shift values of δ 163.3 and δ 163.9 for C-2 and δ 181.7 and δ 181.9 for C-4 in **19** and **24**, respectively). HMBC correlations from CH_3_-18 and H-9 to C-2, from H-9 and H-10 to C-3, and from H-8 and CH_3_-12 to C-4 support the pyrano[2,3-*b*]chromen-4-one skeleton, whereas HMBC correlations from H-9′ to C-2′, H-9′ and H-10 to C-3′, and from CH_3_-14′ to C-4′ support the pyrano[3,2-*c*]coumarin skeleton (Figure 7). Furthermore, HMBC correlations from H-9 to C-11′ and C-13′ as well as from H-10 to C-3′ and C-9′ and from H-9′ to C-10 and C-11 proved the bridging between the pyrano[2,3-*b*]chromen-4-one and the pyrano[3,2-*c*]coumarin skeleton as shown in Figure 3 and Figure 7. Based on analysis of coupling constants and ROESY correlations (Figure 7), **19** was assigned the relative configuration 9R*,10R*,9′R*,11′R* and **24** was assigned the relative configuration 9R*,10S*,9′S*,11′S*. Additionally, X-ray crystal structure analysis of the racemate of **19** yielded the structure shown in perspective drawing (ORTEP-3) [39] in Figure 8. This agrees with the structure established based on the NMR data, confirming the 9R*,10R*,9′R*,11′R* configuration of **19**.

After chiral separation of **19** and **24** (1:1 ratios of enantiomers **19a**, **19b** and **24a**, **24b**, see Figures S2 and S3, Supplementary Material), ECD spectra of both pairs of enantiomers were acquired. These are shown in Figure 7, and comparison with the calculated ECD spectra of **19a** and **24a** allowed assignment of the absolute configurations to be 9*R*,10*R*,9′*R*,11′*R* for **19a** and 9*S*,10*S*,9′*S*,11′*S* for **19b** (with Δ_ESI_ of 0.77), and 9*R*,10*S*,9′*S*,11′*S* for **24a** and 9*S*,10*R*,9′*R*,1′*R* for **24b** (with Δ_ESI_ of 0.87) (Figure 7). ^1^H NMR, COSY, ROESY, HSQC and HMBC spectra are provided in Figures S100–S105 and S112–S117, Supplementary Material, and fully assigned ^1^H and ^13^C NMR data are provided in Table 4 and Table 5. Compounds **19a**/**b** and **24a**/**b** are new compounds for which the names gerbeloid 10a/gerbeloid 10b and gerbeloid 12a/gerbeloid 12b, respectively, are proposed.

**Figure 9:**
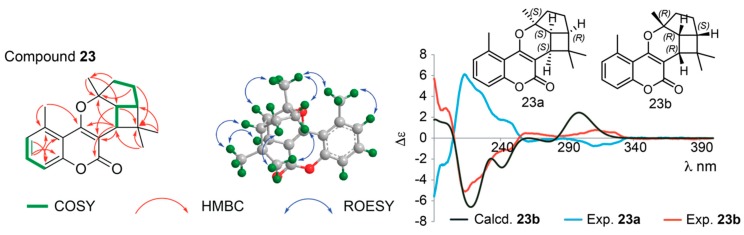
**Left**: Key COSY and HMBC correlations of **23. Middle**: Key ROESY correlations to establish relative configuration of one of the enantiomers of **23**. **Right**: Calculated ECD spectra of **23b** and experimental ECD spectra of the enantiomers **23a/b**. The enantiomeric structures of **23** shown with absolute configuration of the stereocenters determined based on the ECD data.

Compound **23** showed a [M + H]^+^ ion with *m*/*z* 311.1641 (C_20_H_23_O_3_^+^, ΔM 0.2 ppm) and a [M + Na]^+^ ion with *m*/*z* 333.1446 (C_20_H_22_O_3_Na^+^, ΔM 4.5 ppm), which suggested the molecular formula C_20_H_22_O_3_, with a hydrogen deficiency index of 10. The ^1^H and ^13^C NMR spectra of **23** show signals corresponding to H-6, H-7 and H-8 in the aromatic region and the methyl group at C-5 as well as characteristic chemical shifts for C-2 (δ 165.2), C-3 (δ 103.9), and C-4 (δ 163.6) as also observed for **14** and **17**, suggesting they share the same 5-methylcoumarin skeleton. The pyrano[3,2-*c*]coumarin core skeleton is supported by HMBC correlations from CH_3_-16 and CH_3_-17 to C-4, from CH_3_-17 to C-10, and from H-9 to C-2 and C-4 (Figure 9) as well as correlations in the COSY spectrum between H-9 and H-10. In fact, the COSY spectrum showed correlations corresponding to the H-9 ↔ H-10 ↔ H-14 ↔ H-13 ↔ H-12 spin system, and HMBC correlations from H-12 to C-11 and C-17 revealed the cyclopentane ring system, whereas HMBC correlations from H-9, H-14, CH_3_-18 and CH_3_-19 to C-15 and from H-18 and H-19 to C-9 and C-14 revealed the dimethylated cyclobutane ring system. Thus, **23** was identified as a 5-methylcoumarin with a 6/5/4 ring system fused at C-3 and C-4. Two coumarins with similar 6/5/4 ring systems have been reported before [40], but linked to either C-5 and C-6 or C-7 and C-8 of the coumarin moiety; and the NMR data for the 6/5/4 ring system of the previously reported compounds resemble those observed for **23** (Table 2 and Table 3) [40,41]. The coupling constants between H-9 and H-10 (^3^*J* = 9.4 Hz) and between H-10 and H-14 (^3^*J* = 8.1 Hz) are in close agreement with those previously reported for a similar 6/5/4 ring system with H-9, H-10 and H-14 being *cis*, and thus on the same side of the molecule. Similarly, the ROESY correlations between H-9 ↔ H-18 ↔ H-14 ↔ H-13A and H-10 ↔ H-17 ↔ H-12A show these to be on one plane of the molecule, whereas ROESY correlations between H-19 and H12B/H-13B shows these to be on the other plane of the molecule - and thus the relative configuration of **23** is 9R*,10R*,11R*,14S*.

Chiral resolution of **23** gave two enantiomers (**23a** and **23b**) in a 1:1 ratio (Figure S2, Supplementary Material), and comparison of the ECD spectra of **23a** and **23b** with the ECD spectrum calculated for **23b** (Figure 9), allowed assignment of the absolute configuration to be 9*R*,10*R*,11*R*,14*S* for **23a** and 9*S*,10*S*,11*S*,14*R* for **23b**, respectively, with Δ_ESI_ of 0.88. ^1^H NMR, COSY, ROESY, HSQC and HMBC spectra are provided in Figures S106–S111, Supplementary Material, and fully assigned ^1^H and ^13^C NMR data are provided in Table 2 and Table 3. Compounds **23a**/**b** are new compounds for which the names gerbeloid 11a/gerbeloid 11b are proposed.

The material eluted with peaks *14*–*19* and *23*–*25* were correlated with both α-glucosidase and PTP1B inhibitory activity in the high-resolution inhibition profile (Figure 1 and Figure 2). Due to limited amount of material isolated of the racemic mixtures as well as the individual enantiomers after chiral separation, it was not possible to make dilution series to obtain full dose-response curves. However, for the racemic mixtures of **17**–**19**, **24** and **25**, it was possible to test the inhibitory activity at a single concentration between 32.2 and 201.6 μM. The results are provided in Table 6 and show that the tested compounds show weak to moderate inhibitory activity towards both α-glucosidase and PTP1B. 

The reported values may be underestimated, because typically only one enantiomer of a racemic mixture is an active inhibitor of the target enzyme. Thus, isolation of more material and subsequent chiral separation are needed to obtain full dose-response curves for both enantiomers in both the α-glucosidase and the PTP1B assay, as well as for being able to study the mode of inhibition of the many compounds correlated with one or both activities. The only prior study with dual inhibitory activity of PTP1B and α-glucosidase with this kind of compounds, is a recent study with a series of prenylated coumarins from *Angelica decursiva*, showing weak α-glucosidase inhibitory activity with IC_50_ values ranging from 65 to 172 μM, and PTP1B inhibitory activity with IC_50_ values ranging from 5 to 59 μM.

**Figure 10:**
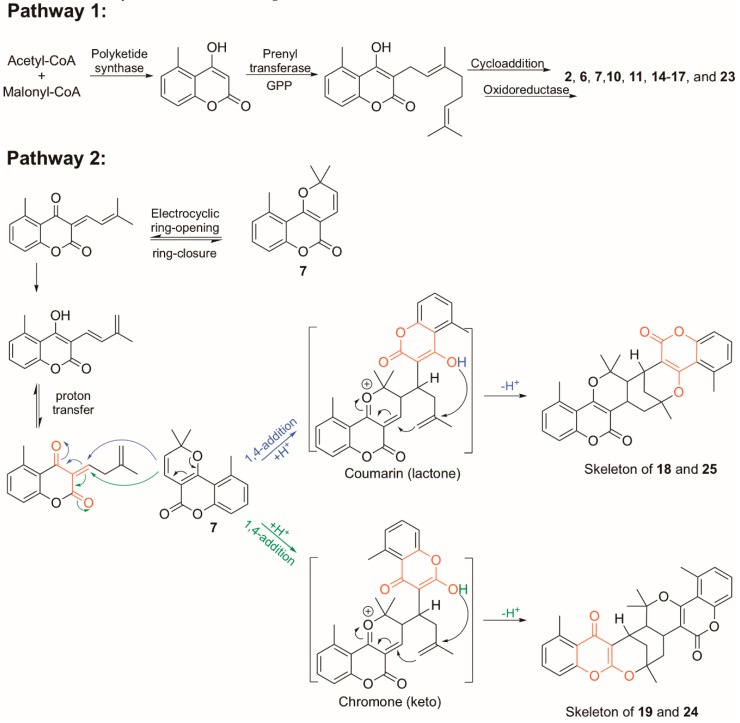
Possible biosynthetic pathway for the prenyl- and geranyl substituted coumarin derivatives isolated in this study.

The coumarin skeleton is synthesized from acetyl-CoA and malonyl-CoA through the polyketide pathway in plants [42]. Inspired by the biosynthetic pathway of tetrahydrocannabinolic acid (THCA) in *Cannabis sativa* [43], the monoterpenoid-coumarins identified in this study are plausibly first added a monoterpenoid chain to C-3 of the coumarin skeleton, catalyzed by prenyl transferase (Pathway 1 in Figure 10). As mentioned above, [4 + 2] cycloaddition reactions have been utilized for the total synthesis of structures similar to **11**, **14**, **15** and **17** (Scheme S1, Supplementary Material) [38], and therefore cyclisations via different Diels-Alder cycloadditions between the monoterpenoid and coumarin, followed by oxidations or reductions catalyzed by oxidoreductase enzymes, could be involved in the formation of **2**, **6**, **7**, **10**, **11**, **14**–**17** and **23** (Figure 10).

There are no prior studies on the biosynthesis of coumarins related to the dimeric coumarin derivatives isolated in this study, but total synthesis of pyranylquinoline alkaloids [44] suggests mechanisms for the plausible biosynthetic route for **18**, **19**, **24** and **25** presented as pathway 2 in Figure 10. In this route, the precursor of **18**, **19**, **24** and **25** is suggested to be **7**, which is seen as a major peak in the ethyl acetate extract of *G. piloselloides* (Figure 1). The key steps in the dimerization of **7** include a ring opening, a hydride shift and a 1,4-addition (Figure 10), followed by 1,4-addition reactions leading to **18** and **25** with two coumarin moieties or **19** and **24** with one coumarin moiety and one chromone moiety in the structure (Figure 10).

## 3. Materials and Methods

### 3.1. Plant Material and Extraction Procedure

Aerial parts of *Gerbera piloselloides* (L.) Cass was collected from Sandaohe Village, Duyun City, Guizhou Province, People′s Republic of China in September 2017 and authenticated by Associate Prof. Chunhua Liu (Provincial Key Laboratory of Pharmaceutics in Guizhou Province, Guizhou Medical University). A voucher specimen (accession number JX20170208) is deposited at the Guizhou Medical University. The air-dried material (1.7 kg) was milled and extracted three times with 2 L ethyl acetate (1 h of ultra-sonication for each extraction). The combined extracts were filtered and concentrated under reduced pressure to give 20 g of dark green crude extract.

### 3.2. Chemicals and Reagents

Recombinant human protein tyrosine phosphatase 1B (PTP1B) (BML-SE332-0050, EC 3.1.3.48) was purchased from Enzo Life Sciences (Farmingdale, NY, USA). α-Glucosidase from *Saccharomyces cerevisiae* type I, lyophilized powder (EC 3.2.1.20), *p*-nitrophenyl α-D-glucopyranoside (*p*-NPG), *p*-nitrophenyl phosphate (*p*NPP), acarbose, sodium phosphate monobasic dihydrate, disodium phosphate dibasic, sodium azide, sodium chloride, dimethyl sulfoxide (DMSO), tris-(hydroxymethyl)-aminomethane (Tris), bis-(2-hydroxyethyl)-imino-tris-(hydroxymethylmethane) (Bis-Tris), *N*,*N*,*N*′,*N*′-ethylenediaminetetraacetate (EDTA), dithiothreitol (DTT), methanol-*d*_4_, and HPLC grade acetonitrile were purchased from Sigma-Aldrich (St. Louis, MO, USA), and formic acid (FA) was purchased from Merck (Darmstadt, Germany). Water was purified by deionization and filtration through a 0.22 μm membrane (Millipore, Billerica, MA, USA).

### 3.3. LC-PDA-HRMS

LC-PDA-HRMS analyses were performed on a model 1260 analytical-scale HPLC system consisting of a G1322A degasser, a G1311A quaternary pump, a G1316A thermostatted column compartment, and a G1315A photodiode-array detector (Agilent, Santa Clara, CA, USA) and a Bruker micrOTOF-Q II high-resolution mass spectrometer (Bruker Daltonik, Bremen, Germany). The column eluate was connected to a T-piece splitter directing 1% of the flow to the mass spectrometer and 99% of the flow the photodiode-array detector. The micrOTOF-Q II mass spectrometer, equipped with an ESI source, was operated in the positive-ion mode using a drying temperature of 200 °C, a capillary voltage of 4100 V, a nebulizer pressure of 2.0 bar, and a drying gas flow of 7 L/min. A solution of sodium formate clusters was injected automatically at the beginning of each run to enable internal mass calibration. Chromatographic separations were performed at 40 °C on a Luna C_18_(2) column, 150 × 4.6 mm i.d., 3 μm particle size, 100 Å pore size (Phenomenex, Torrance, CA, USA) at a flow rate of 0.5 mL/min and using a binary gradient of mobile phase A (CH_3_CN:H_2_O 5:95 + 0.1% FA) and mobile phase B (CH_3_CN:H_2_O 95:5 + 0.1% FA). Chromatographic separation and mass spectrometry were controlled using Hystar ver. 3.2 software (Bruker Daltonik).

### 3.4. Dual High-Resolution PTP1B/α-Glucosidase Inhibition Profiling

High-performance liquid chromatography (HPLC) was performed with an Agilent 1200 series instrument consisting of a G1322A degasser, a G1311A quaternary solvent pump, a G1313A high-performance auto sampler, a G1316A thermostatic column compartment, a G1315B photodiode-array detector, and a G1364C fraction collector, all controlled by Agilent ChemStation software ver. B.03.02. 10 μL of crude extract (50 mg/mL in methanol) was separated at 40 °C on a Phenomenex Luna C_18_(2) column (150 mm × 4.6 mm i.d., 3 μm particle size, 100 Å pore size), using the following binary elution gradient of mobile phase A (CH_3_CN:H_2_O 5:95 + 0.1% FA) and mobile phase B (CH_3_CN:H_2_O 95:5 + 0.1% FA) at a flow rate of 0.5 mL/min: 0 min, 10% B; 30 min, 80% B; 50 min, 100% B and 65 min, 100% B. The eluate from 2 to 65 min was fractionated into 176 wells of two 96-well plates (excluding 8 wells in each plate for the blank control), to give a resolution of 2.8 data points per min. 10 μL of fraction F2 was separated at 40 °C on a Kinetex pentafluorophenyl (PFP) column (150 mm × 4.6 mm i.d., 2.6 μm particle size, 100 Å pore size) (Phenomenex) using the following binary elution gradient of mobile phase C (MeOH:H_2_O 5:95 + 0.1% FA) and mobile phase D (MeOH:H_2_O 95:5 + 0.1% FA) at a flow rate of 0.5 mL/min: 0 min, 60% D; 15 min, 80% D; 30 min, 100% D and 45 min, 100% D. The eluate from 2 to 40 min was fractionated into one 96-well plate (88 wells) to give a resolution of 2.3 data points per min. The fractions in all wells were subsequently evaporated to dryness under reduced pressure, and after PTP1B and a-glucosidase assaying, dual high-resolution PTP1B/α-glucosidase inhibition profiles of crude extract and fraction F2 were constructed as detailed by Wubshet and coworkers [45].

### 3.5. Targeted Isolation of PTP1B/α-Glucosidase Inhibitors and Major Metabolites

Crude ethyl acetate extract (100 mg/mL in MeOH) was separated using a preparative-scale Agilent 1100 HPLC system comprising two G1361A preparative-scale solvent delivery pumps, a G2260A autosampler, and a G1365B multiple wavelength detector, controlled by Agilent ChemStation software ver. B.01.01. Injections (900 μL) were separated at room temperature on a Phenomenex Luna C_18_ column (250 mm × 21.2 mm i.d., 5 μm particle size) using the following binary gradient of mobile phase A and B, *vide supra*, at a flow rate of 17 mL/min: 0 min, 10% B; 30 min, 80% B; 50 min, 100% B, 65 min, 100% B. Manual collection of eluate from 15.8 to 16.5 min (peak *1*), 17.8 to 18.6 min (peak *2*), 25.0 to 25.6 min (peak *3*), 25.8 to 26.2 min (peak *4*), 26.3 to 26.7 min (peak *5*), 26.8 to 28.1 min (peak *6*), 30.1 to 31.3 min (peak *7*), 32.0 to 32.5 min (peak *8*), 32.5 to 33.1 min (peak *9*), 35.7 to 39.6 min (Fraction 1), 39.8 to 41.5 min (Fraction 2), 41.6 to 42.3 min (peak *23*), 42.7 to 43.4 min (peak *24*), 44.2 to 44.9 min (peak *25*) and 53.1 to 54.4 min (peaks *26-27*) afforded 11 mg of peak *1*, 10 mg of peak *2*, 14.2 mg of peak *3*, 9.4 mg of peak *5*, 10.8 mg of peak *6*, 85.6 mg of peak *7*, 9.8 mg of peak *8*, 51.1 mg of peak *9*, 54.2 mg of Fraction 1, 67.0 mg of Fraction 2, 13.1 mg of peak *23*, 7.8 mg of peak *24*, 12.8 mg of peak *25* and 4.4 mg of peaks *26*–*27*. Compounds **5** and **6** were purified from the material eluted as peaks *5* and *6* on the above-mentioned analytical-scale HPLC system and PFP column (10 μL injections of a 25 mg/mL solution, flow rate 0.5 mL/min) using the following binary gradient elution profile of mobile phase C and D: 0 min, 60% D; 15 min, 80% D; 35 min, 90% D; 45 min, 100% D and 47 min, 60% D. This yielded 1.2 mg of **5** (*t*_R_ = 10.6 min) and 1.0 mg of **6** (*t*_R_ = 14.3 min). Compounds **10**-**12** were purified from fraction F1 (10 μL injections of a 20 mg/mL solution, flow rate 0.5 mL/min) using the following binary gradient elution profile of mobile phase C and D: 0 min, 70% D; 10 min, 80% D; 30 min, 81% D; 45 min, 90% D and 55 min, 100% D. This afford 0.7 mg of **10** (*t*_R_ = 17.0 min), 1.2 mg of **11** (*t*_R_ = 18.6 min) and 0.8 mg of **12** (*t*_R_ = 19.5 min). Compounds **14**–**19** were purified from fraction F2 (10 mL injections of a 20 mg/mL solution, flow rate 0.5 mL/min) using the following binary gradient elution profile of mobile phase C and D: 0 min, 60% D; 15 min, 80% D; 30 min, 100% D, 45 min, 100% D. This yielded 1.6 mg of **14** (*t*_R_ = 26.3 min), 1.5 mg of **15** (*t*_R_ = 27.0 min), 2.5 mg of **16** (*t*_R_ = 28.2 min), 2.1 mg of **17** (*t*_R_ = 29.1 min), 1.2 mg of **18** (*t*_R_ = 31.5 min), and 1.7 mg of **19** (*t*_R_ = 31.8 min). Compounds **23**, **24** and **25** were purified after repeated separation of the material eluted as peaks *23*, *24* and *25* (15 mL injections of a 25 mg/mL solution) using the following binary gradient of mobile phase C and D as indicated after each compound: **23** (*t*_R_ = 28.4 min): 0 min, 60% D; 15 min, 80% D; 30 min, 100% D; 45 min, 100% D and 47 min, 60% D; **24** (*t*_R_ = 26.0 min) and **25** (*t*_R_ = 27.0 min): 0 min, 70% D; 10 min, 90% D; 35 min, 95% D; 45 min, 100% D and 55 min, 100% D. This gave 1.5 mg of **23**, 1.1 mg of **24** and 1.7 mg of **25**.

### 3.6. Chiral Separation of Enantiomers

Chiral resolution of **6**, **15**, **18**, **19**, **23**, **24** and **25** was performed at room temperature on a Dionex Ultimate instrument consisting of a LPG-3200BX pump and a MWD-3000SD UV detector, both controlled by Chromeleon software ver. 6.80 (Thermo Fisher Scientific, Waltham, Ca, USA), and with a Rheodyne 9725I injector with 10 μL loop. Enantiomers of compounds **6**, **15**, **18**, **19**, **23** and **24** were separated at a flow rate of 1 mL/min on a Chiralpak AD-H column (250 × 4.6 mm i.d., 5.0 μm particle size) (Chiral Technologies, West Chester, PA, USA) isocratically eluted with the following mixtures of isopropanol (mobile phase E) and heptane (mobile phase F): 20% E and 80% F for **6**; 1% E and 99% F for **15**; 10% E and 90% F for **18**, **19** and **24**; 5% E and 95% F for **23** and **24**. Enantiomers of compound **14** were separated at a flow rate of 0.8 mL/min on a Chiralpak OD-H column (250 × 4.6 mm i.d., 5.0 μm particle size) (Chiral Technologies) isocratically eluted at room temperature with 2% E and 98% F. Enantiomers of compounds **11** and **17** were separated at a flow rate of 1 mL/min on a Lux Amylose-2 column (250 × 4.6 mm i.d., 5.0 μm particle size) (Chiral Technologies) isocratically eluted with 1% E and 99% F. The chiral resolution of ten pairs of enantiomers gave 0.40 mg/0.40 mg of **6a/b**, 0.15 mg/0.15 mg of **11a/b**, 0.20 mg/0.20 mg of **14a/b**, 0.20 mg/0.17 mg of **15a/b**, 0.50 mg/0.40 mg of **17a/b**, 0.17 mg/0.20 mg of **18a/b**, 0.20 mg/0.20 mg of **19a/b**, 0.19 mg/0.15 mg of **23a/b**, 0.30 mg/0.20 mg of **24a/b** and 0.18 mg/0.20 mg of **25a/b**.

Compound **2**: white, amorphous powder; insufficient material available for optical rotation; HPLC-UV (MeOH in H_2_O + 0.1% FA) λ_max_ 203, 230, 282-293 nm; ^1^H and ^13^C NMR data, see Table 1 and Table 3; ECD (*c* 9.0 mM, CH_3_CN) λ_max_ (Δε) 207 (−7.1), 223.5 (+1.02), 264 (+0.31), 294 (+0.91) nm; (+)HRESIMS *m*/*z* [M + H]^+^ calcd for C_15_H_17_O_5_^+^, 277.1071, found 277.1083, ΔM −4.5 ppm.

Compound **5**: white, amorphous powder; insufficient material available for optical rotation; HPLC-UV (MeOH in H_2_O + 0.1% FA) λ_max_ 199, 260, 294 nm; ^1^H and ^13^C NMR data, see Table 1 and Table 3; (+)HRESIMS *m*/*z* [M + H]^+^ calcd for C_18_H_23_O_3_^+^, 287.1642, found 287.1637, ΔM +1.6 ppm; [M + Na]^+^ calcd for C_18_H_22_O_3_Na^+^, 309.1461, found 309.1463, ΔM −0.6 ppm.

Compound **6**: white, amorphous powder; insufficient material available for optical rotation; HPLC-UV (MeOH in H_2_O + 0.1% FA) λ_max_ 198, 212, 248, 348 nm; ^1^H and ^13^C NMR data, see Table 1 and Table 3; (+)HRESIMS *m*/*z* [M + H]^+^ calcd for C_20_H_23_O_5_^+^, 343.1540, found 343.1527, ΔM +3.8 ppm; [M + Na]^+^ calcd for C_20_H_22_O_5_Na^+^, 365.1359, found 365.1355, ΔM +1.2 ppm.

Compound **6a:** ECD (*c* 5.8 mM, CH_3_CN) λ_max_ (Δε) 201 (−19.0), 220 (+14.0), 250 (+7.1), 282 (−2.2), 312 (+4.4), 325 (+3.6) nm.

Compound **6b:** ECD (*c* 5.8 mM, CH_3_CN) λ_max_ (Δε) 201 (+19.0), 220 (−14.0), 250 (−7.1), 282 (+2.2), 312 (−4.4), 325 (−3.6) nm.

Compound **8**: white, amorphous powder; [α]_D_^25^ + 60.8 (*c* 7.2 mM MeOH); HPLC-UV (MeOH in H_2_O + 0.1% FA) λ_max_ 204, 235, 266, 365 nm; ^1^H NMR data, see Table S1, Supplementary Material; ECD (*c* 6.3 mM, CH_3_CN) λ_max_ (Δε) 195 (+17.5), 223.5 (−8.2), 266.1 (+2.3) nm; (+)HRESIMS *m*/*z* [M + H]^+^ calcd for C_18_H_23_O_5_^+^, 319.1540, found 319.1535, ΔM +1.6 ppm.

Compound **10**: white, amorphous powder; insufficient material available for optical rotation; HPLC-UV (MeOH in H_2_O + 0.1% FA) λ_max_ 207, 225-237, 294, 310 nm; ^1^H and ^13^C NMR data, see Table 1 and Table 3; (+)HRESIMS *m*/*z* [M + H]^+^ calcd for C_20_H_23_O_3_^+^, 311.1642, found 311.1644, ΔM −0.7 ppm; [M + Na]^+^ calcd for C_20_H_22_O_3_Na^+^, 333.1461, found 333.1460, ΔM +0.3 ppm.

Compound **11**: white, amorphous powder; insufficient material available for optical rotation; HPLC-UV (MeOH in H_2_O + 0.1% FA) λ_max_ 207, 229, 275, 299 nm; ^1^H and ^13^C NMR data, see Table 1 and Table 3; (+)HRESIMS *m*/*z* [M + H]^+^ calcd for C_20_H_23_O_3_^+^, 311.1642, found 311.1644, ΔM −0.7 ppm; [M + Na]^+^ calcd for C_20_H_22_O_3_Na^+^, 333.1461, found 333.1460, ΔM +0.3 ppm.

Compound **11a:** ECD (*c* 2.4 mM, CH_3_CN) λ_max_ (Δε) 193 (+4.0), 223 (−2.2), 300 (−0.2) nm.

Compound **11b:** ECD (*c* 2.4 mM, CH_3_CN) λ_max_ (Δε) 193 (−6.5), 223 (+3.7), 300 (+0.8) nm.

Compound **12**: white, amorphous powder; insufficient material available for optical rotation; HPLC-UV (MeOH in H_2_O + 0.1% FA) λ_max_ 200, 231, 260, 300–310 nm; ^1^H NMR data, see Table S1, Supplementary Material; (+)HRESIMS *m*/*z* [M + H]^+^ calcd for C_18_H_23_O_2_^+^, 271.1693, found 271.1701, ΔM −3.1 ppm; [M + Na]^+^ calcd for C_18_H_22_O_2_Na^+^, 293.1512, found 293.1515, ΔM −1.0 ppm.

Compound **14**: white, amorphous powder; insufficient material available for optical rotation; HPLC-UV (MeOH in H_2_O + 0.1% FA) λ_max_ 206, 227, 283-306, 319 nm; ^1^H and ^13^C NMR data, see Table 1 and Table 3; (+)HRESIMS *m*/*z* [M + H]^+^ calcd for C_20_H_23_O_3_^+^, 311.1642, found 311.1644, ΔM −0.7 ppm; [M + Na]^+^ calcd for C_20_H_22_O_3_Na^+^, 333.1461, found 333.1460, ΔM +0.3 ppm.

Compound **14a:** ECD (*c* 3.2 mM, CH_3_CN) λ_max_ (Δε) 194 (−1.0), 210 (+2.0), 237 (+0.5), 280 (−0.4) nm.

Compound **14b:** ECD (*c* 3.2 mM, CH_3_CN) λ_max_ (Δε) 194 (+1.2), 210 (−1.7), 233 (−0.5), 280 (+0.5) nm.

Compound **15**: white, amorphous powder; insufficient material available for optical rotation; HPLC-UV (MeOH in H_2_O + 0.1% FA) λ_max_ 206, 231, 278–298 nm; ^1^H and ^13^C NMR data, see Table 2 and Table 3; (+)HRESIMS *m*/*z* [M + H]^+^ calcd for C_20_H_23_O_3_^+^, 311.1642, found 311.1644, ΔM −0.7 ppm; [M + Na]^+^ calcd for C_20_H_22_O_3_Na^+^, 333.1461, found 333.1460, ΔM +0.3 ppm.

Compound **15a:** ECD (*c* 3.2 mM, CH_3_CN) λ_max_ (Δε) 190 (+19.3), 215 (−10.5), 250 (−1.9), 273 (−3.9), 292 (−3.6) nm.

Compound **15b:** ECD (*c* 2.7 mM, CH_3_CN) λ_max_ (Δε) 190 (−21.6), 215 (+13.5), 250 (+3.3), 273 (+5.0), 292 (+4.7) nm.

Compound **17**: white, amorphous powder; insufficient material available for optical rotation; HPLC-UV (MeOH in H_2_O + 0.1% FA) λ_max_ 207, 221, 293–309 nm; ^1^H and ^13^C NMR data, see Table 2 and Table 3; (+)HRESIMS *m*/*z* [M + H]^+^ calcd for C_20_H_23_O_3_^+^, 311.1642, found 311.1644, ΔM −0.7 ppm; [M + Na]^+^ calcd for C_20_H_22_O_3_Na^+^, 333.1461, found 333.1460, ΔM +0.3 ppm.

Compound **17a:** ECD (*c* 5.9 mM, CH_3_CN) λ_max_ (Δε) 194 (−3.1), 211 (+11.1), 279 (−0.7), 308 (+1.1), 323 (+0.9) nm.

Compound **17b:** ECD (*c* 6.4 mM, CH_3_CN) λ_max_ (Δε) 194 (+3.5), 211 (−10.9), 280 (+1.2), 309 (−1.3), 325 (−0.7) nm.

Compound **18**: white, amorphous powder; insufficient material available for optical rotation; HPLC-UV (MeOH in H_2_O + 0.1% FA) λ_max_ 208, 218, 292–321 nm; ^1^H and ^13^C NMR data, see Table 2 and Table 3; (+)HRESIMS *m*/*z* [M + H]^+^ calcd for C_30_H_29_O_6_^+^, 485.1959, found 485.1943, ΔM +3.2 ppm; [M + Na]^+^ calcd for C_30_H_28_O_6_Na^+^, 507.1778, found 507.1760, ΔM +3.6 ppm.

Compound **18a:** ECD (*c* 1.7 mM, CH_3_CN) λ_max_ (Δε) 198 (+11.7), 212 (−54.0), 275 (−5.7), 308 (+10.3) nm.

Compound **18b:** ECD (*c* 2.0 mM, CH_3_CN) λ_max_ (Δε) 199 (−7.5), 212 (+35.9), 275 (+5.5), 308 (−9.6) nm.

Compound **19**: Colourless needle crystals (CD_3_OD); insufficient material available for optical rotation; HPLC-UV (MeOH in H_2_O + 0.1% FA) λ_max_ 206, 221, 277–291 nm; ^1^H and ^13^C NMR data, see Table 2 and Table 3; (+)HRESIMS *m*/*z* [M + H]^+^ calcd for C_30_H_29_O_6_^+^, 485.1959, found 485.1943, ΔM +3.2 ppm; [M + Na]^+^ calcd for C_30_H_28_O_6_Na^+^, 507.1778, found 507.1760, ΔM +3.6 ppm.

Compound **19a:** ECD (*c* 2.0 mM, CH_3_CN) λ_max_ (Δε) 190 (−29.5), 202 (−10.7), 216 (−19.7), 233 (+16.3), 249 (+2.1), 266 (+6.0), 290 (−8.6), 310 (+7.4) nm.

Compound **19b:** ECD (*c* 2.0 mM, CH_3_CN) λ_max_ (Δε) 190 (+28.1), 202 (+12.9), 216 (+20.0), 232 (−14.9), 249 (−2.1), 266 (−6.0), 292 (+8.4), 310 (−7.2) nm.

Compound **23**: yellowish, amorphous solid; insufficient material available for optical rotation; HPLC-UV (MeOH in H_2_O + 0.1% FA) λ_max_ 208, 219, 296-326 nm; ^1^H and ^13^C NMR data, see Table 2 and Table 3; (+)HRESIMS *m*/*z* [M + H]^+^ calcd for C_20_H_23_O_3_^+^, 311.1642, found 311.1641, ΔM +0.2 ppm; [M + Na]^+^ calcd for C_20_H_22_O_3_Na^+^, 333.1461, found 333.1446, ΔM +4.5 ppm.

Compound **23a:** ECD (*c* 3.2 mM, CH_3_CN) λ_max_ (Δε) 190 (−5.5), 213 (+6.1), 244 (+1.7), 261 (−0.3), 311 (−0.7) nm.

Compound **23b:** ECD (*c* 2.4 mM, CH_3_CN) λ_max_ (Δε) 190 (+5.7), 213 (−5.1), 244 (−1.1), 258 (+0.4), 311 (+0.7) nm.

Compound **24**: yellowish, amorphous solid; insufficient material available for optical rotation; HPLC-UV (MeOH in H_2_O + 0.1% FA) λ_max_ 201, 225, 278–292 nm; ^1^H and ^13^C NMR data, see Table 2 and Table 3; (+)HRESIMS *m*/*z* [M + H]^+^ calcd for C_30_H_29_O_6_^+^, 485.1959, found 485.1943, ΔM +3.2 ppm; [M + Na]^+^ calcd for C_30_H_28_O_6_Na^+^, 507.1778, found 507.1760, ΔM +3.6 ppm.

Compound **24a:** ECD (*c* 2.0 mM, CH_3_CN) λ_max_ (Δε) 190 (−25.0), 204 (+14.7), 216 (+24.3), 233 (−29.3), 260 (−0.6), 280 (+9.5), 306 (−31.1) nm.

Compound **24b:** ECD (*c* 3.1 mM, CH_3_CN) λ_max_ (Δε) 190 (+26.7), 204 (−13.5), 216 (−23.4), 233 (+30.1), 260 (+1.4), 280 (−8.5), 306 (+32.0) nm.

Compound **25**: yellowish, amorphous solid; insufficient material available for optical rotation; HPLC-UV (MeOH in H_2_O + 01% FA) λ_max_ 206, 222, 278–292 nm; ^1^H and ^13^C NMR data, see Table 2 and Table 3; (+)HRESIMS *m*/*z* [M + H]^+^ calcd for C_30_H_29_O_6_^+^, 485.1959, found 485.1943, ΔM +3.2 ppm; [M + Na]^+^ calcd for C_30_H_28_O_6_Na^+^, 507.1778, found 507.1760, ΔM +3.6 ppm.

Compound **25a:** ECD (*c* 2.0 mM, CH_3_CN) λ_max_ (Δε) 190 (+6.4), 203 (+17.6), 212 (−18.1), 223 (+4.7), 238 (+3.5), 274 (−6.0), 307 (+4.6) nm.

Compound **25b:** ECD (*c* 1.8 mM, CH_3_CN) λ_max_ (Δε) 190 (−6.0), 203 (−16.9), 212 (+18.3), 223 (−4.3), 238 (−3.1), 274 (+6.1), 307 (−4.4) nm.

### 3.7. ECD Data Acquisition and Calculations

ECD spectra were recorded on a J-1500 spectrometer (JASCO, Tokyo, Japan) equipped with a 1 mm path length cuvette. Calculation of ECD spectra was performed essentially as suggested for good computational practice for assignment of absolute configuration by comparison of calculated and experimental ECD spectra [46]. Thus, after establishment of the relative configuration by NMR spectroscopy, conformational searches for **6a**, **11b**, **14b**, **15b**, **17a**, **18b**, **19a**, **23b**, **24a** and **25b** were performed with MacroModel in Maestro ver. 11.8 (Schrödinger, New York, NY, USA) in the gas phase (energy window 21 kJ/mol) using Molecular Merck force field *static* (MMFFs). This yielded one conformer for **15b**, **17a**, **18b**, **19a**, **24a** and **25b**; two conformers for **11b**, **14b** and **23b**; and ten conformers for compound **6a** (Boltzmann′s population fraction weight threshold >0.5%, see supplementary information). The geometries of the selected conformers were optimized using density functional theory (DFT) with B3LYP/6-31G(d,p) level of theory in CH_3_CN [47,48] adopting to the polarizable continuum model (PCM) to describe the solvent [49,50]. Subsequently, the ECD spectra of each conformer were calculated with time-dependent density functional theory (TDDFT) at the B3LYP/6-311G(d,p) level in CH_3_CN solution as described by PCM [51]. 20 excited states of each conformer were included for **6a**, **11b**, **14b**, **15b**, **17a** and **23b**, 30 excited states of each conformer were included for **18b**, **24a** and **25b**, and 40 excited states of each conformer were included for **19a**. All DFT and TDDFT calculations were performed using the Gaussian16 package [52] (Gaussian, Inc., Wallingford, CT, USA) with Gaussian′s default setup for the PCM calculations. The final calculated ECD spectrum of each compound was generated by averaging the individual spectra of its low-energy conformer according to their Boltzmann distribution using SpecDis23 ver. 1.71 (Berlin, Germany) [53] adopting the following bandwidths and UV shifts: **6a**: 0.18 eV, −4 nm, **11b**: 0.30 eV, −1 nm, **14b**: 0.20 eV, −4 nm, **15b**: 0.30 eV, 4 nm, **17a**: 0.30 eV, −3 nm, **18b**: 0.28 eV, 2 nm, **19a**: 0.20 eV, 2 nm, **23b**: 0.20 eV, −1 nm, **24a**: 0.28 eV, 8 nm, and **25b**: 0.20 eV, 1 nm. Calculated and experimental ECD spectra were compared quantitatively by calculation of the enantiomeric similarity index (Δ_ESI_) [53] using built-in routines in SpecDisc. Geometry optimization and calculation of ECD spectra were also attempted with CAM-B3LYP in combination with e.g., the 6-311G(d,p) and/or aug-cc-pvdz basis sets, but resulted in less reliable data.

### 3.8. NMR Experiments

NMR experiments were recorded with standard pulse programs on 600 MHz Avance III spectrometers (Bruker BioSpin, Karlsruhe, Germany) equipped with 1.7-mm TCI and 5-mm DCH cryoprobes using 1.7- or 2.5-mm NMR tubes, respectively. Samples were recorded in methanol-*d*_4_ at 300 K, and ^1^H and ^13^C NMR chemical shifts were referenced to the residual solvent signal of methanol-*d*_4_ (δ 3.31 and δ 49.0, respectively). ^1^H NMR spectra were recorded using 30° pulses and 64 k data points. For the 2D NMR experiments, phase-sensitive DQF-COSY and ROESY spectra were recorded using a gradient-based pulse sequence with 12 ppm spectral width and 2k × 512 data points (processed with forward linear prediction to 1k data points in F1); a multiplicity-edited HSQC spectrum was acquired with the following parameters: ^1^*J*_C,H_ = 145 Hz, spectral width 12 ppm for ^1^H and 170 ppm for ^13^C, 1730 × 256 data points (processed with both forward linear prediction and zero filling to 1k data points in F1), and 1.0 s relaxation delay; HMBC experiment was optimized for ^n^*J*_C,H_ = 8.0 Hz (long range), ^1^*J*_C,H min_ = 125 Hz, ^1^*J*_C,H max_ = 160 Hz and acquired using a spectral width of 12 ppm for ^1^H and 240 ppm for ^13^C, 2k × 256 data points (processed with forward linear prediction to 512 data points and zero filling to 1k data points in F1), and a 1.0 s relaxation delay.

### 3.9. X-ray Crystallographic Analysis of 19

Single crystals suitable for X-ray diffraction studies were grown in deuterated methanol. A single crystal was mounted and immersed in a stream of nitrogen gas [*T* = 123(2) K]. Data were collected, using graphite-monochromated CuKα radiation (λ = 1.54178 Å) on a Bruker D8 Venture diffractometer. Data collection and cell refinement were performed using the Bruker Apex2 Suite software (Bruker AXS, Madison, WI, USA). Data reduction using SAINT ver. 8.37A (Bruker AXS) and multi-scan correction for absorption using Bruker/Siemens Area Detector Absorption Correction program SADABS-2016-2 (Bruker/Siemens Area Detector Absorption Correction program) were performed within the Apex2 Suite. The crystal data, data collection and the refinement data are given in Supplementary Material Table S16.

Positions of all non-hydrogen atoms were found by direct methods (SHELXS97) [54]. Full-matrix least-squares refinements (SHELXL-2018/3) [55] were performed on *F*^2^, minimizing Σw(*F*_o_^2^ – *kF*_c_^2^)^2^, with anisotropic displacement parameters of the non-hydrogen atoms. The positions of hydrogen atoms were located in subsequent difference electron density maps and were included in calculated position with fixed isotropic displacement parameters (*Uiso* = 1.2*Ueq* for CH, and *Uiso* = 1.5*Ueq* for CH_3_). Refinement (space group *C*2/*c*: 374 parameters, 5191 unique reflections) converged at *R_F_* = 0.0357, *wRF*^2^ = 0.0868 [4591 reflections with Fo > 4σ(*F*_o_); w^−1^ = (σ^2^(*F_o_*^2^) + (0.0381*P*)^2^ + 5.584*P*), where *P* = (*F_o_*^2^ + 2*F*_c_^2^)/3; S = 1.037]. The residual electron density varied between -0.33 and 0.35 e Å^−3^. Complex scattering factors for neutral atoms were taken from International Tables for Crystallography as incorporated in SHELXL [55,56]. Fractional atomic coordinates, a list of anisotropic displacement parameters, and a complete list of geometrical data has been deposited in the Cambridge Crystallographic Data Centre (CCDC 1982785). Copies of the data can be obtained, free of charge, on application to the director, CCDC, 12 Union Road, Cambridge CB2 1EZ, UK (fax: +44 1223 336 033 or e-mail: deposit@ccdc.cam.ac.uk).

## 4. Conclusions

To summarize, 13 new prenyl- and geranyl-substituted coumarin derivatives were isolated and identified in the ethyl acetate extract of *G. piloselloides*, guided by combined use of HPLC-PDA-HRMS and dual high-resolution PTP1B/α-glucosidase inhibition profiling for pinpointing new natural products as inhibitors of PTP1B and/or α-glucosidase. Compound **6** with a furan-oxepane 5/7 ring system represents a novel class of geranyl-substituted coumarin, and compounds **19** and **24** also represent a novel class of dimeric natural products consisting of both coumarin and chromone moieties. Interestingly, chiral resolution showed that ten of the compounds were racemic mixtures, and the absolute configuration of the separated enantiomers **6a/b**, **11a/b**, **14a/b**, **15a/b**, **17a/b**, **18a/b**, **19a/b**, **23a/b**, **24a/b** and **25a/b** were determined by comparison of their experimental and calculated ECD spectra as well as by single X-ray crystallography of one of the isolated dimeric coumarin derivatives (**19**). This study demonstrates the advantage of the combined use of dual high-resolution inhibition profiling and hyphenated LC-PDA-HRMS for pinpointing new compounds with potential as dual inhibitors, and the microplate-based inhibition profiling can be extended to include multiple pharmacological targets. Further improvement in the preparative-scale chiral separation of enantiomers is however needed to allow isolation of enantiomerically pure material for further characterization of the mode of inhibition.

## Figures and Tables

**Table 1:** ^1^H NMR (600 MHz) Spectroscopic Data of **2**, **5**, **6**, and **10**^a^.

Pos.	δ_H_ (*J*, in Hz)			Pos.	δ_H_ (*J*, in Hz)
	2	6	10		5
6	7.12, d (7.4 Hz)	7.26, d (8.4 Hz)	6.99, d (7.5 Hz)	3	5.76, d (9.8 Hz)
7	7.45, dd (8.3, 7.4 Hz)	7.56, dd (8.4, 7.5 Hz)	7.29, dd (8.3, 7.5 Hz)	4	6.42, d (9.8 Hz)
8	7.19, d (8.3 Hz)	7.20, d (7.5 Hz)	7.03, d (8.3 Hz)	5	7.56, d (2.1 Hz)
9	4.58, d (4.1 Hz)	5.51, d (7.6 Hz)	3.83, d (10.5 Hz)	7	7.70, d (2.1 Hz)
10	3.75, d (4.1 Hz)	4.78, d (7.6 Hz)	5.32, br s	10	2.52, s
11				11	1.46, s
12	2.72, s	1.98, ddd (14, 13.0, 3.3 Hz); 1.80, m	2.25, m2.02, m	12	1.47, s
13	1.52, s	2.00, m; 1.68, m	1.77, m	1′	2.95, dd (13.3, 5.5 Hz); 2.68, dd (13.4, 7.8 Hz)
14	1.57, s	3.52, d (10.0 Hz)	2.96, br s	2′	4.31, dd (7.4, 5.7 Hz)
16		4.73*^b^*; 4.81*^b^*	4.52, s; 4.54, s	4′	4.76*^b^*; 4.83 ^b^
17		1.66, s	1.68, s	5′	1.81, s
18		2.72, s	2.76, s		
19		1.58, s	1.70, s		

^a^ NMR data obtained with samples in methanol-*d_4_*. ^b^ Signals overlapping with residual solvent signal of methanol at δ 3.31, water at δ 4.87 or other signals from the molecule.

**Table 2:** ^13^C NMR (150 MHz) Spectroscopic Data of **2**, **5**, **6**, **10**, **11**, **14**, **15**, **17**, and **23**^a^.

Pos.	δ_C_								Pos.	δ_C_
	2	6	10	11	14	15	17	23		5
2	164.7	161.4	165.5	162.6	164.1	162.7	163.9	165.2	2	78.9
3	101.8	102.7	106.4	100.5	102.9	99.7	102.9	103.9	3	132.0
4	163.7	171.1	171.4	182.2	162.4	181.8	163.5	163.6	4	122.8
4a	115.5	112.2	118.6	122.3	120	121	116.1	116.1	4a	121.7
5	139.0	138.5	138.8	133.0	138.0	141.3	138.2	138.1	5	126.4
6	128.9	115.5	127.6	133.1	128.2	128.9	128.7	128.9	6	130.7
7	132.8	133.8	130.7	128.9	131.6	133.0	132.0	132.1	7	133.1
8	115.9	127.5	115.1	116.2	115.4	116.3	115.7	115.9	8	127.7
8a	155.5	157.9	155.2	155.7	154.9	155.8	155.0	155.0	8a	156.8
9	66.5	76.1	37.3	31.1	31.7	33.7	34.1	37.4	9	199.6
10	73.8	95.9	126.0	121.7	121.0	123.1	122.8	38.4	10	26.1
11	83.1	74.0	135.5	135.4	135.2	134.7	135.2	88.2	11	28.6
12	23.3	36.1	31.4	30.5	30.3	32.2	32.0	40.6	12	28.6
13	24.4	29.9	30.3	21.4	21.2	25.4	25.5	26.6	1′	36.8
14	22.9	78.3	45.4	41.0	40.3	47.1	46.2	48	2′	75.8
15		147.5	150	84.8	83.6	86.4	86.3	40.4	3′	148.6
16		111.0	110.6	22.4	23.3	22.6	23.9	23.4	4′	111.1
17		19.5	19.2	23.4	23.4	23.3	23.4	27.6	5′	17.7
18		21.1	23.2	25.5	25.6	27.1	27.3	34.1		
19		30.8	23.4	25.4	25.0	20.1	20.3	17.9		

^a^ NMR data obtained with samples in methanol-*d*_4_.

**Table 3:** ^1^H NMR (600 MHz) Spectroscopic Data of **11**, **14**, **15**, **17**, and **23**^a^.

Pos.	11	14	15	17	23
6	7.13, d (7.3 Hz)	7.08, d (7.4 Hz)	7.14, d (7.5 Hz)	7.08, d (7.5 Hz)	7.11, d (7.5 Hz)
7	7.47, dd (8.3, 7.3 Hz)	7.39, dd (8.2, 7.4 Hz)	7.48, dd (8.3, 7.5 Hz)	7.39, dd (8.3, 7.5 Hz)	7.41, dd (8.4, 7.5 Hz)
8	7.22, d (8.3 Hz)	7.13, d (8.2 Hz)	7.23, d (8.3 Hz)	7.13, d (8.3 Hz)	7.16, d (8.3 Hz)
9	3.51, dd (6.7, 4.0 Hz)	3.42, dd (6.8, 5.4 Hz)	3.21, d (10.8 Hz)	3.11, d (10.9 Hz)	3.01, d (9.4 Hz)
10	6.25, br s	6.18, br s	6.24, m	6.24, m	2.72 (m)
12	2.02, m; 1.98, m	2.06, m; 1.98, m	2.19, br s	2.19, m	1.91, dd (13.0, 7.5 Hz); 2.07, dd (12.8, 7.5 Hz)
13	2.04, m; 1.38, m	2.02, m; 1.34, m	1.96, m; 1.49, m	1.98, m; 1.47, m	1.76, dd (14.0, 7.5 Hz); 1.73, m
14	1.88, ddd (11.5, 6.1, 3.0 Hz)	1.88, ddd (11.8, 6.3, 3.0 Hz)	1.7, ddd (13.0, 10.8, 2.1 Hz)	1.71, ddd (12.7, 11.0, 2.0 Hz)	2.50, dd (8.1, 7.0 Hz)
16	2.80, s	2.69, s	2.81, s	2.70, s	2.74, s
17	1.67, s	1.68, s	1.68, s	1.68, s	1.56, s
18	1.51, s	1.57, s	1.54, s	1.61, s	1.40, s
19	1.42, s	1.41, s	1.26, s	1.26, s	0.88, s

^a^ NMR data obtained with samples in methanol-*d_4_*.

**Table 4:** ^13^C NMR (150 MHz) Spectroscopic Data of **18**, **19**, **24**, and **25**^a.^

Pos.	δ_C_				Pos.	δ_C_			
	18	19	24	25		18	19	24	25
2	163.5	163.3	163.9	163.0	2′	165.0	163.8	163.5	163.4
3	103.0	99.6	99.2	102.6	3′	101.3	104.9	109.0	109.0
4	167.5	181.7	181.9	164.2	4′	164.0	166.3	163.1	163.0
4a	115.1	121.5	122.2	115.8	4a′	115.7	115.4	116.0	115.6
5	138.3	141.2	141.3	138.3	5′	138.0	138.3	138.3	138.3
6	128.7	128.7	128.6	128-7	6′	128.5	128.5	128.4	128.8
7	132.4	133.0	131.9	132.1	7′	131.9	132.0	132.7	132.2
8	115.6	116.4	115.4	115.6	8′	115.3	115.9	115.9	115.8
8a	154.9	155.8	155.7	155.1	8a′	154.7	155.1	155.1	155.2
9	28.8	26.2	25.9	26.4	9′	28.9	28.8	26.5	26.7
10	52.9	45.0	54.1	53.1	10′	38.2	31.9	30.2	30.5
11	81.4	86.3	86.4	85.0	11′	83.3	80.8	81.0	81.5
12	23.5	22.4	22.3	23.9	12′	23.6	23.6	23.4	23.7
13	26.8	24.9	28.3	28.9	13′	43.2	40.3	40.1	40.8
14	19.7	28.9	20.5	21.2	14′	27.8	28.3	28.9	29.3

^a^ NMR data obtained with samples in methanol-*d*_4_.

**Table 5:** ^1^H NMR (600 MHz) Spectroscopic Data of **18**, **19**, **24**, and **25**^a^.

Pos.	δ_H_ (*J*, in Hz)			
	18	19	24	25
6	7.16, d (7.4 Hz)	7.13, d (7.3 Hz)	7.13, d (7.5 Hz)	7.07, d (7.5 Hz)
7	7.46, dd (8.2, 7.4 Hz)	7.48, dd (8.2, 7.3 Hz)	7.40, dd (8.2, 7.5 Hz)	7.38, dd (8.0, 7.5 Hz)
8	7.19, d (8.2 Hz)	7.24, d (8.0 Hz)	7.15, d (8.2 Hz)	7.11, d (8.0 Hz)
9	2.68, td (12.0, 4.2 Hz)	3.10, br dt (13.1, 6.3 Hz)	2.72, td (12.7, 4.2 Hz)	2.68, td (12.6, 4.2 Hz)
10	2.17, dd (12.0, 1.9 Hz)	2.15, br d (6.6 Hz)	1.62, dd (12.4, 3.2 Hz)	1.62, dd (12.4, 3.1 Hz)
12	2.70, s	2.76, s	2.78, s	2.69, s
13	1.91, s	1.67, s	1.79, s	1.87, s
14	1.10, s	1.77, s	1.43, s	1.44, s
6′	7.09, d (7.4 Hz)	7.15, d (7.5 Hz)	7.09, d (7.6 Hz)	7.10, d (7.8 Hz)
7′	7.39, dd (8.4, 7.4 Hz)	7.44, dd (8.3, 7.5 Hz)	7.46, dd (8.4, 7.6 Hz)	7.41, dd (8.3, 7.8 Hz)
8′	7.12, d (8.4 Hz)	7.17, d (8.3 Hz)	7.21, d (8.4 Hz)	7.15, d (8.3 Hz)
9′	3.59, q (2.7 Hz)	3.50, br q	3.08, q (3.2 Hz)	3.13, q (3.1 Hz)
10′	2.22, dd (13.2, 2.8 Hz) 1.90, dt (13.2, 2.8 Hz)	2.31, dd (14.1, 2.8 Hz); 1.82, dt (14.1, 2.5 Hz)	2.29, dd (14.0, 3.4 Hz); 1.53, dd (14.0, 2.6 Hz)	2.28, dd (13.9, 3.4 Hz); 1.55, dd (13.9, 2.7 Hz)
12′	2.81, s	2.84, s	2.73, s	2.74, s
13′	3.51, ddd (14.0, 4.2, 2.6 Hz); 1.47, dd (14.0, 12.0 Hz)	3.02, ddd (14.2, 5.5, 2.5 Hz); 1.71, dd (14.2, 13.1 Hz)	3.80, dd (14.7, 4.2 Hz); 1.40 dd (14.7, 13.1 Hz)	3.68, dd (14.8, 4.3 Hz); 1.48, dd (14.8, 13.0 Hz)
14′	1.61, s	1.61, s	1.78, s	1.76, s

^a^ NMR data obtained with samples in methanol-*d*_4_.

**Table 6:** Percentage inhibition of **17**–**19**, **24** and **25** against α-glucosidase and PTP1B at the concentration shown in brackets.

Compound	α-Glucosidase (μM)	PTP1B (μM)
**17**	32% (201.6)	N.D.^a^
**18**	40% (159.1)	N.D.^a^
**19**	48% (129.1)	67% (64.4)
**24**	51% (129.1)	47% (32.2)
**25**	45% (64.4)	32% (129.1)

^a^ N.D. Not detected.

## Supplementary Materials

The following are available online, HRMS and 1D data of known compounds, chromatograms from chiral separations, ECD spectra, details of ECD calculation, X-ray crystallographic analysis of **19**, and 1D and 2D NMR spectra of new compounds identified in this study.
