# Supplementary material for: Structure Elucidation of Prenyl- and Geranyl-Substituted Coumarins in Gerbera piloselloides by NMR Spectroscopy, Electronic Circular Dichroism Calculations, and Single Crystal X-ray Crystallography
Source: Molecules. 2020 Apr 8;25(7):1706. doi: 10.3390/molecules25071706 (PMC7180714; doi:10.3390/molecules25071706)
Supplement: Supplementary file 1 [file molecules-25-01706-s001.pdf]

Supplementary Material for:

Structure Elucidation of Prenyl- and Geranyl Substituted Coumarin Derivatives in *Gerbera piloselloides* by NMR Spectroscopy, Electronic Circular Dichroism Calculations, and Single Crystal X-ray Crystallography

Tuo Li,[1] Xue Ma,[2] Daniil Fedotov,[3] Louise Kjaerulff,[1] Karla Frydenvang,[1] Sonia Coriani,[2] Paul Robert Hansen,[1] Kenneth T. Kongstad,[1] and Dan Staerk[1,*]

[1] Department of Drug Design and Pharmacology, Faculty of Health and Medical Sciences, University of Copenhagen, Universitetsparken 2, DK-2100 Copenhagen, Denmark

[2] Engineering Research Center for the Development and Application of Ethnic Medicine and TCM, Guizhou Medical University, No.4 Beijing Road, Yunyan District, Guiyang 550004, People's Republic of China

[3] Department of Chemistry, Technical University of Denmark, Kemitorvet, DK-2800, Kgs. Lyngby, Denmark

[*]Correspondence: ds@sund.ku.dk; Tel.: +45 35336177

Table of Contents

Table S1. Retention time, name, structure, (+)HRESIMS, and ^{1}H NMR data of compounds 1, 3, 4 and 7-9, 12 and 16. 7

Figure S1. Chiral separation of 6, 11, 14 and 15. 9

Figure S2. Chiral separation of 17, 18, 19 and 23. 10

Figure S3. Chiral separation of 24 and 25. 11

Scheme 1. Possible biosynthesis of 11, 14, 15 and 17 via a [4+2] cycloaddition. 12

Figure S4. Optimized geometries of the predominant conformers of 6a at the B3LYP/6-31G(d,p) level in CH_3CN (PCM). 13

Table S2. Conformational analysis of 6a at 298K 14

Table S3. Cartesian coordinates of optimized 6a conformers. B3LYP/6-31G(d,p) in CH_3CN (PCM) 15

Figure S5. Optimized geometries of the predominant conformers of 11b at the B3LYP/6-31G(d,p) level in CH_3CN (PCM). 20

Table S4. Conformational analysis of 11b at 298K 20

Table S5. Cartesian coordinates of optimized 11b conformers. B3LYP/6-31G(d,p) in CH_3CN (PCM) 21

Figure S6. Optimized geometries of the predominant conformers of 14b at the B3LYP/6-31G(d,p) level in CH_3CN (PCM). 22

Table S6. Conformational analysis of 14b at 298K 22

Table S7. Cartesian coordinates of the optimized 14b conformers. B3LYP/6-31G(d,p) in CH_3CN (PCM) 23

Table S8. Cartesian coordinates of the optimized 15b. B3LYP/6-31G(d,p) in CH_3CN (PCM) 24

Table S9. Cartesian coordinates of the optimized 17b. B3LYP/6-31G(d,p) in CH_3CN (PCM) 25

Table S10. Cartesian coordinates of the optimized 18b. B3LYP/6-31G(d,p) in CH_3CN (PCM) 26

Table S11. Cartesian coordinates of the optimized 19a. B3LYP/6-31G(d,p) in CH_3CN (PCM) 28

Figure S7. Optimized geometries of the predominant conformers of 23b at the B3LYP/6-31G(d,p) level in CH_3CN solvent as described by PCM. 30

Table S12. Conformational analysis of 23b at 298K 30

Table S13. Cartesian coordinates of the optimized 23b conformers. B3LYP/6-31G(d,p) in CH_3CN (PCM). 31

Table S14. Cartesian coordinates of the optimized 24a. B3LYP/6-31G(d,p) in CH_3CN (PCM). 32

Table S15. Cartesian coordinates of the optimized 25b. B3LYP/6-31G(d,p) in CH_3CN (PCM). 34

Figure S8. ECD spectra of 2 and 8 36

Figure S9. ECD spectra of 6a and 6b 37

Figure S10. ECD spectra of 11a and 11b 38

Figure S11. ECD spectra of 14a and 14b 39

Figure S12. ECD spectra of 15a and 15b 40

Figure S13. ECD spectra of 17a and 17b 41
Figure S14. ECD spectra of 18a and 18b 42
Figure S15. ECD spectra of 19a and 19b 43
Figure S16. ECD spectra of 23a and 23b 44
Figure S17. ECD spectra of 24a and 24b 45
Figure S18. ECD spectra of 25a and 25b 46
Figure S19. UV spectra obtained from HPLC-PDA-HRMS analysis of new compounds identified in *G. piloselloides* 47
Figure S20. ^{1}H NMR spectrum of 1 (600 MHz, methanol-d_4). 49
Figure S21. ^{1}H NMR spectrum of 2 (600 MHz, methanol-d_4) 50
Figure S22. ^{13}C NMR spectrum of 2 (151 MHz, methanol-d_4) 51
Figure S23. HSQC spectrum of 2 (600 MHz, methanol-d_4) 52
Figure S24. COSY spectrum of 2 (600 MHz, methanol-d_4)) 53
Figure S25. HMBC spectrum of 2 (600 MHz, methanol-d_4) 54
Figure S26. ROESY spectrum of 2 (600 MHz, methanol-d_4) 55
Figure S27. ^{1}H NMR spectrum of 3 (600 MHz, methanol-d_4). 56
Figure S28. COSY spectrum of 3 (600 MHz, methanol-d_4) 57
Figure S29. HSQC spectrum of 3 (600 MHz, methanol-d_4) 58
Figure S30. ^{1}H NMR spectrum of 4 (600 MHz, methanol-d_4). 59
Figure S31. COSY spectrum of 4 (600 MHz, methanol-d_4) 60
Figure S32. HSQC spectrum of 4 (600 MHz, methanol-d_4) 61
Figure S33. ^{1}H NMR spectrum of 5 (600 MHz, methanol-d_4) 62
Figure S34. ^{13}C NMR spectrum of 5 (151 MHz, methanol-d_4) 63
Figure S35. HSQC spectrum of 5 (600 MHz, methanol-d_4) 64
Figure S36. COSY spectrum of 5 (600 MHz, methanol-d_4) 65
Figure S37. HMBC spectrum of 5 (600 MHz, methanol-d_4) 66
Figure S38. ROESY spectrum of 5 (600 MHz, methanol-d_4) 67
Figure S39. ^{1}H NMR spectrum of 6 (600 MHz, methanol-d_4) 68
Figure S40. ^{13}C NMR spectrum of 6 (151 MHz, methanol-d_4) 69
Figure S41. HSQC spectrum of 6 (600 MHz, methanol-d_4) 70
Figure S42. COSY spectrum of 6 (600 MHz, methanol-d_4) 71
Figure S43. HMBC spectrum of 6 (600 MHz, methanol-d_4) 72
Figure S44. ROESY spectrum of 6 (600 MHz, methanol-d_4) 73

Figure S45. ^{1}H NMR spectrum of 7 (600 MHz, chloroform-*d*) 74
Figure S46. COSY spectrum of 7 (600 MHz, chloroform-*d*) 75
Figure S47. HQSC spectrum of 7 (600 MHz, chloroform-*d*) 76
Figure S48. ^{1}H NMR spectrum of 8 (600 MHz, methanol-d_4) 77
Figure S49. HSQC spectrum of 8 (600 MHz, methanol-d_4) 78
Figure S50. COSY spectrum of 8 (600 MHz, methanol-d_4). 79
Figure S51. HMBC spectrum of 8 (600 MHz, methanol-d_4). 80
Figure S52. ROESY spectrum of 8 (600 MHz, methanol-d_4) 81
Figure S53. ^{1}H NMR spectrum of 9 (600 MHz, methanol-d_4) 82
Figure S54. HSQC spectrum of 9 (600 MHz, methanol-d_4) 83
Figure S55. HMBC spectrum of 9 (600 MHz, methanol-d_4) 84
Figure S56. ^{1}H NMR spectrum of 10 (600 MHz, methanol-d_4) 85
Figure S57. HSQC spectrum of 10 (600 MHz, methanol-d_4) 86
Figure S58. COSY spectrum of 10 (600 MHz, methanol-d_4) 87
Figure S59. HMBC spectrum of 10 (600 MHz, methanol-d_4) 88
Figure S60. ROESY spectrum of 10 (600 MHz, methanol-d_4) 89
Figure S61. ^{1}H NMR spectrum of 11 (600 MHz, methanol-d_4) 90
Figure S62. ^{13}C NMR spectrum of 11 (151 MHz, methanol-d_4) 91
Figure S63. HSQC spectrum of 11 (600 MHz, methanol-d_4) 92
Figure S64. COSY spectrum of 11 (600 MHz, methanol-d_4) 93
Figure S65. HMBC spectrum of 11 (600 MHz, methanol-d_4). 94
Figure S66. ROESY spectrum of 11 (600 MHz, methanol-d_4) 95
Figure S67. ^{1}H NMR spectrum of 12 (600 MHz, methanol-d_4) 96
Figure S68. HSQC spectrum of 12 (600 MHz, methanol-d_4) 97
Figure S69. COSY spectrum of 12 (600 MHz, methanol-d_4). 98
Figure S70. HMBC spectrum of 12 (600 MHz, methanol-d_4). 99
Figure S71. ROESY spectrum of 12 (600 MHz, methanol-d_4). 100
Figure S72. ^{1}H NMR spectrum of 14 (600 MHz, methanol-d_4) 101
Figure S73. ^{13}C NMR spectrum of 14 (151 MHz, methanol-d_4) 102
Figure S74. HSQC spectrum of 14 (600 MHz, methanol-d_4) 103
Figure S75. COSY spectrum of 14 (600 MHz, methanol-d_4) 104
Figure S76. HMBC spectrum of 14 (600 MHz, methanol-d_4) 105
Figure S77. ROESY spectrum of 14 (600 MHz, methanol-d_4) 106

Figure S78. ^{1}H NMR spectrum of 15 (600 MHz, methanol-d_4) 107
Figure S79. ^{13}C NMR spectrum of 15 (151 MHz, methanol-d_4) 108
Figure S80. HSQC spectrum of 15 (600 MHz, methanol-d_4) 109
Figure S81. COSY spectrum of 15 (600 MHz, methanol-d_4) 110
Figure S82. HMBC spectrum of 15 (600 MHz, methanol-d_4) 111
Figure S83. ROESY spectrum of 15 (600 MHz, methanol-d_4) 112
Figure S84. ^{1}H NMR spectrum of 16 (600 MHz, methanol-d_4). 113
Figure S85. ^{13}C NMR spectrum of 16 (150 MHz, methanol-d_4) 114
Figure S86. COSY spectrum of 16 (600 MHz, methanol-d_4) 115
Figure S87. HSQC spectrum of 16 (600 MHz, methanol-d_4) 116
Figure S88. ^{1}H NMR spectrum of 17 (600 MHz, methanol-d_4) 117
Figure S89. ^{13}C NMR spectrum of 17 (151 MHz, methanol-d_4) 118
Figure S90. HSQC spectrum of 17 (600 MHz, methanol-d_4) 119
Figure S91. COSY spectrum of 17 (600 MHz, methanol-d_4) 120
Figure S92. HMBC spectrum of 17 (600 MHz, methanol-d_4) 121
Figure S93. ROESY spectrum of 17 (600 MHz, methanol-d_4) 122
Figure S94. ^{1}H NMR spectrum of 18 (600 MHz, methanol-d_4) 123
Figure S95. J-MOD NMR spectrum of 18 (600 MHz, methanol-d_4) 124
Figure S96. HSQC spectrum of 18 (600 MHz, methanol-d_4) 125
Figure S97. COSY spectrum of 18 (600 MHz, methanol-d_4) 126
Figure S98. HMBC spectrum of 18 (600 MHz, methanol-d_4) 127
Figure S99. ROESY spectrum of 18 (600 MHz, methanol-d_4) 128
Figure S100. ^{1}H NMR spectrum of 19 (600 MHz, methanol-d_4) 129
Figure S101. J-MOD NMR spectrum of 19 (151 MHz, methanol-d_4) 130
Figure S102. HSQC spectrum of 19 (600 MHz, methanol-d_4) 131
Figure S103. COSY spectrum of 19 (600 MHz, methanol-d_4) 132
Figure S104. HMBC spectrum of 19 (600 MHz, methanol-d_4) 133
Figure S105. ROESY spectrum of 19 (600 MHz, methanol-d_4) 134
Figure S106. ^{1}H NMR spectrum of 23 (600 MHz, methanol-d_4) 135
Figure S107. ^{13}C NMR spectrum of 23 (151 MHz, methanol-d_4) 136
Figure S108. HSQC spectrum of 23 (600 MHz, methanol-d_4) 137
Figure S109. COSY spectrum of 23 (600 MHz, methanol-d_4) 138
Figure S110. HMBC spectrum of 23 (600 MHz, methanol-d_4) 139

Figure S111. ROESY spectrum of 23 (600 MHz, methanol-d_4) 140
Figure S112. ^{1}H NMR spectrum of 24 (600 MHz, methanol-d_4) 141
Figure S113. ^{13}C NMR spectrum of 24 (151 MHz, methanol-d_4) 142
Figure S114. HSQC spectrum of 24 (600 MHz, methanol-d_4) 143
Figure S115. COSY spectrum of 24 (600 MHz, methanol-d_4) 144
Figure S116. HMBC spectrum of 24 (600 MHz, methanol-d_4) 145
Figure S117. ROESY spectrum of 24 (600 MHz, methanol-d_4) 146
Figure S118. ^{1}H NMR spectrum of 25 (600 MHz, methanol-d_4) 147
Figure S119. ^{13}C NMR spectrum of 25 (151 MHz, methanol-d_4) 148
Figure S120. HSQC spectrum of 25 (600 MHz, methanol-d_4) 149
Figure S121. COSY spectrum of 25 (600 MHz, methanol-d_4) 150
Figure S122. HMBC spectrum of 25 (600 MHz, methanol-d_4) 151
Figure S123. ROESY spectrum of 25 (600 MHz, methanol-d_4) 152
Table S16. Crystal data, data collection and structure refinement data of 19 153

Table S1. Retention time, name, structure, (+)HRESIMS, and ^{1}H NMR data of compounds **1**, **3**, **4** and **7-9**, **12** and **16**.

No.	RT (min)	Name	Structure	*m/z* (MF, ppm)	^{1}H NMR (nH, m, *J* (in Hz))[a,b]
**1**	18.6	Marmesin	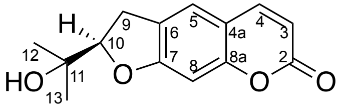	255.1016 [M + H]$^+$ ($C_{16}H_{15}O_3^+$, ΔM -0.1)	7.84 (1H, d, 9.4 Hz, H-4); 7.39 (1H, s, H-5); 6.71 (1H, s, H-8); 6.18 (1H, d, 9.4 Hz, H-3); 4.75 (1H, t, 9.3 Hz, H-10); 3.23-3.25 (2H, m, H-9); 1.28 (3H, s, H-12); 1.22 (3H, s, H-13)
**3**	28.4	7-Demethyl-suberosin	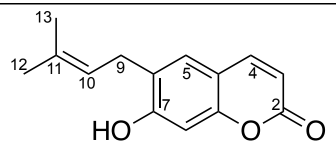	231.1021 [M + H]$^+$ ($C_{14}H_{15}O_3^+$, ΔM -2.3)	7.82 (1H, d, 9.4 Hz, H-4); 7.28 (1H, s, H-5); 6.70 (1H, s, H-8); 6.16 (1H, d, 9.4 Hz, H-3); 5.33 (1H, t, 7.4 Hz, H-10); 3.32 (2H, overlapping, H-9); 1.75 (3H, s, H-12); 1.71 (3H, s, H-13)
**4**	29.6	Apigravin	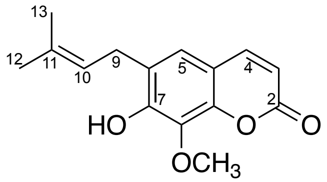	261.1118 [M + H]$^+$ ($C_{15}H_{17}O_4^+$, ΔM 1.3) 283.094 [M + Na]$^+$ ($C_{15}H_{16}O_4Na^+$, ΔM 0.3)	7.84 (1H, d, 9.5 Hz, H-4); 6.90 (1H, s, H-5); 6.28 (1H, d, 9.5 Hz, H-3); 5.27 (1H, t, 7.2 Hz, H-10); 3.89 (3H, s, 8-*O*CH$_3$); 3.37 (2H, m, H-9); 1.74 (3H, s, H-12); 1.74 (3H, s, H-13)
**7**	34.1	Bothrioclinin	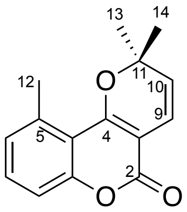	243.1018 [M + H]$^+$ ($C_{15}H_{15}O_3^+$, ΔM -0.9) 265.0826 [M + Na]$^+$ ($C_{15}H_{14}O_3Na^+$, ΔM 3.4)	7.36 (1H, dd, 8.3, 7.6 Hz, H-7); 7.17 (1H, d, 8.3 Hz, H-6); 7.03 (1H, d, 7.6 Hz, H-8); 6.56 (1H, d, 10.0 Hz, H-9); 5.49 (1H, d, 10.0 Hz, H-10); 2.74 (3H, s, H-12); 1.60 (6H, s, H-13, H-14)
**8**	34.7	(+) 2-[(2*R*)-6-acetyl-2,3-Dihydro-5-hydroxy-benzofuran-2-yl]prop-2-enyl 15-methylbutanoate	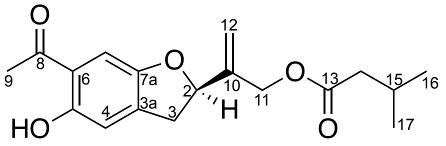	319.1535 [M + H]$^+$ ($C_{18}H_{23}O_5^+$, ΔM 1.6) 341.1355 [M + Na]$^+$ ($C_{18}H_{22}O_5Na^+$, ΔM 1.3)	7.16 (1H, s, H-7); 6.79 (1H, s, H-4); 5.33 (1H, br s, H-12); 5.29 (1H, t, 8.5 Hz, H-2), 5.24 (1H, br s, H-12); 4.72 (1H, d, 13.3 Hz, H-11), 4.63 (1H, d, 13.3 Hz, H-11), 3.45 (1H, dd, 16.9, 8.5 Hz, H-3); 3.17 (1H, dd, 16.9, 8.5, H-3); 2.56 (3H, s, H-9); 2.14 (2H, m, H-14), 2.00 (1H, m, H-15), 0.91 (3H, d, 5.2 Hz, H-17), 0.90 (3H, d, 5.2 Hz, H-16)

**9**	35.7	3,5-Bis-(isopent-2-en-1-yl)-4-hydroxyacetophenone	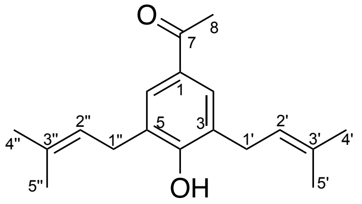	273.1849 [M + H]$^+$ ($C_{18}H_{25}O_2^+$, ΔM 0) 295.1652 [M + Na]$^+$ ($C_{18}H_{24}O_2Na^+$, ΔM 5.6)	7.60 (2H, s, H-2, H-6); 5.33 (2H, t, 7.3 Hz, H-2', H-2"); 3.35 (2H, br s, H-1', H-1"); 3.34 (2H, br s, H-1'. H-1"); 2.49 (3H, s, H-8); 1.76 (6H, s, H-4', H-4"); 1.73 (6H, s, H-5', H-5")
**12**	38.5	6-Acetyl-2,2-dimethyl-8-(3'-methyl-2'-butenyl)-2*H*-chromen	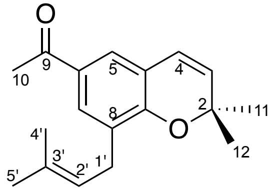	271.1701 [M + H]$^+$ ($C_{18}H_{23}O_2^+$, ΔM -3.1) 293.1515 [M + Na]$^+$ ($C_{18}H_{22}O_2Na^+$, ΔM -1.0)	7.64 (1H, d, 2.1 Hz, H-7); 7.53 (1H, d, 2.1 Hz, H-5); 6.42 (1H, d, 9.9 Hz, H-4); 5.75 (1H, d, 9.9 Hz, H-3); 5.25 (1H, tsep, 7.4, 1.4 Hz, H-2'); 3.28 (2H, d, 7.4 Hz, H-1'); 2.51 (3H, s, H-10); 1.74 (3H, s, H-4'); 1.73 (3H, s, H-5'); 1.44 (6H, s, H-11, H-12)
**16**	43.2	Mutisicoumarin B	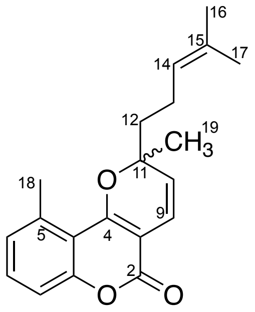	311.1629 [M + H]$^+$ ($C_{20}H_{23}O_3^+$, ΔM 4.1) 333.1469 [M + Na]$^+$ ($C_{20}H_{22}O_3Na^+$, ΔM -2.4)	7.44 (1H, dd, 8.3, 7.5 Hz, H-7); 7.17 (1H, dd, 8.3, 1.3 Hz, H-8); 7.12 (1H, dd, 7.5, 1.3 Hz, H-6); 6.50 (1H, d, 10.1 Hz, H-9); 5.56 (1H, d, 10.1 Hz, H-10); 5.13 (1H, tq, 7.2, 1.4 Hz, H-14); 2.20 (2H, m, H-13); 1.88 (2H, m, H-12); 2.75 (3H, s, H-18); 1.57 (3H, s, H-19); 1.54 (3H, d, 1.43 Hz, H-16); 1.52 (3H, br s, H-17)

[a] NMR data obtained at 600 MHz with samples in methanol-d_4. [b] Multiplicities reported as apparent splittings: s = singlet, d = doublet, t = triplet, q = quartet, sep = septet, m = multiplet, br = broad.

Figure S1. Chiral separation of **6, 11, 14** and **15**.

8 sample_analytisk

Sample Name:	**sample_analytisk**	*Injection Volume:*	**20,0**
Vial Number:	**6**	*Channel:*	**UV_VIS_4**
Sample Type:	**unknown**	*Wavelength:*	**280.0**
Control Program:	**35min-1,0mL_min_20A_80B**	*Bandwidth:*	**4**
Quantif. Method:	**Default1_do not change**	*Dilution Factor:*	**1,0000**
Recording Time:	**29-10-2018 12:34**	*Sample Weight:*	**1,0000**
Run Time (min):	**35,00**	*Sample Amount:*	**1,0000**

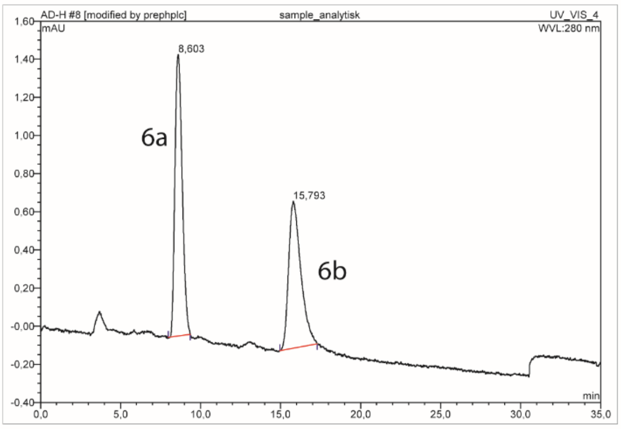

No.	Ret.Time min	Peak Name	Height mAU	Area mAU*min	Rel.Area %	Amount	Type
1	8,60	n.a.	1,476	0,776	53,35	n.a.	BMB*
2	15,79	n.a.	0,770	0,679	46,65	n.a.	BMB*
**Total:**			2,246	1,455	100,00	0,000	

91 sample

Sample Name:	**sample**	*Injection Volume:*	**20,0**
Vial Number:	**86**	*Channel:*	**UV_VIS_1**
Sample Type:	**unknown**	*Wavelength:*	**210.0**
Control Program:	**45min-1,0mL_min_1A_99B**	*Bandwidth:*	**4**
Quantif. Method:	**Default1_do not change**	*Dilution Factor:*	**1,0000**
Recording Time:	**28-12-2018 20:36**	*Sample Weight:*	**1,0000**
Run Time (min):	**30,36**	*Sample Amount:*	**1,0000**

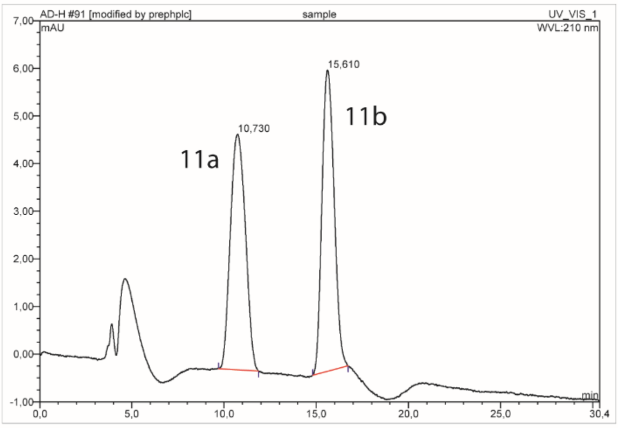

No.	Ret.Time min	Peak Name	Height mAU	Area mAU*min	Rel.Area %	Amount	Type
1	10,73	n.a.	4,947	4,676	49,16	n.a.	BMB
2	15,61	n.a.	6,322	4,835	50,84	n.a.	BMB
**Total:**			11,270	9,511	100,00	0,000	

86 sample

Sample Name:	**sample**	*Injection Volume:*	**20,0**
Vial Number:	**81**	*Channel:*	**UV_VIS_4**
Sample Type:	**unknown**	*Wavelength:*	**280.0**
Control Program:	**45min-0,8mL_min_2A_98B**	*Bandwidth:*	**4**
Quantif. Method:	**Default1_do not change**	*Dilution Factor:*	**1,0000**
Recording Time:	**20-12-2018 13:32**	*Sample Weight:*	**1,0000**
Run Time (min):	**27,78**	*Sample Amount:*	**1,0000**

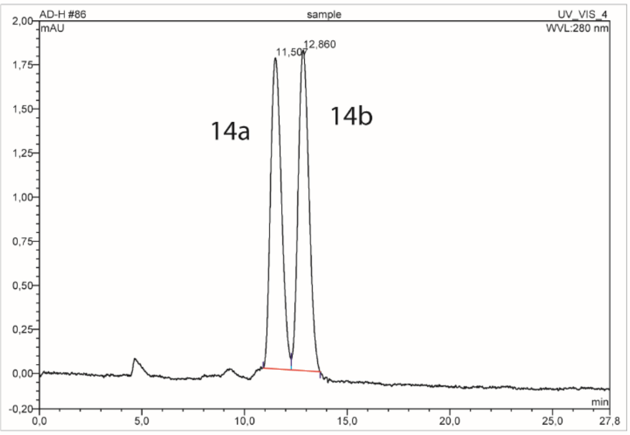

No.	Ret.Time min	Peak Name	Height mAU	Area mAU*min	Rel.Area %	Amount	Type
1	11,51	n.a.	1,764	1,080	49,37	n.a.	BM
2	12,86	n.a.	1,817	1,108	50,63	n.a.	MB
**Total:**			3,581	2,188	100,00	0,000	

79 sample

Sample Name:	**sample**	*Injection Volume:*	**20,0**
Vial Number:	**74**	*Channel:*	**UV_VIS_1**
Sample Type:	**unknown**	*Wavelength:*	**210.0**
Control Program:	**45min-1,0mL_min_1A_99B**	*Bandwidth:*	**4**
Quantif. Method:	**Default1_do not change**	*Dilution Factor:*	**1,0000**
Recording Time:	**19-12-2018 17:07**	*Sample Weight:*	**1,0000**
Run Time (min):	**23,95**	*Sample Amount:*	**1,0000**

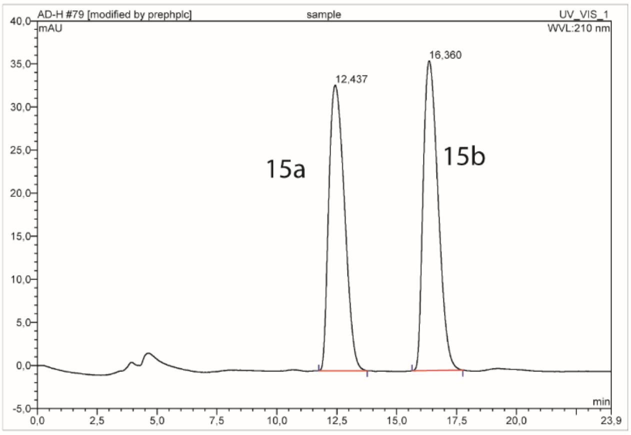

No.	Ret.Time min	Peak Name	Height mAU	Area mAU*min	Rel.Area %	Amount	Type
1	12,44	n.a.	33,178	25,175	49,22	n.a.	BMB
2	16,36	n.a.	35,995	25,971	50,78	n.a.	BMB
**Total:**			69,172	51,146	100,00	0,000	

Figure S2. Chiral separation of **17**, **18**, **19** and **23.**

73 sample

Sample Name:	**sample**	*Injection Volume:*	**20,0**
Vial Number:	**68**	*Channel:*	**UV_VIS_4**
Sample Type:	**unknown**	*Wavelength:*	**280.0**
Control Program:	**45min-1,0mL_min_1A_99B**	*Bandwidth:*	**4**
Quantif. Method:	**Default1_do not change**	*Dilution Factor:*	**1,0000**
Recording Time:	**19-12-2018 12:33**	*Sample Weight:*	**1,0000**
Run Time (min):	**28,50**	*Sample Amount:*	**1,0000**

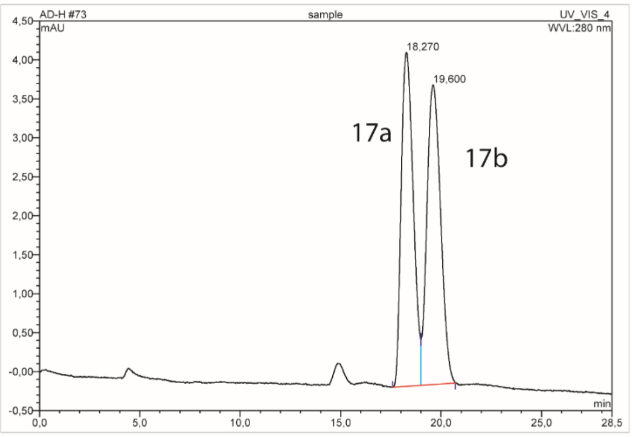

No.	Ret.Time min	Peak Name	Height mAU	Area mAU*min	Rel.Area %	Amount	Type
1	18,27	n.a.	4,288	2,978	49,39	n.a.	BM
2	19,60	n.a.	3,849	3,051	50,61	n.a.	MB
**Total:**			8,137	6,030	100,00	0,000	

62 sample

Sample Name:	**sample**	*Injection Volume:*	**20,0**
Vial Number:	**57**	*Channel:*	**UV_VIS_1**
Sample Type:	**unknown**	*Wavelength:*	**210.0**
Control Program:	**35min-1,0mL_min_10A_90B**	*Bandwidth:*	**4**
Quantif. Method:	**Default1_do not change**	*Dilution Factor:*	**1,0000**
Recording Time:	**16-12-2018 17:52**	*Sample Weight:*	**1,0000**
Run Time (min):	**27,77**	*Sample Amount:*	**1,0000**

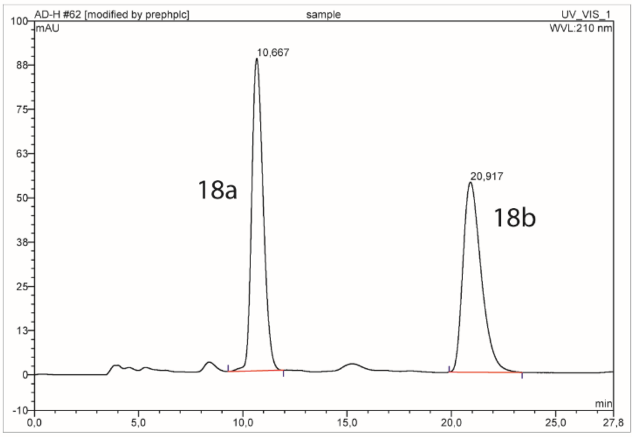

No.	Ret.Time min	Peak Name	Height mAU	Area mAU*min	Rel.Area %	Amount	Type
1	10,67	n.a.	88,237	56,270	50,78	n.a.	BMB
2	20,92	n.a.	53,716	54,550	49,22	n.a.	BMB
**Total:**			141,953	110,820	100,00	0,000	

5 sample_analytisk

Sample Name:	**sample_analytisk**	*Injection Volume:*	**20,0**
Vial Number:	**3**	*Channel:*	**UV_VIS_4**
Sample Type:	**unknown**	*Wavelength:*	**280.0**
Control Program:	**25min-1,0mL_min_10A_90B**	*Bandwidth:*	**4**
Quantif. Method:	**Default1_do not change**	*Dilution Factor:*	**1,0000**
Recording Time:	**23-10-2018 12:09**	*Sample Weight:*	**1,0000**
Run Time (min):	**23,65**	*Sample Amount:*	**1,0000**

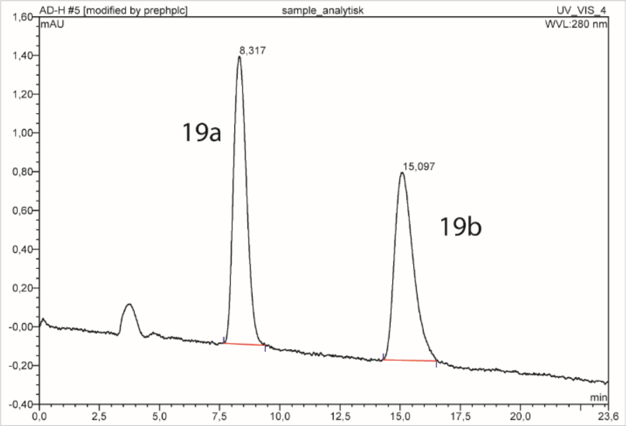

No.	Ret.Time min	Peak Name	Height mAU	Area mAU*min	Rel.Area %	Amount	Type
1	8,32	n.a.	1,486	0,929	51,56	n.a.	BMB*
2	15,10	n.a.	0,969	0,872	48,44	n.a.	BMB*
**Total:**			2,456	1,801	100,00	0,000	

53 sample

Sample Name:	**sample**	*Injection Volume:*	**20,0**
Vial Number:	**48**	*Channel:*	**UV_VIS_1**
Sample Type:	**unknown**	*Wavelength:*	**210.0**
Control Program:	**35min-1,0mL_min_5A_95B**	*Bandwidth:*	**4**
Quantif. Method:	**Default1_do not change**	*Dilution Factor:*	**1,0000**
Recording Time:	**15-12-2018 20:12**	*Sample Weight:*	**1,0000**
Run Time (min):	**21,36**	*Sample Amount:*	**1,0000**

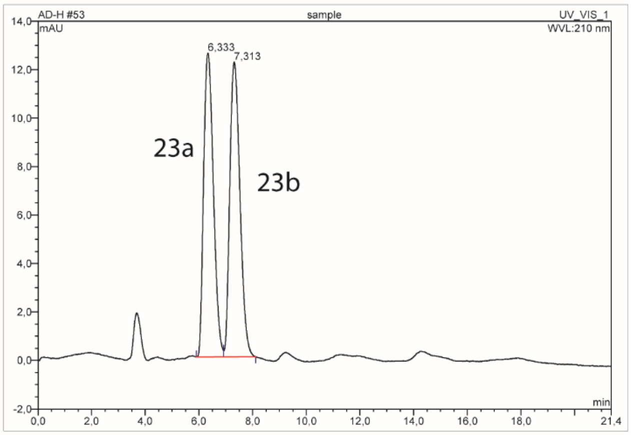

No.	Ret.Time min	Peak Name	Height mAU	Area mAU*min	Rel.Area %	Amount	Type
1	6,33	n.a.	12,536	5,297	49,80	n.a.	BM
2	7,31	n.a.	12,162	5,339	50,20	n.a.	MB
**Total:**			24,697	10,636	100,00	0,000	

Figure S3. Chiral separation of **24** and **25.**

59 sample

Sample Name:	**sample**	*Injection Volume:*	**20,0**
Vial Number:	**54**	*Channel:*	**UV_VIS_1**
Sample Type:	**unknown**	*Wavelength:*	**210.0**
Control Program:	**35min-1,0mL_min_5A_95B**	*Bandwidth:*	**4**
Quantif. Method:	**Default1_do not change**	*Dilution Factor:*	**1,0000**
Recording Time:	**16-12-2018 13:39**	*Sample Weight:*	**1,0000**
Run Time (min):	**28,53**	*Sample Amount:*	**1,0000**

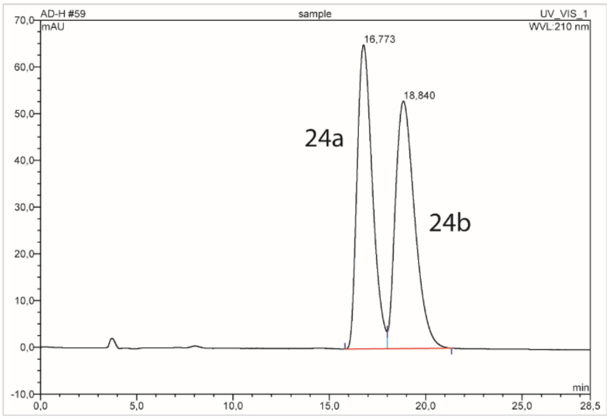

No.	Ret.Time min	Peak Name	Height mAU	Area mAU*min	Rel.Area %	Amount	Type
1	16,77	n.a.	65,027	59,787	49,29	n.a.	BM
2	18,84	n.a.	52,915	61,513	50,71	n.a.	MB
**Total:**			117,942	121,300	100,00	0,000	

66 sample

Sample Name:	**sample**	*Injection Volume:*	**20,0**
Vial Number:	**61**	*Channel:*	**UV_VIS_1**
Sample Type:	**unknown**	*Wavelength:*	**210.0**
Control Program:	**35min-1,0mL_min_10A_90B**	*Bandwidth:*	**4**
Quantif. Method:	**Default1_do not change**	*Dilution Factor:*	**1,0000**
Recording Time:	**16-12-2018 20:54**	*Sample Weight:*	**1,0000**
Run Time (min):	**33,22**	*Sample Amount:*	**1,0000**

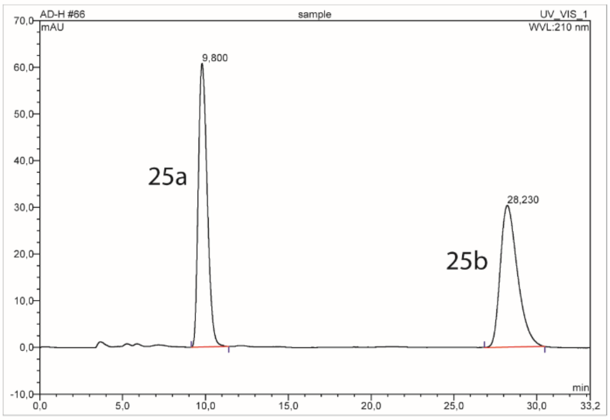

No.	Ret.Time min	Peak Name	Height mAU	Area mAU*min	Rel.Area %	Amount	Type
1	9,80	n.a.	60,731	37,733	50,77	n.a.	BMB
2	28,23	n.a.	30,335	36,586	49,23	n.a.	BMB
**Total:**			91,066	74,319	100,00	0,000	

Scheme 1. Possible biosynthesis of **11**, **14**, **15** and **17** via a [4+2] cycloaddition.

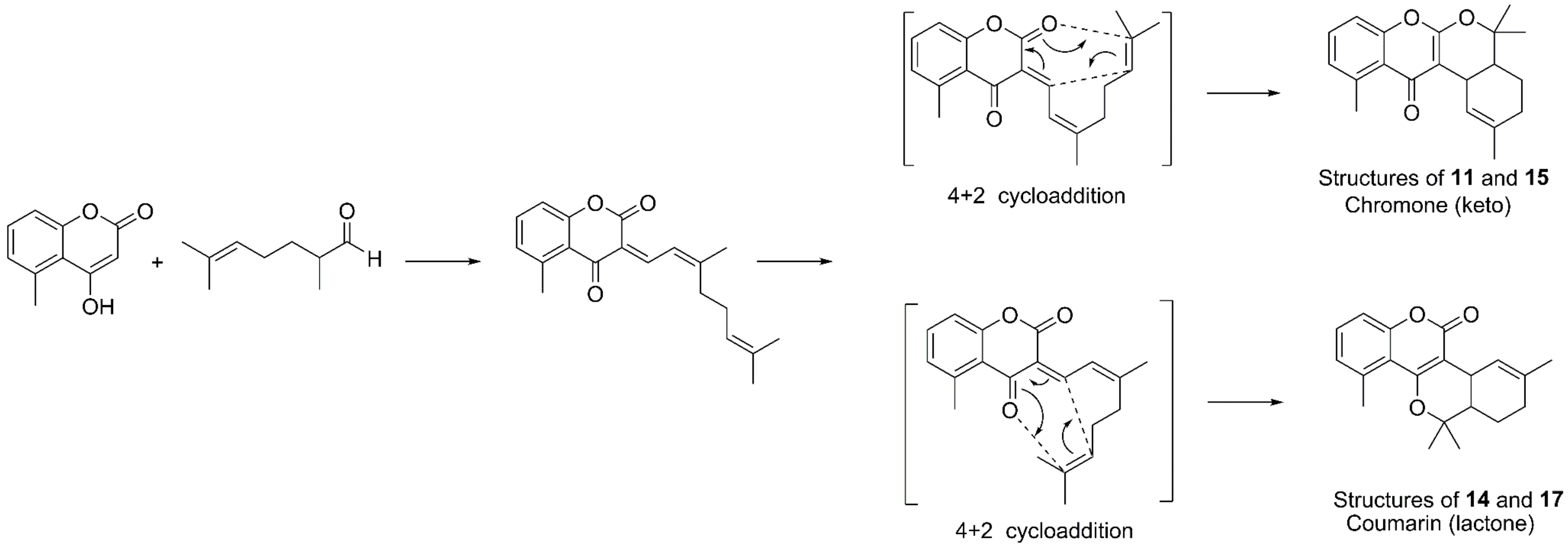

Figure S4. Optimized geometries of the predominant conformers of **6a** at the B3LYP/6-31G(d,p) level in CH_3CN (PCM).

Conformer 1 of **6a**	Conformer 2 of **6a**	Conformer 3 of **6a**	Conformer 4 of **6a**	Conformer 5 of **6a**
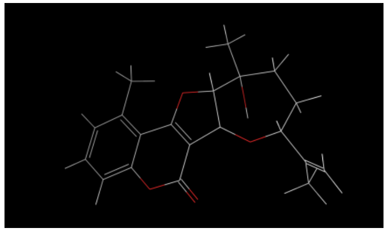	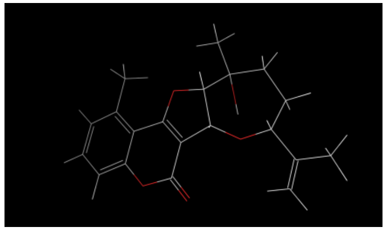	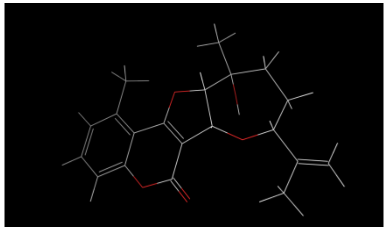	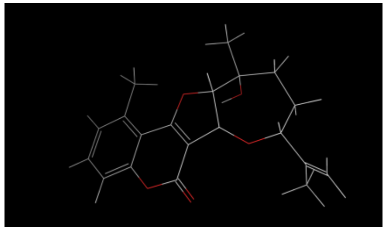	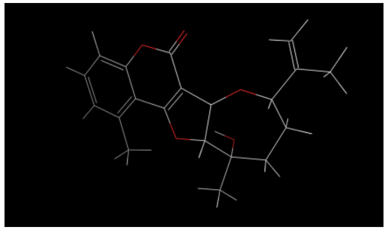

Conformer 6 of **6a**	Conformer 7 of **6a**	Conformer 8 of **6a**	Conformer 9 of **6a**	Conformer 10 of **6a**
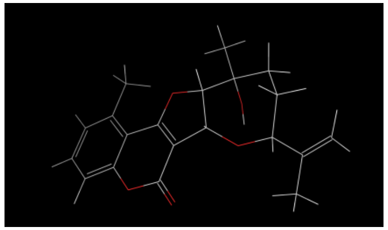	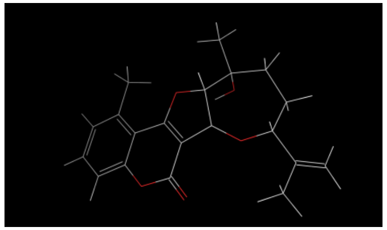	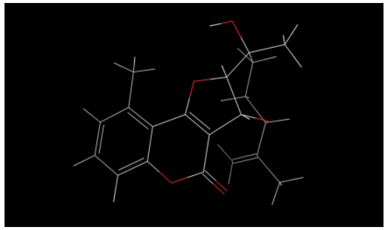	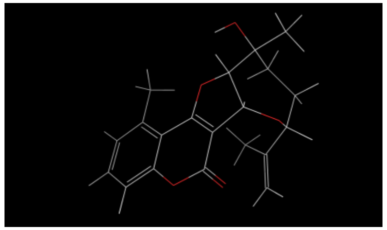	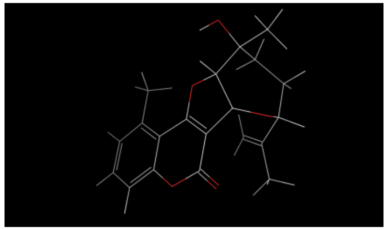

Table S2. Conformational analysis of **6a** at 298K

Conformation	Gibbs Free Energies (in Hartree)	Boltzmann Percentage Weights (%) Population Fractions
**6a**-Conf-1	-1151.194235	20.16%
**6a**-Conf-2	-1151.194780	35.93%
**6a**-Conf-3	-1151.194255	20.59%
**6a**-Conf-4	-1151.190931	0.61%
**6a**-Conf-5	-1151.191999	1.88%
**6a**-Conf-6	-1151.190891	0.58%
**6a**-Conf-7	-1151.177568	0.00%
**6a**-Conf-8	-1151.180052	0.00%
**6a**-Conf-9	-1151.188758	0.06%
**6a**-Conf-10	-1151.194236	20.18%

Table S3. Cartesian coordinates of optimized **6a** conformers. B3LYP/6-31G(d,p) in CH_3CN (PCM)

	**6a**-Conf-1				**6a**-Conf-2		
C	4.964225	0.499828	0.568809	C	4.968104	0.475198	0.575713
C	5.269251	-0.86568	0.643671	C	5.278107	-0.89071	0.611968
C	4.297894	-1.82409	0.393086	C	4.312306	-1.8449	0.326131
C	3.009282	-1.40041	0.064366	C	3.023986	-1.41709	0.001317
C	2.683996	-0.02613	-0.01856	C	2.693784	-0.04194	-0.04209
C	3.686203	0.9478	0.241324	C	3.690318	0.927565	0.253163
O	2.096262	-2.39261	-0.1726	O	2.117555	-2.40561	-0.27287
C	0.757841	-2.15419	-0.50864	C	0.779135	-2.16248	-0.61044
C	0.406699	-0.77345	-0.59815	C	0.423394	-0.78038	-0.65511
C	1.310799	0.227874	-0.37358	C	1.321115	0.217453	-0.39593
C	-0.93728	-0.18177	-0.88961	C	-0.92369	-0.18801	-0.92847
C	-0.59486	1.341521	-0.95023	C	-0.58714	1.337962	-0.94989
O	0.812097	1.459236	-0.53156	O	0.816335	1.450865	-0.51821
C	3.407395	2.430052	0.178523	C	3.405703	2.409932	0.232174
O	0.056394	-3.13268	-0.6955	O	0.081662	-3.13364	-0.83687
O	-1.85978	-0.52574	0.153442	O	-1.84363	-0.56616	0.104837
C	-3.26675	-0.38366	-0.194	C	-3.24586	-0.4239	-0.24752
C	-3.75146	1.016743	0.174617	C	-3.74351	0.972958	0.146541
C	-2.95151	2.159574	-0.46979	C	-2.94928	2.137551	-0.46483
C	-1.45374	2.264295	-0.05502	C	-1.45417	2.239549	-0.0413
C	-3.99045	-1.54949	0.459707	C	-4.01102	-1.54685	0.427621
C	-3.59365	-2.90715	-0.06577	C	-3.77544	-1.76168	1.901523
C	-4.91279	-1.39329	1.414328	C	-4.86546	-2.2913	-0.28133
H	-0.62371	1.694742	-1.98474	H	-0.60974	1.713474	-1.97676
H	-1.35611	-0.52118	-1.84395	H	-1.34131	-0.50372	-1.89143
H	-3.34893	-0.51139	-1.28516	H	-3.33845	-0.54215	-1.33535
O	-1.25935	1.924577	1.318805	O	-1.26485	1.873817	1.32665
C	-0.97981	3.712862	-0.20462	C	-0.9851	3.692632	-0.15974
H	5.740581	1.231015	0.77051	H	5.740127	1.203107	0.804163
H	6.276386	-1.17801	0.901089	H	6.284755	-1.20677	0.866773
H	4.508512	-2.88605	0.445198	H	4.527125	-2.90709	0.347638
H	2.627746	2.721637	0.888235	H	3.0624	2.739578	-0.75253
H	4.31408	2.992305	0.41202	H	4.308405	2.968763	0.488241
H	3.058643	2.732073	-0.81308	H	2.620056	2.67752	0.944696
H	-4.80058	1.114085	-0.12494	H	-4.79133	1.059542	-0.16189
H	-3.72106	1.13717	1.262455	H	-3.72937	1.069503	1.237257
H	-3.01916	2.104052	-1.56442	H	-3.01208	2.10828	-1.56077
H	-3.43658	3.099036	-0.18426	H	-3.44149	3.066632	-0.15799
H	-2.50719	-3.03664	-0.03412	H	-4.03047	-0.87092	2.487715
H	-4.0663	-3.71086	0.504557	H	-2.71896	-1.97538	2.0949
H	-3.8923	-3.01542	-1.11714	H	-4.37285	-2.59625	2.276376
H	-5.41176	-2.25333	1.852307	H	-5.45273	-3.07879	0.182941
H	-5.21089	-0.42101	1.790791	H	-5.0134	-2.13539	-1.3466
H	-1.45671	0.974394	1.388798	H	-1.45186	0.920701	1.377011
H	-1.16169	4.080299	-1.21898	H	-1.16413	4.079813	-1.16721
H	-1.53219	4.344846	0.496223	H	-1.5428	4.308324	0.551318
H	0.086376	3.79841	0.015102	H	0.079808	3.777626	0.066245

	6a-Conf-3				6a-Conf-4		
C	4.996574	0.48926	0.553066	C	4.919849	0.528807	0.673249
C	5.311432	-0.87573	0.580842	C	5.258502	-0.83082	0.672358
C	4.346048	-1.83201	0.300621	C	4.316171	-1.79555	0.346912
C	3.053267	-1.4071	-0.00986	C	3.022454	-1.38434	0.022514
C	2.718054	-0.03297	-0.04461	C	2.661807	-0.01623	0.020211
C	3.714226	0.938692	0.244797	C	3.635954	0.964486	0.352124
O	2.147361	-2.39748	-0.27905	O	2.140309	-2.38182	-0.29454
C	0.804995	-2.15759	-0.60196	C	0.801156	-2.15763	-0.63701
C	0.443789	-0.77659	-0.63766	C	0.415121	-0.78198	-0.65205
C	1.341182	0.223199	-0.38413	C	1.287354	0.221309	-0.34019
C	-0.9073	-0.18662	-0.89728	C	-0.95371	-0.21171	-0.88902
C	-0.57506	1.339574	-0.91644	C	-0.62404	1.312022	-0.94402
O	0.831734	1.45545	-0.49754	O	0.748893	1.450917	-0.41442
C	3.424513	2.420151	0.232707	C	3.324622	2.441684	0.36686
O	0.10859	-3.1307	-0.82423	O	0.128246	-3.14004	-0.89254
O	-1.81633	-0.56419	0.145896	O	-1.81993	-0.62417	0.156632
C	-3.21358	-0.44707	-0.18769	C	-3.2291	-0.45865	-0.11221
C	-3.72187	0.950745	0.202419	C	-3.69256	0.939769	0.289778
C	-2.93591	2.121334	-0.40738	C	-3.00812	2.089754	-0.46478
C	-1.43846	2.232426	0.00413	C	-1.50938	2.31692	-0.17135
C	-3.99117	-1.56775	0.481766	C	-3.9314	-1.6211	0.57491
C	-5.46716	-1.61618	0.166058	C	-3.57719	-2.98042	0.023614
C	-3.40403	-2.462	1.281859	C	-4.7968	-1.46394	1.581318
H	-0.60872	1.719608	-1.9413	H	-0.56086	1.6149	-1.99388
H	-1.33427	-0.50192	-1.85607	H	-1.38157	-0.52325	-1.85123
H	-3.31821	-0.56725	-1.27842	H	-3.3826	-0.58095	-1.19885
O	-1.23505	1.864264	1.369121	O	-1.32012	2.163463	1.234983
C	-0.97963	3.688762	-0.11429	C	-1.1116	3.744584	-0.59043
H	5.768398	1.218799	0.776938	H	5.674834	1.265469	0.928953
H	6.321548	-1.18943	0.824586	H	6.269227	-1.13316	0.92753
H	4.564495	-2.89356	0.315831	H	4.553169	-2.85317	0.336743
H	2.644984	2.682148	0.953984	H	2.547614	2.68232	1.098588
H	4.327813	2.980928	0.482288	H	4.221072	3.010964	0.621746
H	3.070496	2.75267	-0.74722	H	2.96029	2.786258	-0.60486
H	-4.76835	1.047085	-0.10364	H	-4.77085	1.011467	0.107002
H	-3.70608	1.033089	1.295204	H	-3.53276	1.076192	1.362581
H	-3.00763	2.097813	-1.50295	H	-3.13629	1.968773	-1.54852
H	-3.43164	3.045716	-0.09198	H	-3.53027	3.016206	-0.20174
H	-5.64387	-1.58763	-0.91646	H	-2.49215	-3.12268	0.006531
H	-5.91894	-2.52917	0.561183	H	-4.03335	-3.78287	0.609074
H	-6.00573	-0.76309	0.594929	H	-3.92244	-3.07789	-1.01444
H	-3.97995	-3.26079	1.740414	H	-5.27803	-2.32235	2.042141
H	-2.34165	-2.4273	1.489969	H	-5.06265	-0.49132	1.980648
H	-1.42493	0.911715	1.420306	H	-0.37086	2.25732	1.401636
H	-1.16913	4.077454	-1.11926	H	-1.34922	3.933368	-1.64235
H	-1.5352	4.299204	0.602926	H	-1.65693	4.465452	0.024315
H	0.086578	3.779705	0.103272	H	-0.0392	3.910297	-0.44912

	**6a**-Conf-5				**6a**-Conf-6		
C	4.915189	0.516165	0.69252	C	4.955379	0.511552	0.649991
C	5.267037	-0.83911	0.641476	C	5.299628	-0.84605	0.610552
C	4.336715	-1.79923	0.270917	C	4.359609	-1.80563	0.263809
C	3.041562	-1.38832	-0.04838	C	3.062455	-1.39172	-0.04318
C	2.667759	-0.02435	-3.5E-05	C	2.696286	-0.02539	-0.00639
C	3.629569	0.951716	0.37809	C	3.667929	0.950047	0.347319
O	2.172657	-2.38114	-0.41281	O	2.183396	-2.38431	-0.3831
C	0.832919	-2.15672	-0.75703	C	0.840776	-2.15664	-0.71286
C	0.434386	-0.78484	-0.71838	C	0.450459	-0.78212	-0.6886
C	1.293646	0.214104	-0.3615	C	1.318723	0.216135	-0.35269
C	-0.94025	-0.22087	-0.93565	C	-0.92324	-0.21381	-0.90016
C	-0.62177	1.305834	-0.94646	C	-0.60283	1.312812	-0.91202
O	0.742059	1.440294	-0.39293	O	0.772975	1.444509	-0.38828
C	3.303333	2.424106	0.448711	C	3.350208	2.424898	0.404036
O	0.170453	-3.13139	-1.05961	O	0.168119	-3.13188	-0.99062
O	-1.79989	-0.67405	0.09839	O	-1.77983	-0.65972	0.138729
C	-3.20731	-0.51884	-0.17067	C	-3.17968	-0.51495	-0.11966
C	-3.69345	0.877683	0.240441	C	-3.66079	0.877411	0.319452
C	-3.01723	2.04947	-0.48662	C	-2.98751	2.058928	-0.39411
C	-1.52578	2.286685	-0.16581	C	-1.48909	2.286879	-0.10218
C	-3.93515	-1.63108	0.564221	C	-3.9367	-1.64066	0.568574
C	-3.622	-1.81188	2.028176	C	-5.42637	-1.67988	0.322532
C	-4.82101	-2.39604	-0.08154	C	-3.31901	-2.55219	1.325284
H	-0.54474	1.63291	-1.98826	H	-0.54966	1.647836	-1.95258
H	-1.36691	-0.50735	-1.9061	H	-1.35505	-0.50107	-1.86797
H	-3.37494	-0.64855	-1.25073	H	-3.35174	-0.61569	-1.20639
O	-1.35543	2.104456	1.239675	O	-1.29043	2.089978	1.297377
C	-1.13556	3.726403	-0.54842	C	-1.10341	3.729651	-0.47836
H	5.66088	1.249413	0.98299	H	5.708557	1.244289	0.92182
H	6.278617	-1.14166	0.893017	H	6.31288	-1.15092	0.852372
H	4.584294	-2.85332	0.221422	H	4.601083	-2.86154	0.223871
H	2.521413	2.628405	1.186327	H	2.577227	2.642083	1.147298
H	4.193029	2.991897	0.729283	H	4.245878	2.991167	0.668184
H	2.939103	2.802634	-0.51028	H	2.977728	2.793945	-0.55553
H	-4.77105	0.926619	0.04351	H	-4.73803	0.952212	0.137179
H	-3.55489	1.003681	1.317321	H	-3.50737	0.967906	1.398849
H	-3.12818	1.944114	-1.5739	H	-3.12246	1.976095	-1.48064
H	-3.55554	2.964494	-0.2159	H	-3.51501	2.971177	-0.09416
H	-3.86926	-0.91476	2.607967	H	-5.65473	-1.62139	-0.74912
H	-4.17918	-2.65243	2.449283	H	-5.86086	-2.60317	0.713487
H	-2.55191	-1.99347	2.171637	H	-5.94346	-0.83912	0.799597
H	-5.37662	-3.1775	0.42992	H	-3.8771	-3.35652	1.79643
H	-5.02686	-2.26411	-1.14048	H	-2.24772	-2.52523	1.483227
H	-0.40845	2.195146	1.420636	H	-0.34125	2.188635	1.461947
H	-1.36057	3.935742	-1.59921	H	-1.34829	3.949623	-1.5225
H	-1.69594	4.428848	0.074049	H	-1.65007	4.427135	0.161654
H	-0.06677	3.898486	-0.38872	H	-0.03137	3.898208	-0.33793

	**6a**-Conf-7				**6a**-Conf-8		
C	-3.94187	-1.85823	0.672708	C	-4.16098	-1.51527	0.706216
C	-4.77487	-0.76272	0.408485	C	-4.8278	-0.30876	0.455063
C	-4.26153	0.401355	-0.14498	C	-4.15453	0.771562	-0.09677
C	-2.89669	0.460457	-0.43087	C	-2.79812	0.632875	-0.39448
C	-2.04058	-0.6355	-0.17087	C	-2.10776	-0.5768	-0.14581
C	-2.57744	-1.8249	0.393132	C	-2.80801	-1.67897	0.416561
O	-2.45106	1.630137	-0.98517	O	-2.19215	1.72841	-0.94825
C	-1.11193	1.873737	-1.31401	C	-0.83863	1.773099	-1.29976
C	-0.22257	0.793045	-1.03313	C	-0.10809	0.577464	-1.01739
C	-0.66837	-0.3994	-0.53556	C	-0.71556	-0.5384	-0.51266
C	1.271349	0.729762	-1.24876	C	1.356207	0.303598	-1.24989
C	1.474684	-0.80869	-1.15212	C	1.387464	-1.23807	-1.0281
O	0.292626	-1.33286	-0.43729	O	0.107886	-1.59422	-0.38003
C	-1.72232	-3.0314	0.697214	C	-2.13719	-2.99982	0.708475
O	-0.85344	2.960921	-1.80029	O	-0.43664	2.802927	-1.8106
O	2.07819	1.600423	-0.45054	O	2.312486	1.059129	-0.50836
C	2.241751	1.477871	0.987283	C	2.217609	1.198073	0.931492
C	3.20414	0.341813	1.367313	C	1.869863	-0.10093	1.656959
C	2.863356	-1.10294	0.980872	C	2.697979	-1.3209	1.227612
C	2.733495	-1.41668	-0.52091	C	2.550955	-1.85249	-0.2351
C	0.948988	1.629024	1.78109	C	1.384811	2.419205	1.308936
C	0.257425	2.959511	1.588406	C	1.769606	3.691882	0.595033
C	0.517155	0.744334	2.686579	C	0.426839	2.396561	2.242364
H	1.384307	-1.21482	-2.16511	H	1.348062	-1.73749	-2.00062
H	1.538119	1.047049	-2.25997	H	1.643138	0.517641	-2.2827
H	2.799187	2.400814	1.199481	H	3.257033	1.446595	1.191002
O	2.633679	-2.84361	-0.67505	O	2.342564	-3.27609	-0.21314
C	3.962188	-1.00664	-1.33431	C	3.844838	-1.65768	-1.0323
H	-4.36685	-2.75822	1.105736	H	-4.70934	-2.34702	1.136976
H	-5.83376	-0.82511	0.638509	H	-5.88281	-0.21808	0.693305
H	-4.88518	1.261205	-0.3607	H	-4.64785	1.714294	-0.30347
H	-0.92289	-2.78955	1.403693	H	-1.32024	-2.88588	1.427246
H	-2.33426	-3.82551	1.130299	H	-2.86151	-3.70436	1.122777
H	-1.24033	-3.41959	-0.20465	H	-1.7045	-3.43964	-0.19449
H	4.175476	0.598296	0.931449	H	2.056709	0.068671	2.723005
H	3.345315	0.38574	2.453145	H	0.804292	-0.32159	1.57447
H	3.66189	-1.75099	1.35828	H	3.758811	-1.11829	1.414766
H	1.941079	-1.43158	1.469768	H	2.433742	-2.15033	1.890936
H	-0.74021	2.962844	2.034981	H	2.842555	3.894084	0.706214
H	0.843126	3.756224	2.066633	H	1.563973	3.60843	-0.47569
H	0.172974	3.226497	0.532806	H	1.216083	4.54831	0.988875
H	-0.37669	0.940865	3.272712	H	-0.10537	3.303607	2.516005
H	1.029783	-0.18724	2.89481	H	0.136751	1.493558	2.768281
H	1.861064	-3.13408	-0.16813	H	1.498307	-3.43565	0.233847
H	3.864062	-1.36199	-2.3642	H	4.115758	-0.60148	-1.07305
H	4.857331	-1.46111	-0.90048	H	4.64958	-2.21259	-0.54201
H	4.088217	0.07724	-1.34778	H	3.73668	-2.04146	-2.05132

	6a-Conf-9				6a-Conf-10		
C	4.924798	0.757228	0.610139	C	4.964185	0.499908	0.568777
C	5.324729	-0.58398	0.674402	C	5.269331	-0.86558	0.643413
C	4.42874	-1.60493	0.392211	C	4.297984	-1.82403	0.392888
C	3.119583	-1.26955	0.043091	C	3.009286	-1.4004	0.064397
C	2.697996	0.079421	-0.02642	C	2.683932	-0.02615	-0.01842
C	3.624104	1.117806	0.264197	C	3.686092	0.947825	0.241502
O	2.286297	-2.32129	-0.22813	O	2.096246	-2.39262	-0.17243
C	0.942904	-2.17301	-0.59775	C	0.757886	-2.15425	-0.50852
C	0.492233	-0.81986	-0.65554	C	0.40668	-0.77354	-0.59806
C	1.315266	0.240709	-0.40034	C	1.310744	0.227825	-0.37354
C	-0.87704	-0.3264	-1.0009	C	-0.93732	-0.1819	-0.8896
C	-0.67208	1.229265	-0.94445	C	-0.59487	1.341402	-0.95038
O	0.732531	1.435634	-0.55306	O	0.812095	1.459146	-0.53183
C	3.242829	2.577537	0.214301	C	3.407139	2.430055	0.178877
O	0.318679	-3.19102	-0.83381	O	0.056427	-3.13275	-0.69539
O	-1.83475	-0.7996	-0.04393	O	-1.85979	-0.52571	0.153474
C	-3.21223	-0.7455	-0.50224	C	-3.2668	-0.38358	-0.19393
C	-3.53639	0.625949	-1.11245	C	-3.75138	1.016796	0.174876
C	-3.07436	1.86879	-0.32911	C	-2.95146	2.159577	-0.46967
C	-1.57179	1.968334	0.075303	C	-1.45366	2.264277	-0.05515
C	-4.05585	-1.2202	0.669527	C	-3.99051	-1.54949	0.459673
C	-3.62408	-2.53709	1.26914	C	-3.59366	-2.90707	-0.06595
C	-5.13259	-0.5638	1.112839	C	-4.91277	-1.39332	1.41436
H	-0.7687	1.674742	-1.93813	H	-0.62378	1.694536	-1.98492
H	-1.1949	-0.64919	-1.99909	H	-1.3561	-0.52142	-1.84391
H	-3.31368	-1.49963	-1.30076	H	-3.34899	-0.51116	-1.28509
O	-1.34429	1.427358	1.380155	O	-1.25915	1.924687	1.318704
C	-1.17414	3.4444	0.174313	C	-0.97975	3.712832	-0.20495
H	5.643471	1.538912	0.834931	H	5.740493	1.23113	0.770547
H	6.346594	-0.82775	0.947356	H	6.276528	-1.17786	0.900649
H	4.713508	-2.64993	0.433812	H	4.508661	-2.88598	0.444896
H	2.445667	2.807633	0.927345	H	4.313309	2.992358	0.414232
H	4.108056	3.199437	0.453894	H	3.060274	2.732393	-0.81333
H	2.873083	2.863967	-0.77411	H	2.626061	2.721233	0.887133
H	-3.09656	0.657783	-2.11564	H	-4.80056	1.114274	-0.12447
H	-4.61603	0.686571	-1.28163	H	-3.72077	1.137159	1.262714
H	-3.32812	2.739517	-0.94332	H	-3.01929	2.104012	-1.56429
H	-3.63474	1.972725	0.604894	H	-3.43645	3.099067	-0.1841
H	-2.61546	-2.4685	1.687922	H	-3.89211	-3.01518	-1.11738
H	-3.59026	-3.32138	0.502313	H	-2.5072	-3.03657	-0.03406
H	-4.30986	-2.85588	2.057733	H	-4.06641	-3.71086	0.504193
H	-5.7313	-0.96531	1.925431	H	-5.41174	-2.25337	1.852336
H	-5.46666	0.377664	0.691375	H	-5.21083	-0.42104	1.79087
H	-1.58376	0.486101	1.315359	H	-1.4569	0.974626	1.388892
H	-1.23476	3.937055	-0.80033	H	-1.53196	4.34486	0.495984
H	-1.85311	3.955729	0.863003	H	0.086495	3.798385	0.01447
H	-0.1561	3.540344	0.556338	H	-1.16188	4.080197	-1.21929

Figure S5. Optimized geometries of the predominant conformers of **11b** at the B3LYP/6-31G(d,p) level in CH_3CN (PCM).

Conformer 1 of **11b**	Conformer 2 of **11b**
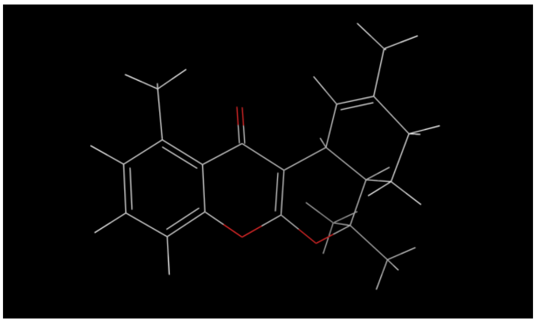	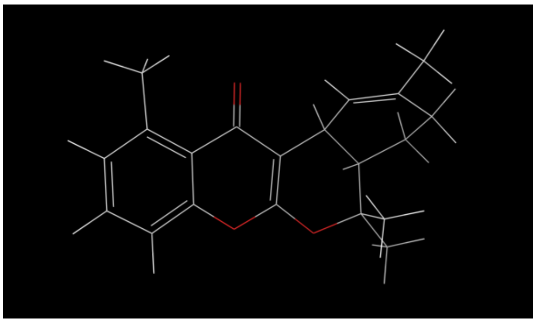

Table S4. Conformational analysis of **11b** at 298K

Conformation	Gibbs Free Energies (in Hartree)	Boltzmann Percentage Weights (%) Population Fractions
**11b**-Conf-1	-1000.768682	88.93%
**11b**-Conf-2	-1000.766881	11.07%

Table S5. Cartesian coordinates of optimized **11b** conformers. B3LYP/6-31G(d,p) in CH_3CN (PCM)

	**11b**-Conf-1				**11b**-Conf-2		
C	4.755122	0.300156	-0.39365	C	-4.97094	0.345207	0.236045
C	4.685218	-0.95137	-1.01741	C	-4.96177	-1.02605	0.518499
C	3.479657	-1.63254	-1.08209	C	-3.77087	-1.73522	0.497549
C	2.355878	-1.03386	-0.51234	C	-2.59886	-1.04399	0.189707
C	2.388114	0.219629	0.1212	C	-2.57096	0.330548	-0.10362
C	3.635598	0.903932	0.181058	C	-3.80355	1.043004	-0.077
O	1.197403	-1.77258	-0.59258	O	-1.45452	-1.81068	0.196454
C	0.042477	-1.24886	-0.13772	C	-0.26675	-1.23622	-0.07559
C	-0.0828	-0.00437	0.403487	C	-0.1046	0.082484	-0.37183
C	1.122592	0.762154	0.68417	C	-1.26431	0.953579	-0.4408
O	1.093042	1.806071	1.356498	O	-1.15516	2.144807	-0.77876
C	3.797919	2.257026	0.831878	C	-3.89968	2.52139	-0.36988
O	-0.92544	-2.14663	-0.30417	O	0.679263	-2.16694	-0.02824
C	-2.16791	-1.9232	0.471001	C	2.092618	-1.70977	0.034108
C	-2.5816	-0.44226	0.306756	C	2.250998	-0.56214	-1.00056
C	-1.46217	0.502221	0.823919	C	1.262103	0.604399	-0.76784
C	-2.97781	-0.06986	-1.13415	C	3.677177	-0.00276	-1.18438
C	-3.50805	1.364856	-1.21008	C	4.165371	0.928489	-0.06205
C	-2.63287	2.34882	-0.47241	C	3.087046	1.829293	0.494843
C	-1.70995	1.942733	0.408611	C	1.795424	1.663426	0.181577
C	-1.88373	-2.29562	1.931332	C	2.873146	-2.95582	-0.38155
C	-3.15845	-2.91572	-0.13459	C	2.356417	-1.37077	1.503777
C	-2.86339	3.80771	-0.77195	C	3.550191	2.913904	1.433194
H	-1.48496	0.497074	1.923754	H	1.116371	1.099262	-1.7408
H	-3.46146	-0.29354	0.945695	H	1.971379	-1.029	-1.95363
H	5.707463	0.820318	-0.35407	H	-5.91255	0.885443	0.260117
H	5.577607	-1.39064	-1.45197	H	-5.88973	-1.53636	0.756502
H	3.385608	-2.60413	-1.55387	H	-3.72313	-2.79672	0.712796
H	4.825653	2.608158	0.706273	H	-4.93389	2.856394	-0.25414
H	3.115865	2.994826	0.402527	H	-3.26079	3.105649	0.296908
H	3.565485	2.218331	1.899122	H	-3.56455	2.751102	-1.38436
H	-2.10597	-0.17219	-1.79146	H	4.395489	-0.81333	-1.34096
H	-3.74199	-0.75311	-1.51288	H	3.660852	0.5685	-2.12006
H	-4.52945	1.411937	-0.80136	H	4.987406	1.547163	-0.44787
H	-3.60222	1.670273	-2.26076	H	4.613135	0.356738	0.762807
H	-1.08318	2.675011	0.906169	H	1.052229	2.344592	0.588003
H	-2.79492	-2.17645	2.524729	H	2.66177	-3.22324	-1.42061
H	-1.56615	-3.33971	1.995466	H	3.94724	-2.78785	-0.27583
H	-1.10472	-1.6728	2.375167	H	2.598013	-3.79881	0.258076
H	-3.24217	-2.79477	-1.2156	H	3.413458	-1.15157	1.664772
H	-2.83321	-3.93945	0.069178	H	1.774392	-0.5095	1.834674
H	-4.14708	-2.77458	0.311143	H	2.093481	-2.23396	2.12174
H	-2.68459	4.026445	-1.8329	H	4.087479	2.489765	2.291907
H	-3.90501	4.090864	-0.56979	H	4.252734	3.594621	0.9347
H	-2.21379	4.453631	-0.17469	H	2.713316	3.505787	1.814352

Figure S6. Optimized geometries of the predominant conformers of **14b** at the B3LYP/6-31G(d,p) level in CH_3CN (PCM).

Conformer 1 of **14b**	Conformer 2 of **14b**
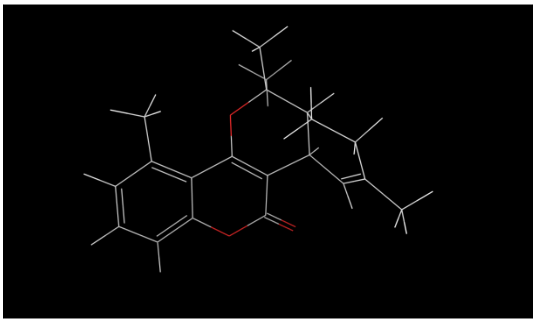	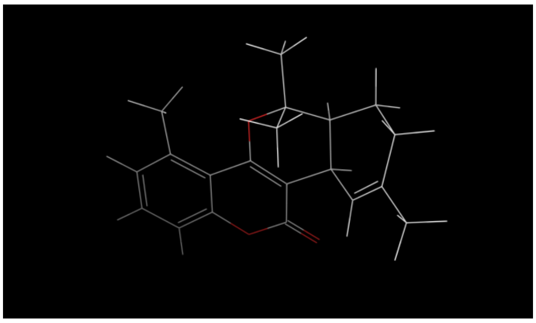

Table S6. Conformational analysis of **14b** at 298K

Conformation	Gibbs Free Energies (in Hartree)	Boltzmann Percentage Weights (%) Population Fractions
**14b**-Conf-1	-1001.338791	8.57%
**14b**-Conf-2	-1001.340836	91.43%

Table S7. Cartesian coordinates of the optimized **14b** conformers. B3LYP/6-31G(d,p) in CH_3CN (PCM)

	**14b**-Conf-1				**14b**-Conf-2		
C	4.50405	0.826642	0.42911	C	3.67115	-1.91252	-0.21664
C	4.963438	-0.49471	0.411895	C	2.40256	-1.41496	0.07289
C	4.075702	-1.53487	0.189376	C	2.04851	-0.07039	-0.13834
C	2.728936	-1.23117	-0.01401	C	3.03294	0.81	-0.67381
C	2.235434	0.089688	0.003998	C	4.29477	0.29188	-0.95897
C	3.160936	1.151735	0.231822	C	4.61829	-1.04847	-0.73539
O	1.915159	-2.30823	-0.23315	O	1.52293	-2.31643	0.59869
C	0.55832	-2.18306	-0.48325	C	0.20632	-2.00116	0.90147
C	-0.00782	-0.8591	-0.43403	C	-0.24003	-0.66353	0.59931
C	0.796064	0.231404	-0.20843	C	0.67498	0.28324	0.2097
C	-1.47979	-0.72216	-0.77252	C	-1.70954	-0.32015	0.83457
C	-1.84015	0.753887	-1.04373	C	-2.01802	1.11771	0.3513
C	-1.12992	1.723312	-0.06524	C	-0.87042	2.06687	0.74945
O	0.325293	1.48618	-0.18485	O	0.37168	1.58386	0.11478
O	-0.04599	-3.21693	-0.72818	C	-2.65744	-1.33328	0.2138
C	2.779369	2.614759	0.2705	C	-3.50153	-1.08569	-0.79135
C	-2.39771	-1.38808	0.239264	C	-3.55013	0.25912	-1.47158
C	-3.61165	-0.9377	0.581564	C	-2.32992	1.12617	-1.15526
C	-4.18546	0.333328	-0.00065	C	2.79235	2.27296	-0.96845
C	-3.37027	0.900326	-1.17473	O	-0.44553	-2.88676	1.41878
C	-1.45679	1.579191	1.425013	C	-1.03283	3.48784	0.21539
C	-1.28289	3.178475	-0.50897	C	-0.64207	2.10785	2.26553
C	-4.47908	-1.65828	1.581335	C	-4.46522	-2.12639	-1.29828
H	5.212278	1.63098	0.601782	H	-1.88458	-0.36622	1.91736
H	6.016345	-0.70488	0.57094	H	-2.91232	1.46188	0.88266
H	4.390413	-2.57198	0.16522	H	3.88073	-2.95744	-0.02751
H	-1.61386	-1.26406	-1.72197	H	5.04665	0.95526	-1.37017
H	-1.41556	1.01086	-2.02263	H	5.61184	-1.4119	-0.96997
H	2.313257	2.938465	-0.66316	H	-2.6406	-2.31719	0.66644
H	2.061609	2.826329	1.066979	H	-4.47138	0.77889	-1.17227
H	3.672065	3.22152	0.44013	H	-3.63403	0.11774	-2.55553
H	-2.03004	-2.31657	0.668726	H	-1.46435	0.75247	-1.7122
H	-5.21224	0.141427	-0.34148	H	-2.51552	2.1435	-1.50303
H	-4.29466	1.075004	0.802981	H	1.95609	2.41628	-1.6543
H	-3.65159	0.353224	-2.08241	H	2.55378	2.83343	-0.06321
H	-3.64592	1.943581	-1.35649	H	3.6873	2.70543	-1.41814
H	-1.30193	0.559677	1.78065	H	-1.97869	3.9094	0.56331
H	-0.81281	2.253368	1.996959	H	-0.22059	4.11884	0.58153
H	-2.49367	1.85652	1.623313	H	-1.01888	3.51338	-0.87358
H	-2.31298	3.515371	-0.37138	H	-1.541	2.48238	2.76109
H	-0.63395	3.822845	0.090011	H	-0.4094	1.12649	2.6792
H	-1.01509	3.297422	-1.56272	H	0.18465	2.78189	2.49823
H	-5.43265	-1.96258	1.130335	H	-4.27997	-2.34996	-2.35534
H	-3.9875	-2.55158	1.976648	H	-4.39201	-3.05696	-0.73185
H	-4.73154	-1.00478	2.426971	H	-5.49861	-1.76563	-1.23283

Table S8. Cartesian coordinates of the optimized **15b.** B3LYP/6-31G(d,p) in CH_3CN (PCM)

C	4.899619	0.469312	-0.23382
C	4.933224	-0.84784	-0.70724
C	3.766098	-1.59196	-0.78392
C	2.575285	-0.98881	-0.37905
C	2.503588	0.328764	0.102361
C	3.712426	1.077588	0.176934
O	1.458164	-1.78859	-0.46457
C	0.247376	-1.27722	-0.16962
C	0.030069	0.014317	0.215714
C	1.175108	0.86412	0.503917
O	1.046923	1.971908	1.052349
C	3.763783	2.502866	0.673345
O	-0.65805	-2.24041	-0.31504
C	-2.0205	-2.00205	0.227646
C	-2.38901	-0.5506	-0.1285
C	-1.39795	0.462757	0.481133
C	-3.81929	-0.11136	0.212959
C	-4.12458	1.234518	-0.46782
C	-2.95955	2.202682	-0.45837
C	-1.73539	1.850192	-0.04089
C	-2.87311	-3.02625	-0.51705
C	-1.97537	-2.30417	1.726759
C	-3.25853	3.591075	-0.96388
H	-1.54103	0.479492	1.57442
H	-2.27769	-0.48688	-1.22073
H	5.823038	1.038362	-0.18331
H	5.875883	-1.28865	-1.01587
H	3.751427	-2.61478	-1.14306
H	3.449401	2.573813	1.717601
H	4.781919	2.890911	0.583326
H	3.086306	3.149892	0.110942
H	-4.55422	-0.85583	-0.10741
H	-3.92132	-0.01011	1.300419
H	-4.99517	1.704925	0.008845
H	-4.42622	1.063104	-1.5125
H	-0.93409	2.580917	-0.03278
H	-2.44604	-4.02515	-0.39352
H	-2.91876	-2.79554	-1.58509
H	-3.88997	-3.04143	-0.11864
H	-1.26273	-1.66264	2.25039
H	-2.96316	-2.15052	2.170159
H	-1.68535	-3.34543	1.890913
H	-2.36738	4.224795	-0.96031
H	-3.65282	3.56126	-1.9884
H	-4.02814	4.078597	-0.35107

Table S9. Cartesian coordinates of the optimized **17b**. B3LYP/6-31G(d,p) in CH_3CN (PCM)

C	-4.5013	-2.183	-3.6711
C	-4.4555	-3.5145	-3.265
C	-5.1064	-3.8933	-2.1022
C	-5.7955	-2.9315	-1.3611
C	-5.865	-1.5852	-1.7315
C	-5.1952	-1.2071	-2.921
O	-6.4445	-3.4147	-0.2131
C	-7.2256	-2.5962	0.5798
C	-7.1684	-1.1407	0.3162
C	-6.6122	-0.6885	-0.8245
C	-7.8752	-0.211	1.2796
C	-7.5271	1.2605	0.9423
C	-7.6317	1.5019	-0.5728
O	-6.7099	0.6196	-1.2461
C	-7.5932	-0.4312	2.7562
C	-7.6399	0.554	3.6749
C	-7.9597	1.9851	3.3129
C	-8.3528	2.1878	1.8428
O	-7.9409	-3.0783	1.4515
C	-7.1543	2.917	-0.9399
C	-9.0365	1.3021	-1.1606
H	-6.4656	1.4181	1.2016
H	-8.9531	-0.3813	1.1461
C	-5.1691	0.2038	-3.4497
C	-7.3794	0.2936	5.1315
H	-3.9837	-1.9029	-4.5874
H	-3.9154	-4.2492	-3.856
H	-5.0858	-4.9283	-1.7728
H	-7.4021	-1.4458	3.0988
H	-8.7692	2.3614	3.9494
H	-7.0731	2.5958	3.5267
H	-9.423	1.9804	1.719
H	-8.2025	3.2414	1.5872
H	-7.0666	3.0265	-2.0277
H	-7.8345	3.6932	-0.5773
H	-6.1548	3.1097	-0.5333
H	-9.401	0.2792	-1.026
H	-9.7632	1.9886	-0.7154
H	-9.0259	1.4717	-2.244
H	-4.7014	0.8781	-2.7254
H	-4.5819	0.2776	-4.372
H	-6.1811	0.5421	-3.693
H	-7.1467	-0.7582	5.3282
H	-6.531	0.8929	5.4774
H	-8.2589	0.558	5.7274

Table S10. Cartesian coordinates of the optimized **18b**. B3LYP/6-31G(d,p) in CH_3CN (PCM)

C	6.53727	0.34714	-0.75713
C	6.76473	-0.97659	-1.14931
C	5.71659	-1.88241	-1.17786
C	4.44501	-1.44189	-0.80885
C	4.18478	-0.11449	-0.41368
C	5.27263	0.80847	-0.38824
O	3.45653	-2.38342	-0.87437
C	2.1454	-2.12893	-0.50291
C	1.84299	-0.81206	0.00379
C	2.79902	0.17748	-0.05533
C	0.43918	-0.52524	0.50363
C	0.3816	0.87961	1.13245
C	1.1185	1.91411	0.25686
O	2.52946	1.46314	0.21397
O	1.35816	-3.05114	-0.65724
C	5.14454	2.25657	0.02806
C	-0.0634	-1.5646	1.53333
C	-1.4225	-1.19356	2.14934
C	-1.39714	0.21079	2.7476
C	-1.06015	1.21018	1.62655
C	-2.16824	1.0851	0.60187
O	-2.43337	-1.23751	1.06551
C	-2.79534	-0.12634	0.41013
C	-3.90356	-0.27316	-0.52807
C	-4.32658	0.91543	-1.15851
O	-3.73717	2.12356	-0.90493
C	-2.66333	2.27002	-0.04458
C	-4.57737	-1.48819	-0.85305
C	-5.6235	-1.43516	-1.77515
C	-6.02469	-0.23967	-2.38166
C	-5.37636	0.94637	-2.07706
O	-2.22922	3.40713	0.08954
C	-4.22191	-2.83489	-0.2646
C	-1.89021	-2.25087	3.14281
C	1.19879	3.28692	0.91856
C	0.64517	2.03747	-1.19389
H	0.98691	0.84465	2.05009
H	-0.24397	-0.5621	-0.35546
H	-1.07629	2.2277	2.02257
H	7.36979	1.04339	-0.73588
H	7.7638	-1.29454	-1.43013
H	5.85001	-2.91613	-1.47586
H	6.12878	2.73067	0.01299
H	4.4826	2.81323	-0.64008
H	4.72784	2.35595	1.03322
H	0.66127	-1.64451	2.35342
H	-0.13464	-2.54647	1.06434
H	-2.3677	0.44848	3.19523
H	-0.64701	0.24171	3.54371
H	-6.13917	-2.35617	-2.02826
H	-6.84384	-0.24182	-3.09369
H	-5.65522	1.89269	-2.5263
H	-3.17773	-3.09704	-0.45165

H	-4.35879	-2.85279	0.81953
H	-4.85693	-3.60675	-0.70568
H	-1.18377	-2.3188	3.9747
H	-2.87456	-1.99339	3.54339
H	-1.95207	-3.23107	2.66211
H	1.54594	3.20539	1.9529
H	1.89516	3.9255	0.36815
H	0.21455	3.75926	0.90678
H	-0.34285	2.49738	-1.22514
H	1.33687	2.68342	-1.74215
H	0.6044	1.0724	-1.70335

Table S11. Cartesian coordinates of the optimized **19a**. B3LYP/6-31G(d,p) in CH_3CN (PCM)

C	-6.61388	-1.35549	-1.08761
C	-5.98186	-2.51813	-0.63047
C	-4.64882	-2.52113	-0.21748
C	-3.91974	-1.2988	-0.26285
C	-4.58765	-0.15095	-0.72315
C	-5.91931	-0.15687	-1.13822
C	-2.49251	-1.17431	0.130948
C	-1.92758	0.163995	0.073721
C	-2.68124	1.193826	-0.40187
O	-3.96332	1.075057	-0.79424
C	-0.46682	0.384472	0.39339
C	0.003037	1.772744	-0.11528
C	-1.04425	2.908067	0.066847
O	-2.29567	2.451779	-0.58836
O	-1.80412	-2.15054	0.474373
C	-4.04178	-3.81763	0.262787
C	-0.12149	0.085233	1.872992
C	1.321263	0.445056	2.262431
C	1.618531	1.898348	1.907241
C	1.445491	2.080435	0.392001
C	-1.44504	3.301382	1.491864
C	-0.66827	4.152018	-0.73907
O	2.245888	-0.43695	1.514189
C	2.764536	-0.05286	0.338266
C	2.423496	1.132371	-0.26756
C	2.968802	1.492721	-1.54793
O	3.875387	0.613697	-2.1177
C	4.240223	-0.56311	-1.52181
C	3.718611	-0.97167	-0.27565
C	5.161811	-1.32938	-2.23637
C	5.578705	-2.53703	-1.69995
C	5.077081	-2.9677	-0.4669
C	4.1546	-2.21795	0.264302
O	2.707728	2.502155	-2.1859
C	1.601748	0.122406	3.725725
C	3.677391	-2.77829	1.585198
H	0.08197	-0.3616	-0.19385
H	0.086561	1.684892	-1.2051
H	1.720765	3.095868	0.098192
H	-7.65203	-1.39132	-1.40233
H	-6.54241	-3.44738	-0.59467
H	-6.37451	0.764255	-1.48411
H	-3.19576	-4.11859	-0.35991
H	-4.79509	-4.60967	0.244467
H	-3.64952	-3.72533	1.278584
H	-0.78386	0.637402	2.547738

H	-0.2966	-0.97744	2.055222
H	2.643861	2.1549	2.194481
H	0.952197	2.54025	2.485294
H	-2.26535	4.02258	1.441174
H	-0.61492	3.781214	2.013647
H	-1.78267	2.446642	2.080553
H	-0.38951	3.889645	-1.7628
H	0.170755	4.672941	-0.27229
H	-1.51635	4.841019	-0.77186
H	5.525462	-0.96115	-3.18891
H	6.295033	-3.15012	-2.23758
H	5.412932	-3.91663	-0.0606
H	1.421903	-0.93725	3.927614
H	2.639684	0.356303	3.978508
H	0.94592	0.71237	4.37199
H	2.59425	-2.92235	1.59712
H	4.15693	-3.74261	1.769
H	3.91263	-2.1111	2.41818

Figure S7. Optimized geometries of the predominant conformers of **23b** at the B3LYP/6-31G(d,p) level in CH_3CN solvent as described by PCM.

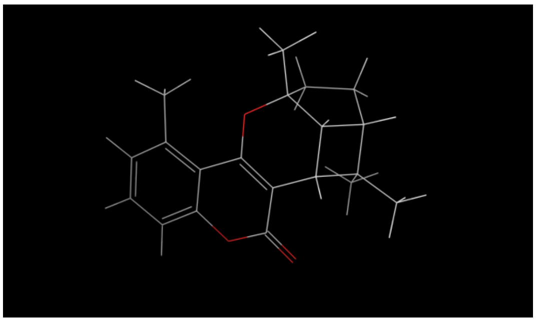

Conformer 1 of **23b**

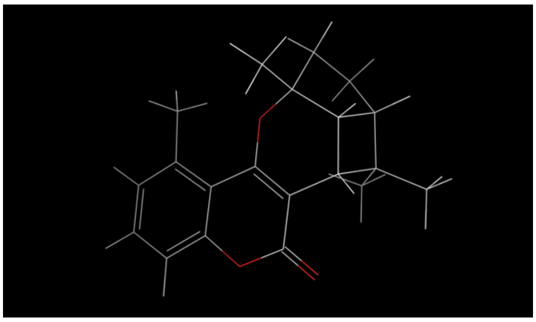

Conformer 2 of **23b**

Table S12. Conformational analysis of **23b** at 298K

Conformation	Gibbs Free Energies (in Hartree)	Boltzmann Percentage Weights (%) Population Fractions
**23b**-Conf-1	-1000.765921	60.87
**23b**-Conf-2	-1000.765539	39.13

Table S13. Cartesian coordinates of the optimized **23b** conformers. B3LYP/6-31G(d,p) in CH_3CN (PCM).

	**23b**-Conf-1				**23b**-Conf-2		
C	4.36793	0.800757	0.2986	C	4.129357	-1.05003	-0.59345
C	4.788291	-0.52969	0.401356	C	4.716074	0.208761	-0.4233
C	3.871251	-1.55938	0.264871	C	3.934283	1.297261	-0.07074
C	2.535348	-1.23639	0.024054	C	2.563971	1.105294	0.10918
C	2.081267	0.094995	-0.08129	C	1.945766	-0.15367	-0.04803
C	3.036333	1.145716	0.061407	C	2.761769	-1.26467	-0.41563
O	1.690931	-2.30577	-0.10128	O	1.858506	2.226074	0.454245
C	0.338908	-2.16446	-0.36518	C	0.484137	2.223136	0.63011
C	-0.18593	-0.82434	-0.44957	C	-0.19213	0.956533	0.50559
C	0.650175	0.258819	-0.32482	C	0.505931	-0.181	0.189013
C	-1.62685	-0.67658	-0.83489	C	-1.67578	0.932135	0.669603
C	-2.12505	0.784984	-0.94766	C	-2.27509	-0.46918	0.950056
C	-1.20676	1.831042	-0.28542	C	-1.35244	-1.68946	0.774774
O	0.218191	1.525672	-0.44034	O	-0.10769	-1.36698	0.037888
C	-2.80092	-1.00973	0.176566	C	-2.6017	1.053545	-0.62538
C	-3.27777	0.489692	0.064393	C	-3.16563	-0.38799	-0.32321
C	-1.57542	1.817478	1.218184	C	-2.14146	-2.58922	-0.18775
C	-3.06492	1.438072	1.258678	C	-2.78988	-1.61358	-1.18988
H	-4.29268	0.571877	-0.33465	H	-4.24051	-0.3727	-0.12046
H	-2.40688	1.088069	-1.95827	H	-2.80128	-0.54514	1.90441
H	-1.77728	-1.23174	-1.76707	H	-1.95848	1.678192	1.415387
C	-3.81416	-1.96885	-0.45812	C	-3.67374	2.131519	-0.43647
C	-2.41283	-1.52614	1.562107	C	-1.88236	1.262879	-1.95563
C	-1.3663	3.211457	-0.91233	C	-0.91829	-2.35083	2.078845
C	2.696732	2.616908	-0.02861	C	2.233963	-2.66544	-0.63107
O	-0.29883	-3.1967	-0.5109	O	-0.03748	3.299614	0.87847
H	5.09832	1.596452	0.407138	H	4.754473	-1.89201	-0.87398
H	5.833545	-0.75536	0.587332	H	5.784241	0.333358	-0.57014
H	4.155236	-2.60317	0.336573	H	4.349139	2.289266	0.067521
H	-0.96878	1.060982	1.725031	H	-1.50431	-3.34932	-0.6498
H	-1.35877	2.777681	1.69542	H	-2.91436	-3.10774	0.391764
H	-3.68316	2.332496	1.119554	H	-3.65655	-2.05231	-1.69209
H	-3.3582	0.997523	2.216606	H	-2.0669	-1.35256	-1.96768
H	-4.71242	-2.05491	0.164687	H	-4.41313	2.090561	-1.24508
H	-3.37801	-2.96704	-0.57257	H	-3.22643	3.131923	-0.43515
H	-4.12773	-1.62168	-1.44904	H	-4.20822	2.001799	0.511186
H	-1.65702	-0.9097	2.055099	H	-1.06904	0.550789	-2.11658
H	-3.29113	-1.56432	2.216544	H	-2.58507	1.164355	-2.79112
H	-2.00858	-2.53948	1.481857	H	-1.45021	2.268902	-1.99952
H	-2.40177	3.54896	-0.81008	H	-1.79562	-2.66762	2.650006
H	-0.71655	3.938758	-0.41731	H	-0.29886	-3.23	1.880258
H	-1.11857	3.183466	-1.97725	H	-0.34429	-1.65314	2.696586
H	1.972309	2.913865	0.733847	H	3.051263	-3.32122	-0.94086
H	2.256125	2.874707	-0.99467	H	1.456876	-2.69433	-1.39838
H	3.603733	3.210838	0.107356	H	1.789894	-3.07676	0.279048

Table S14. Cartesian coordinates of the optimized **24a**. B3LYP/6-31G(d,p) in CH_3CN (PCM).

C	6.654666	-0.1966	-2.11318
C	6.630926	-1.31228	-1.26781
C	5.58222	-1.54285	-0.37599
C	4.506121	-0.61104	-0.33697
C	4.563388	0.495477	-1.2003
C	5.616351	0.721579	-2.08642
C	3.329251	-0.74817	0.562059
C	2.257986	0.216411	0.362327
C	2.479757	1.291474	-0.44805
O	3.575153	1.454505	-1.21268
C	0.957518	0.141342	1.136819
C	-0.06911	1.145933	0.569905
C	0.561017	2.535444	0.328724
O	1.684411	2.34036	-0.62286
O	3.26253	-1.6332	1.431673
C	-0.36065	3.483422	-0.43072
C	1.137245	3.194233	1.583943
C	-1.3953	1.154168	1.427389
C	0.317856	-1.26546	1.145374
C	-1.09549	-1.27597	1.787539
C	-1.37171	0.045011	2.49844
O	-2.09221	-1.46371	0.69537
C	-2.86081	-0.46061	0.250922
C	-2.60371	0.852215	0.571563
C	-4.81713	0.236641	-1.00018
C	-3.98213	-0.83011	-0.60571
O	-4.59974	1.523548	-0.58998
C	-3.52494	1.891614	0.204777
O	-3.46972	3.070168	0.528298
C	-4.29276	-2.14178	-1.07155
C	-5.40826	-2.30247	-1.89447
C	-6.22057	-1.22614	-2.26866
C	-5.92934	0.05318	-1.82284
C	-3.47805	-3.36978	-0.73357
C	-1.30184	-2.49426	2.680548
C	5.633736	-2.77286	0.4987
H	1.181988	0.402143	2.17952
H	-0.3323	0.781429	-0.43128
H	-1.53887	2.126363	1.902774
H	7.488248	-0.0493	-2.79255
H	7.451397	-2.02254	-1.30441
H	5.599055	1.598422	-2.72372
H	0.174367	4.406489	-0.67114
H	-1.24251	3.72919	0.162531
H	-0.69451	3.023194	-1.36521
H	1.898591	2.570922	2.058886
H	1.591022	4.154479	1.325558

H	0.344236	3.37988	2.313661
H	0.235575	-1.64653	0.121818
H	0.974744	-1.9454	1.687555
H	-0.60171	0.209871	3.255881
H	-2.33369	0.013821	3.01857
H	-5.64866	-3.29785	-2.25473
H	-7.07992	-1.39356	-2.91018
H	-6.53409	0.912413	-2.08969
H	-2.43378	-3.26072	-1.03633
H	-3.47187	-3.56904	0.341037
H	-3.89716	-4.24033	-1.24367
H	-1.11301	-3.41566	2.122058
H	-0.60582	-2.4561	3.523357
H	-2.32287	-2.52237	3.071146
H	5.667927	-2.50894	1.558672
H	4.743864	-3.39421	0.372399
H	6.518761	-3.3659	0.253227

Table S15. Cartesian coordinates of the optimized **25b**. B3LYP/6-31G(d,p) in CH_3CN (PCM).

C	-5.86526	-1.40462	-1.92256
C	-6.496	-0.16969	-2.10916
C	-5.92893	0.984509	-1.59349
C	-4.72676	0.880357	-0.89273
C	-4.0695	-0.34891	-0.68711
C	-4.66404	-1.5304	-1.22286
O	-4.23319	2.05488	-0.3986
C	-3.0351	2.139629	0.293173
C	-2.26781	0.926369	0.442699
C	-2.8165	-0.27733	0.061239
C	-0.91981	0.990897	1.132582
C	-0.18267	-0.35512	0.993835
C	-1.12293	-1.53847	1.295672
O	-2.24463	-1.45685	0.336712
O	-2.74857	3.245589	0.727786
C	-4.06767	-2.91383	-1.09198
C	0.003425	2.11414	0.605405
C	1.435875	2.037071	1.199883
C	1.481886	1.051736	2.363227
C	1.176858	-0.34961	1.79707
C	2.34571	-0.67578	0.896574
O	2.35298	1.569469	0.122831
C	2.849728	0.325254	0.099153
C	3.947978	0.080378	-0.82895
C	4.497972	-1.21821	-0.78335
O	4.044613	-2.17249	0.085817
C	2.987458	-1.95962	0.957364
C	4.493052	1.014836	-1.75829
C	5.543608	0.595765	-2.57564
C	6.070897	-0.69872	-2.50804
C	5.550088	-1.61598	-1.60935
O	2.708059	-2.88626	1.705471
C	3.993486	2.432962	-1.91722
C	1.972361	3.41816	1.559534
C	-1.72219	-1.52443	2.705013
C	-0.49464	-2.89073	0.972175
H	1.146117	-1.07971	2.608033
H	0.068543	-0.46088	-0.06927
H	-1.10477	1.194877	2.19548
H	-6.32077	-2.29918	-2.33549
H	-7.43021	-0.11596	-2.65907
H	-6.38532	1.960363	-1.71421
H	-3.05754	-2.96215	-1.50579
H	-3.99192	-3.22578	-0.04739

H	-4.69379	-3.63444	-1.62342
H	-0.43959	3.080365	0.845269
H	0.078368	2.056457	-0.48576
H	0.761241	1.363427	3.123114
H	2.468518	1.052838	2.83593
H	5.961189	1.30146	-3.28697
H	6.888959	-0.98517	-3.16132
H	5.931279	-2.62739	-1.52599
H	4.547245	2.929602	-2.71746
H	2.928995	2.464121	-2.16217
H	4.119344	3.01243	-0.99913
H	1.359139	3.858044	2.351185
H	3.005518	3.35421	1.912079
H	1.93733	4.081628	0.690573
H	-2.3904	-2.38002	2.832924
H	-0.93078	-1.59795	3.456242
H	-2.29252	-0.61281	2.89792
H	0.357275	-3.0919	1.623371
H	-1.23503	-3.68503	1.1018
H	-0.14403	-2.90981	-0.06393

Figure S8. ECD spectra of **2** and **8**.

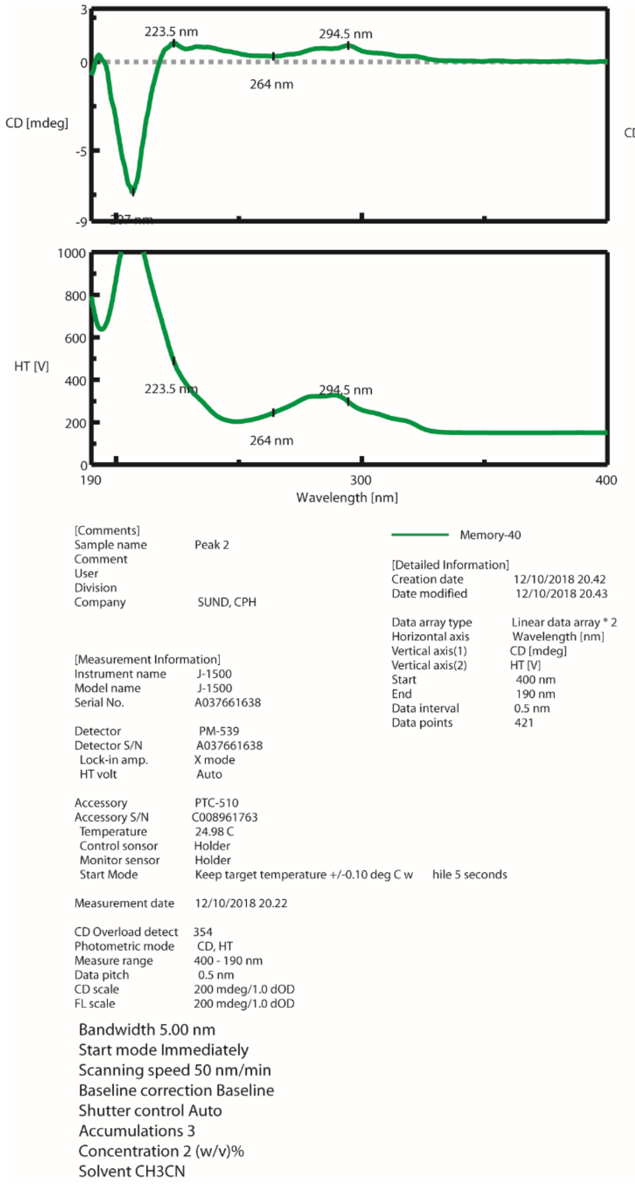

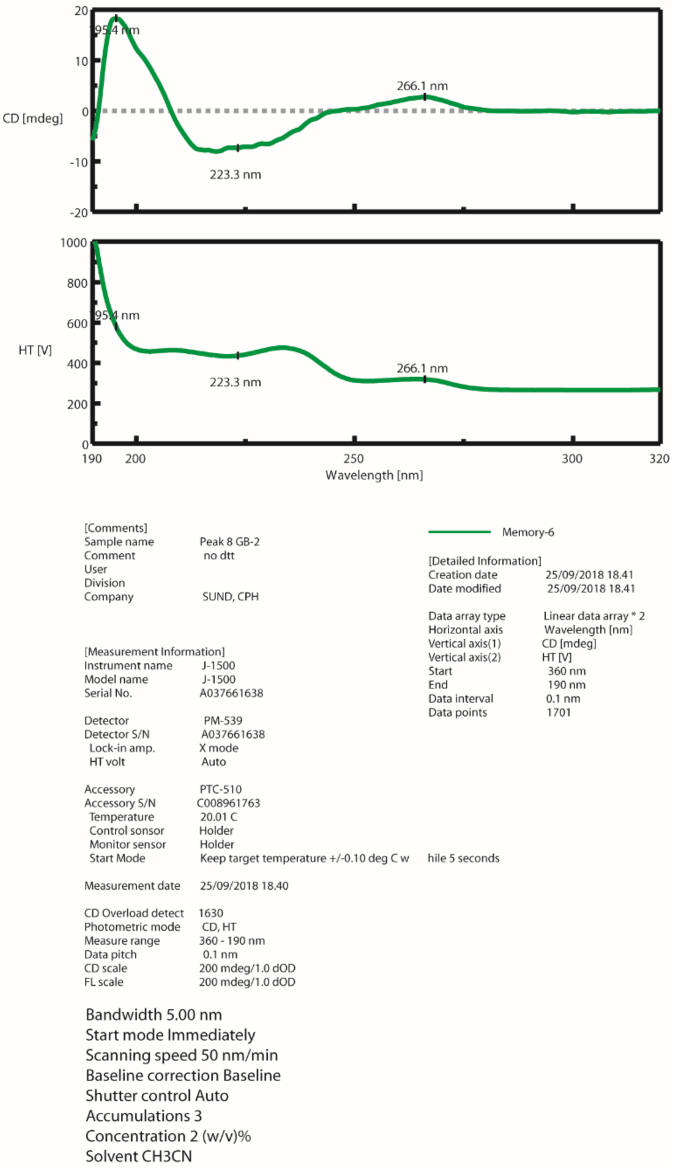

Figure S9. ECD spectra of **6a** and **6b**.

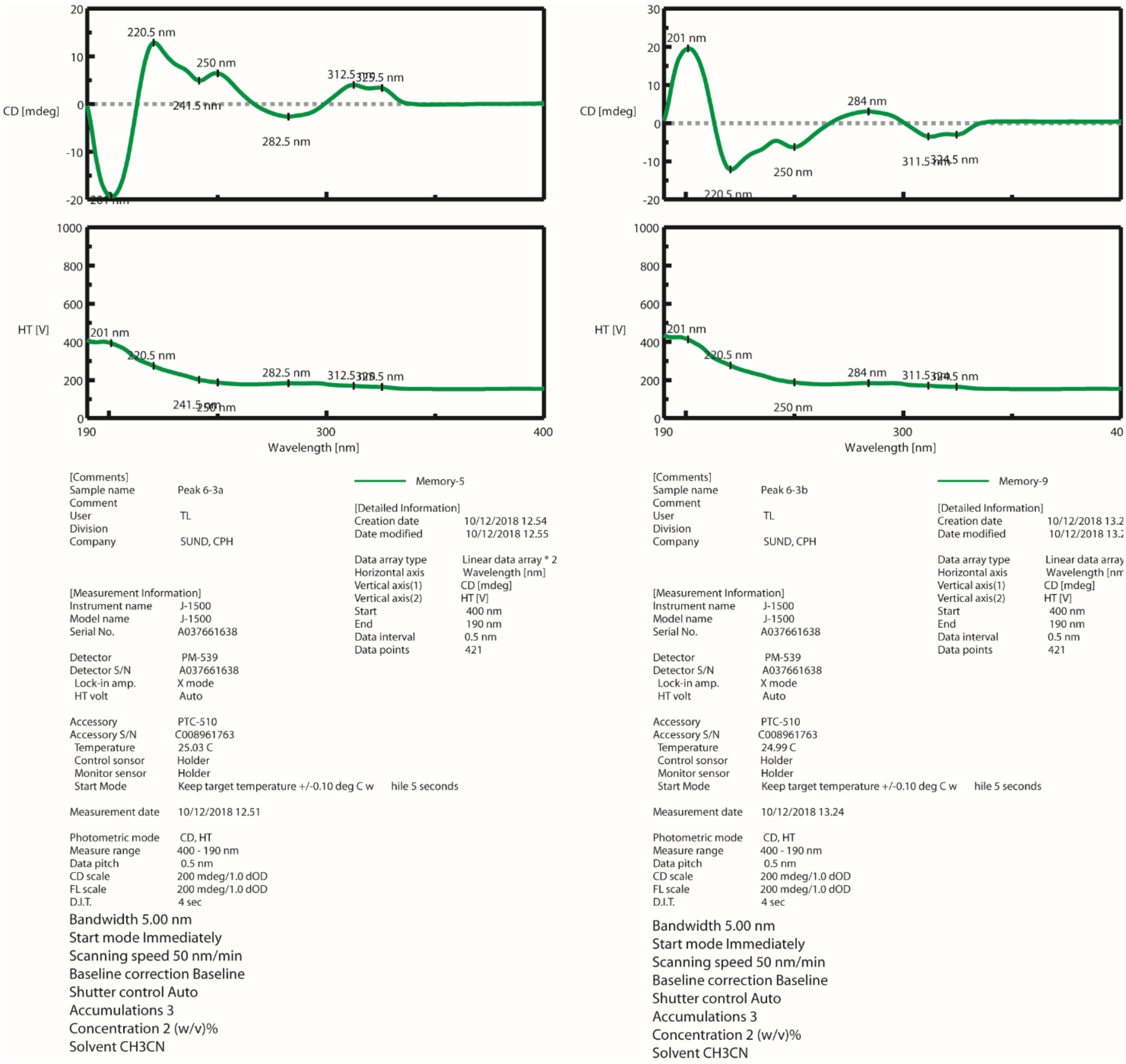

Figure S10. ECD spectra of **11a** and **11b**.

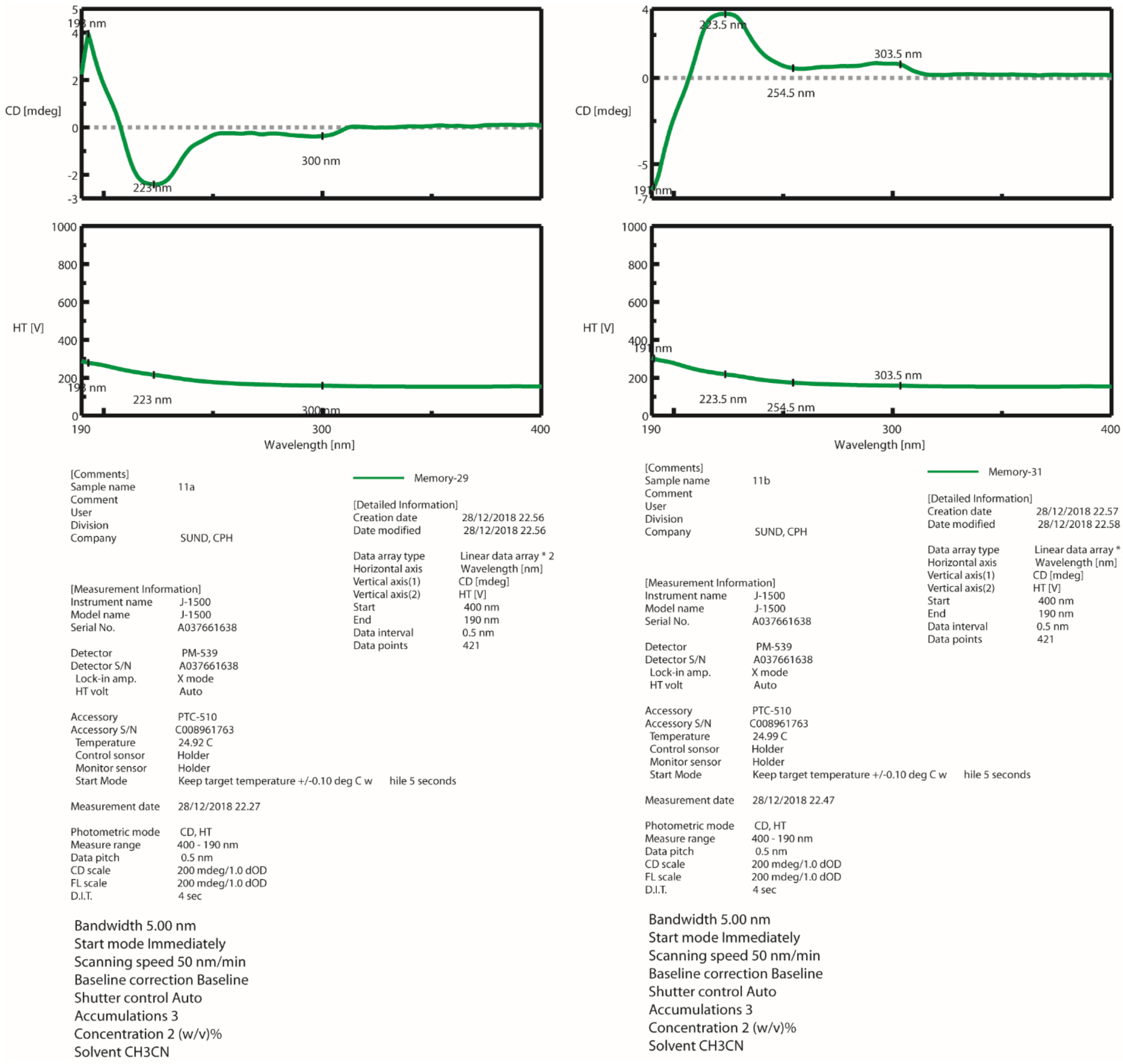

Figure S11. ECD spectra of **14a** and **14b**.

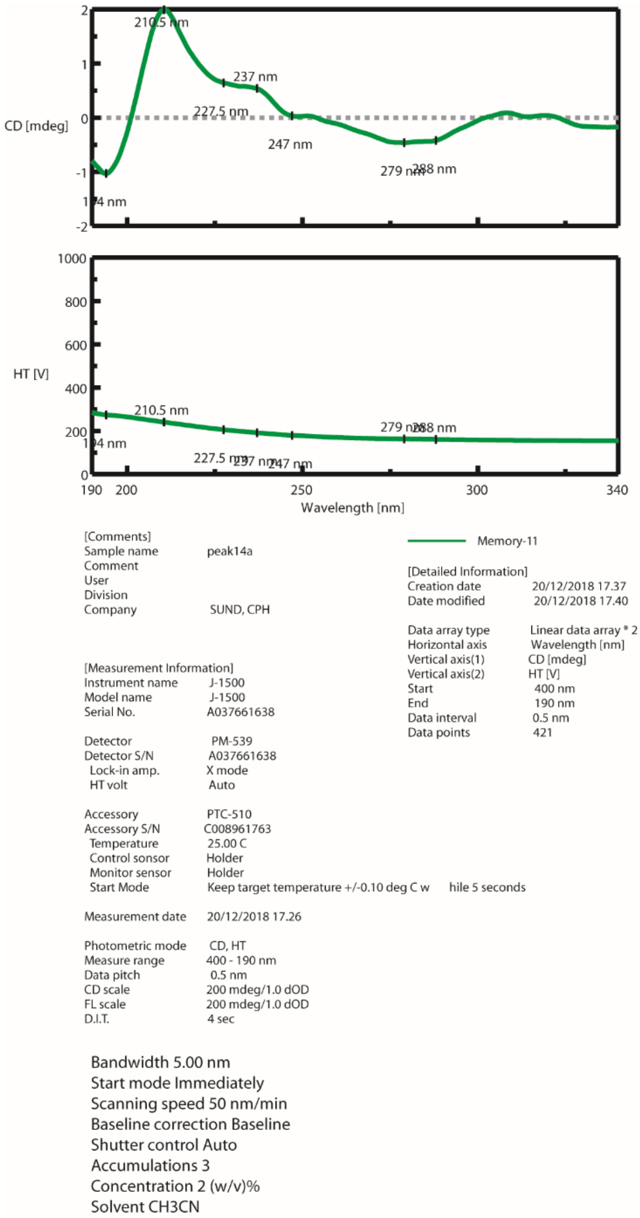

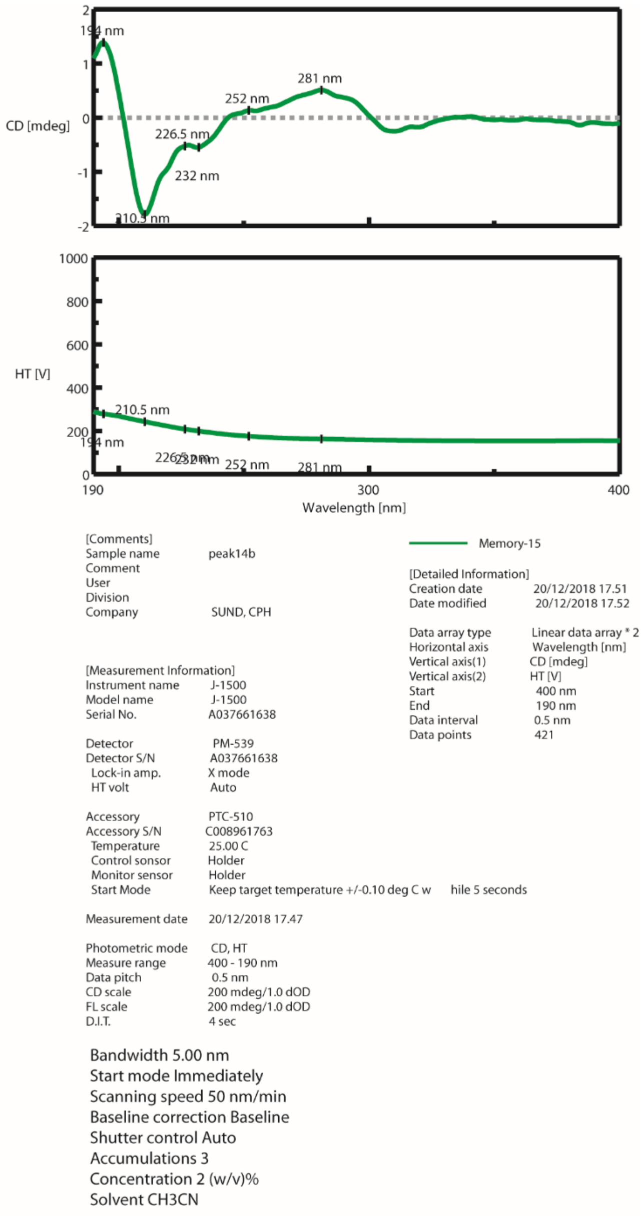

Figure S12. ECD spectra of **15a** and **15b**.

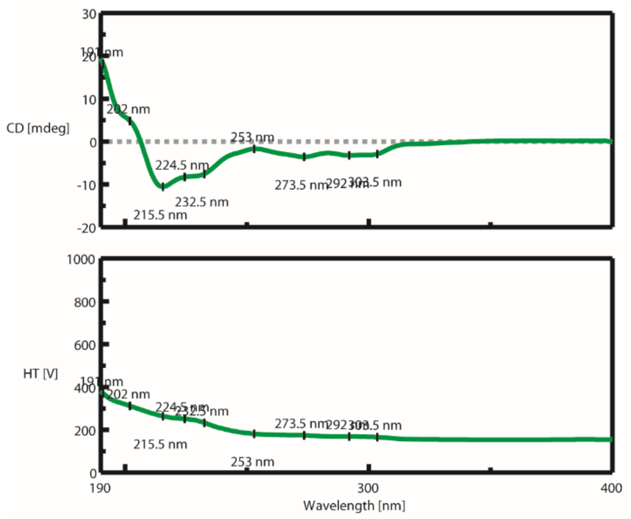

[Comments]
Sample name peak15a
Comment
User
Division
Company SUND, CPH

Memory-17

[Detailed Information]
Creation date 20/12/2018 18.08
Date modified 20/12/2018 18.09

Data array type Linear data array * 2
Horizontal axis Wavelength [nm]
Vertical axis(1) CD [mdeg]
Vertical axis(2) HT [V]
Start 400 nm
End 190 nm
Data interval 0.5 nm
Data points 421

[Measurement Information]
Instrument name J-1500
Model name J-1500
Serial No. A037661638

Detector PM-539
Detector S/N A037661638
Lock-in amp. X mode
HT volt Auto

Accessory PTC-510
Accessory S/N C008961763
Temperature 25.00 C
Control sonsor Holder
Monitor sensor Holder
Start Mode Keep target temperature +/-0.10 deg C w hile 5 seconds

Measurement date 20/12/2018 18.04

Photometric mode CD, HT
Measure range 400 - 190 nm
Data pitch 0.5 nm
CD scale 200 mdeg/1.0 dOD
FL scale 200 mdeg/1.0 dOD
D.I.T. 4 sec

Bandwidth 5.00 nm
Start mode Immediately
Scanning speed 50 nm/min
Baseline correction Baseline
Shutter control Auto
Accumulations 3
Concentration 2 (w/v)%
Solvent CH3CN

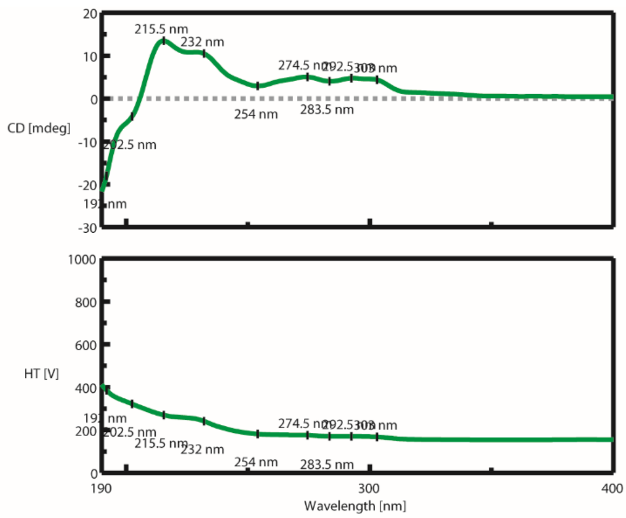

[Comments]
Sample name peak15b
Comment
User
Division
Company SUND, CPH

Memory-19

[Detailed Information]
Creation date 20/12/2018 18.46
Date modified 20/12/2018 18.47

Data array type Linear data array * 2
Horizontal axis Wavelength [nm]
Vertical axis(1) CD [mdeg]
Vertical axis(2) HT [V]
Start 400 nm
End 190 nm
Data interval 0.5 nm
Data points 421

[Measurement Information]
Instrument name J-1500
Model name J-1500
Serial No. A037661638

Detector PM-539
Detector S/N A037661638
Lock-in amp. X mode
HT volt Auto

Accessory PTC-510
Accessory S/N C008961763
Temperature 25.00 C
Control sonsor Holder
Monitor sensor Holder
Start Mode Keep target temperature +/-0.10 deg C w hile 5 seconds

Measurement date 20/12/2018 18.21

Photometric mode CD, HT
Measure range 400 - 190 nm
Data pitch 0.5 nm
CD scale 200 mdeg/1.0 dOD
FL scale 200 mdeg/1.0 dOD
D.I.T. 4 sec

Bandwidth 5.00 nm
Start mode Immediately
Scanning speed 50 nm/min
Baseline correction Baseline
Shutter control Auto
Accumulations 3
Concentration 2 (w/v)%
Solvent CH3CN

Figure S13. ECD spectra of **17a** and **17b**.

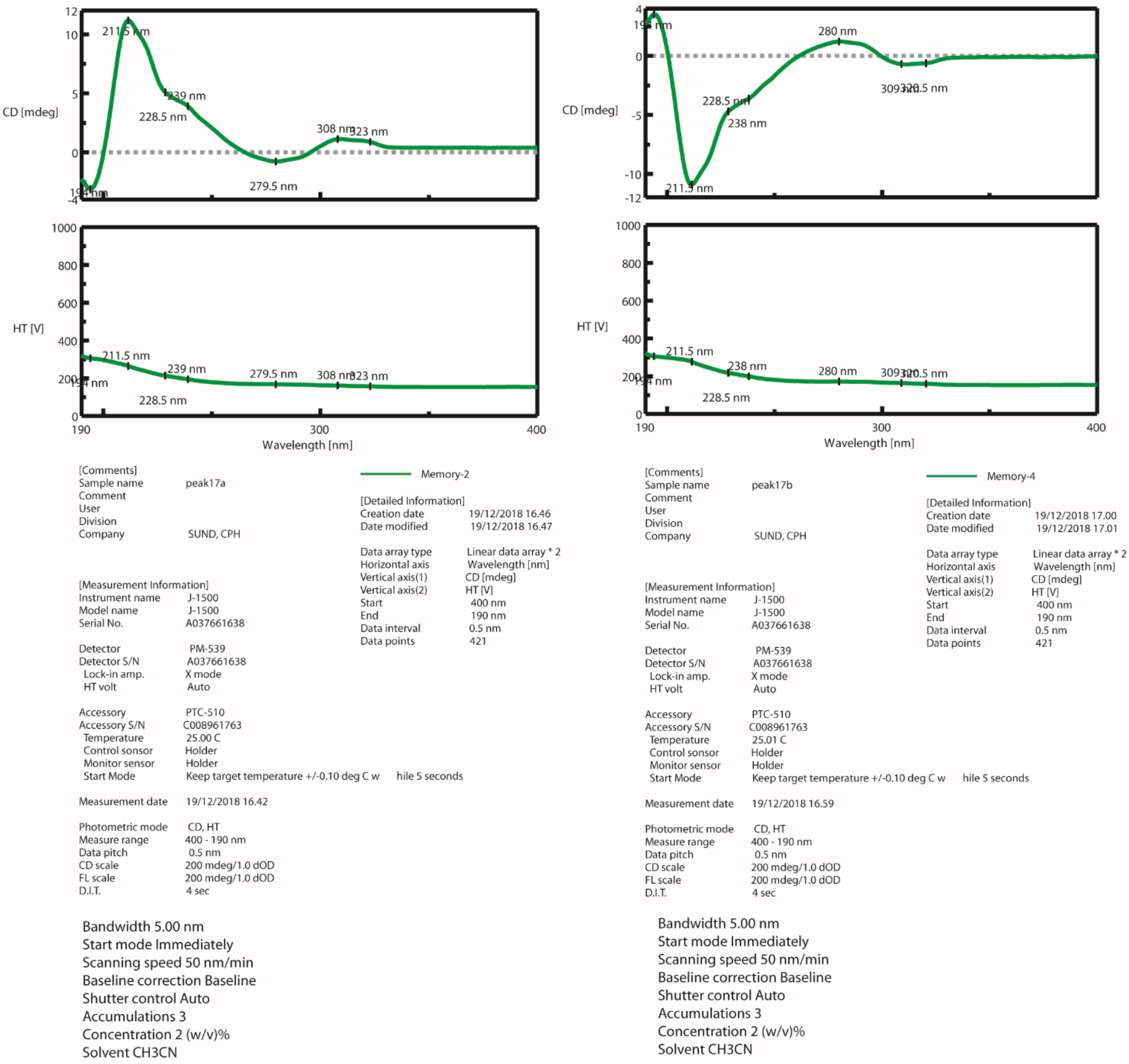

Figure S14. ECD spectra of **18a** and **18b**.

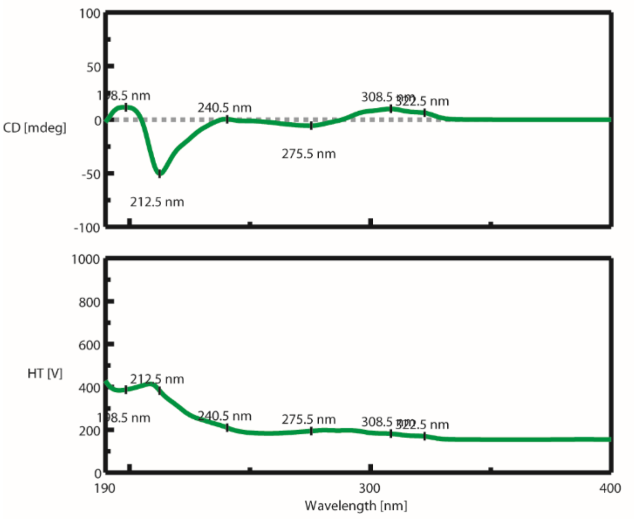

[Comments]
Sample name peak18a
Comment
User
Division
Company SUND, CPH

Memory-18

[Detailed Information]
Creation date 17/12/2018 20.24
Date modified 17/12/2018 20.25

Data array type Linear data array * 2
Horizontal axis Wavelength [nm]
Vertical axis(1) CD [mdeg]
Vertical axis(2) HT [V]
Start 400 nm
End 190 nm
Data interval 0.5 nm
Data points 421

[Measurement Information]
Instrument name J-1500
Model name J-1500
Serial No. A037661638

Detector PM-539
Detector S/N A037661638
Lock-in amp. X mode
HT volt Auto

Accessory PTC-510
Accessory S/N C008961763
Temperature 24.99 C
Control sonsor Holder
Monitor sensor Holder
Start Mode Keep target temperature +/-0.10 deg C w hile 5 seconds

Measurement date 17/12/2018 17.35

Photometric mode CD, HT
Measure range 400 - 190 nm
Data pitch 0.5 nm
CD scale 200 mdeg/1.0 dOD
FL scale 200 mdeg/1.0 dOD
D.I.T. 4 sec

Bandwidth 5.00 nm
Start mode Immediately
Scanning speed 50 nm/min
Baseline correction Baseline
Shutter control Auto
Accumulations 3
Concentration 2 (w/v)%
Solvent CH3CN

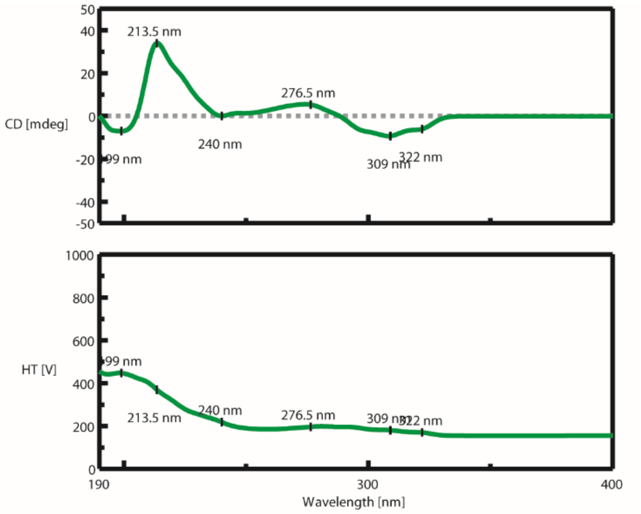

[Comments]
Sample name peak18b
Comment
User
Division
Company SUND, CPH

Memory-19

[Detailed Information]
Creation date 17/12/2018 20.26
Date modified 17/12/2018 20.26

Data array type Linear data array *
Horizontal axis Wavelength [nm]
Vertical axis(1) CD [mdeg]
Vertical axis(2) HT [V]
Start 400 nm
End 190 nm
Data interval 0.5 nm
Data points 421

[Measurement Information]
Instrument name J-1500
Model name J-1500
Serial No. A037661638

Detector PM-539
Detector S/N A037661638
Lock-in amp. X mode
HT volt Auto

Accessory PTC-510
Accessory S/N C008961763
Temperature 24.99 C
Control sonsor Holder
Monitor sensor Holder
Start Mode Keep target temperature +/-0.10 deg C w hile 5 seconds

Measurement date 17/12/2018 17.53

Photometric mode CD, HT
Measure range 400 - 190 nm
Data pitch 0.5 nm
CD scale 200 mdeg/1.0 dOD
FL scale 200 mdeg/1.0 dOD
D.I.T. 4 sec

Bandwidth 5.00 nm
Start mode Immediately
Scanning speed 50 nm/min
Baseline correction Baseline
Shutter control Auto
Accumulations 3
Concentration 2 (w/v)%
Solvent CH3CN

Figure S15. ECD spectra of **19a** and **19b**.

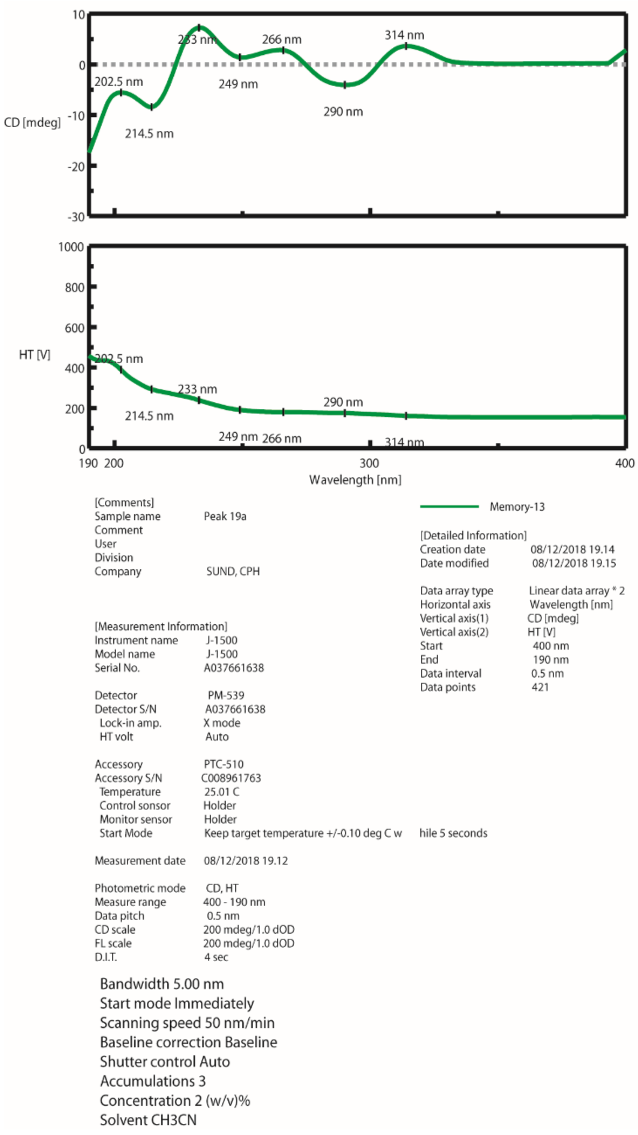

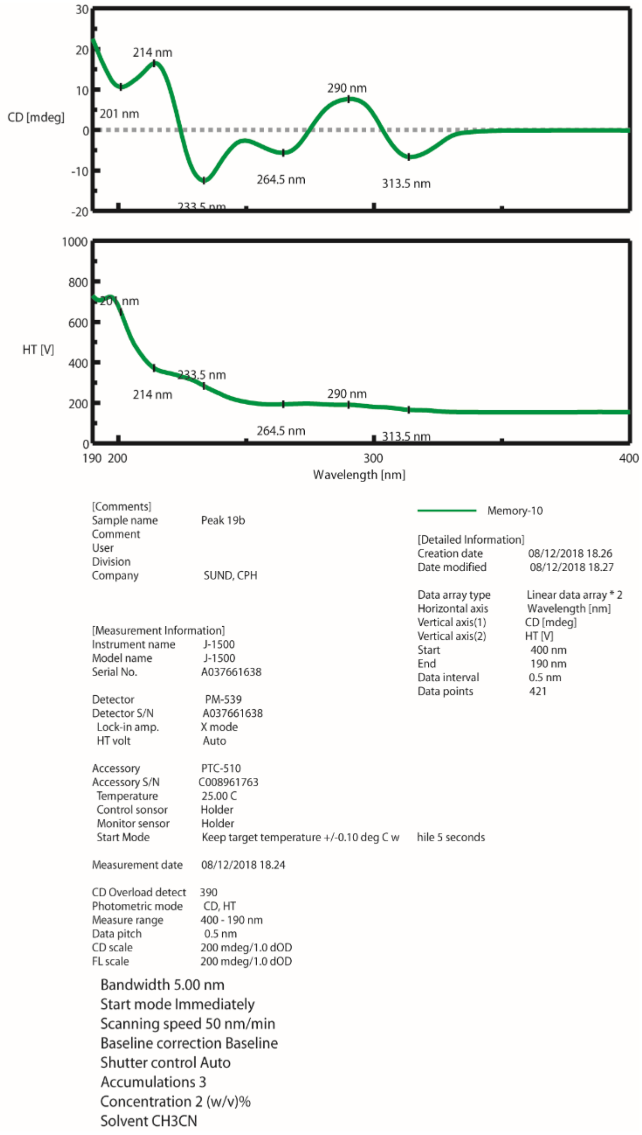

Figure S16. ECD spectra of **23a** and **23b**.

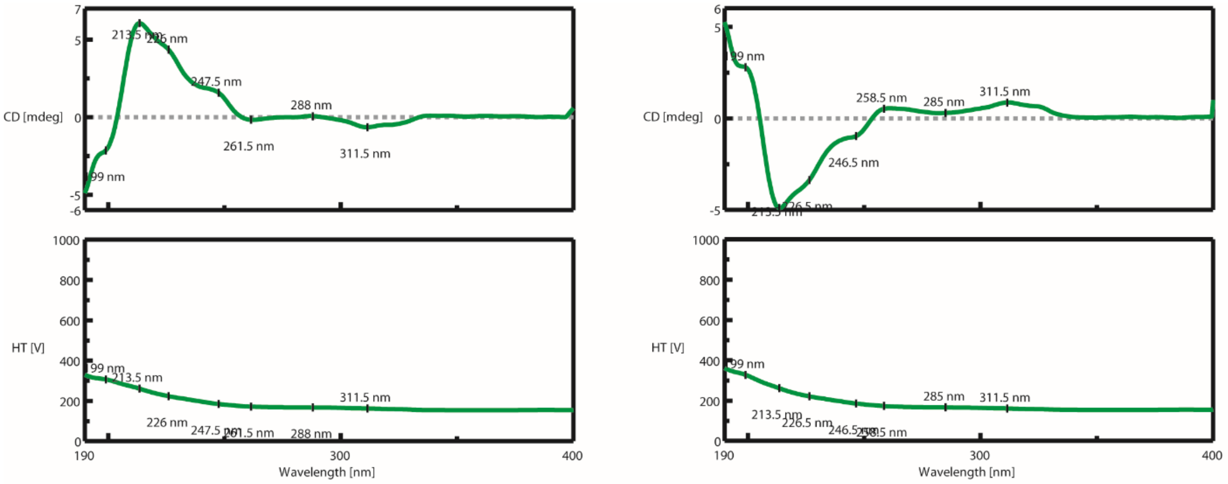

[Comments]
Sample name peak23-a
Comment
User
Division
Company SUND, CPH

Memory-15

[Detailed Information]
Creation date 17/12/2018 20.21
Date modified 17/12/2018 20.22

Data array type Linear data array * 2
Horizontal axis Wavelength [nm]
Vertical axis(1) CD [mdeg]
Vertical axis(2) HT [V]
Start 400 nm
End 190 nm
Data interval 0.5 nm
Data points 421

[Measurement Information]
Instrument name J-1500
Model name J-1500
Serial No. A037661638

Detector PM-539
Detector S/N A037661638
Lock-in amp. X mode
HT volt Auto

Accessory PTC-510
Accessory S/N C008961763
Temperature 25.00 C
Control sonsor Holder
Monitor sensor Holder
Start Mode Keep target temperature +/-0.10 deg C w hile 5 seconds

Measurement date 17/12/2018 16.59

Photometric mode CD, HT
Measure range 400 - 190 nm
Data pitch 0.5 nm
CD scale 200 mdeg/1.0 dOD
FL scale 200 mdeg/1.0 dOD
D.I.T. 4 sec

Bandwidth 5.00 nm
Start mode Immediately
Scanning speed 50 nm/min
Baseline correction Baseline
Shutter control Auto
Accumulations 3
Concentration 2 (w/v)%
Solvent CH3CN

[Comments]
Sample name peak23-b
Comment
User
Division
Company SUND, CPH

Memory-17

[Detailed Information]
Creation date 17/12/2018 20.23
Date modified 17/12/2018 20.24

Data array type Linear data array * 2
Horizontal axis Wavelength [nm]
Vertical axis(1) CD [mdeg]
Vertical axis(2) HT [V]
Start 400 nm
End 190 nm
Data interval 0.5 nm
Data points 421

[Measurement Information]
Instrument name J-1500
Model name J-1500
Serial No. A037661638

Detector PM-539
Detector S/N A037661638
Lock-in amp. X mode
HT volt Auto

Accessory PTC-510
Accessory S/N C008961763
Temperature 24.99 C
Control sonsor Holder
Monitor sensor Holder
Start Mode Keep target temperature +/-0.10 deg C w hile 5 seconds

Measurement date 17/12/2018 17.17

Photometric mode CD, HT
Measure range 400 - 190 nm
Data pitch 0.5 nm
CD scale 200 mdeg/1.0 dOD
FL scale 200 mdeg/1.0 dOD
D.I.T. 4 sec

Bandwidth 5.00 nm
Start mode Immediately
Scanning speed 50 nm/min
Baseline correction Baseline
Shutter control Auto
Accumulations 3
Concentration 2 (w/v)%
Solvent CH3CN

Figure S17. ECD spectra of **24a** and **24b**.

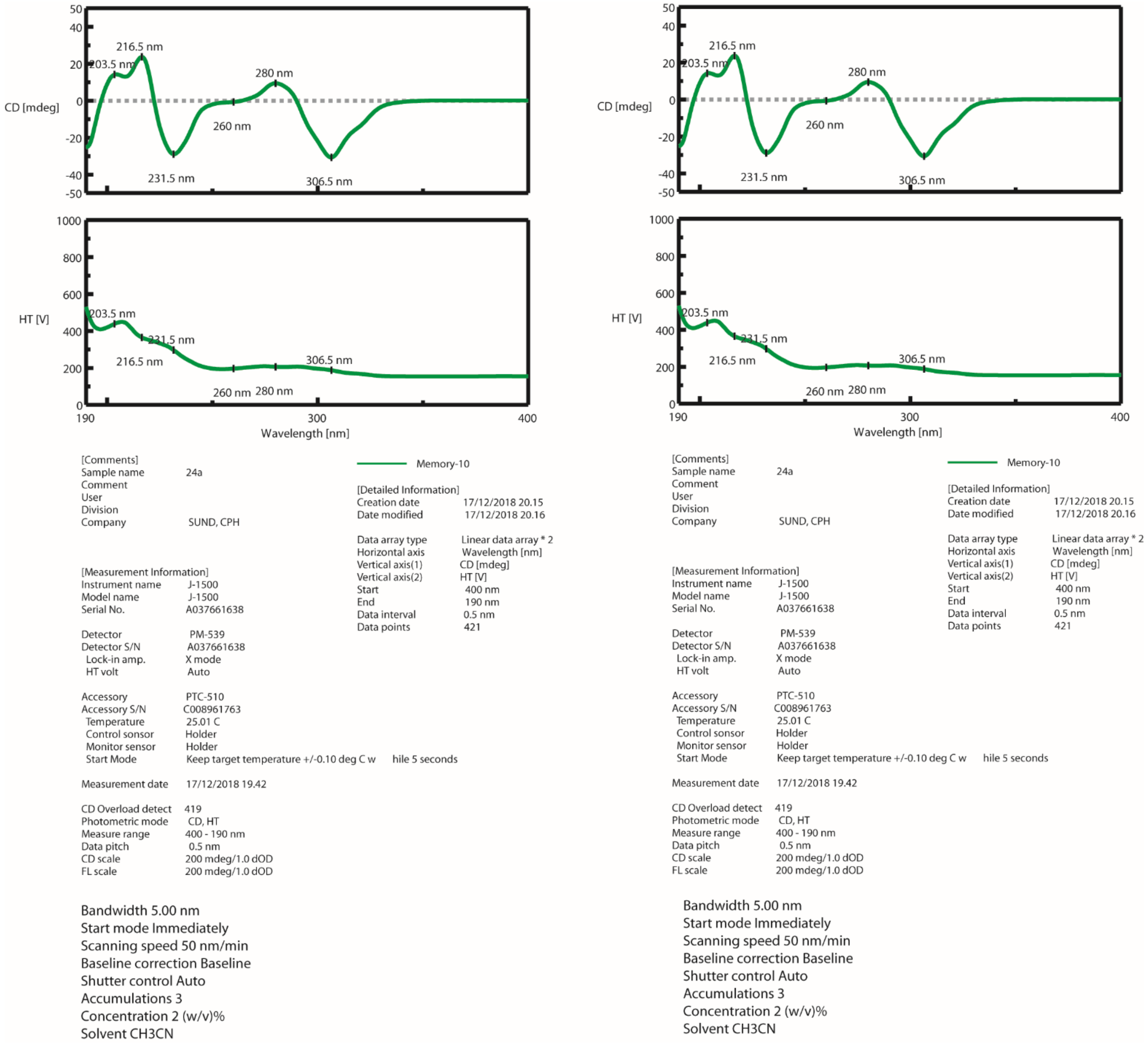

Figure S18. ECD spectra of **25a** and **25b**.

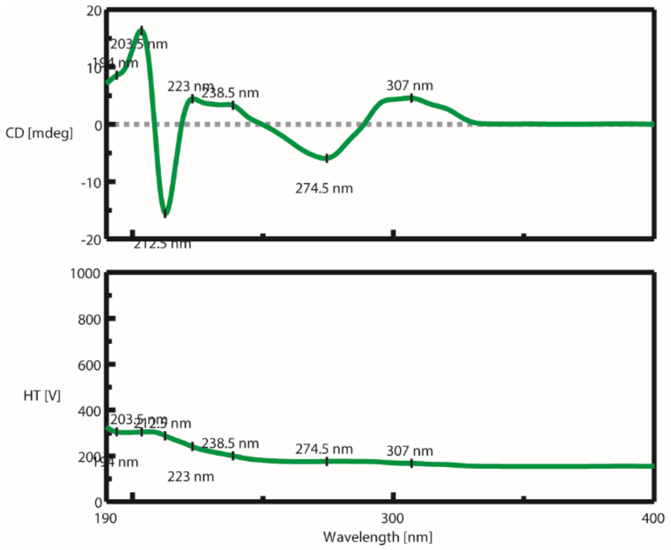

Memory-12

[Comments]
Sample name 25a
Comment
User
Division
Company SUND, CPH

[Detailed Information]
Creation date 17/12/2018 20.18
Date modified 17/12/2018 20.19

Data array type Linear data array * 2
Horizontal axis Wavelength [nm]
Vertical axis(1) CD [mdeg]
Vertical axis(2) HT [V]
Start 400 nm
End 190 nm
Data interval 0.5 nm
Data points 421

[Measurement Information]
Instrument name J-1500
Model name J-1500
Serial No. A037661638

Detector PM-539
Detector S/N A037661638
Lock-in amp. X mode
HT volt Auto

Accessory PTC-510
Accessory S/N C008961763
Temperature 25.01 C
Control sensor Holder
Monitor sensor Holder
Start Mode Keep target temperature +/-0.10 deg C w hile 5 seconds

Measurement date 17/12/2018 19.07

Photometric mode CD, HT
Measure range 400 - 190 nm
Data pitch 0.5 nm
CD scale 200 mdeg/1.0 dOD
FL scale 200 mdeg/1.0 dOD
D.I.T. 4 sec

Bandwidth 5.00 nm
Start mode Immediately
Scanning speed 50 nm/min
Baseline correction Baseline
Shutter control Auto
Accumulations 3
Concentration 2 (w/v)%
Solvent CH3CN

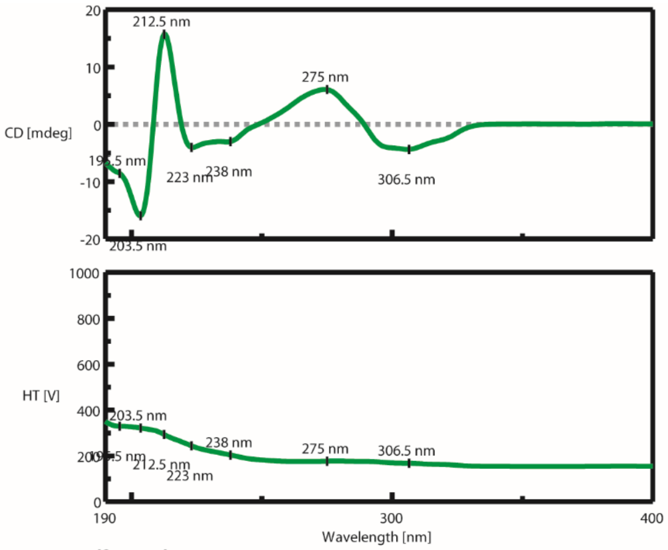

Memory-13

[Comments]
Sample name 25b
Comment
User
Division
Company SUND, CPH

[Detailed Information]
Creation date 17/12/2018 20.19
Date modified 17/12/2018 20.20

Data array type Linear data array * 2
Horizontal axis Wavelength [nm]
Vertical axis(1) CD [mdeg]
Vertical axis(2) HT [V]
Start 400 nm
End 190 nm
Data interval 0.5 nm
Data points 421

[Measurement Information]
Instrument name J-1500
Model name J-1500
Serial No. A037661638

Detector PM-539
Detector S/N A037661638
Lock-in amp. X mode
HT volt Auto

Accessory PTC-510
Accessory S/N C008961763
Temperature 25.01 C
Control sonsor Holder
Monitor sensor Holder
Start Mode Keep target temperature +/-0.10 deg C w hile 5 seconds

Measurement date 17/12/2018 19.24

Photometric mode CD, HT
Measure range 400 - 190 nm
Data pitch 0.5 nm
CD scale 200 mdeg/1.0 dOD
FL scale 200 mdeg/1.0 dOD
D.I.T. 4 sec

Bandwidth 5.00 nm
Start mode Immediately
Scanning speed 50 nm/min
Baseline correction Baseline
Shutter control Auto
Accumulations 3
Concentration 2 (w/v)%
Solvent CH3CN

Figure S19. UV spectra obtained from HPLC-PDA-HRMS analysis of new compounds identified in *G. piloselloides*.

2	5	6
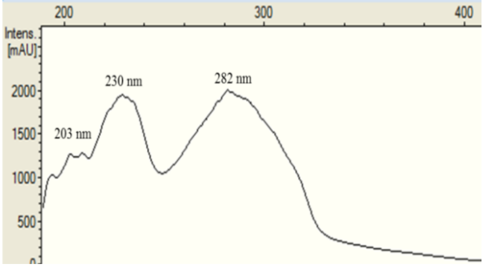	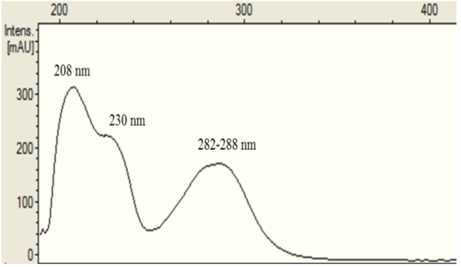	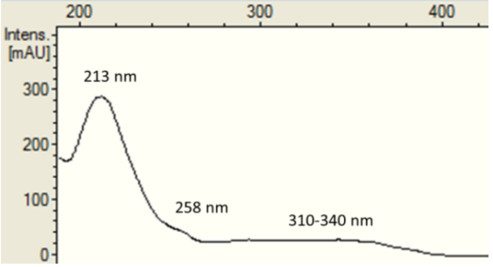

8	10	11
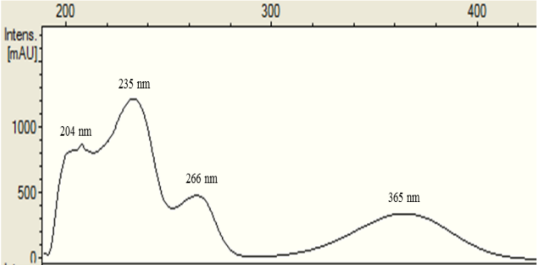	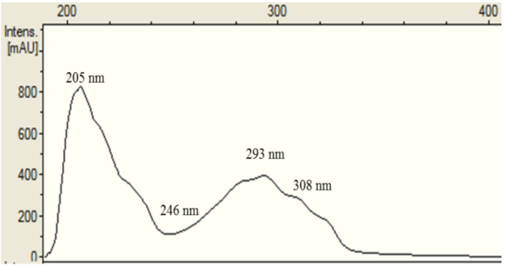	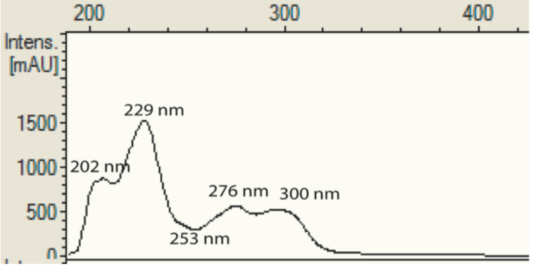

12	14	15
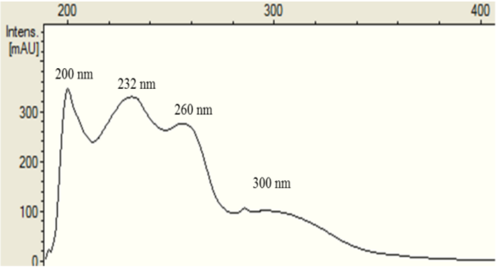	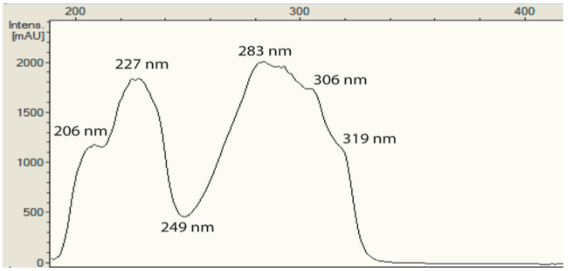	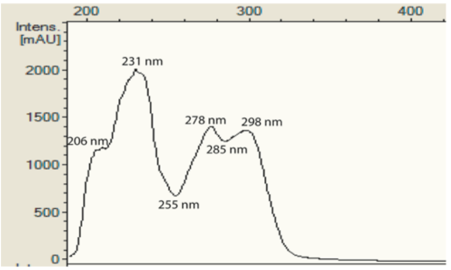

17

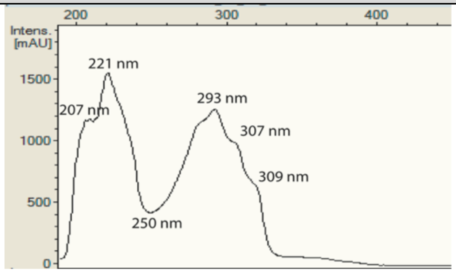

18

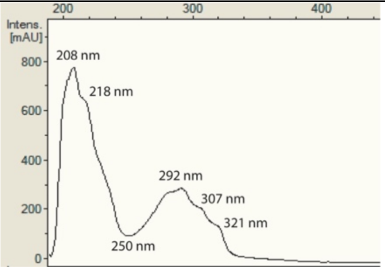

19

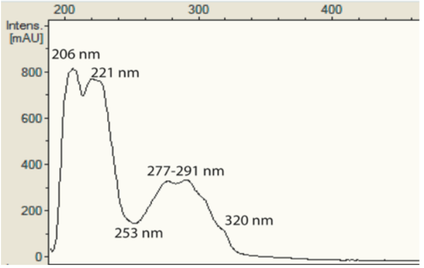

23

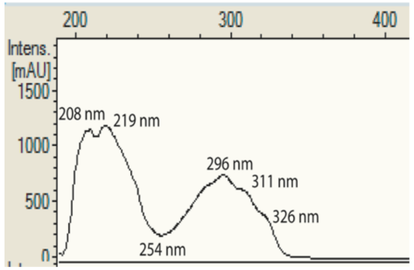

24

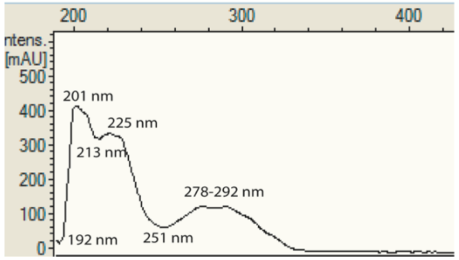

25

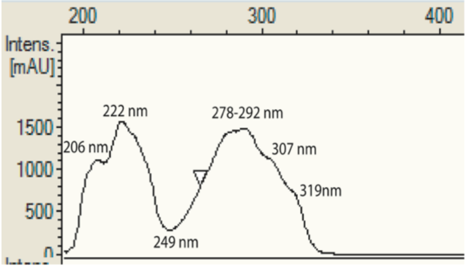

Figure S20. ^{1}H NMR spectrum of **1** (600 MHz, methanol-d_4).

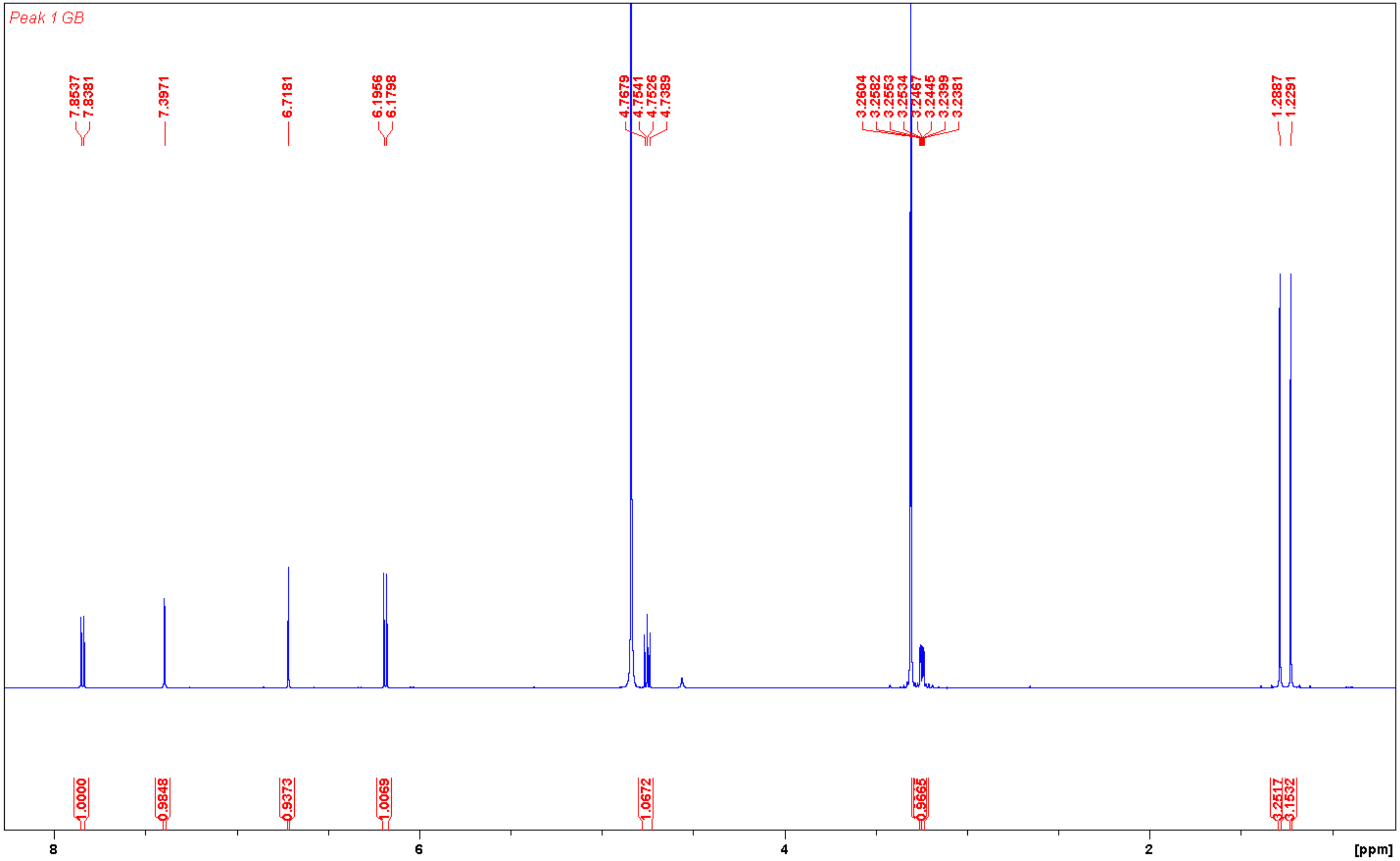

Figure S21. ^{1}H NMR spectrum of **2** (600 MHz, methanol-d_4).

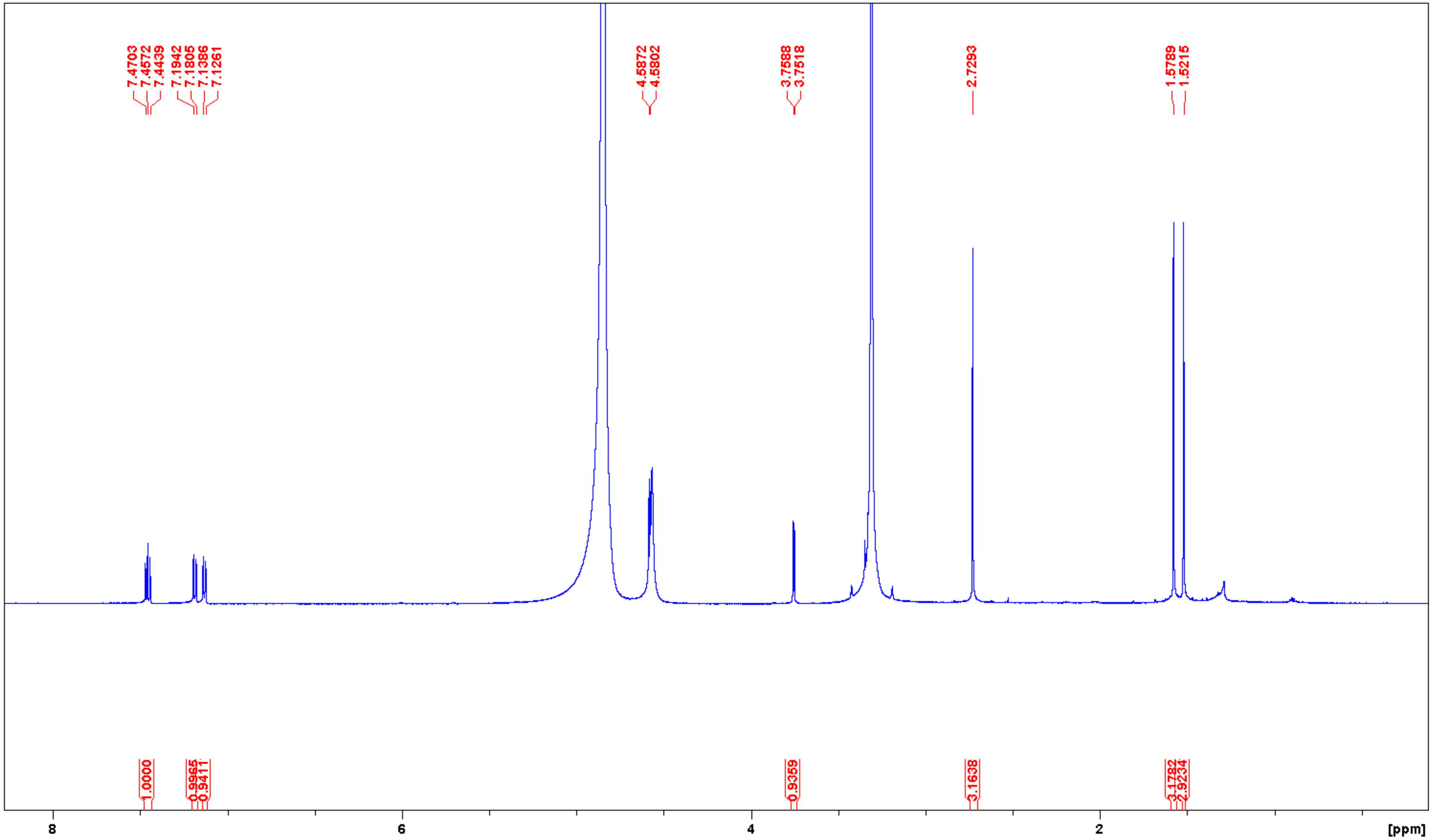

Figure S22. ^{13}C NMR spectrum of **2** (151 MHz, methanol-d_4).

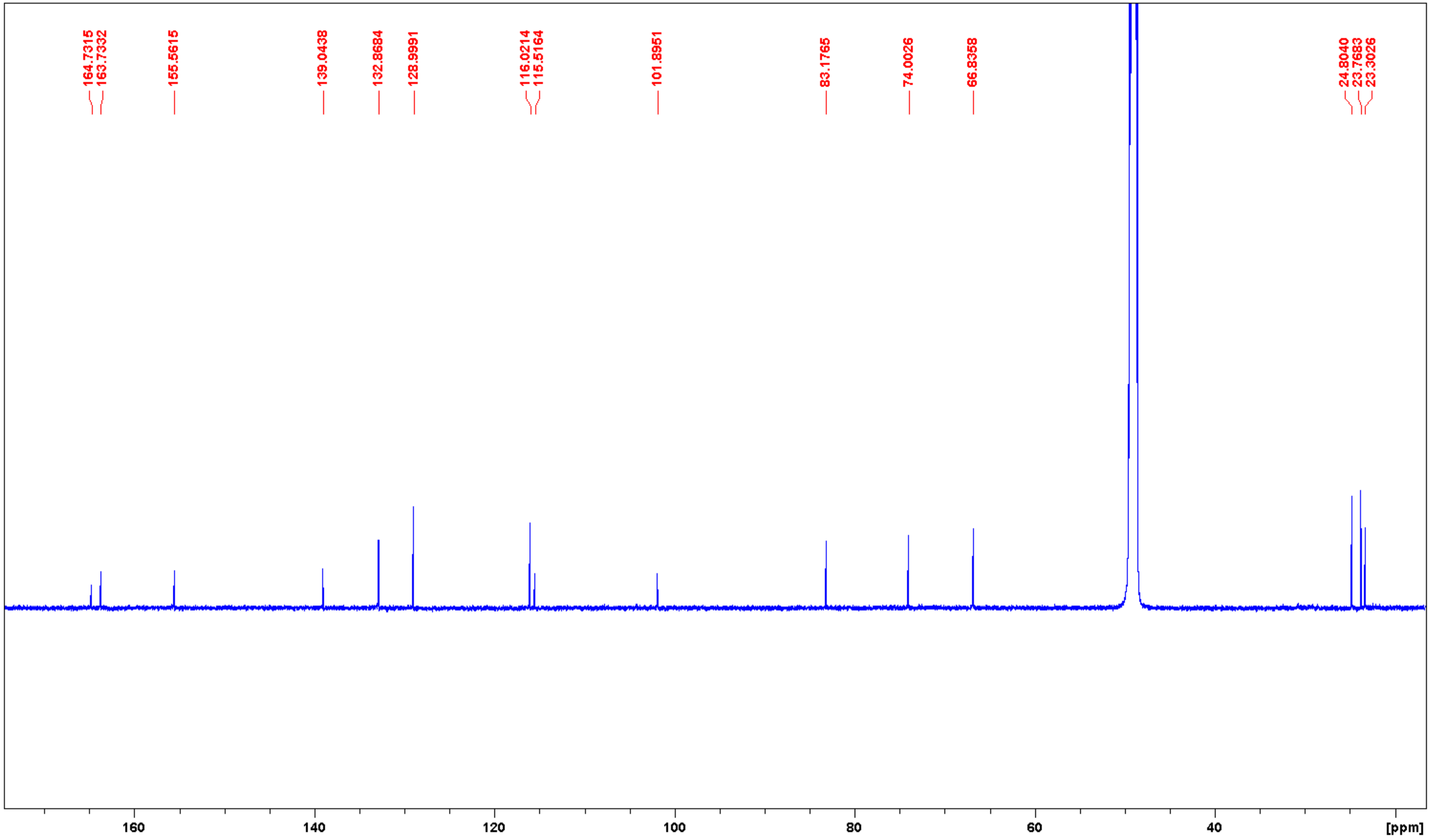

Figure S23. HSQC spectrum of **2** (600 MHz, methanol-d_4).

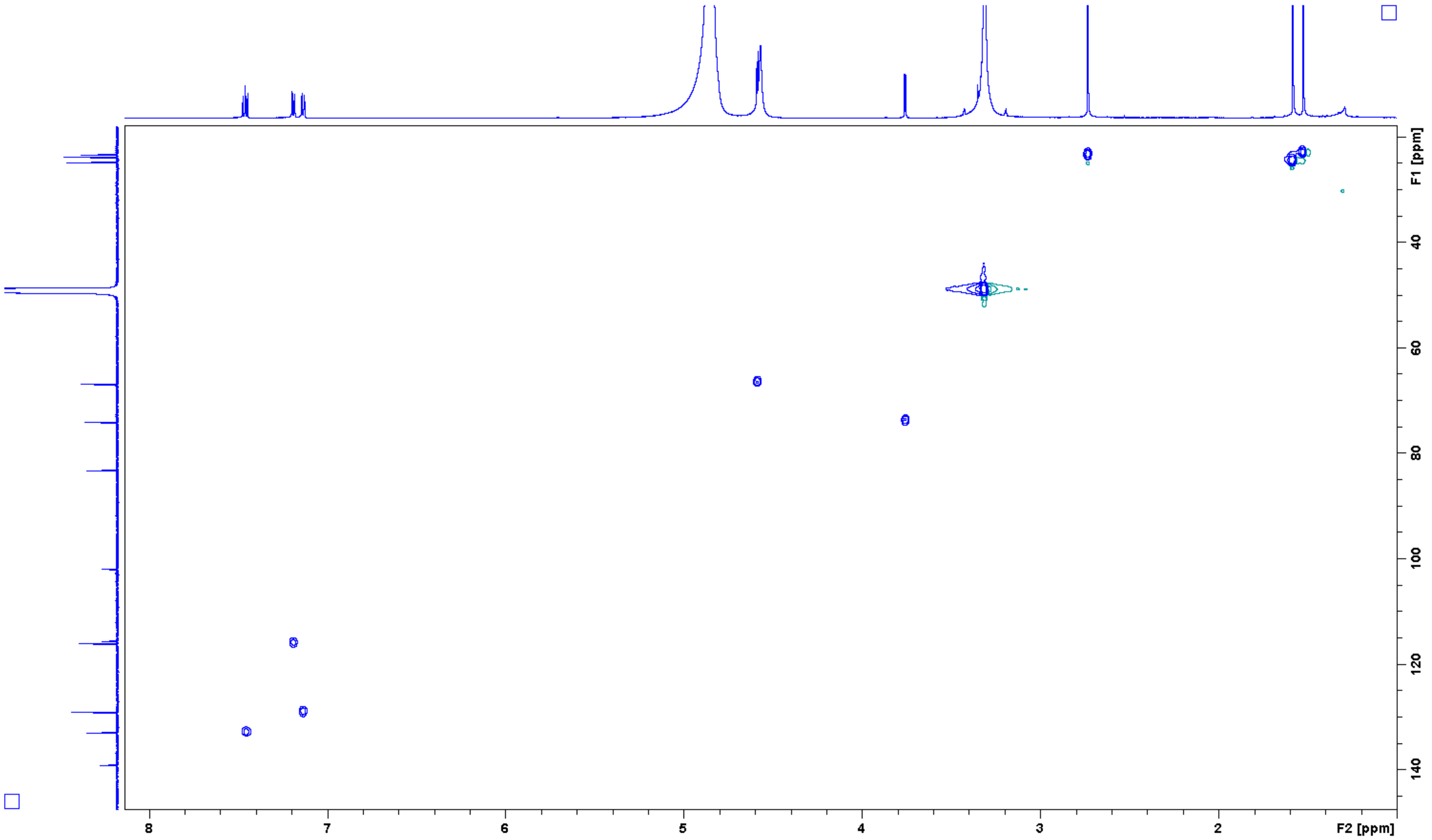

Figure S24. COSY spectrum of **2** (600 MHz, methanol-d_4)).

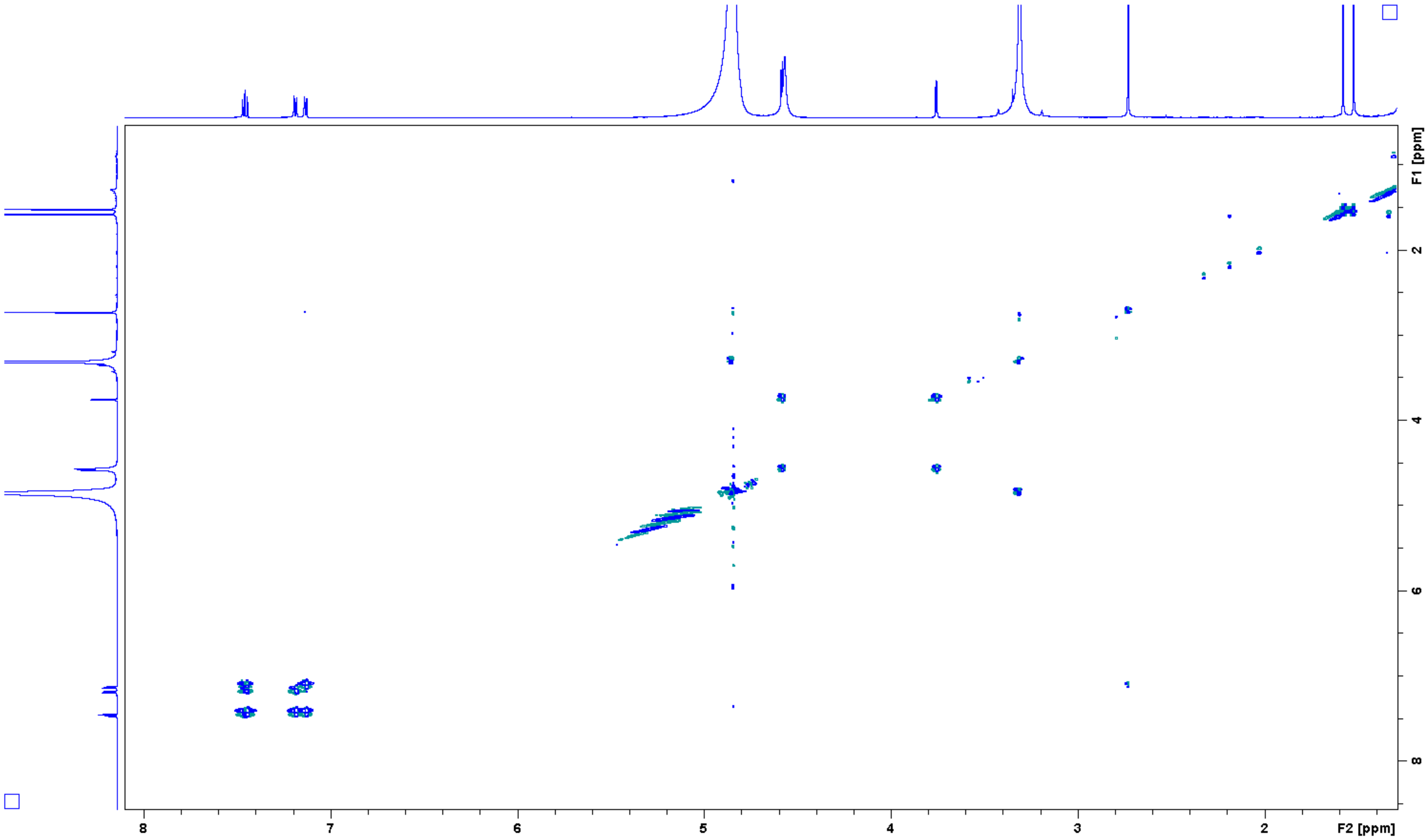

Figure S25. HMBC spectrum of **2** (600 MHz, methanol-d_4).

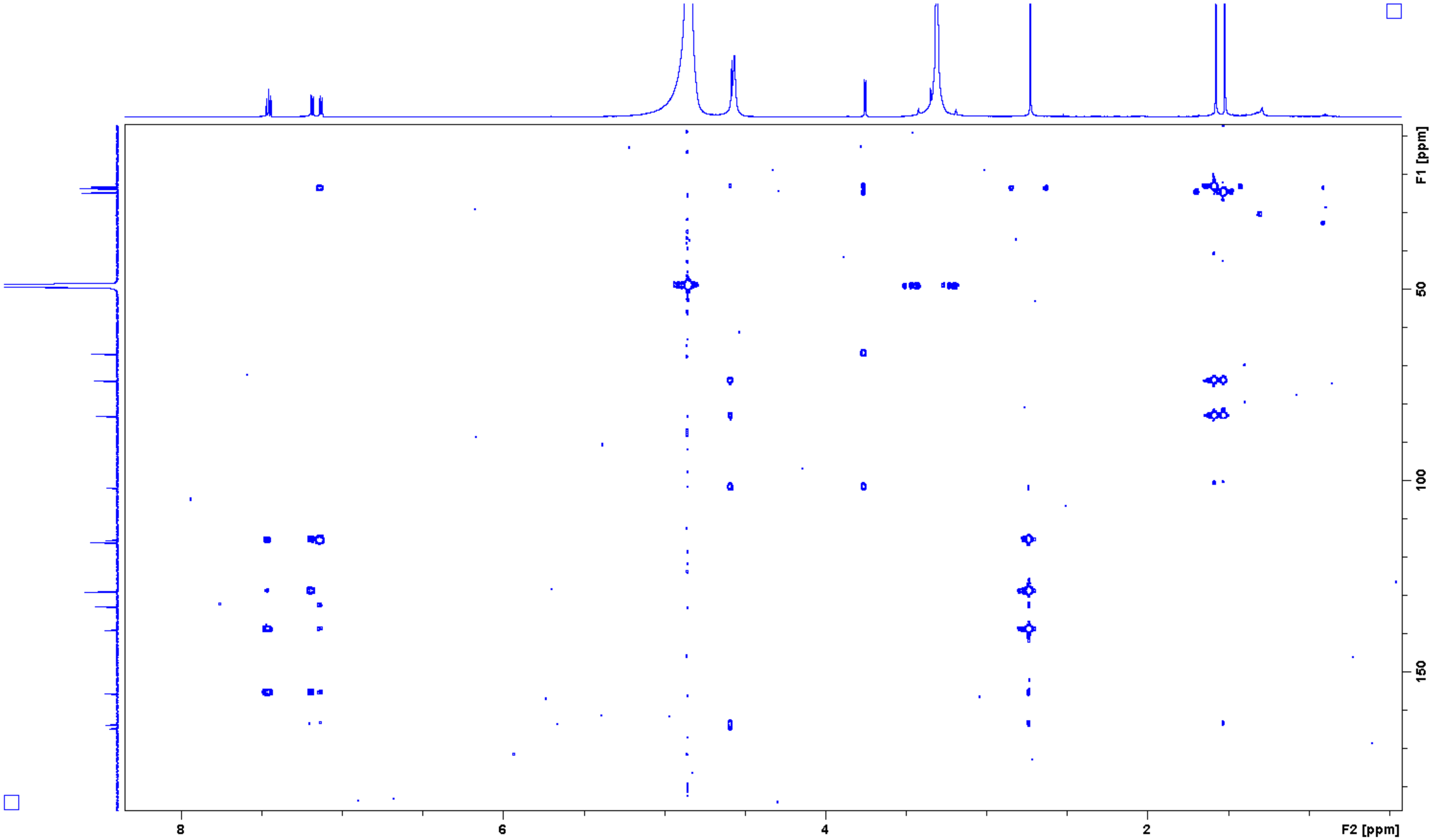

Figure S26. ROESY spectrum of **2** (600 MHz, methanol-d_4).

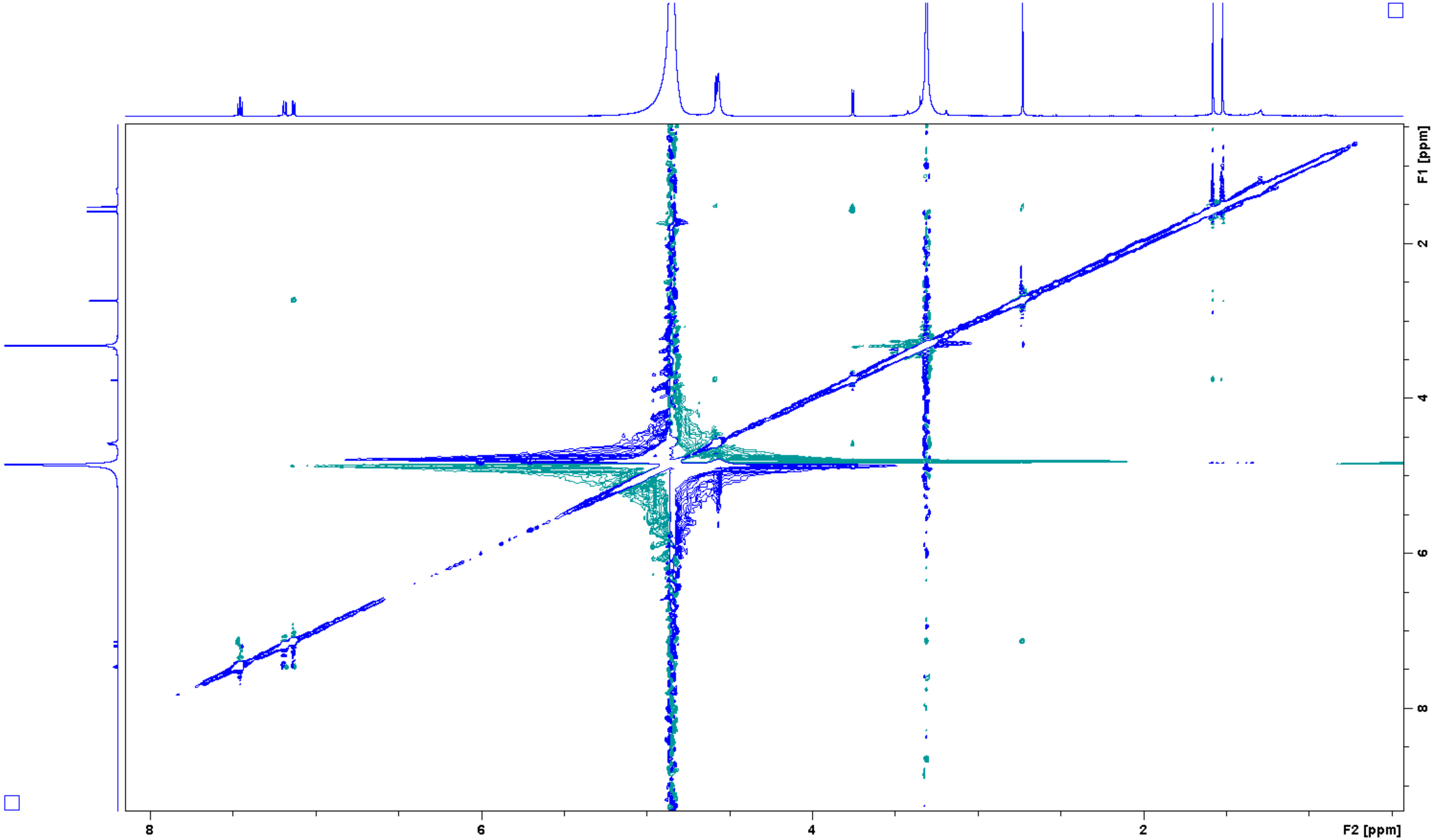

Figure S27. ^{1}H NMR spectrum of **3** (600 MHz, methanol-d_4).

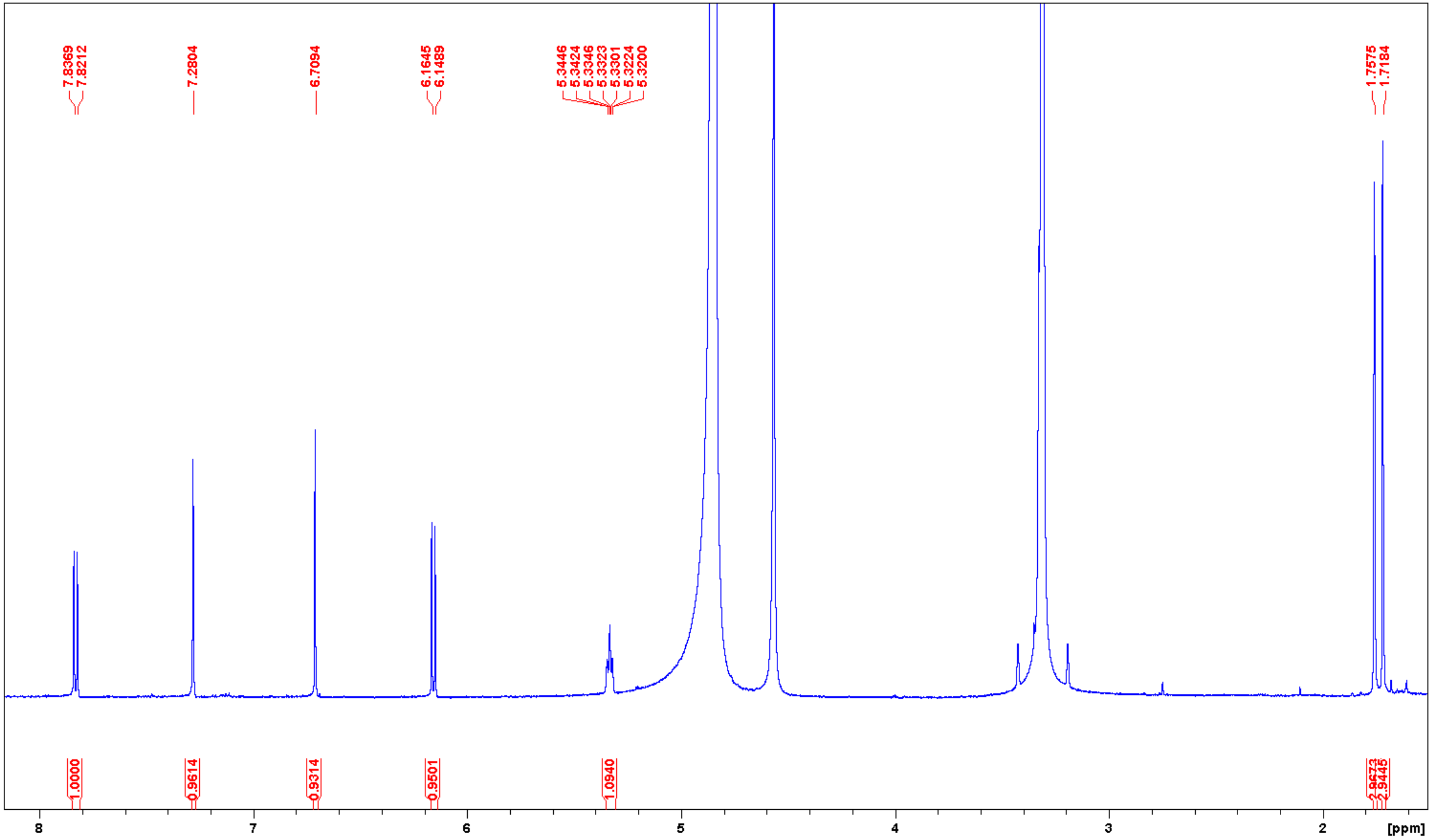

Figure S28. COSY spectrum of **3** (600 MHz, methanol-d_4).

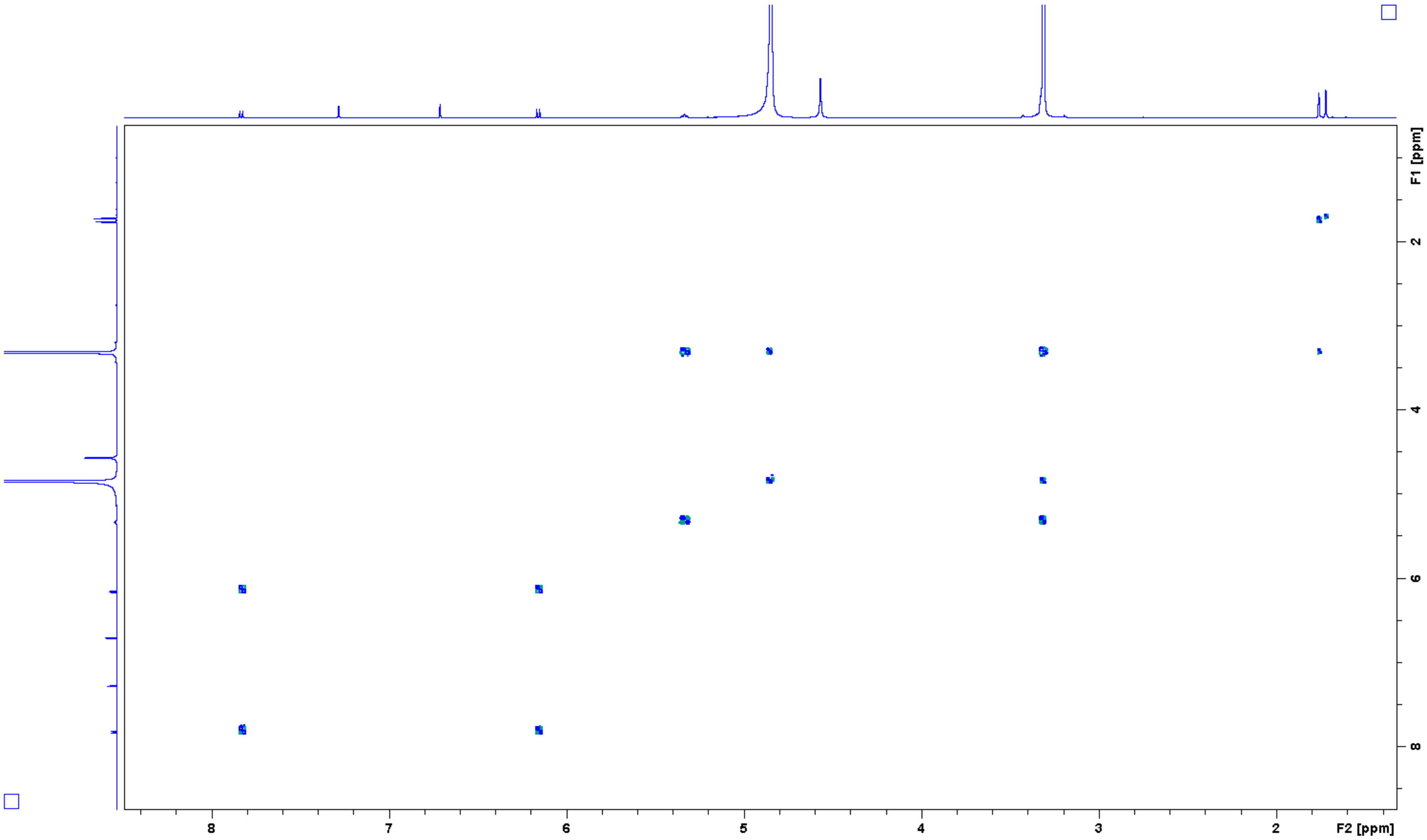

Figure S29. HSQC spectrum of **3** (600 MHz, methanol-d_4).

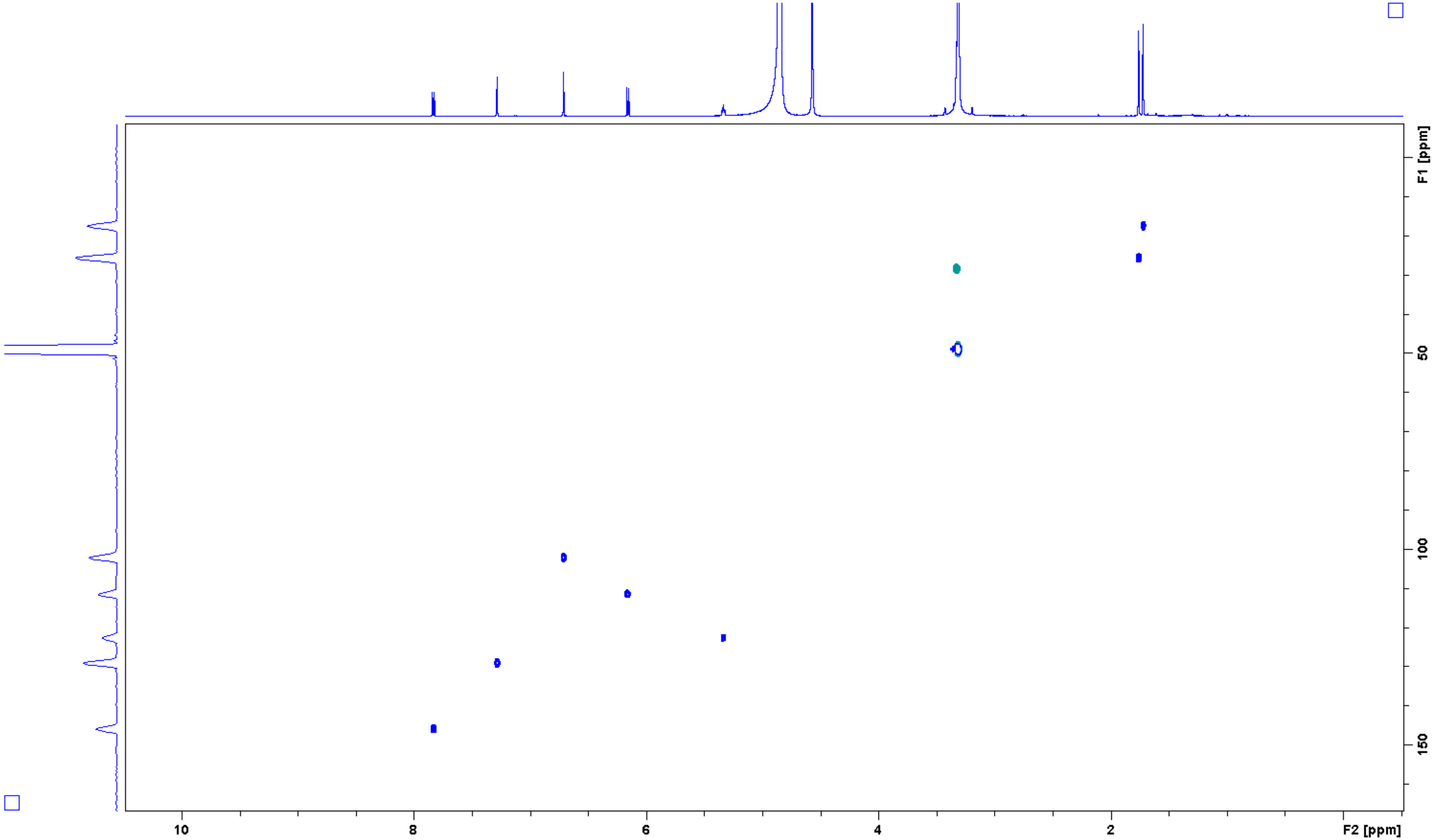

Figure S30. ^{1}H NMR spectrum of **4** (600 MHz, methanol-d_4).

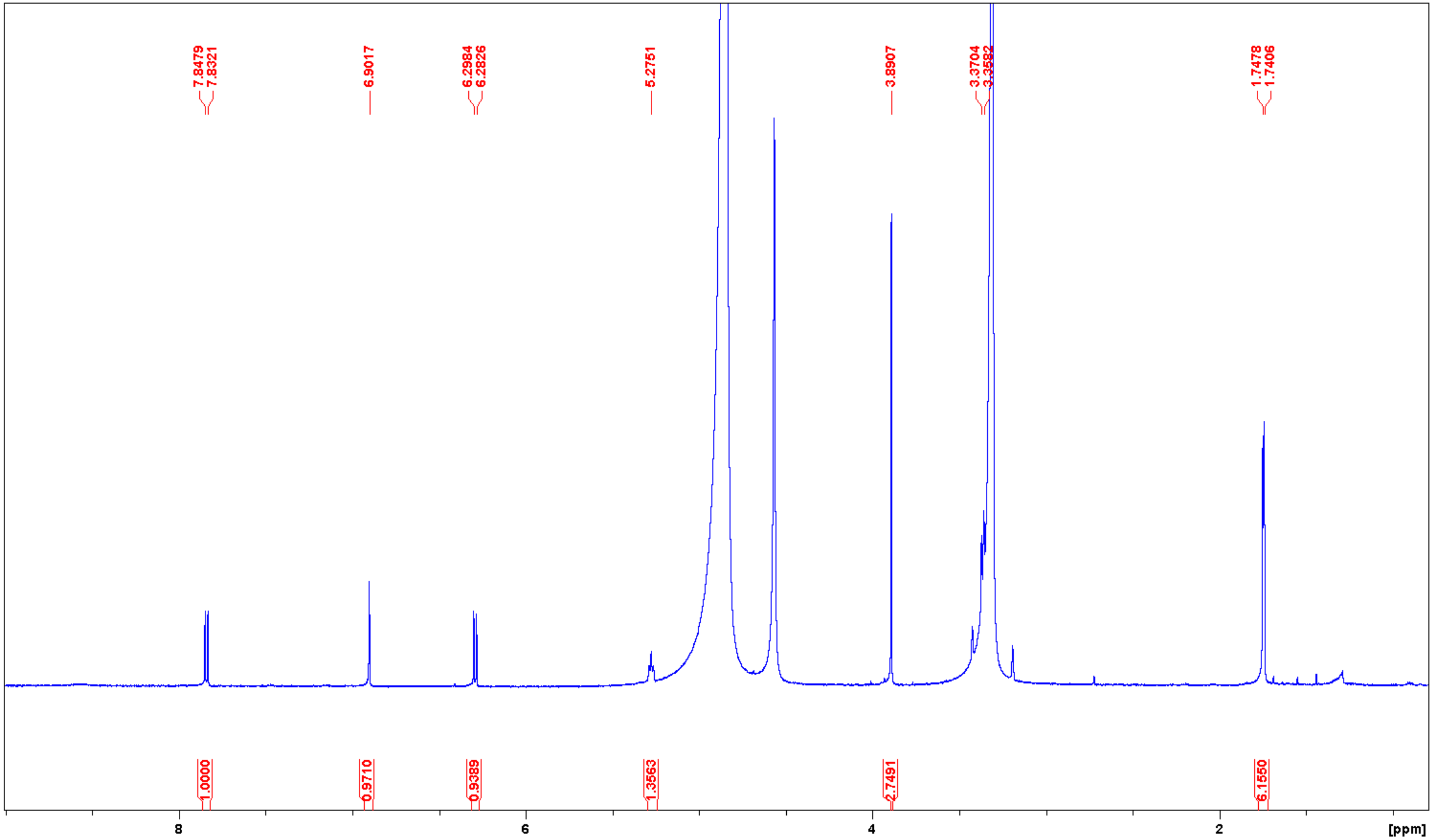

Figure S31. COSY spectrum of **4** (600 MHz, methanol-d_4).

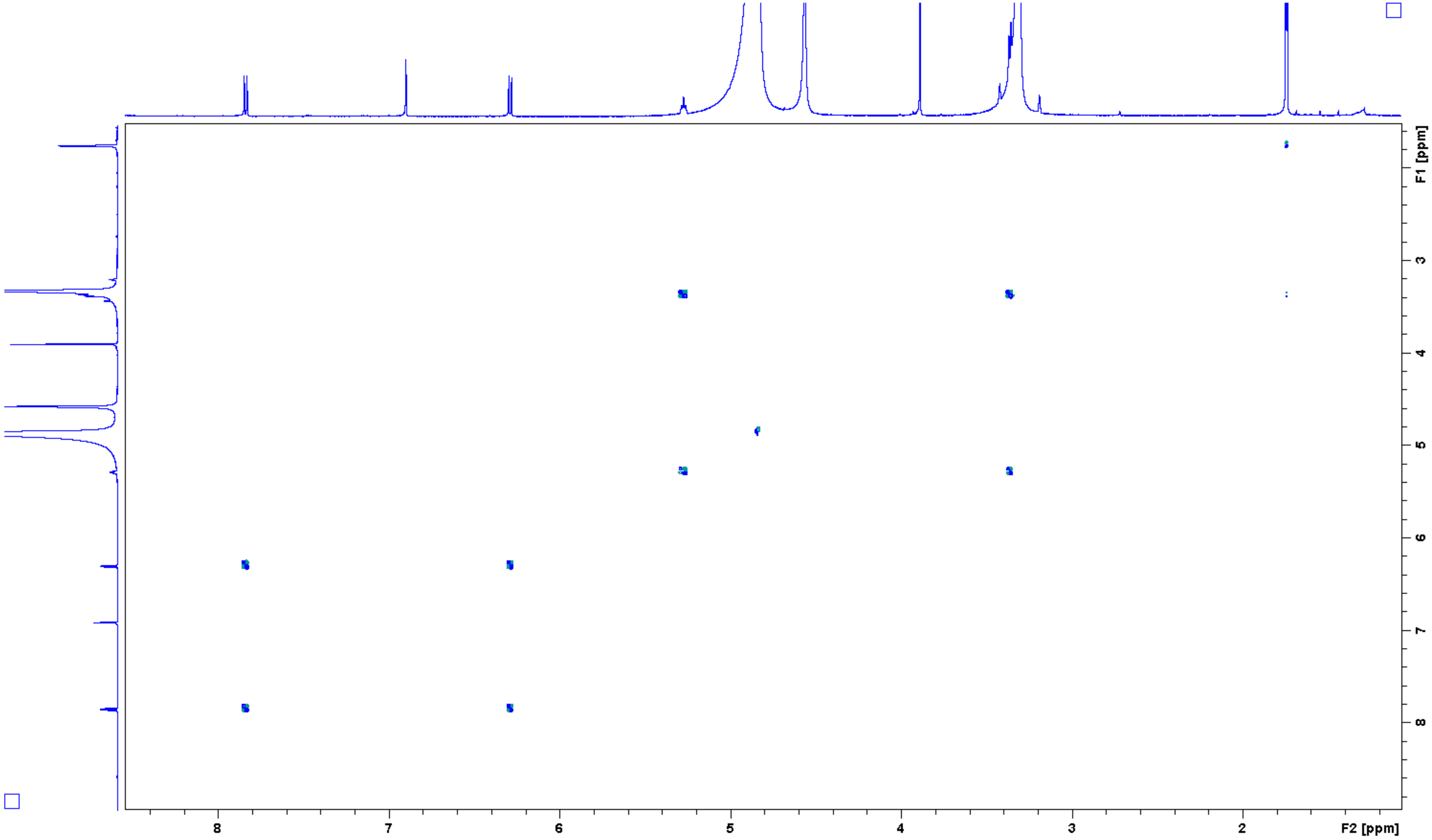

Figure S32. HSQC spectrum of **4** (600 MHz, methanol-d_4).

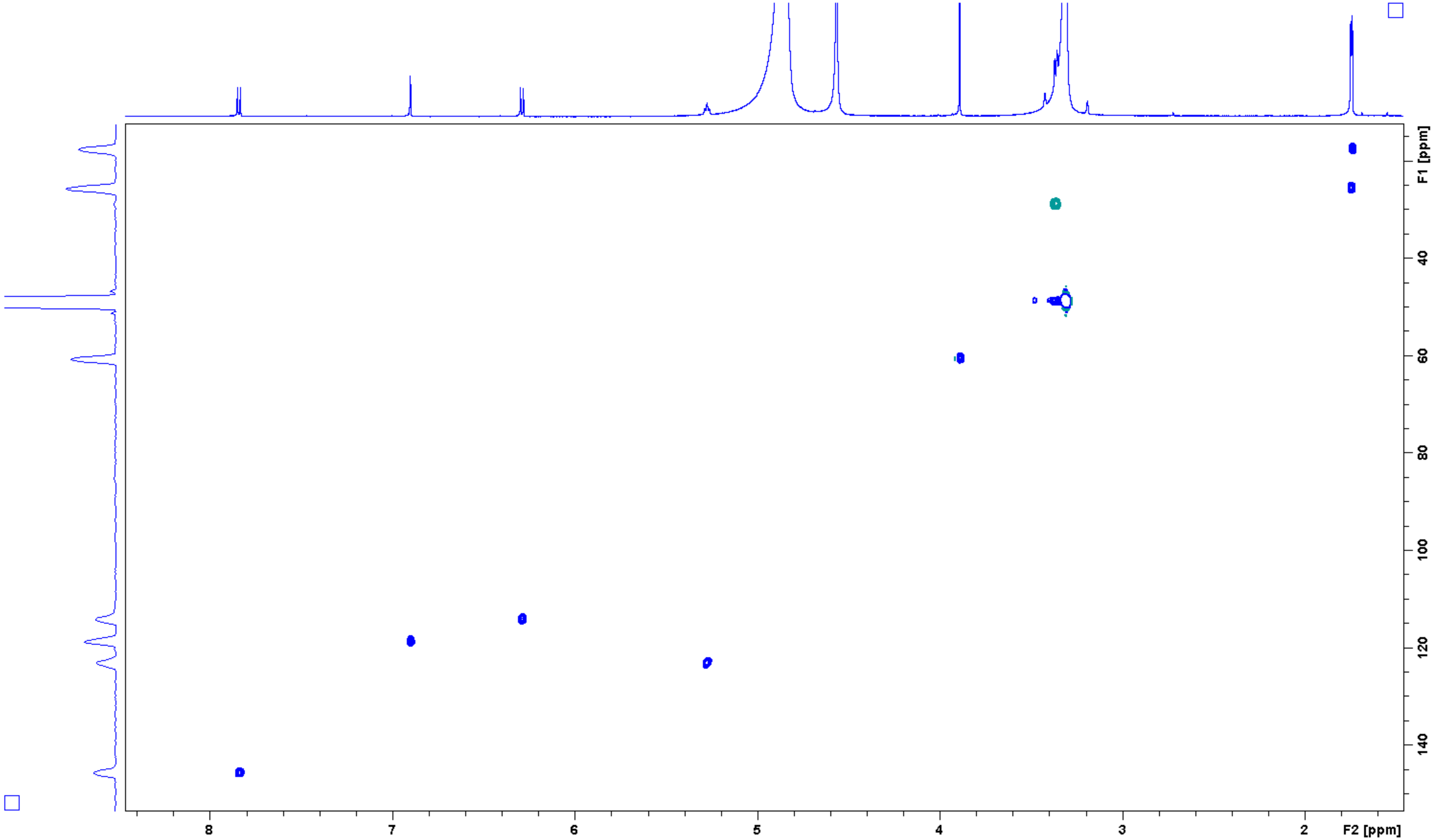

Figure S33. ^{1}H NMR spectrum of **5** (600 MHz, methanol-d_4).

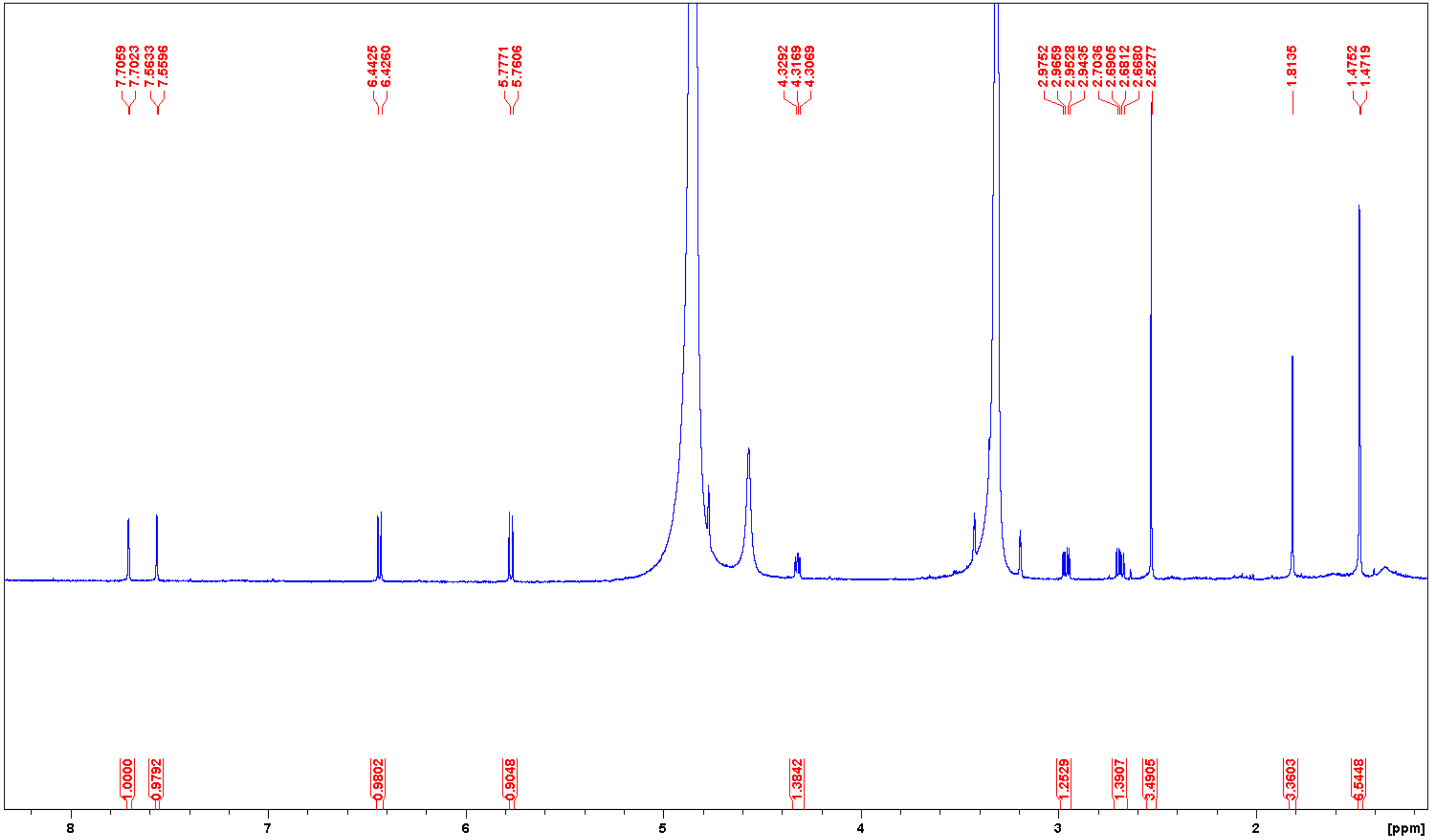

Figure S34. ^{13}C NMR spectrum of **5** (151 MHz, methanol-d_4).

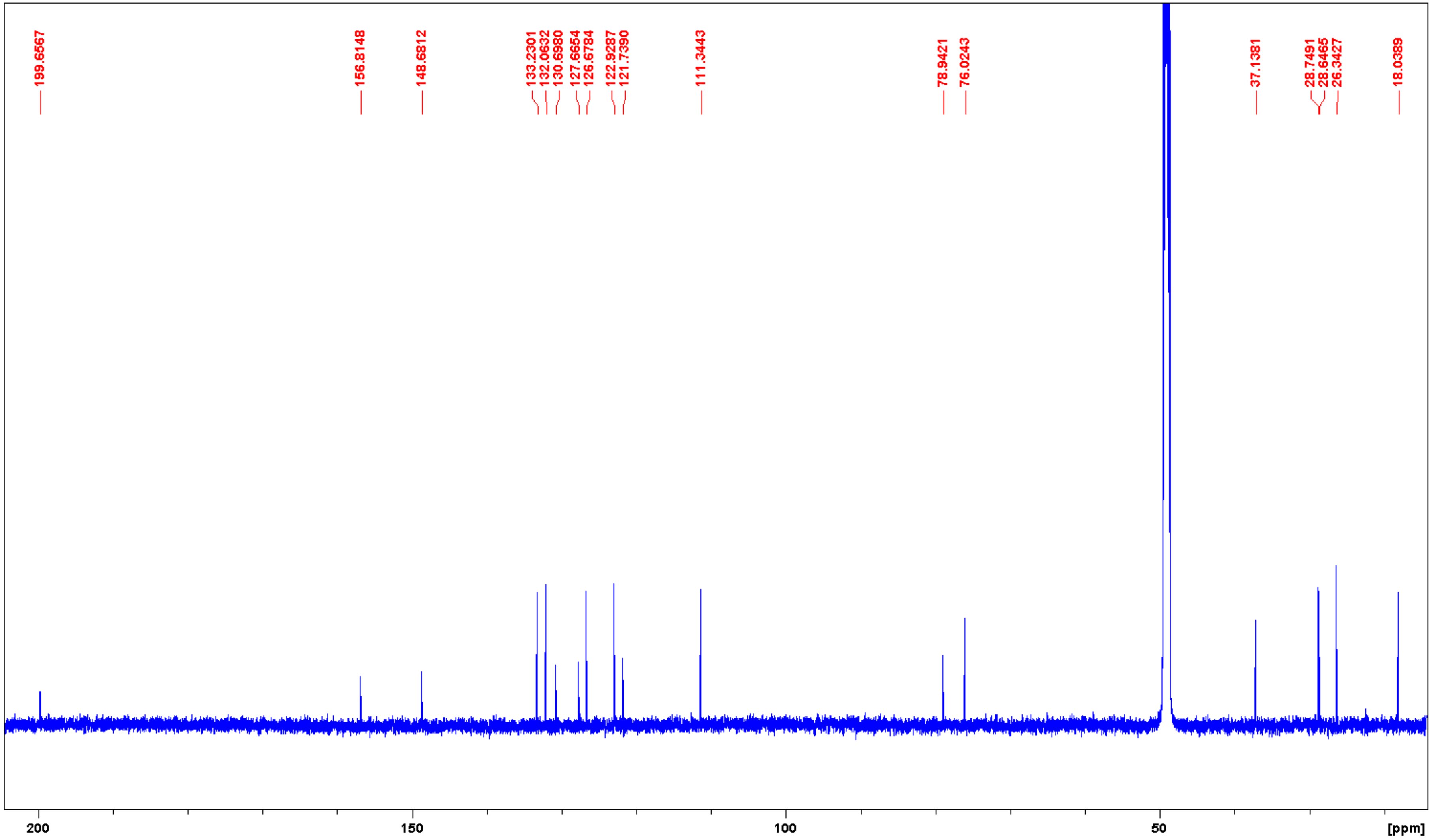

Figure S35. HSQC spectrum of **5** (600 MHz, methanol-d_4).

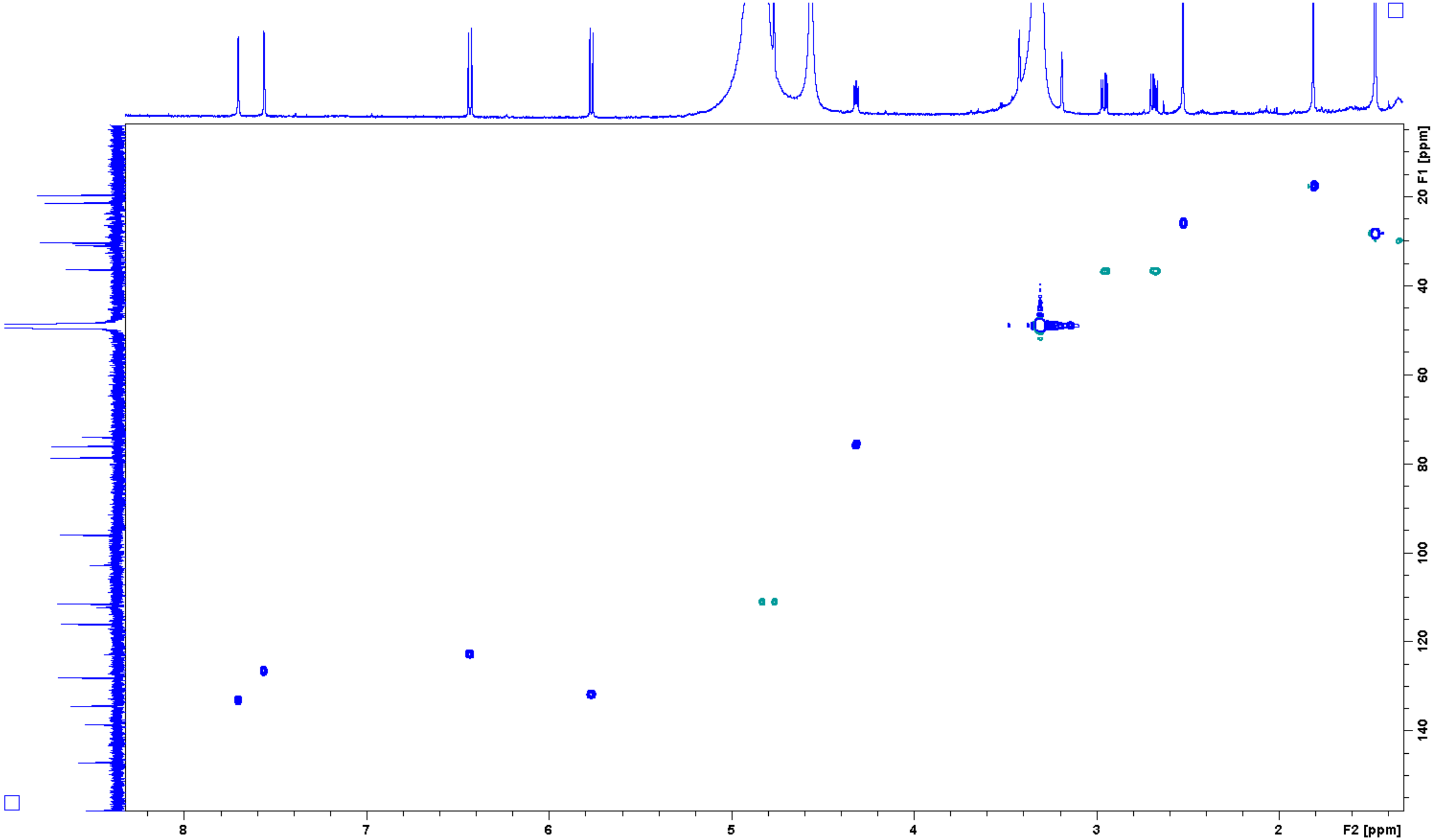

Figure S36. COSY spectrum of **5** (600 MHz, methanol-d_4).

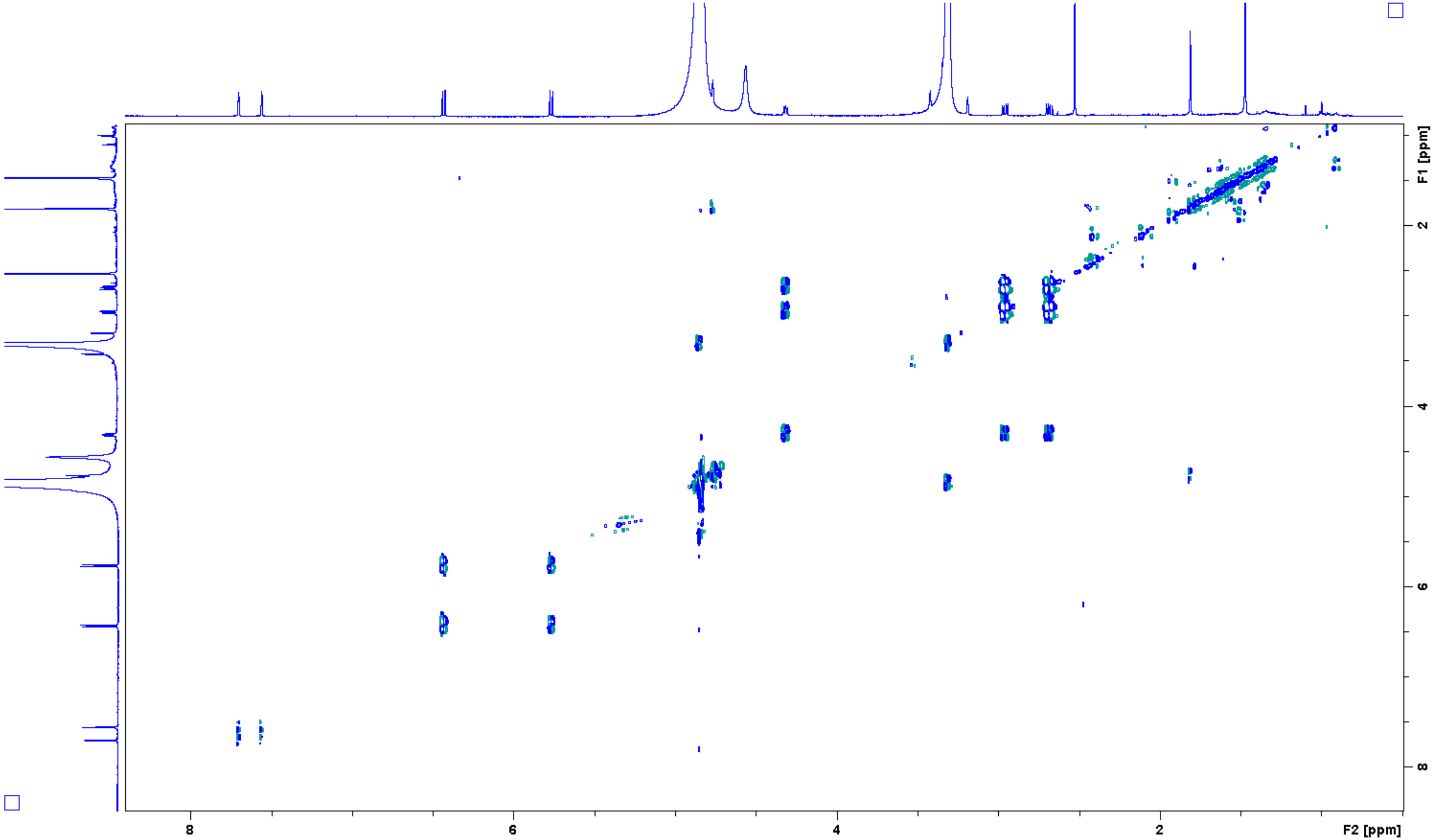

Figure S37. HMBC spectrum of **5** (600 MHz, methanol-d_4).

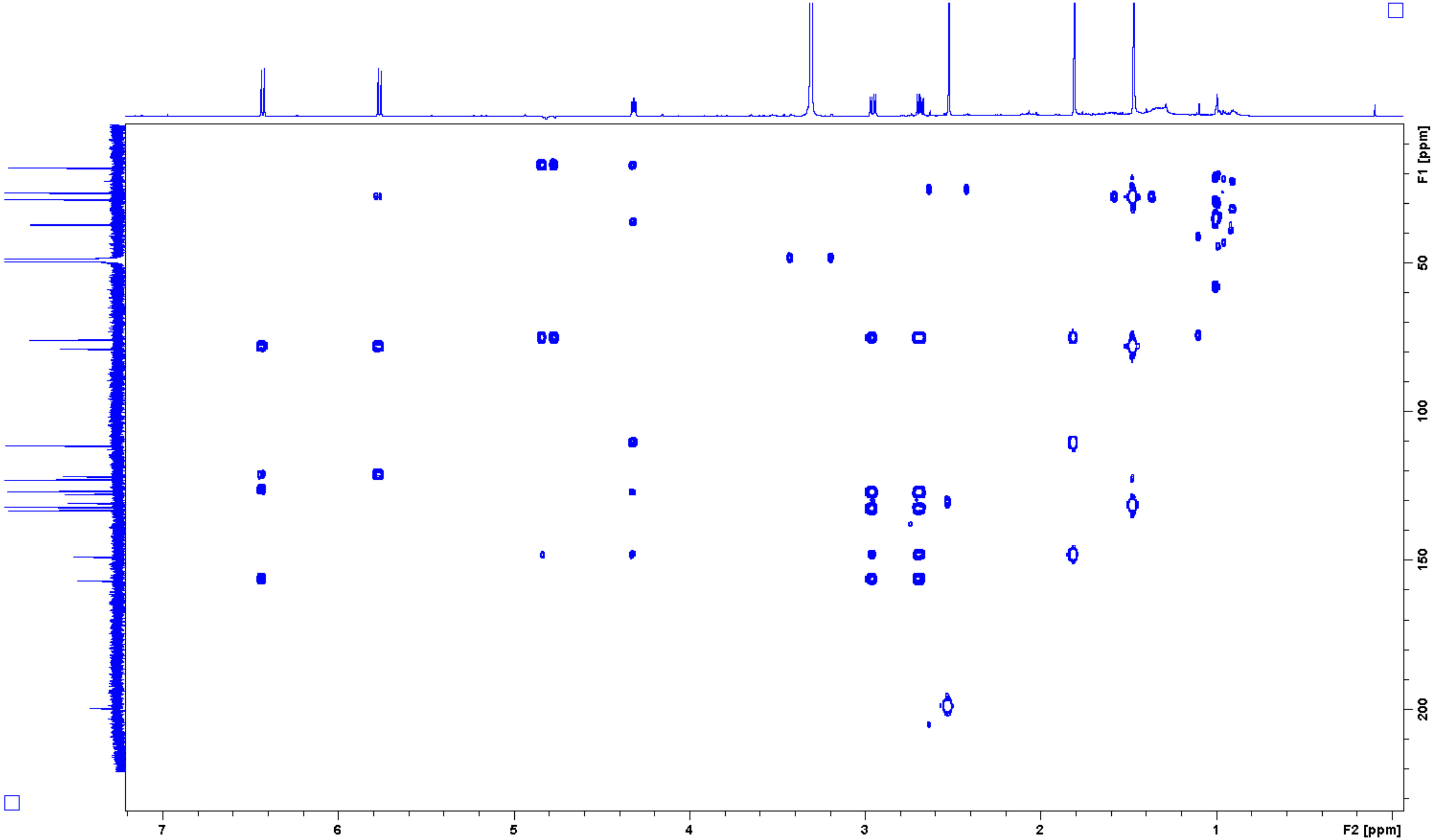

Figure S38. ROESY spectrum of **5** (600 MHz, methanol-d_4).

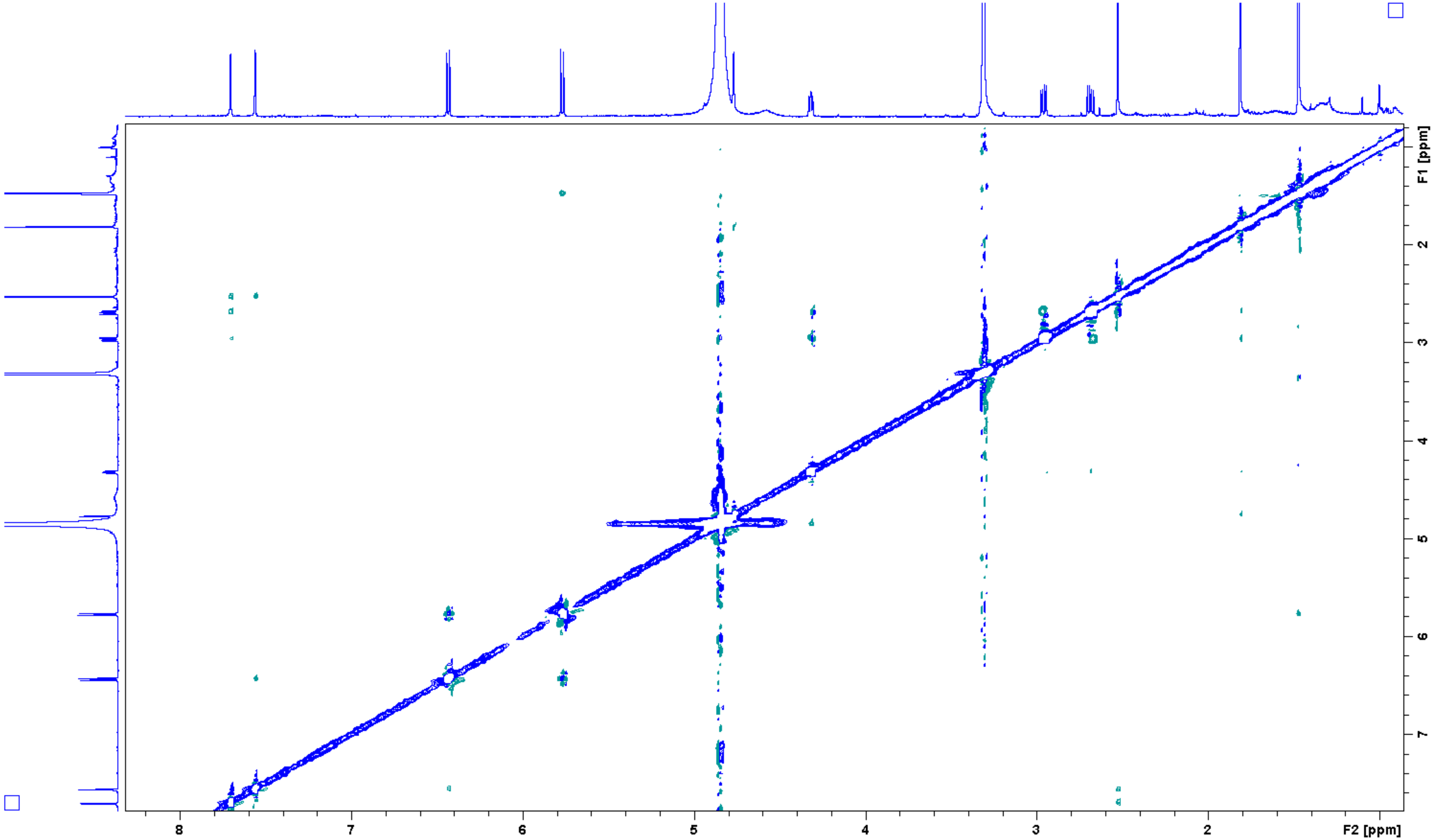

Figure S39. ^{1}H NMR spectrum of **6** (600 MHz, methanol-d_4).

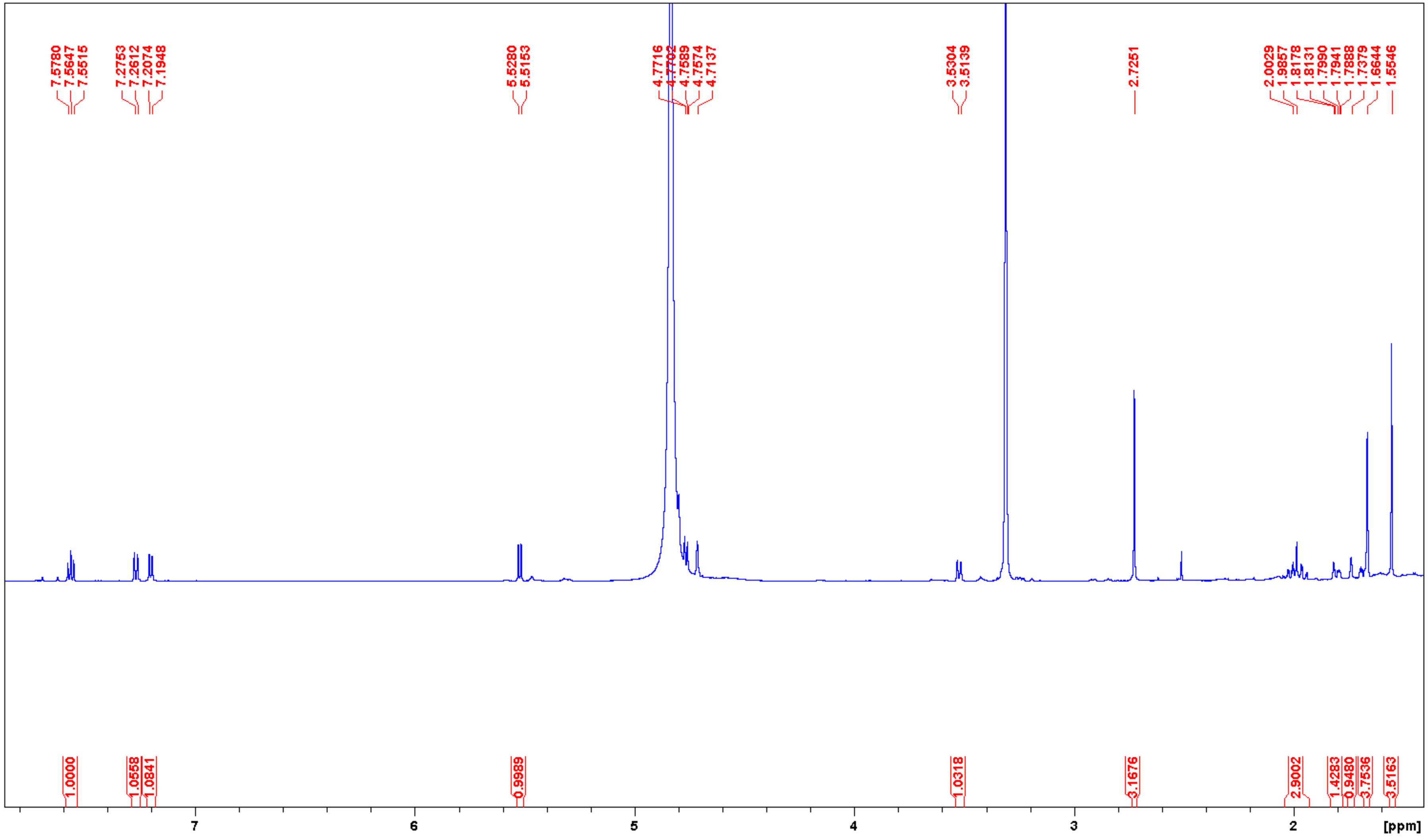

Figure S40. ^{13}C NMR spectrum of **6** (151 MHz, methanol-d_4).

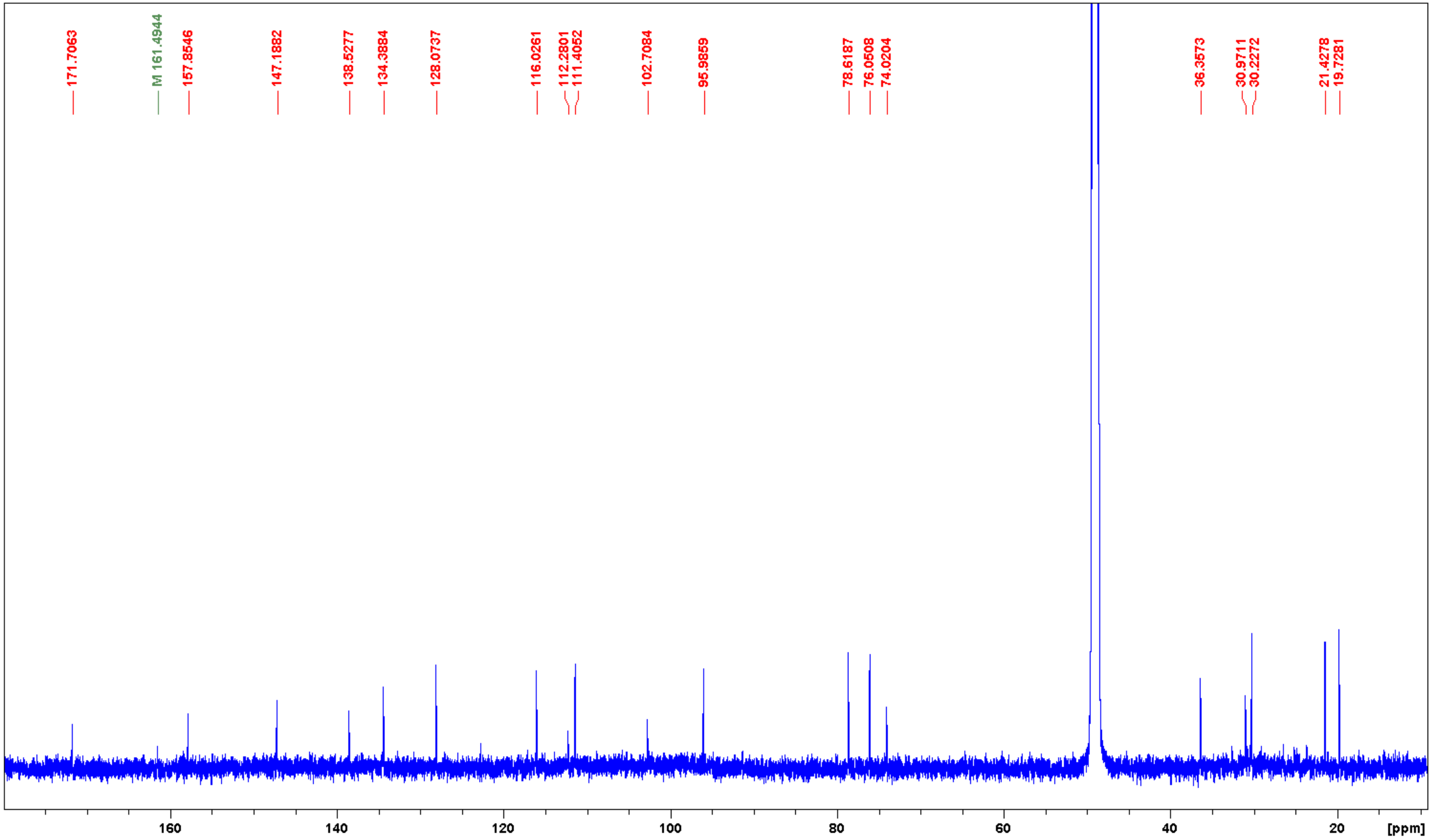

Figure S41. HSQC spectrum of **6** (600 MHz, methanol-d_4).

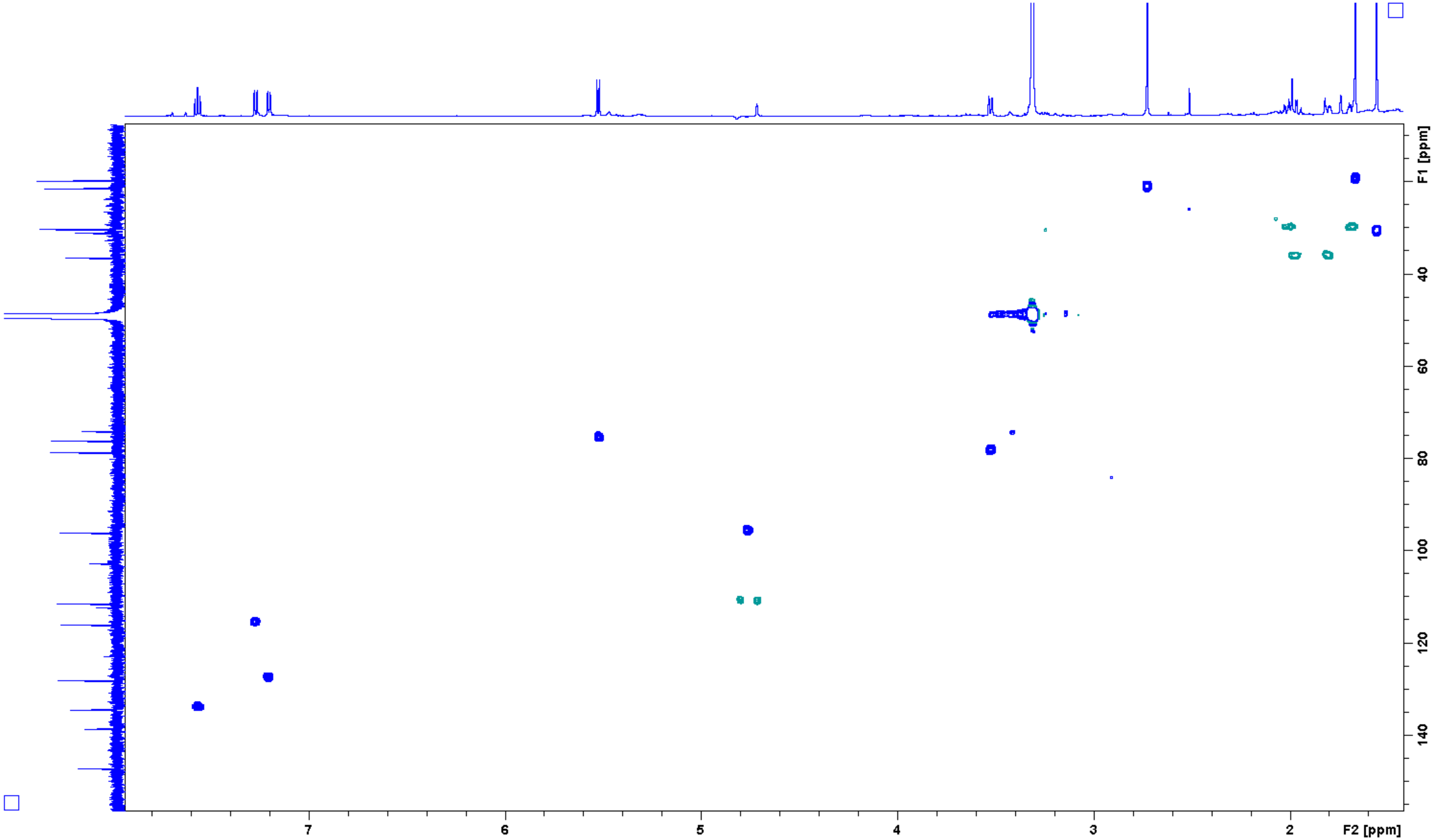

Figure S42. COSY spectrum of **6** (600 MHz, methanol-d_4).

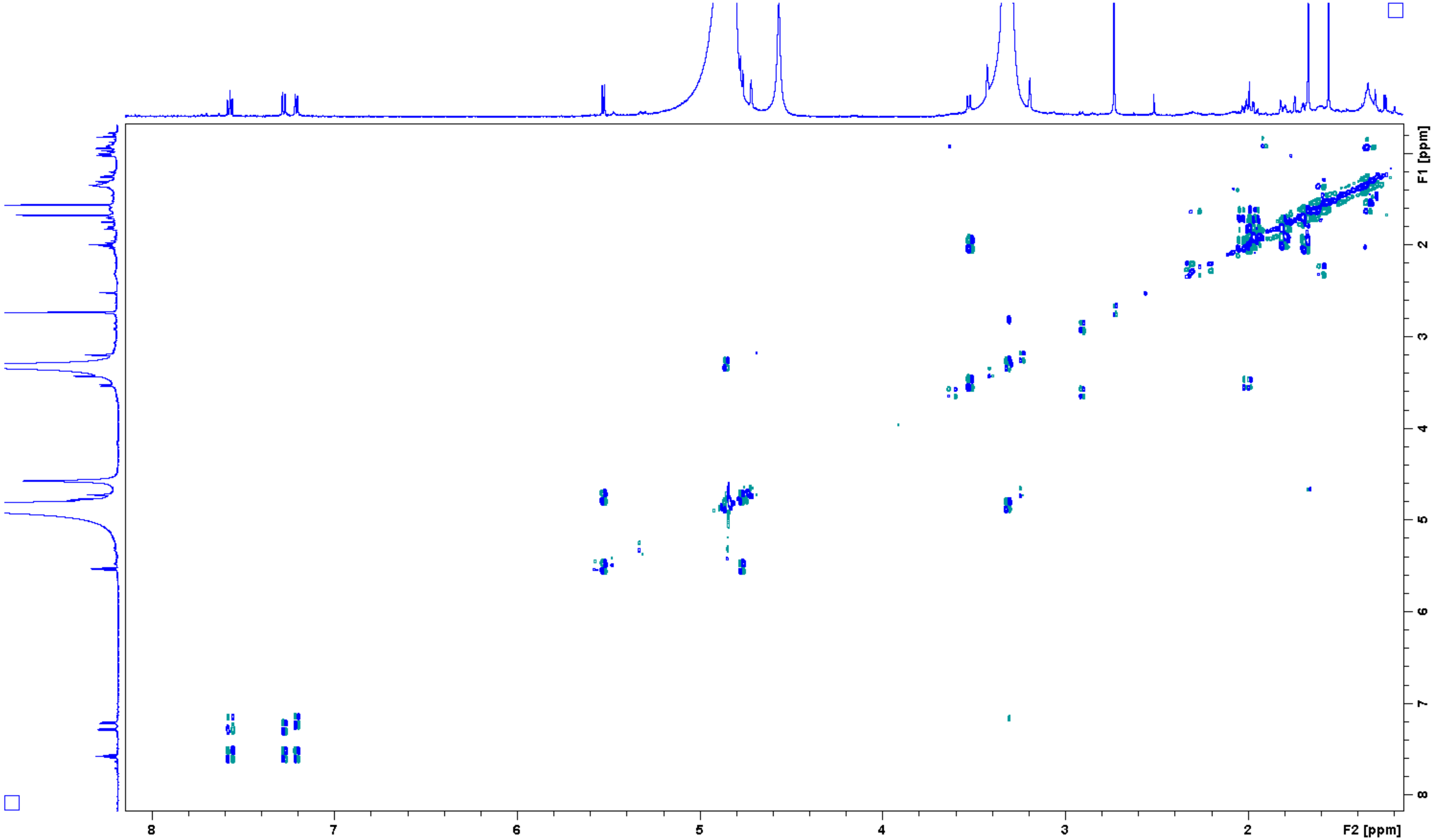

Figure S43. HMBC spectrum of **6** (600 MHz, methanol-d_4).

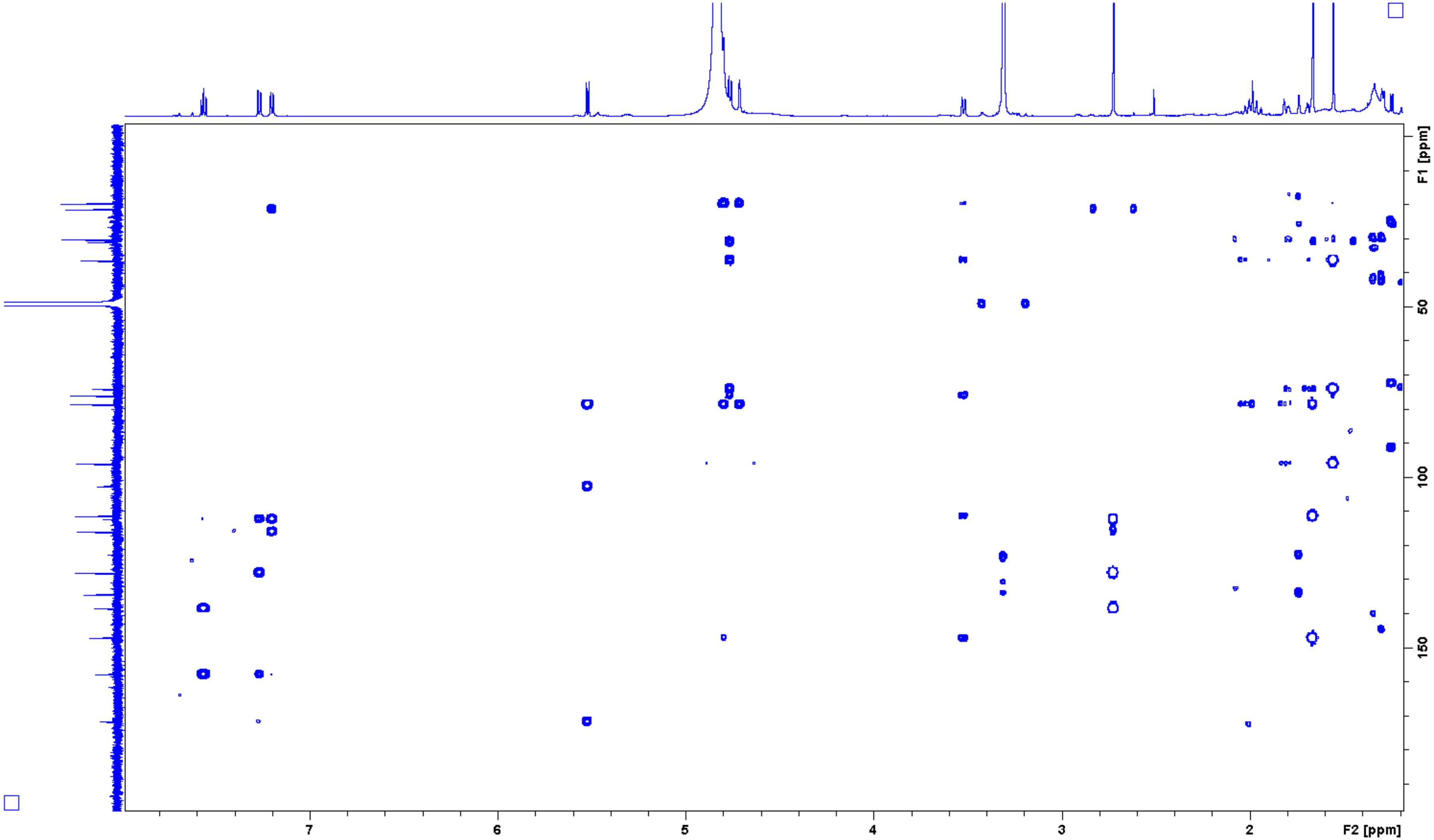

Figure S44. ROESY spectrum of **6** (600 MHz, methanol-d_4).

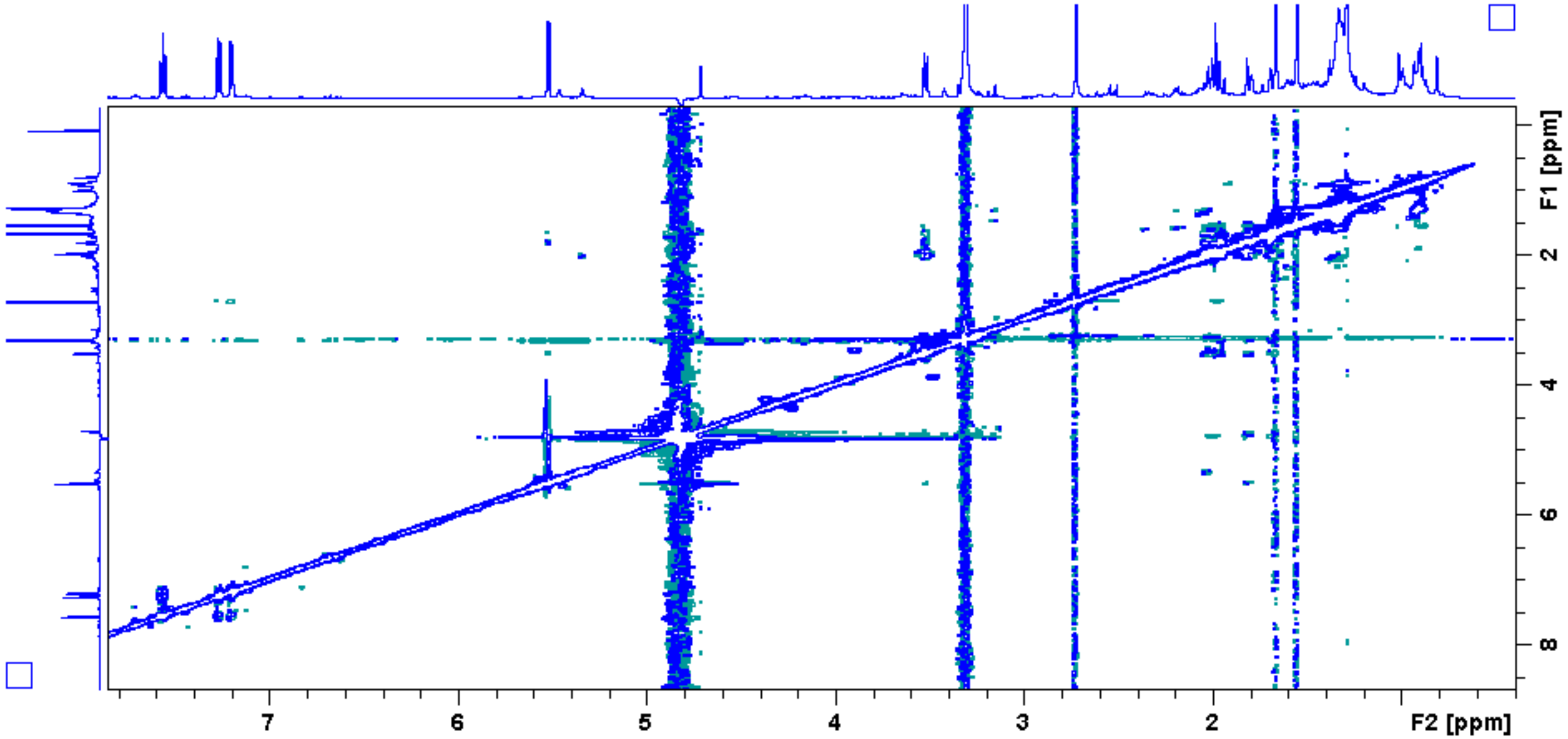

Figure S45. ^{1}H NMR spectrum of **7** (600 MHz, chloroform-*d*).

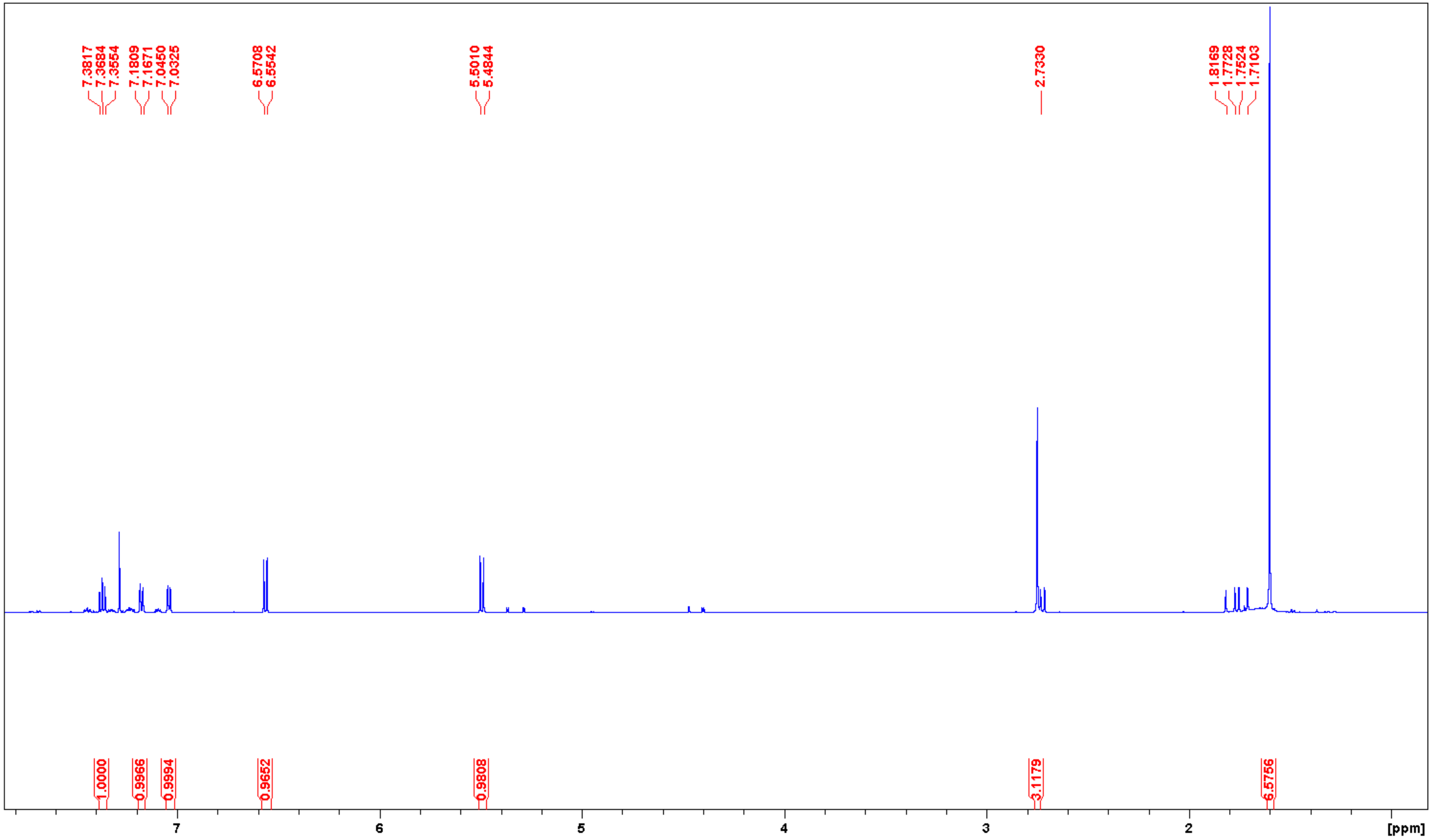

Figure S46. COSY spectrum of **7** (600 MHz, chloroform-*d*).

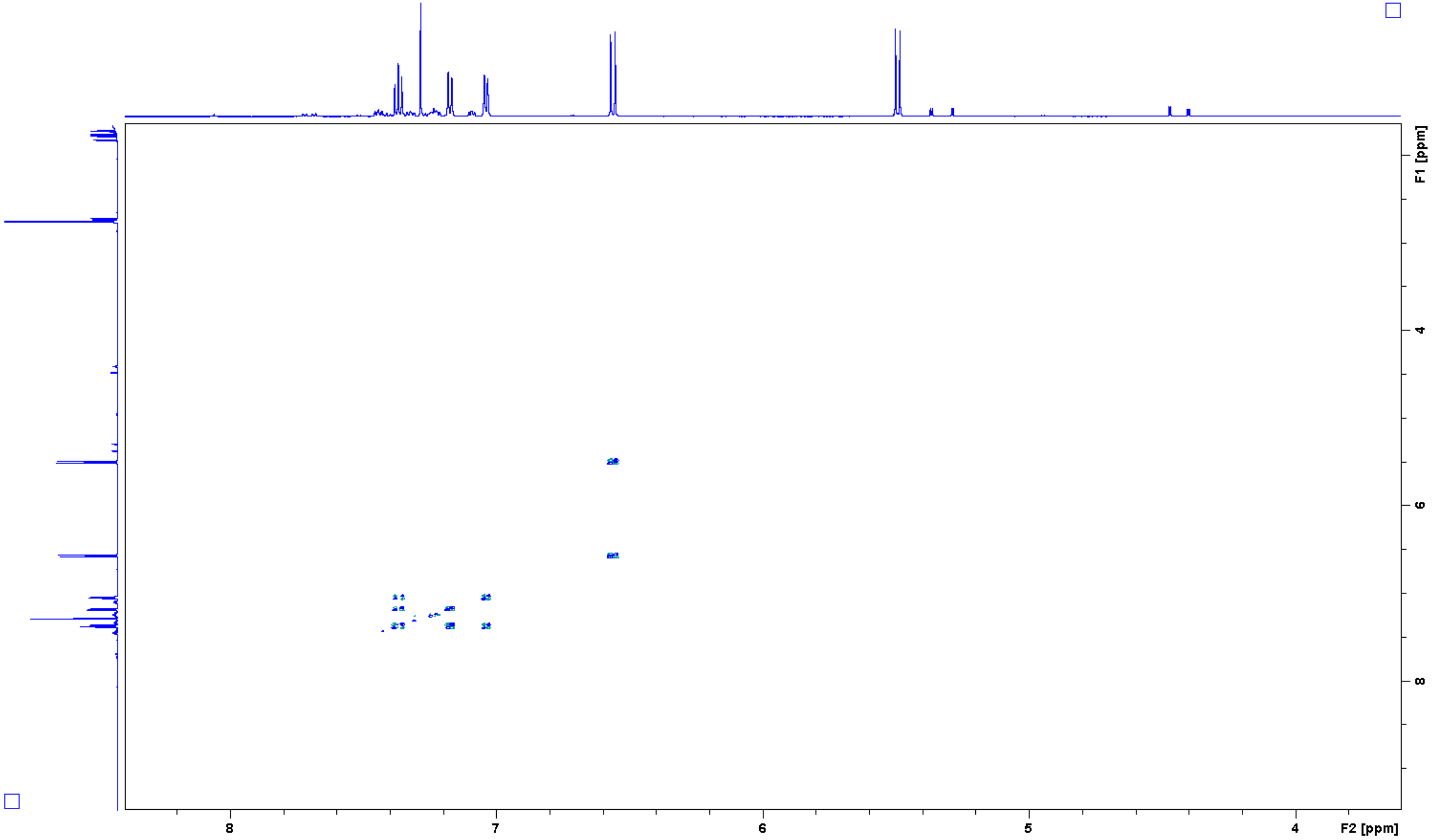

Figure S47. HQSC spectrum of **7** (600 MHz, chloroform-*d*)**.**

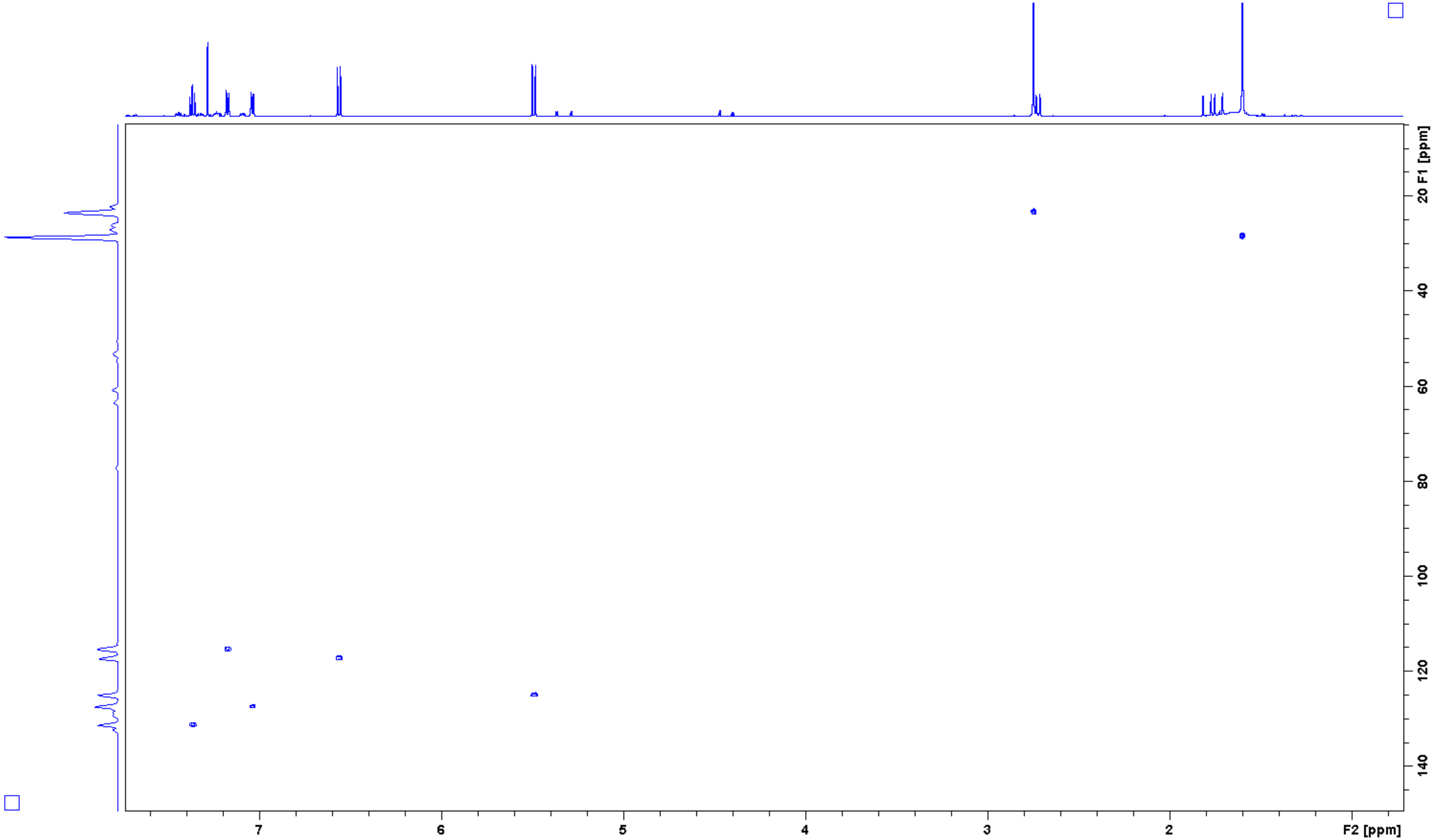

Figure S48. ^{1}H NMR spectrum of **8** (600 MHz, methanol-d_4).

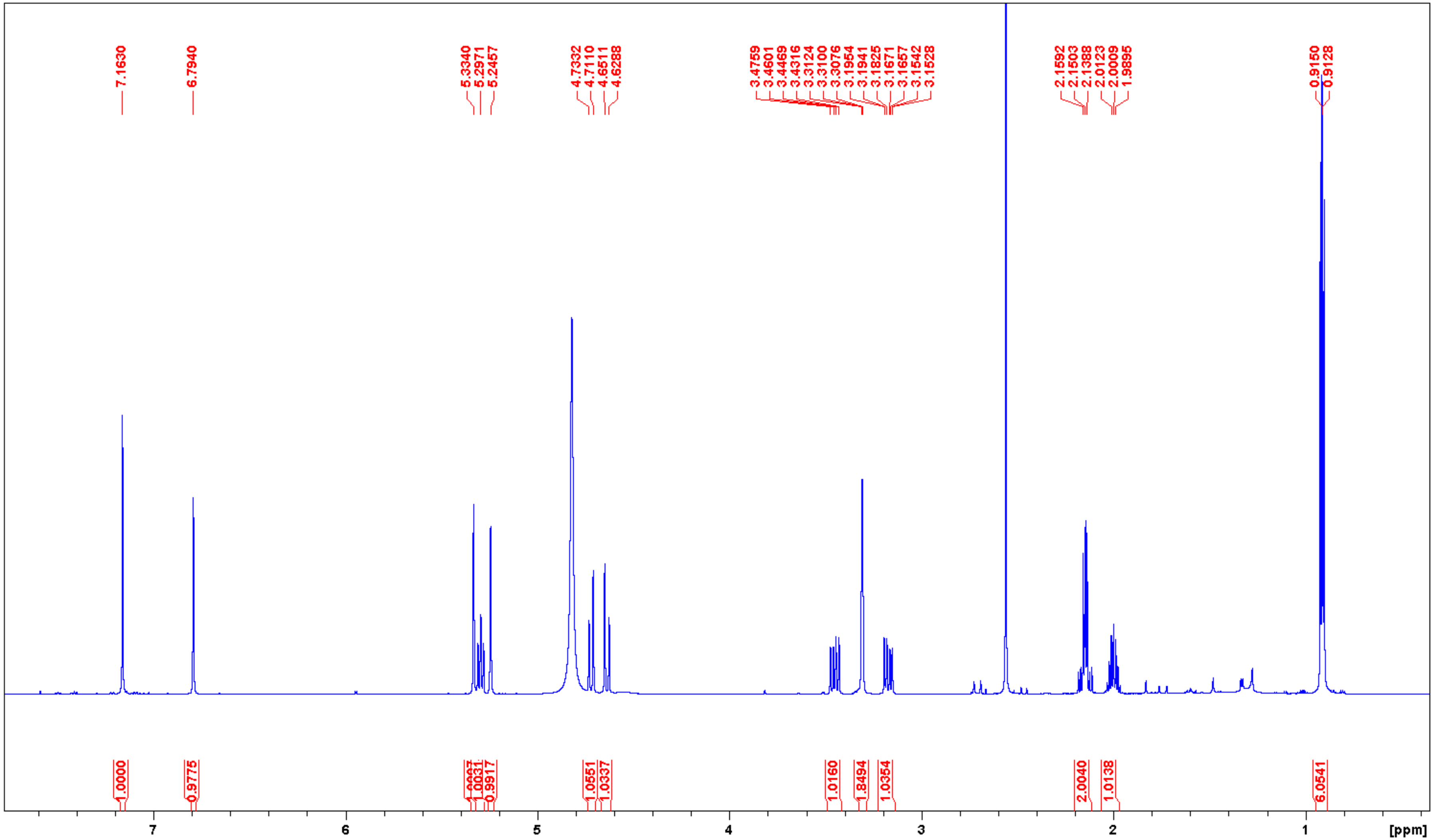

Figure S49. HSQC spectrum of **8** (600 MHz, methanol-d_4).

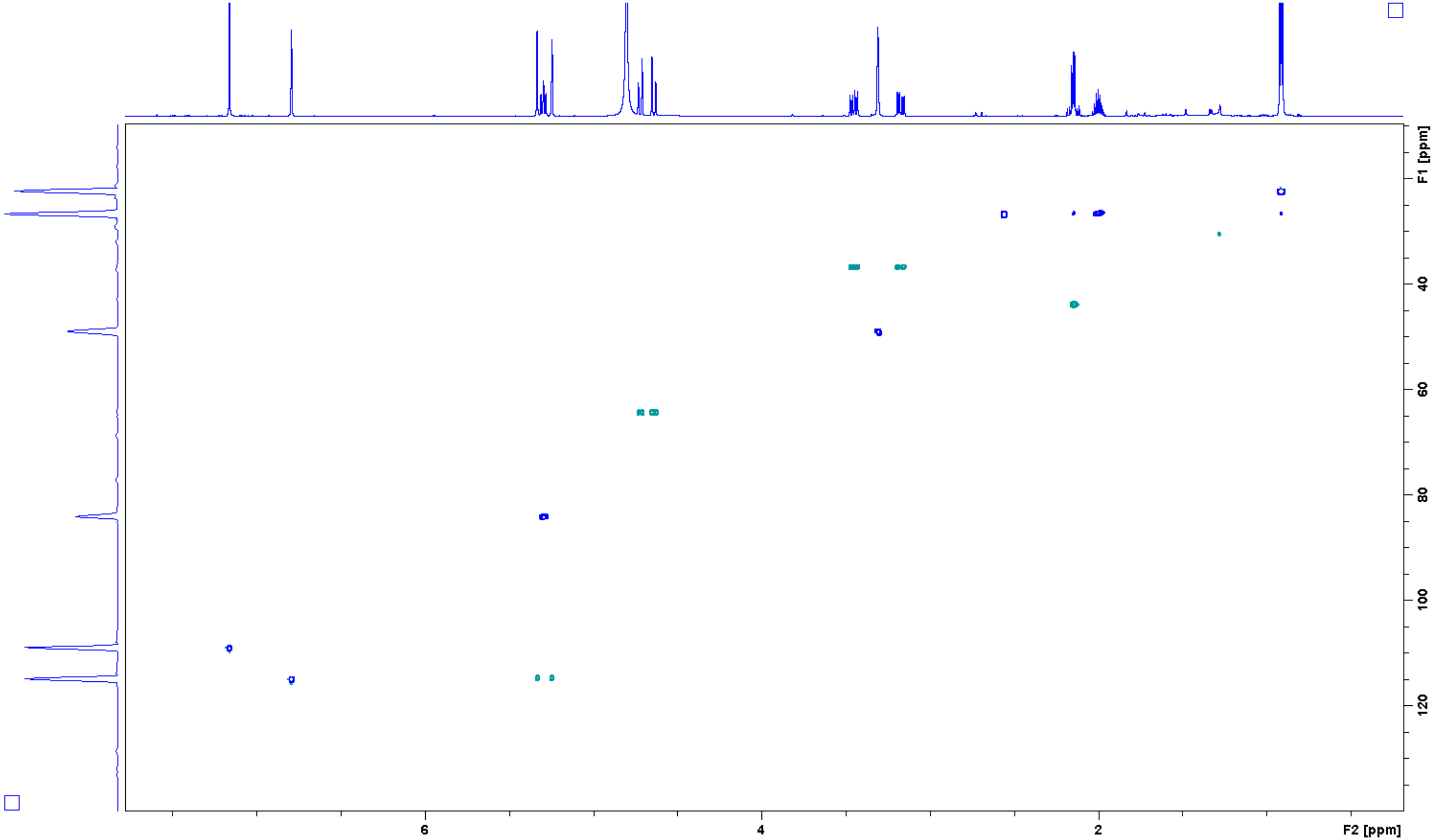

Figure S50. COSY spectrum of 8 (600 MHz, methanol-d_4).

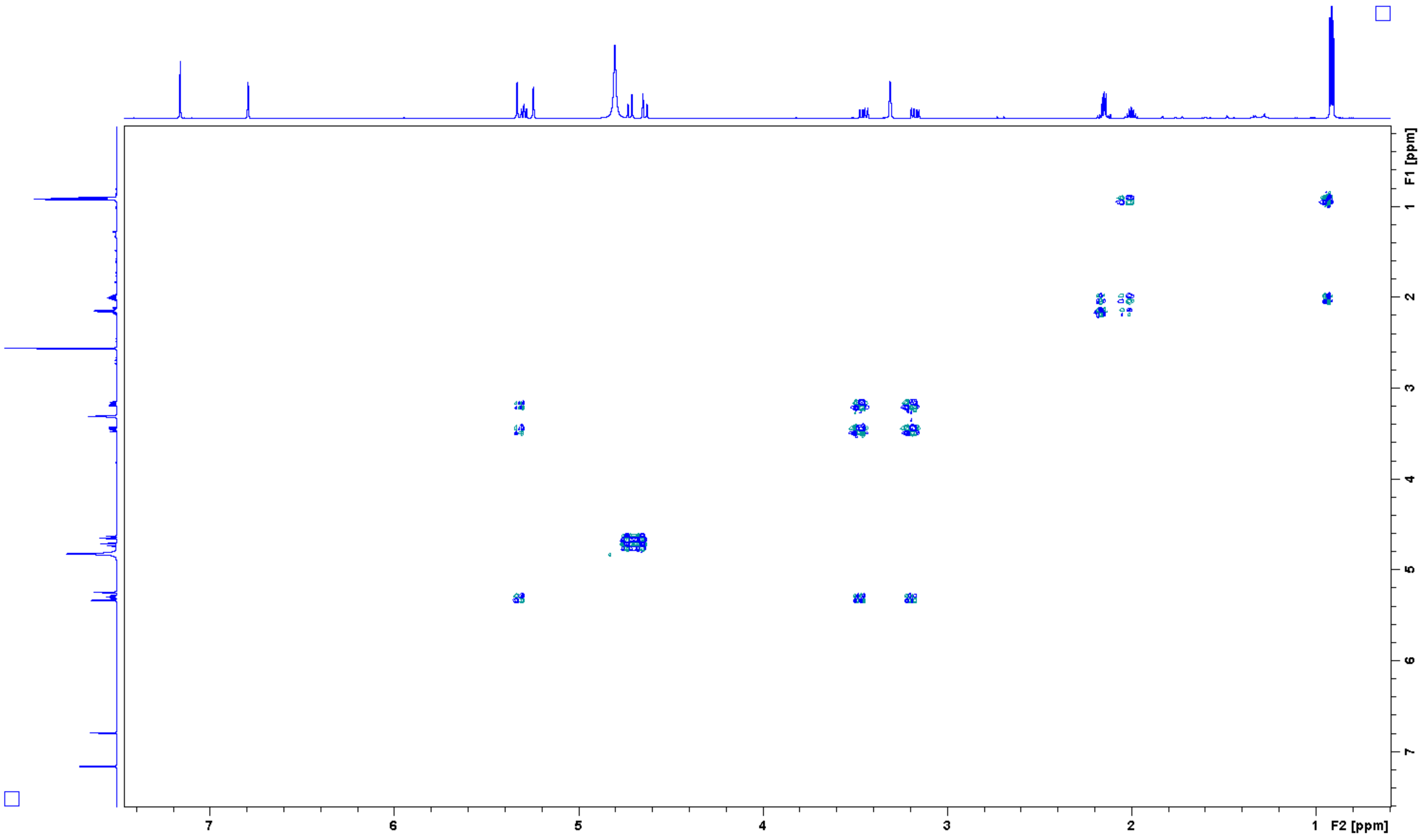

Figure S51. HMBC spectrum of **8** (600 MHz, methanol-d_4).

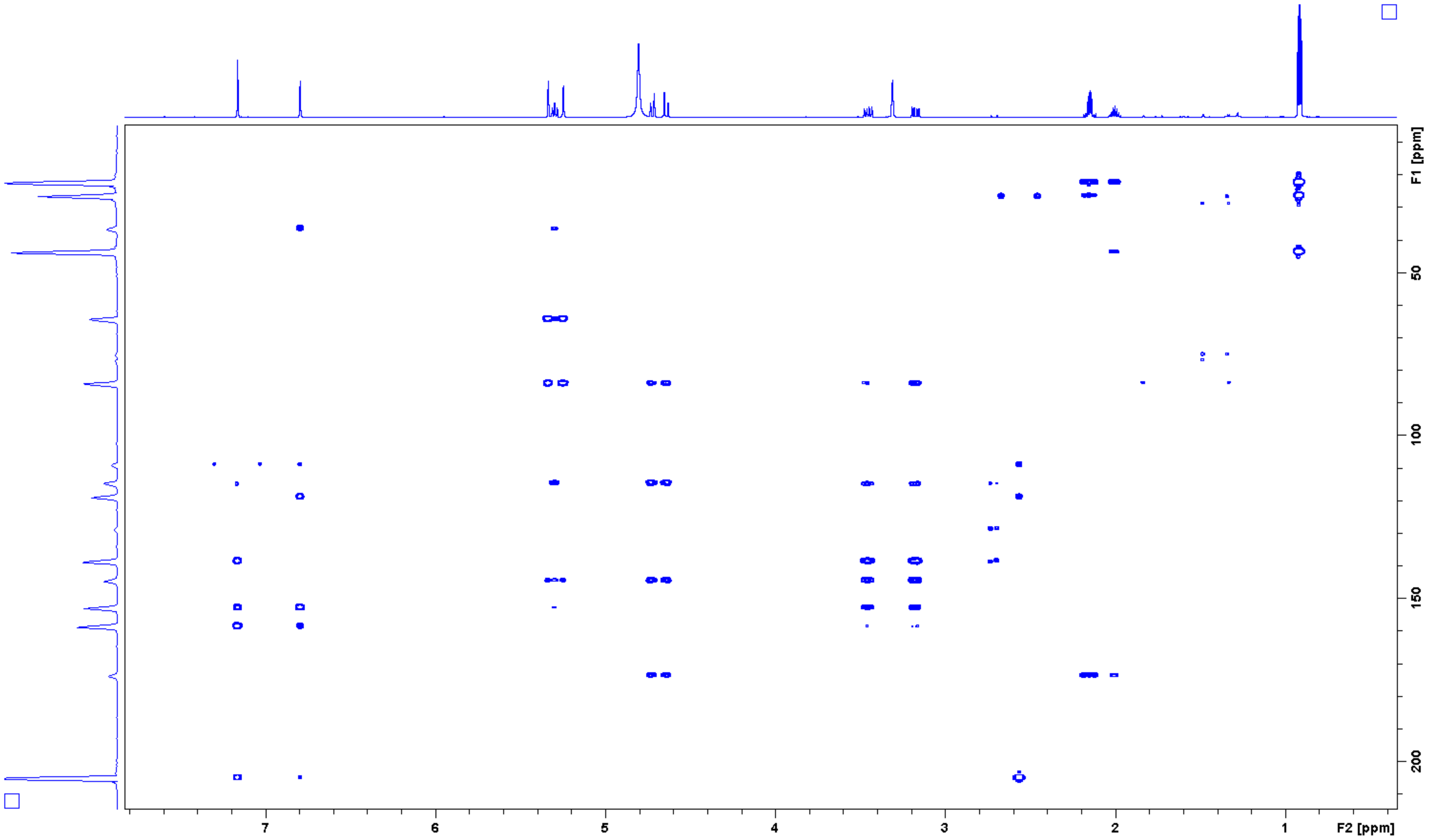

Figure S52. ROESY spectrum of **8** (600 MHz, methanol-d_4).

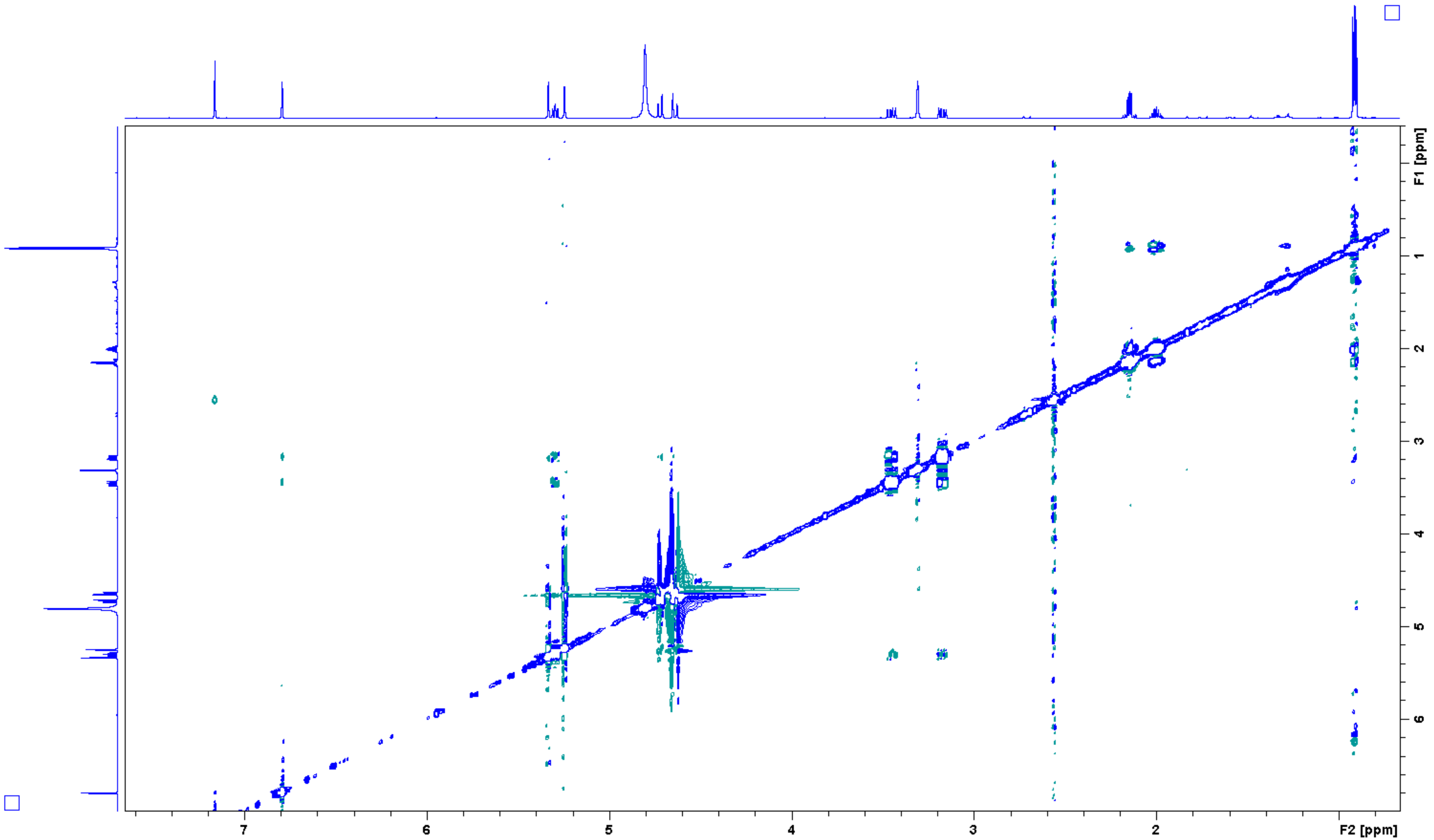

Figure S53. ^{1}H NMR spectrum of **9** (600 MHz, methanol-d_4).

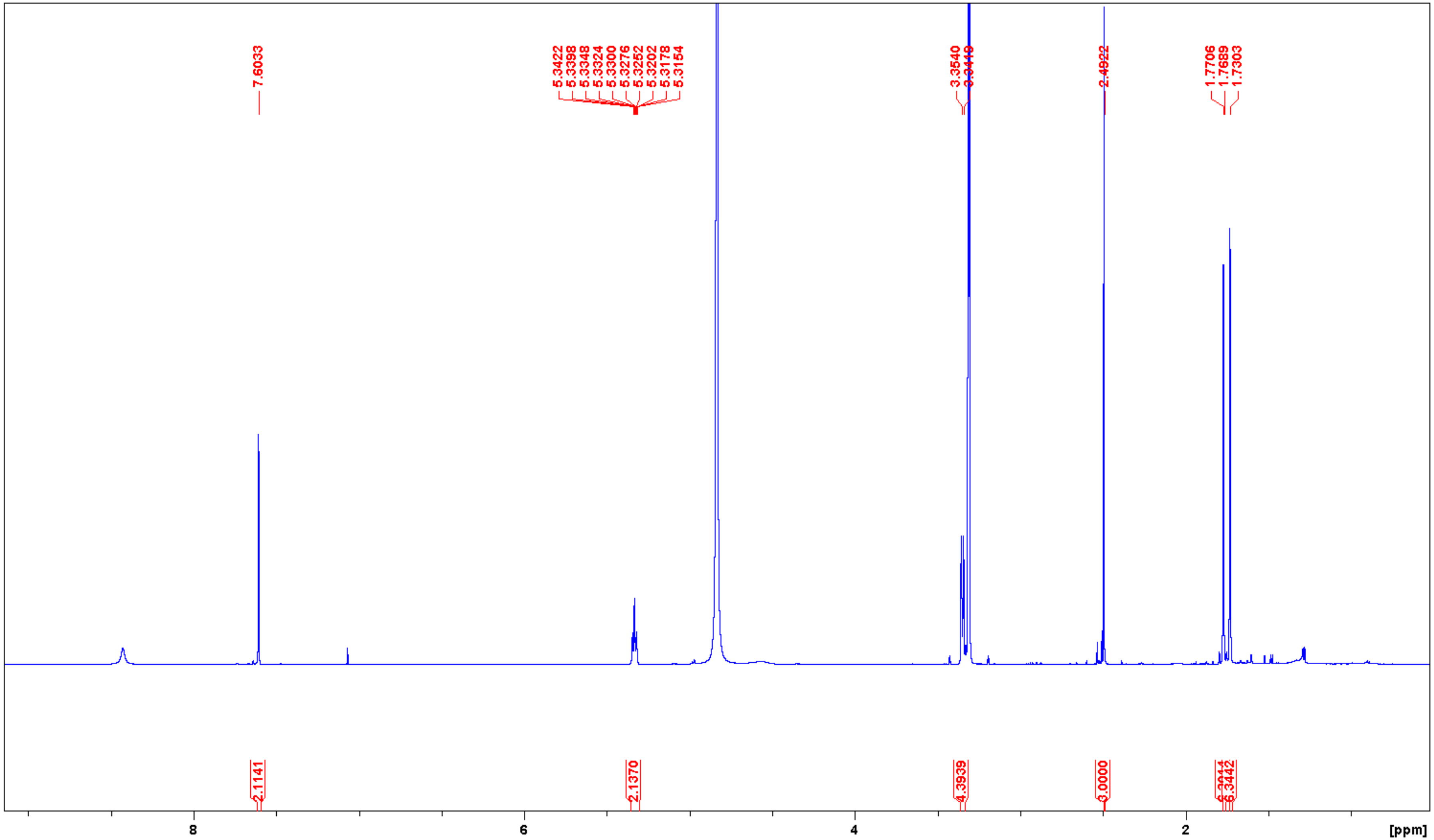

Figure S54. HSQC spectrum of **9** (600 MHz, methanol-d_4).

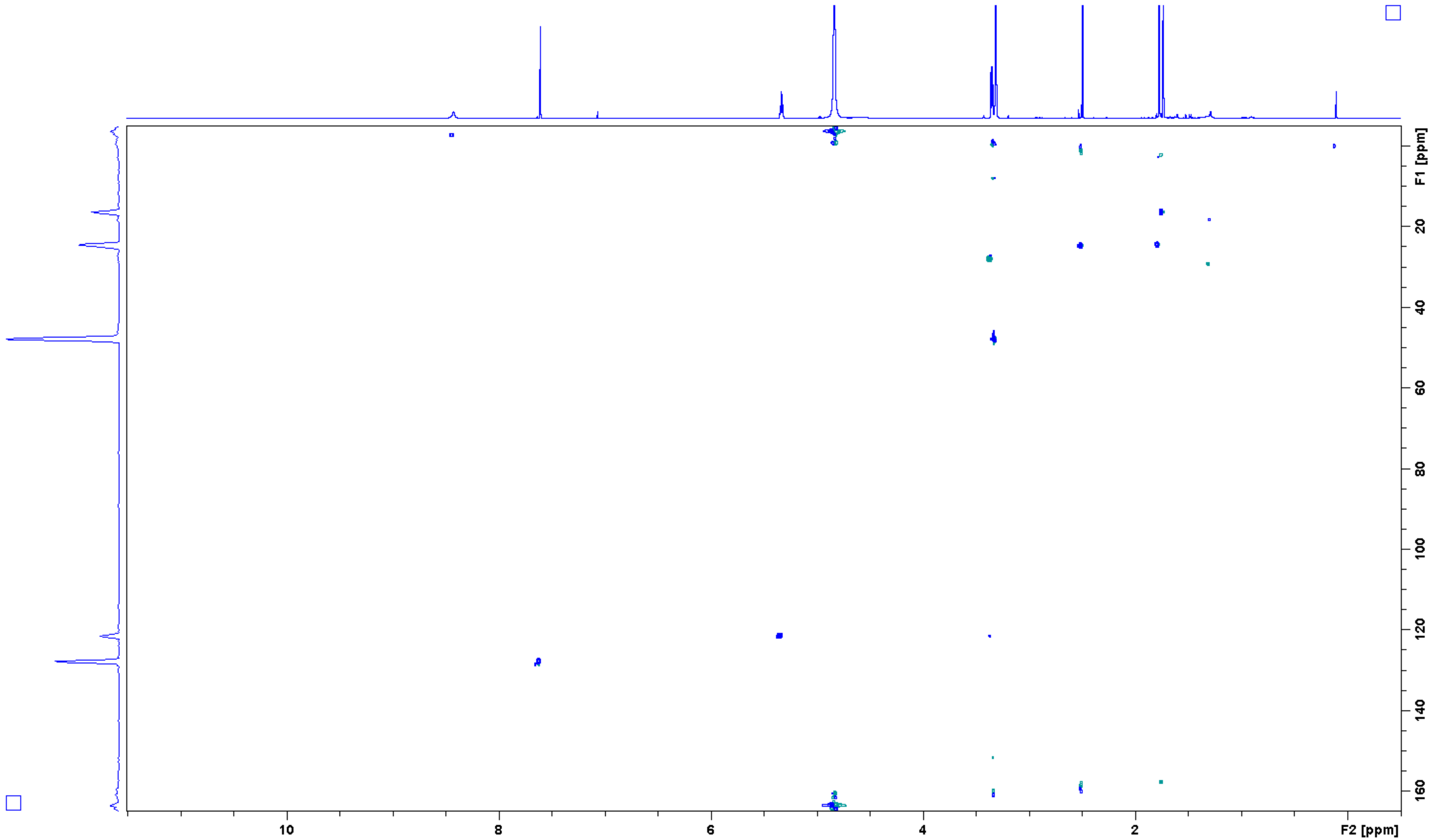

Figure S55. HMBC spectrum of **9** (600 MHz, methanol-d_4).

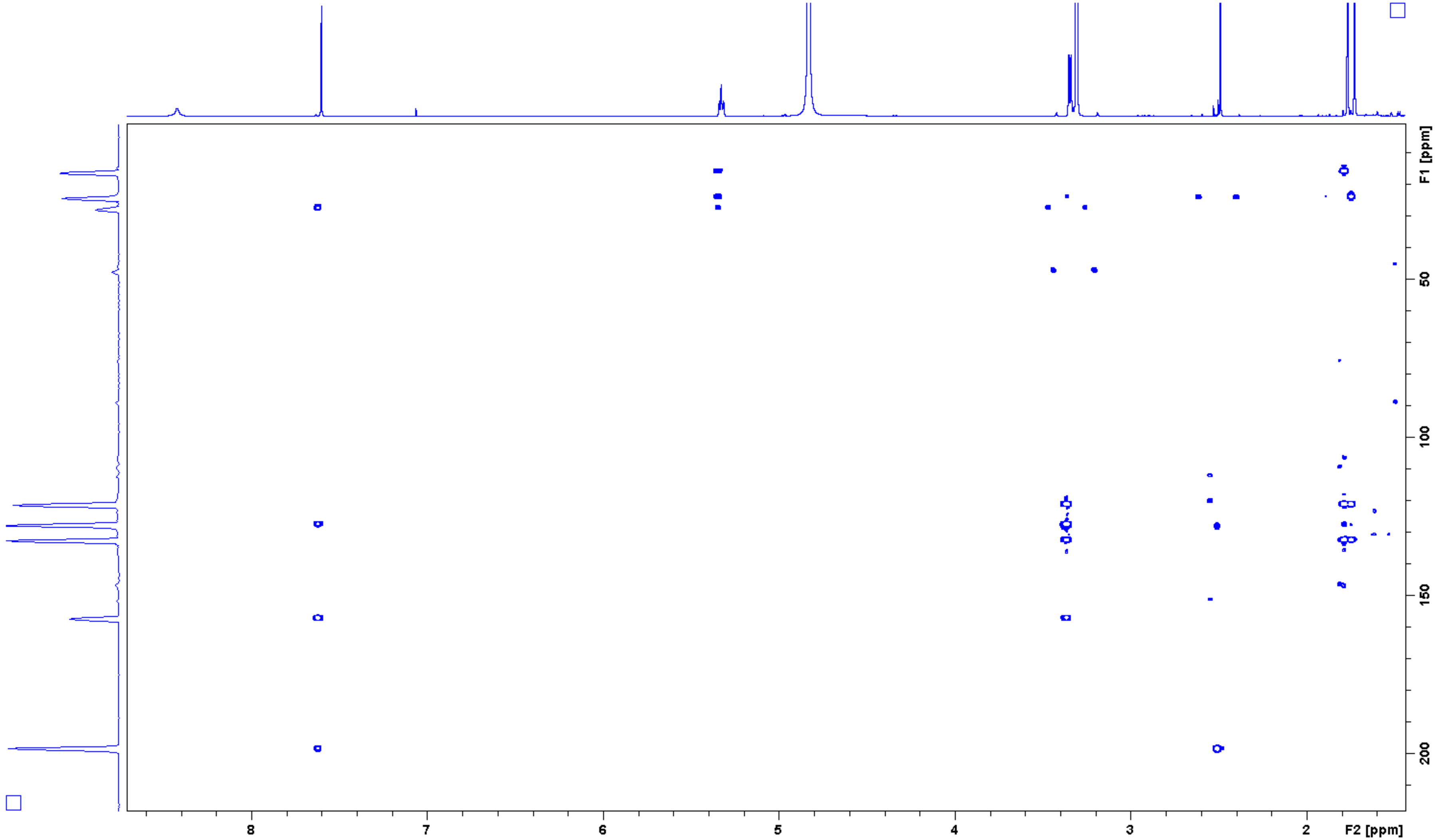

Figure S56. ^{1}H NMR spectrum of **10** (600 MHz, methanol-d_4).

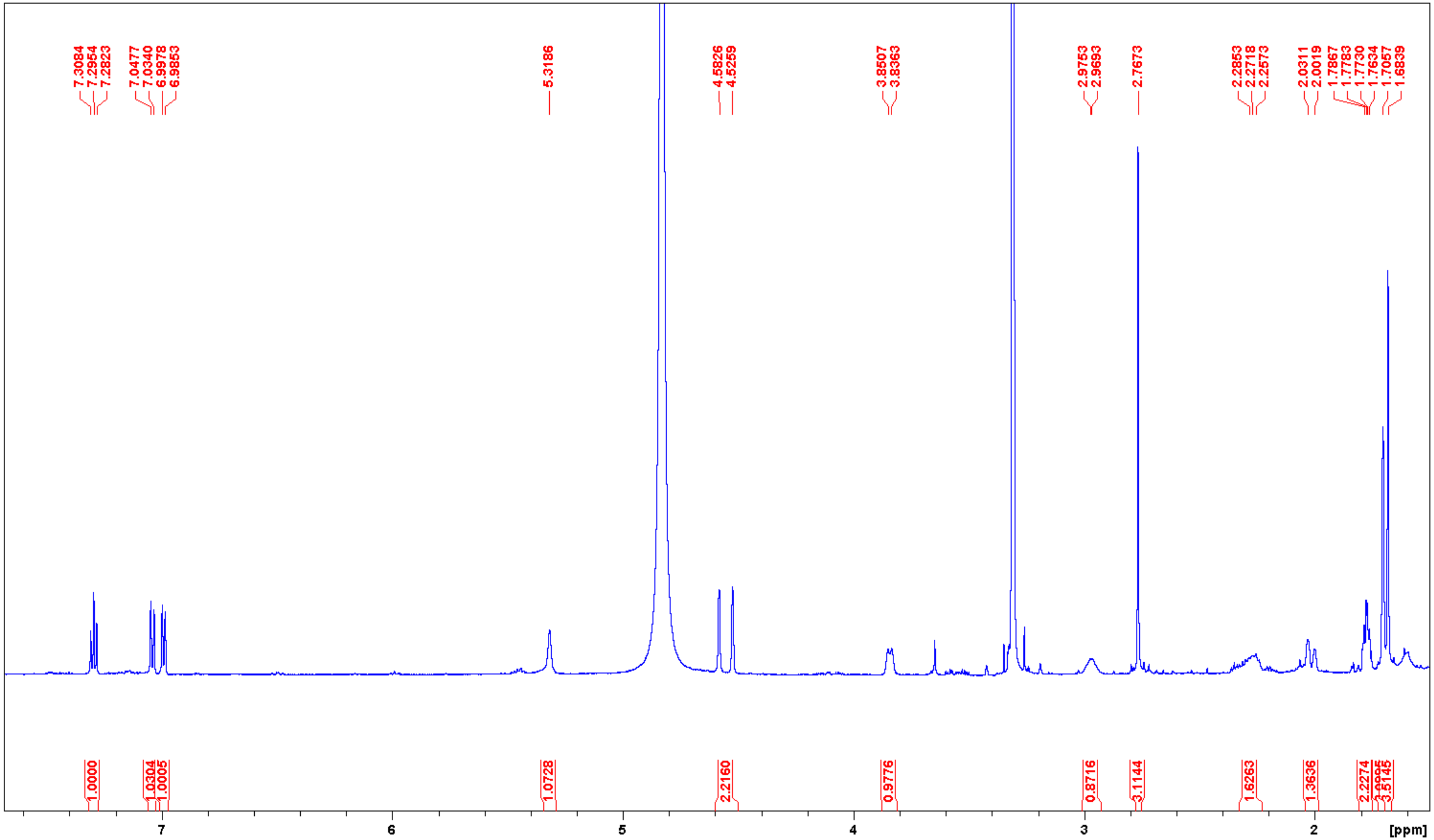

Figure S57. HSQC spectrum of **10** (600 MHz, methanol-d_4).

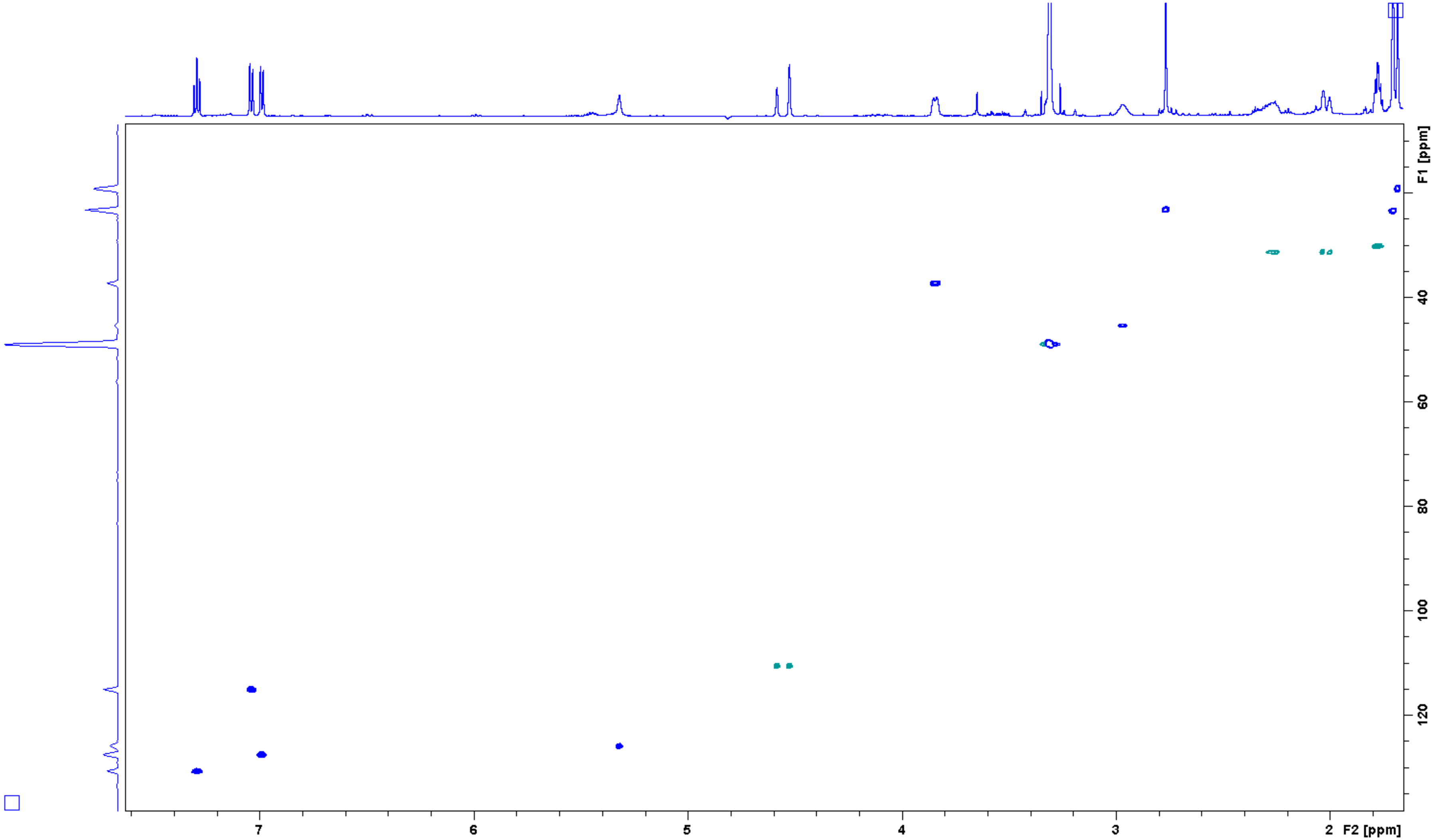

Figure S58. COSY spectrum of **10** (600 MHz, methanol-d_4).

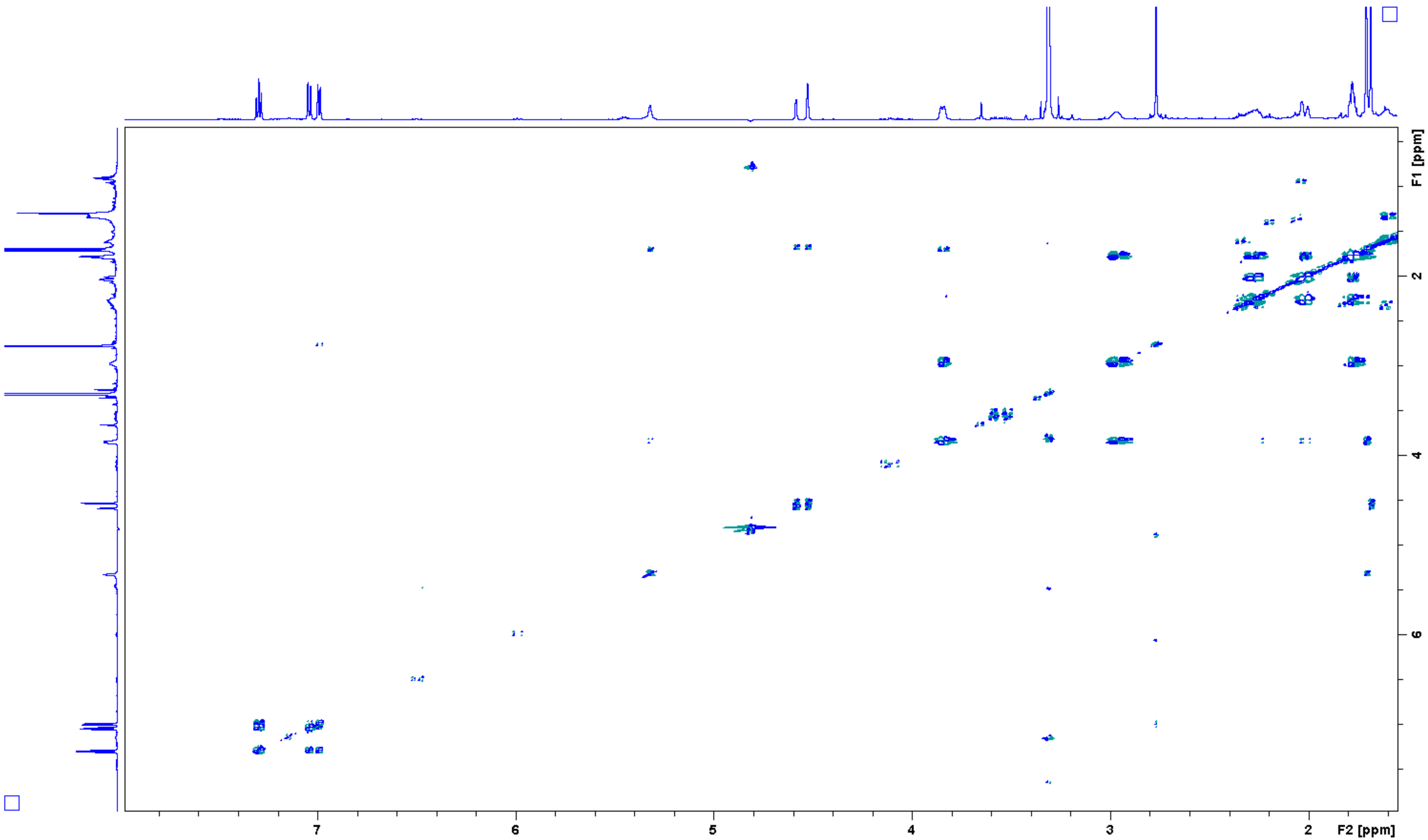

Figure S59. HMBC spectrum of **10** (600 MHz, methanol-d_4).

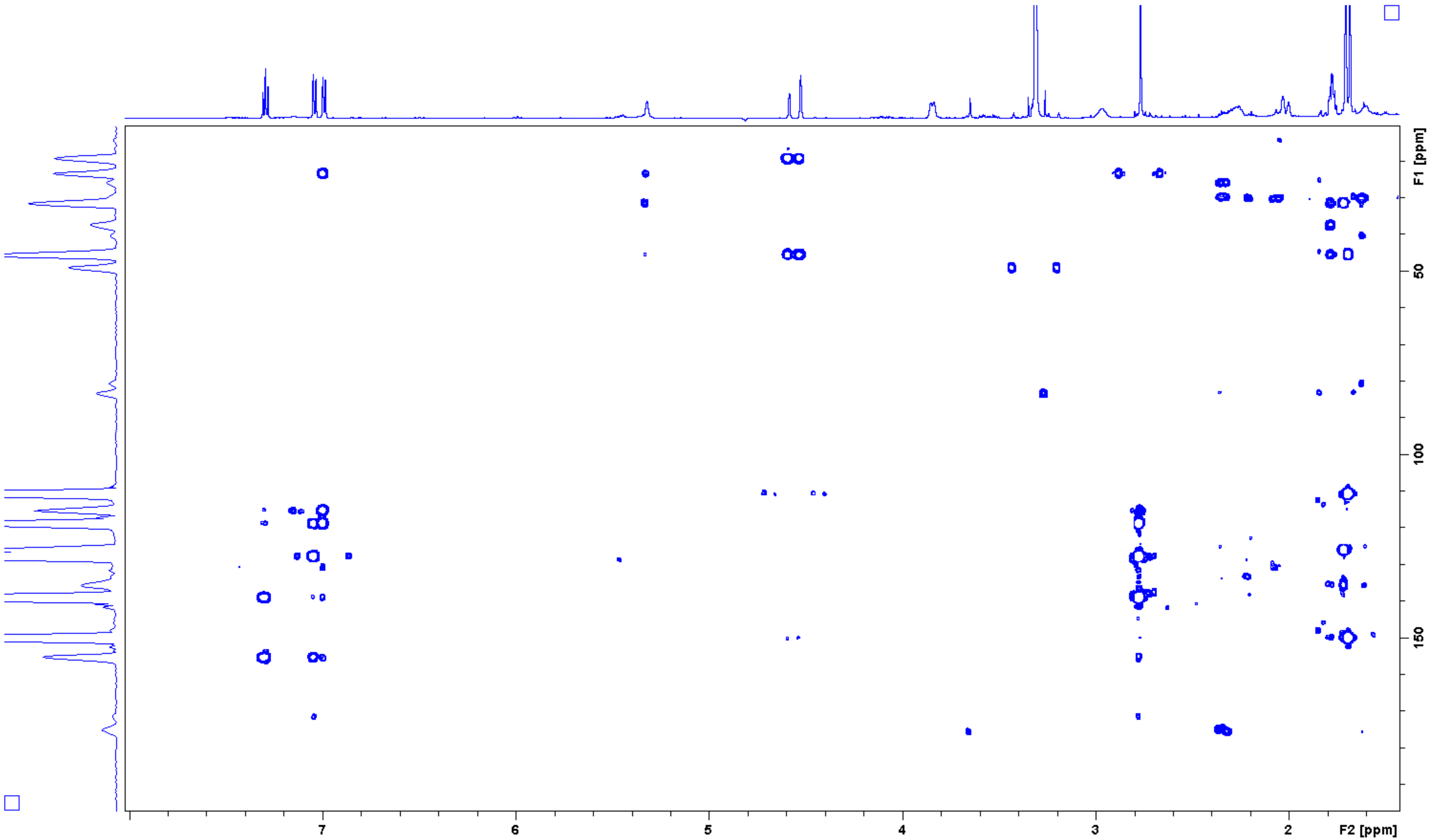

Figure S60. ROESY spectrum of **10** (600 MHz, methanol-d_4).

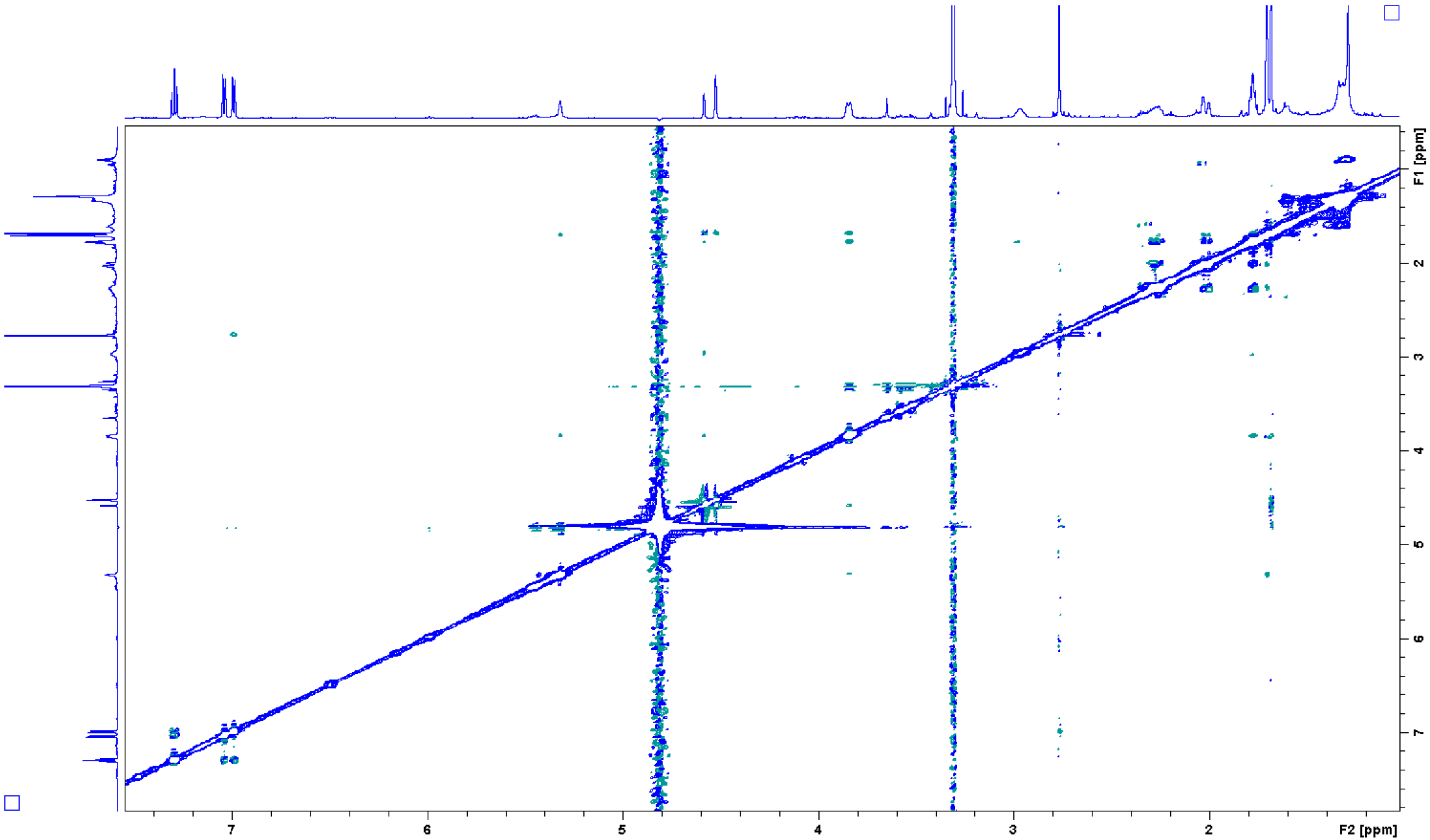

Figure S61. ^{1}H NMR spectrum of **11** (600 MHz, methanol-d_4).

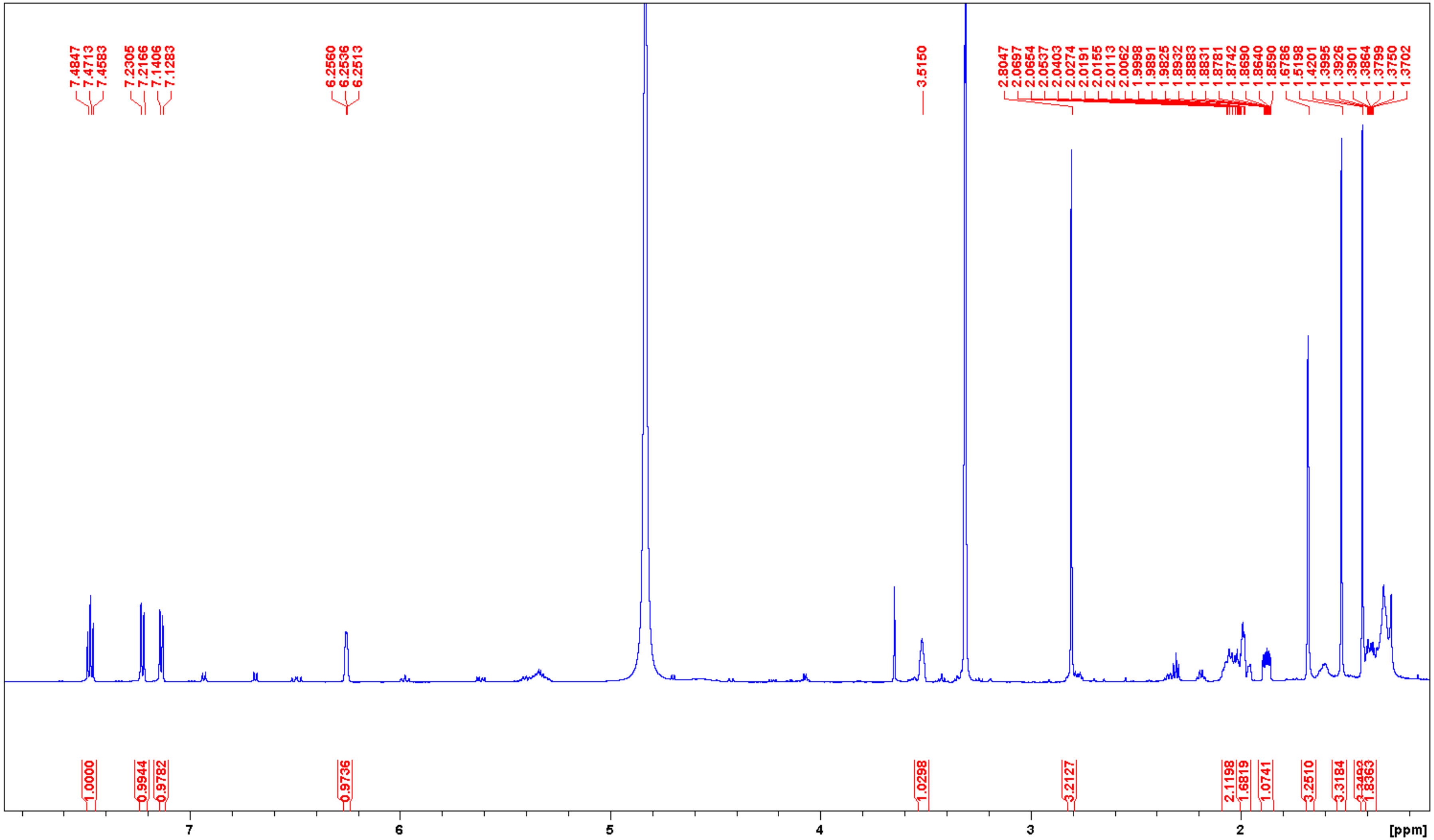

Figure S62. ^{13}C NMR spectrum of **11** (151 MHz, methanol-d_4).

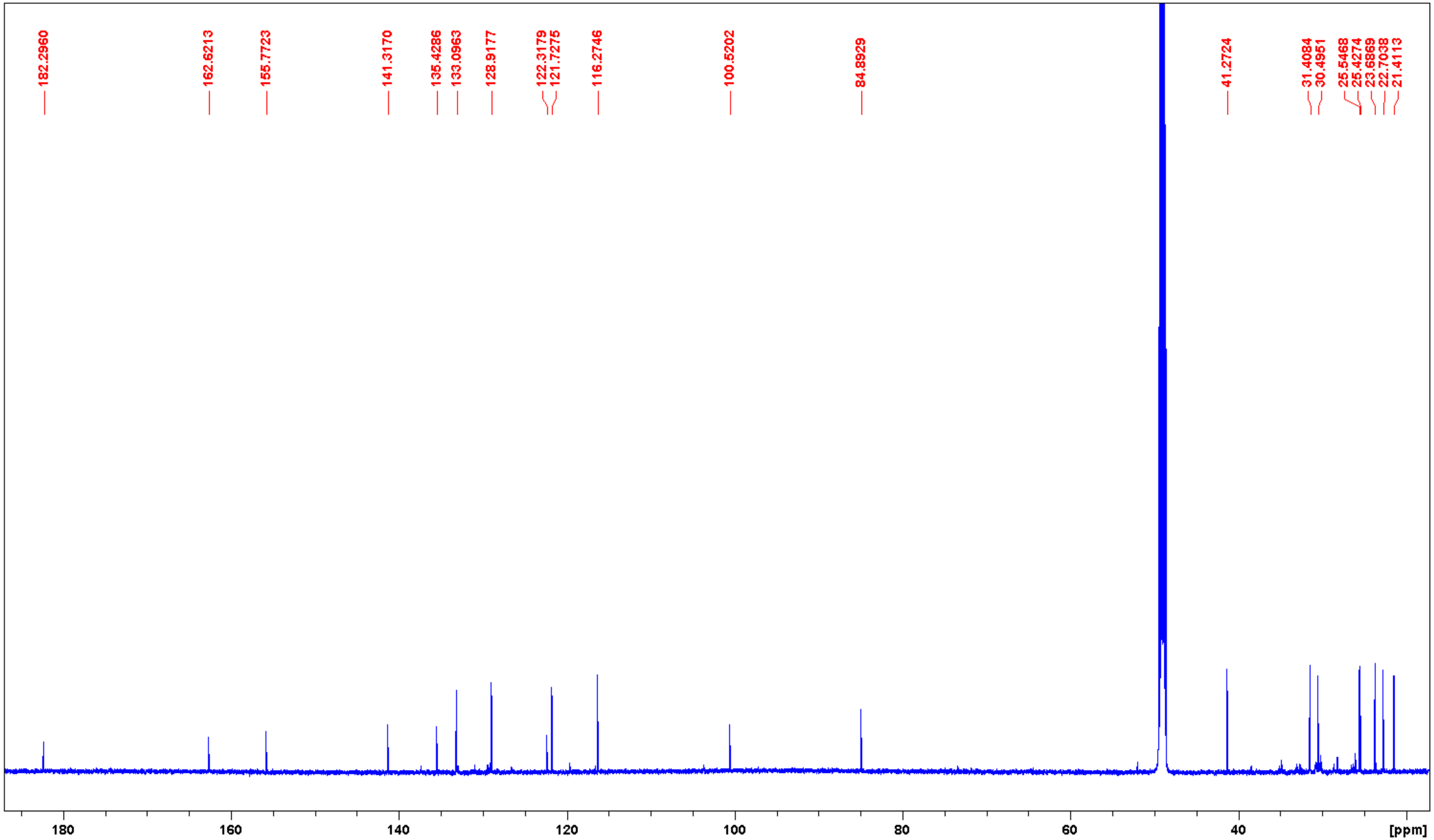

Figure S63. HSQC spectrum of **11** (600 MHz, methanol-d_4).

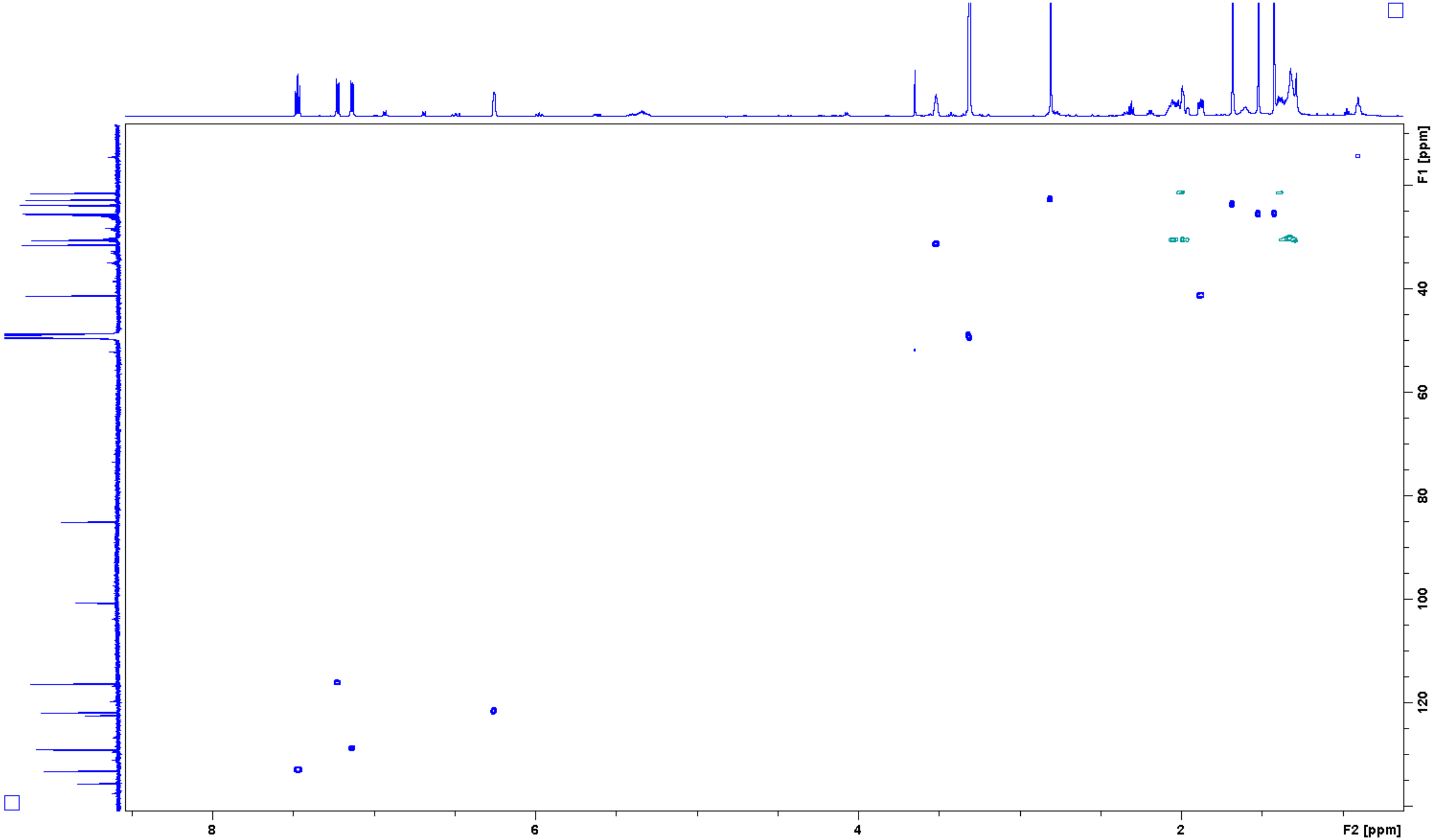

Figure S64. COSY spectrum of **11** (600 MHz, methanol-d_4).

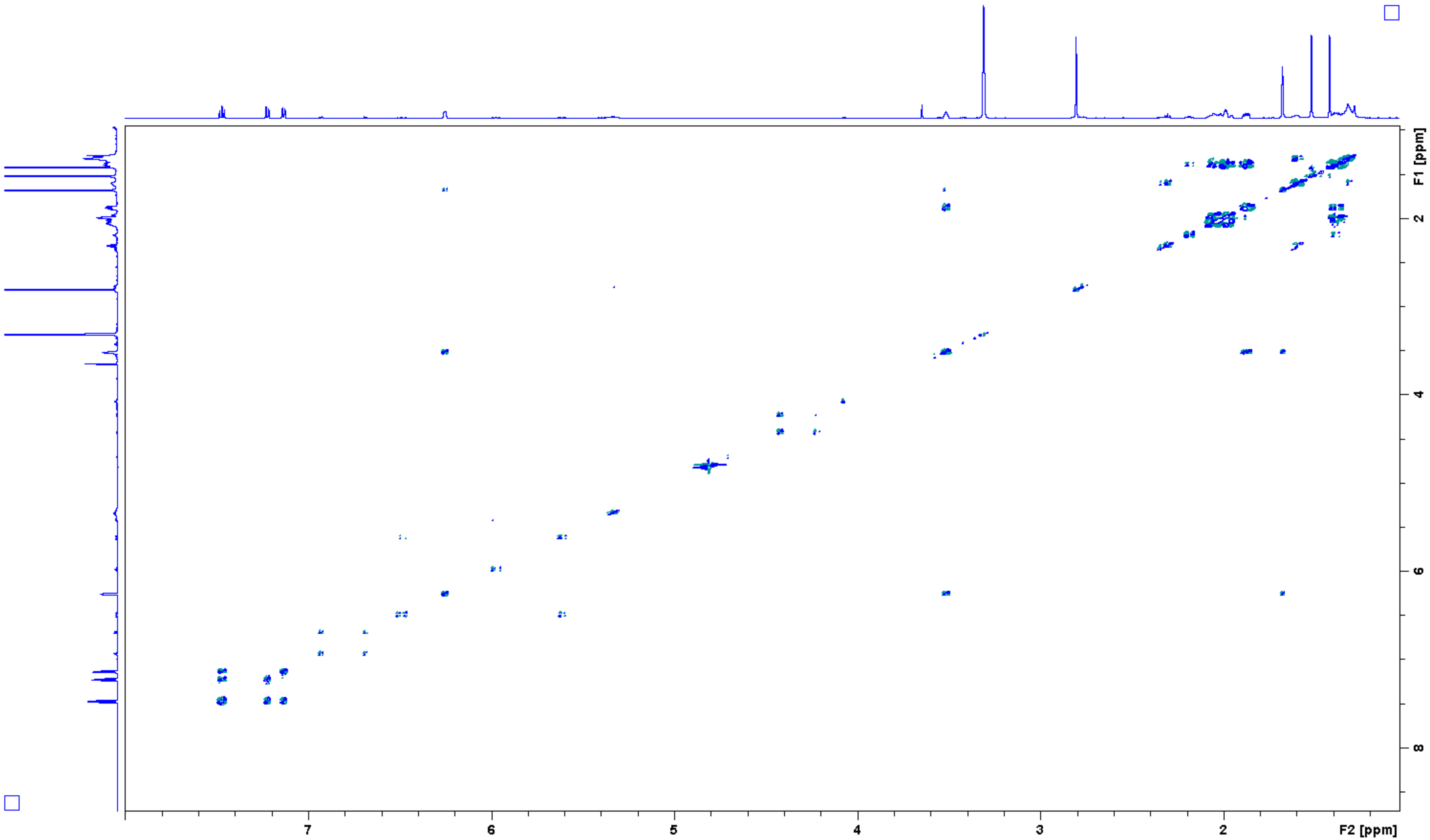

Figure S65. HMBC spectrum of **11** (600 MHz, methanol-d_4).

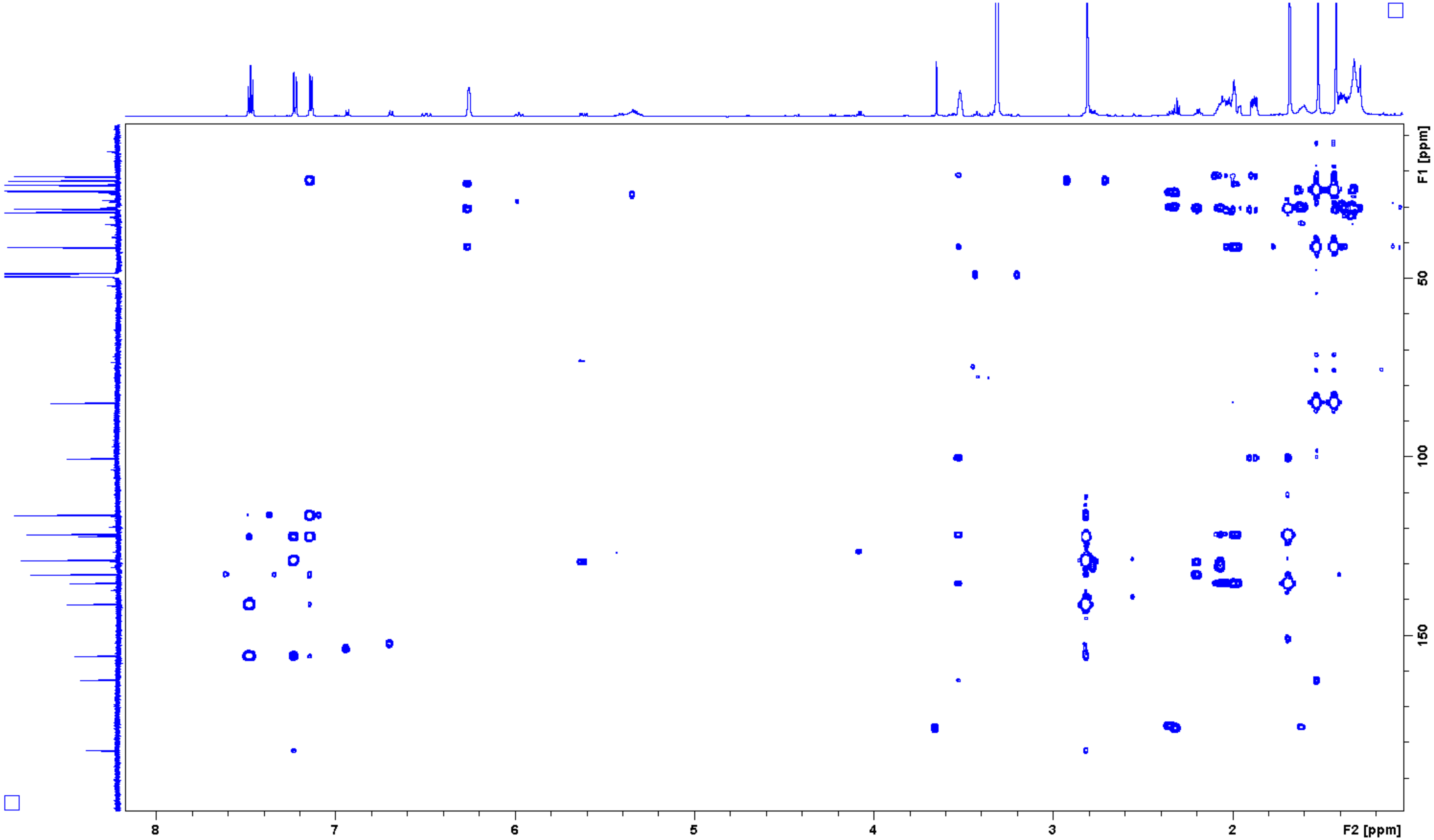

Figure S66. ROESY spectrum of **11** (600 MHz, methanol-d_4).

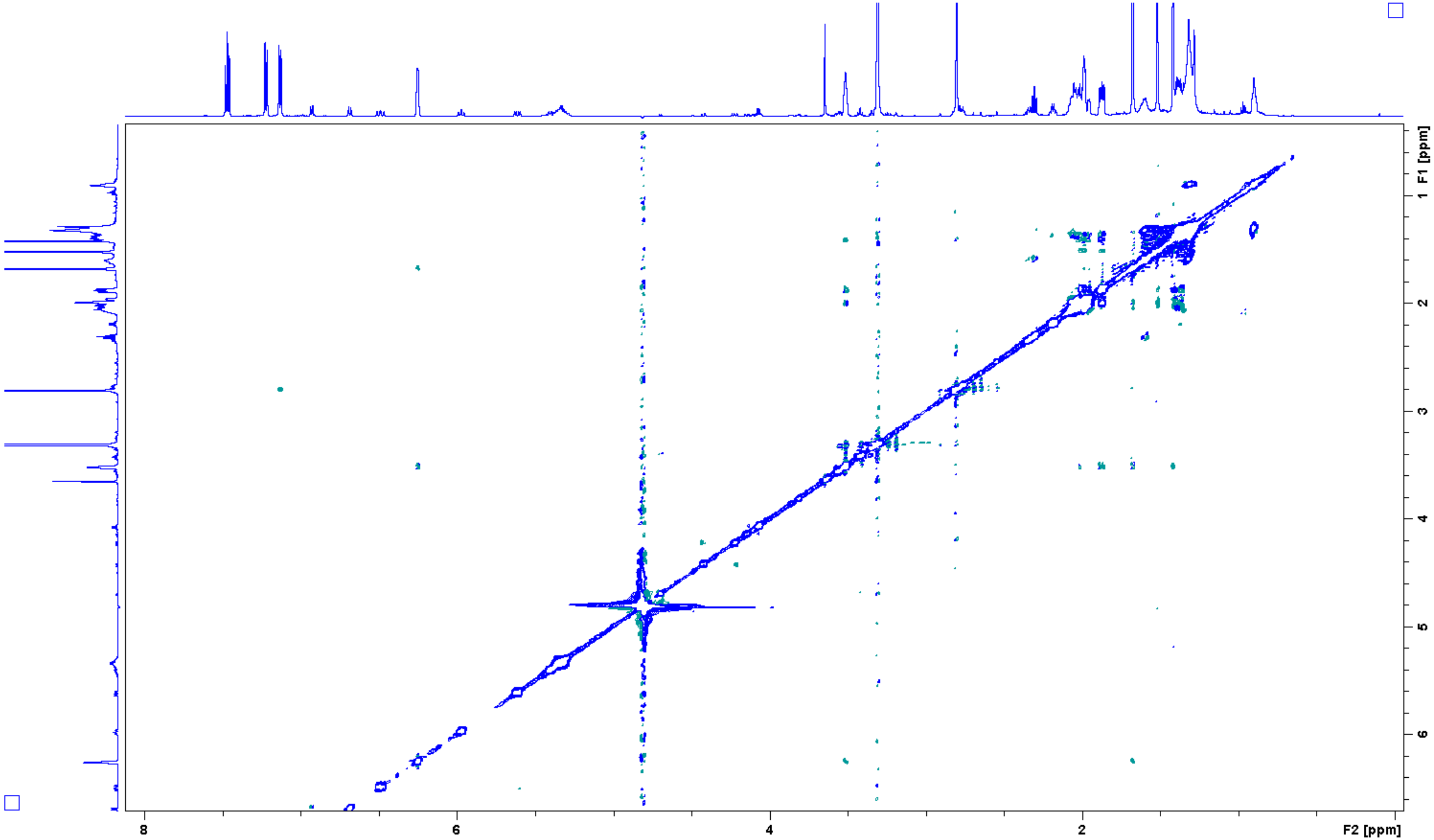

Figure S67. ^{1}H NMR spectrum of **12** (600 MHz, methanol-d_4).

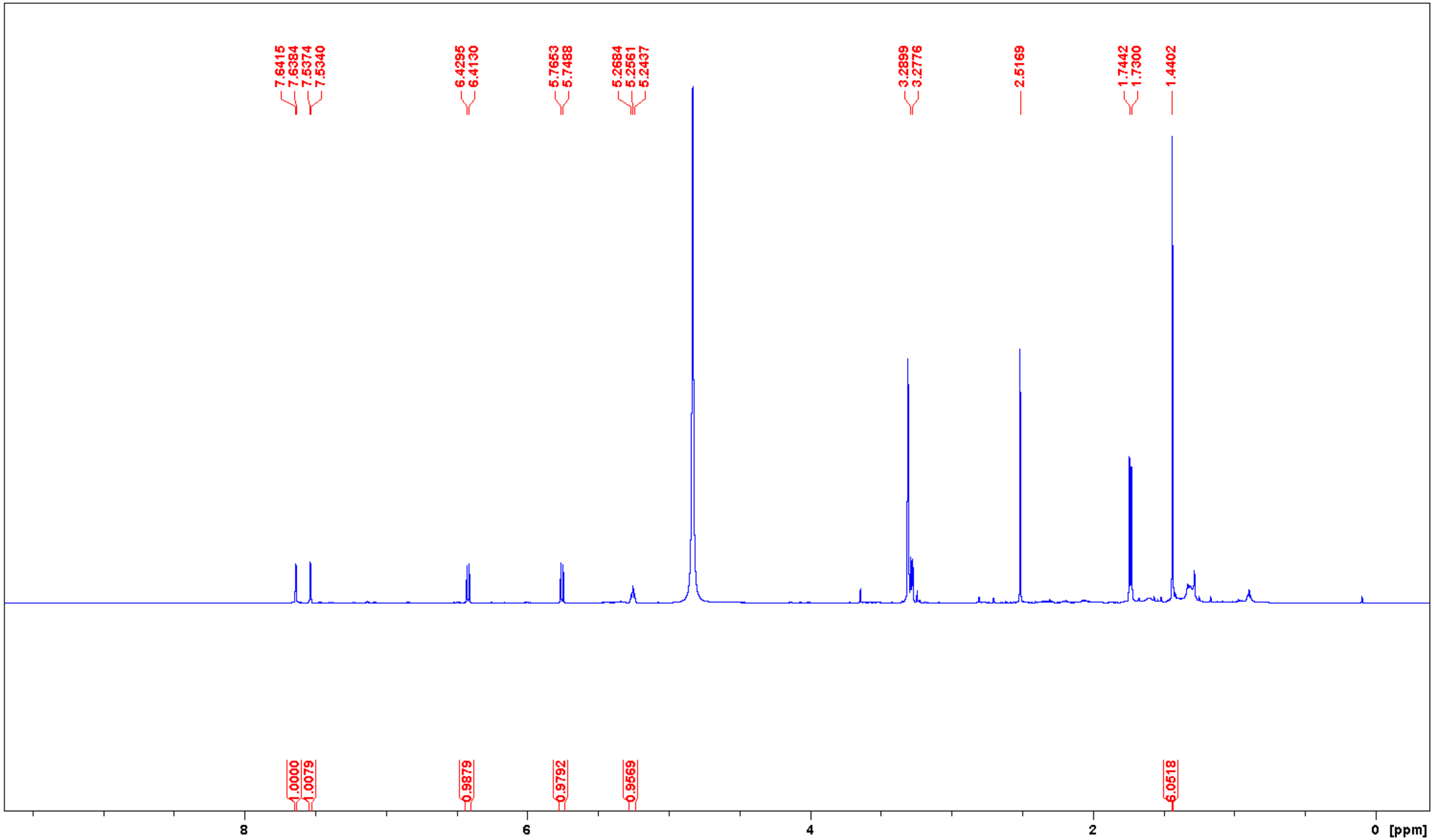

Figure S68. HSQC spectrum of **12** (600 MHz, methanol-d_4).

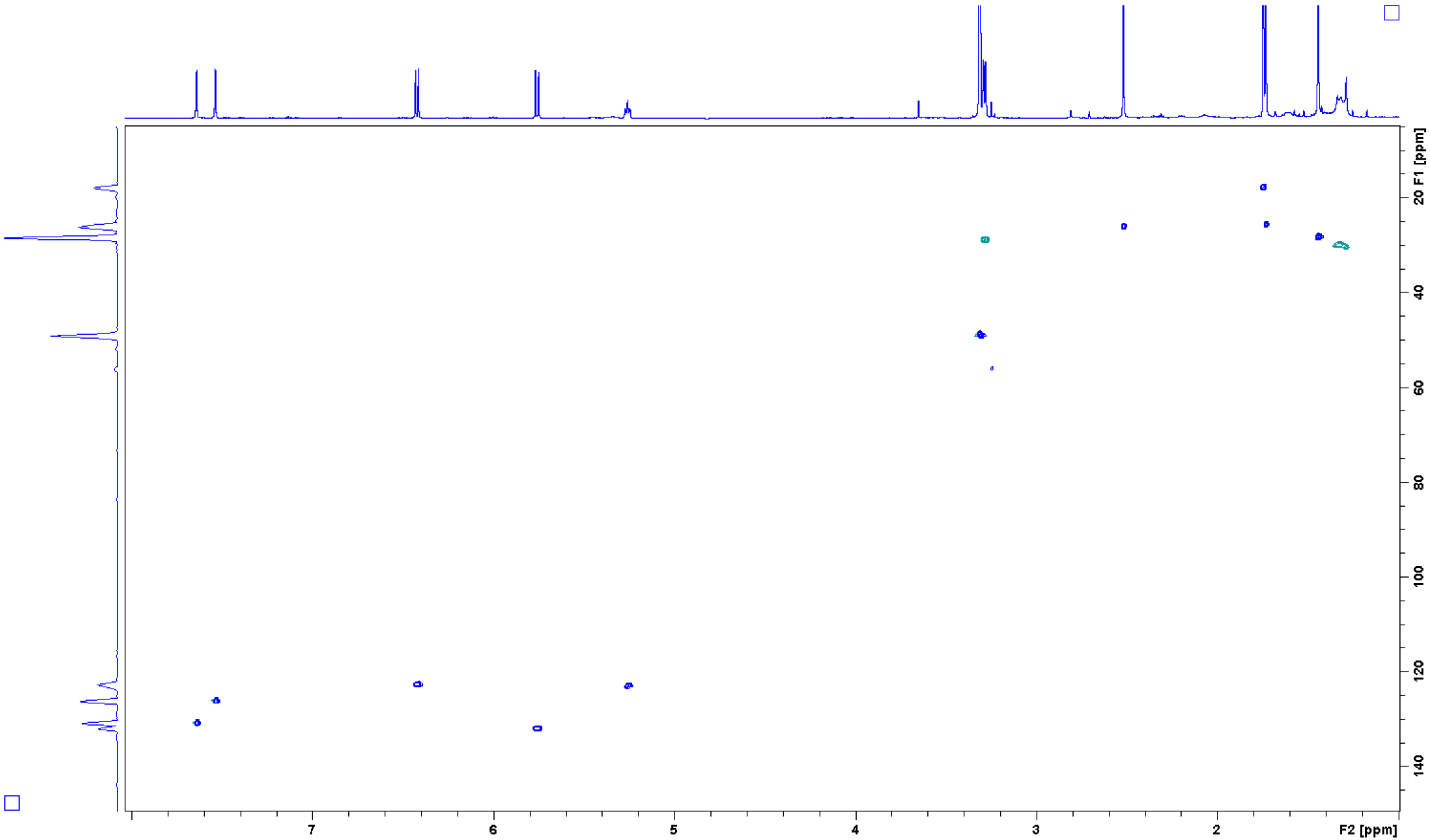

Figure S69. COSY spectrum of **12** (600 MHz, methanol-d_4).

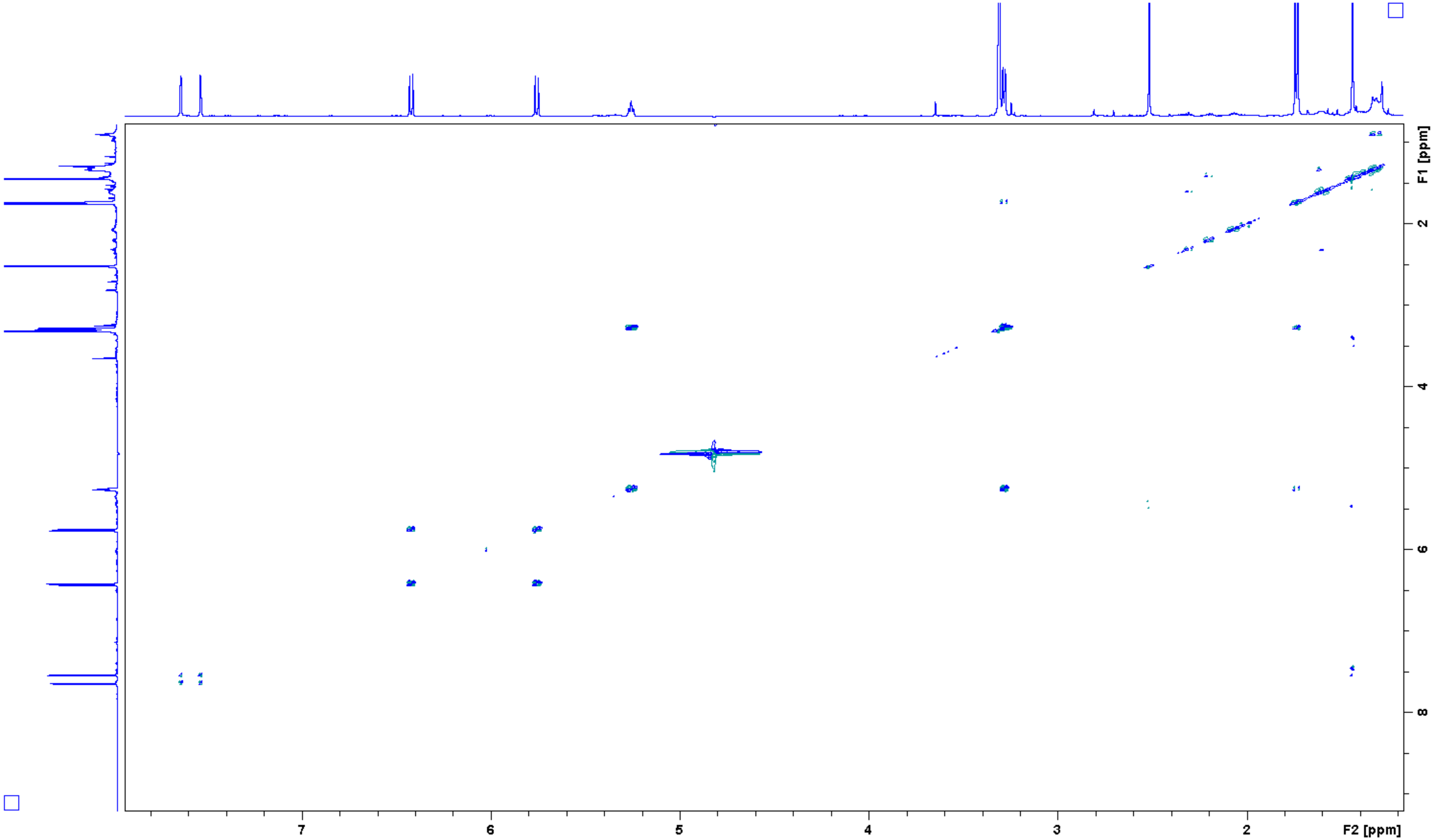

Figure S70. HMBC spectrum of **12** (600 MHz, methanol-d_4).

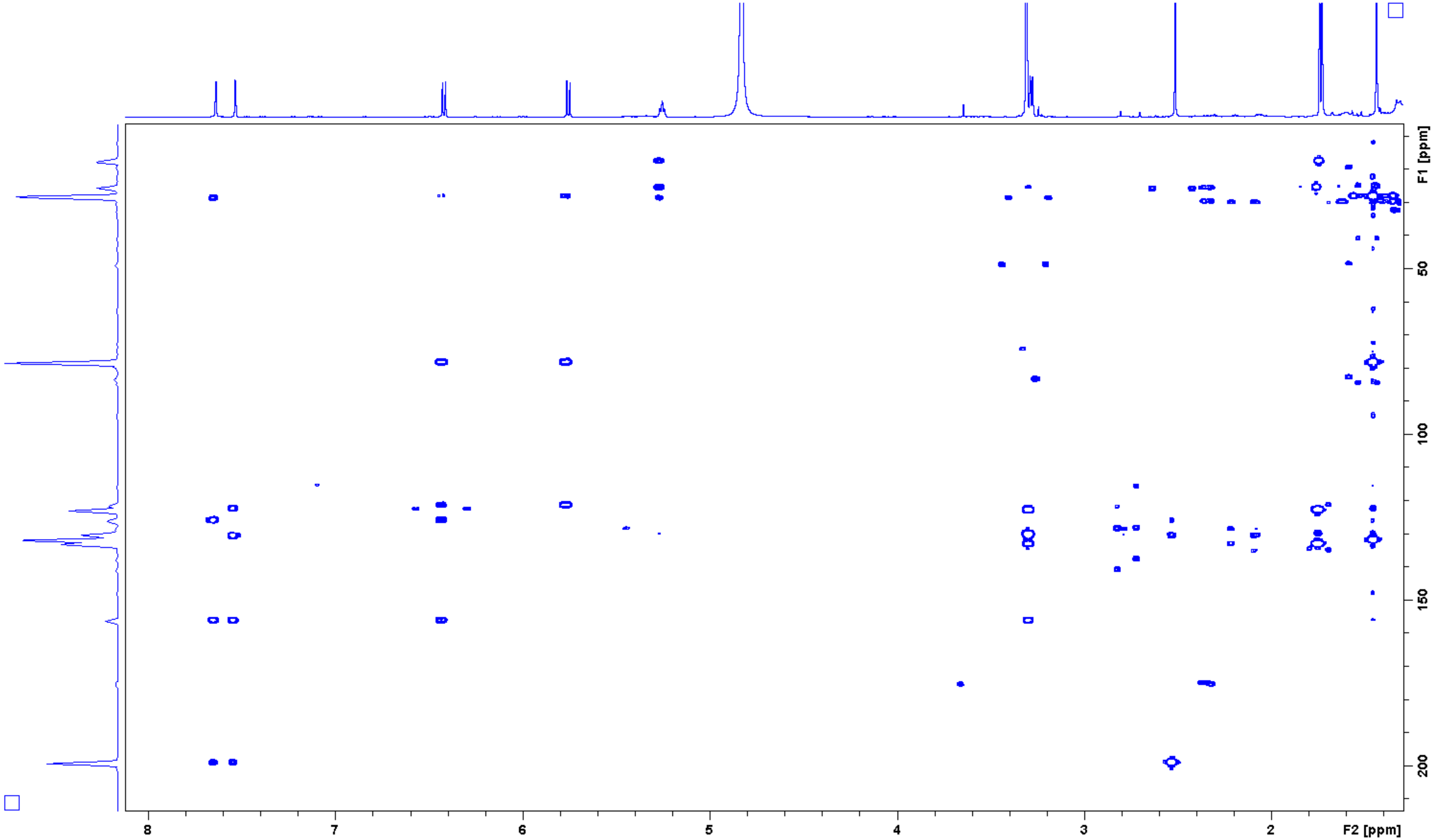

Figure S71. ROESY spectrum of **12** (600 MHz, methanol-d_4).

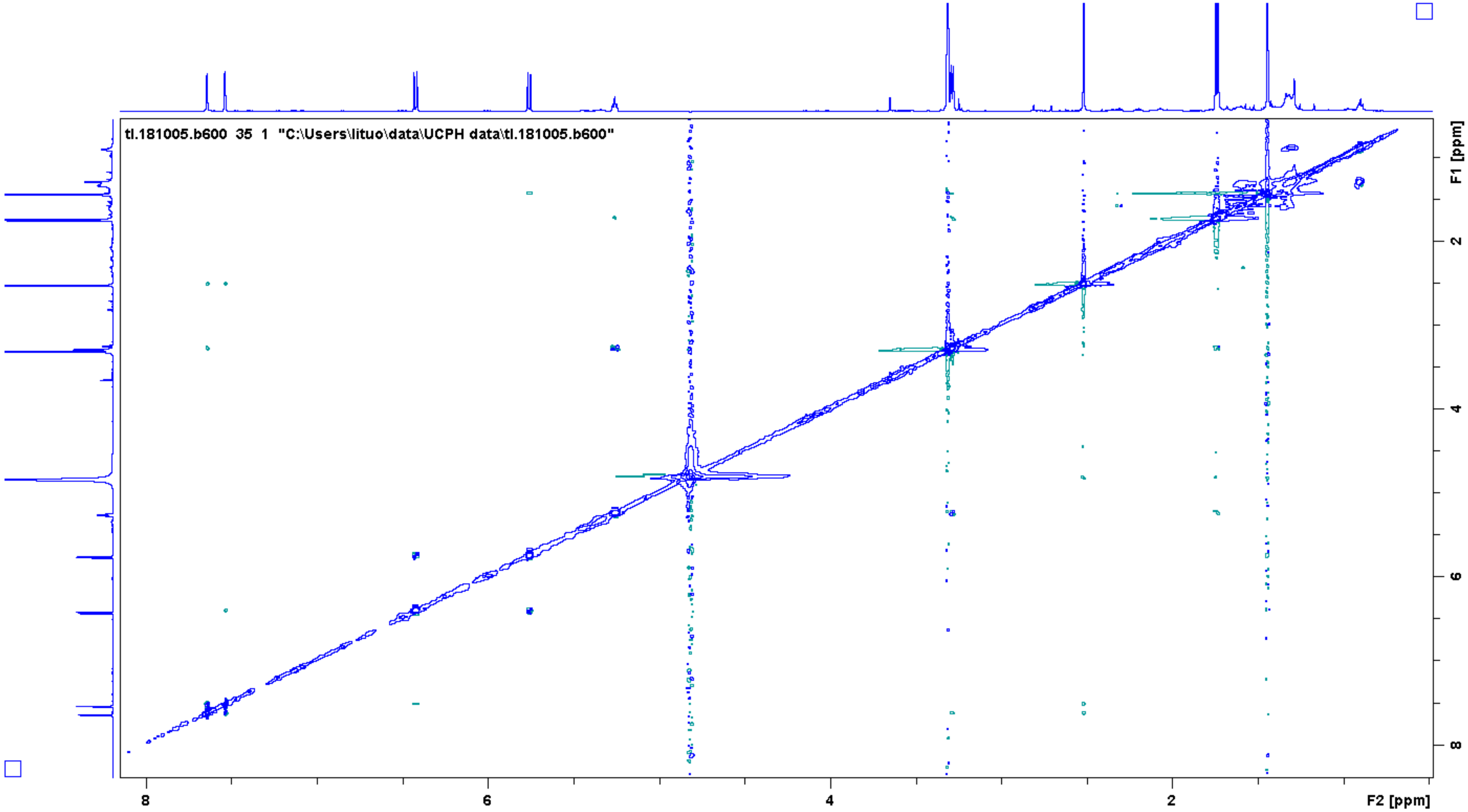

Figure S72. ^{1}H NMR spectrum of **14** (600 MHz, methanol-d_4).

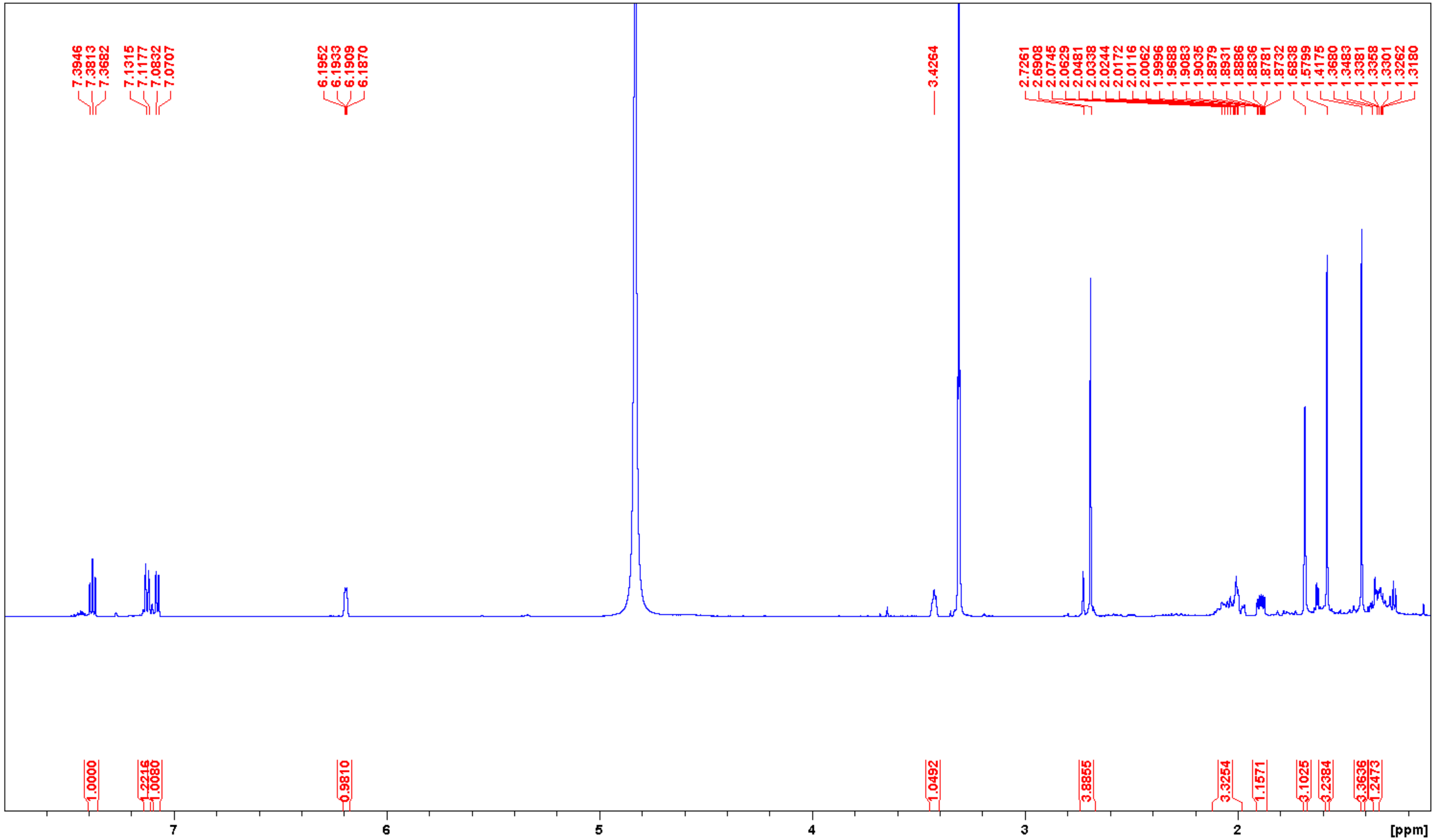

Figure S73. ^{13}C NMR spectrum of **14** (151 MHz, methanol-d_4).

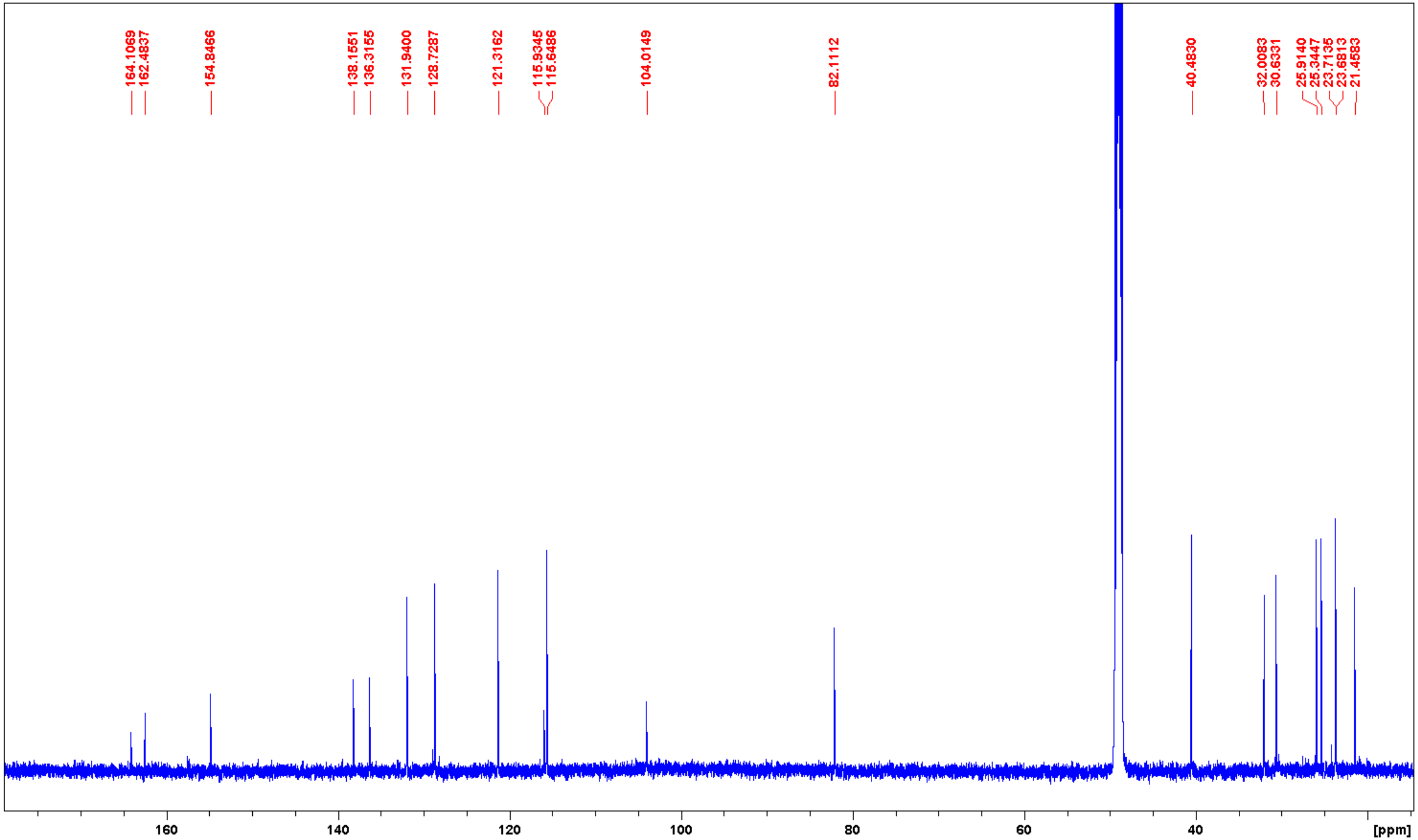

Figure S74. HSQC spectrum of **14** (600 MHz, methanol-d_4).

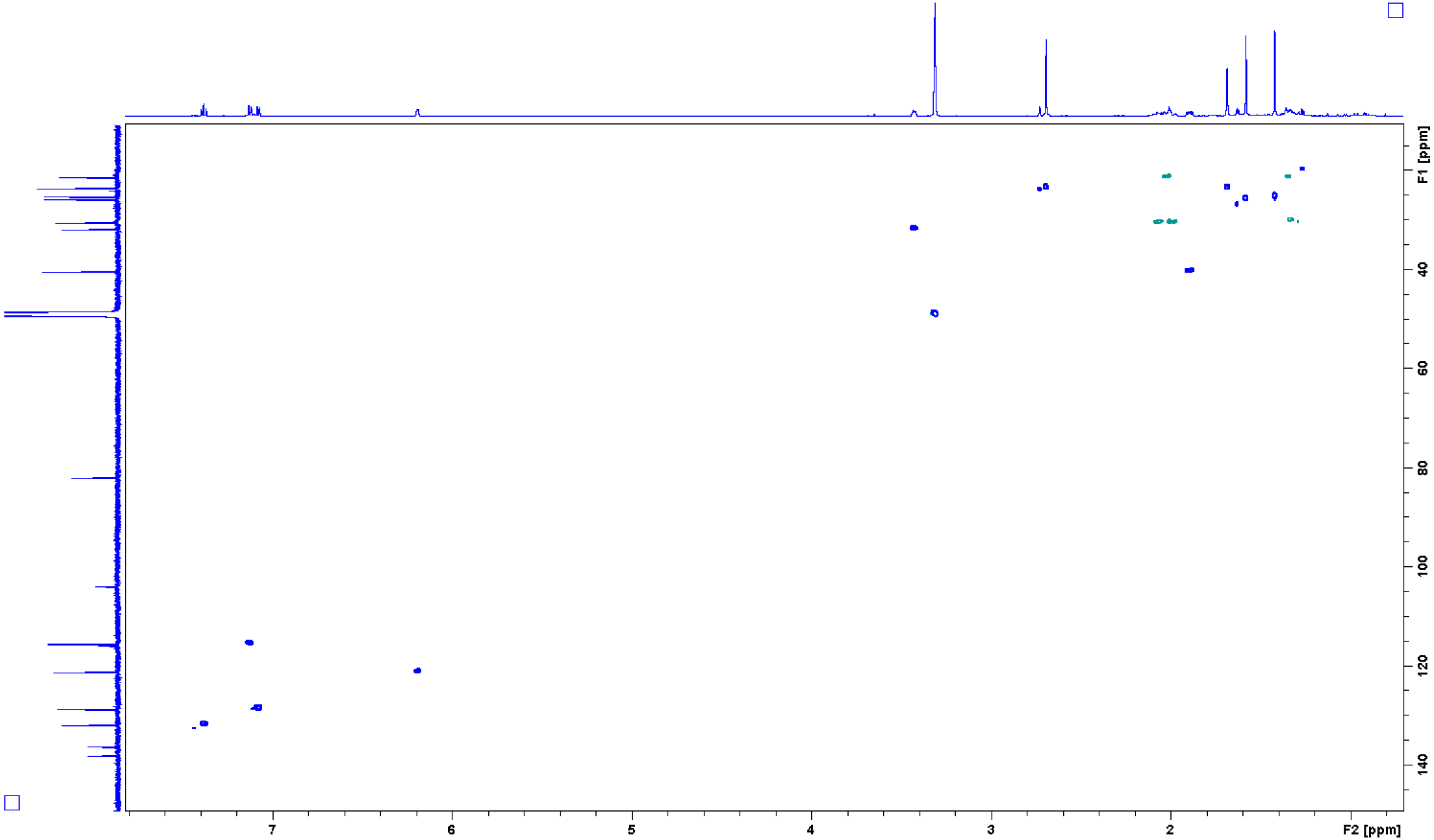

Figure S75. COSY spectrum of **14** (600 MHz, methanol-d_4).

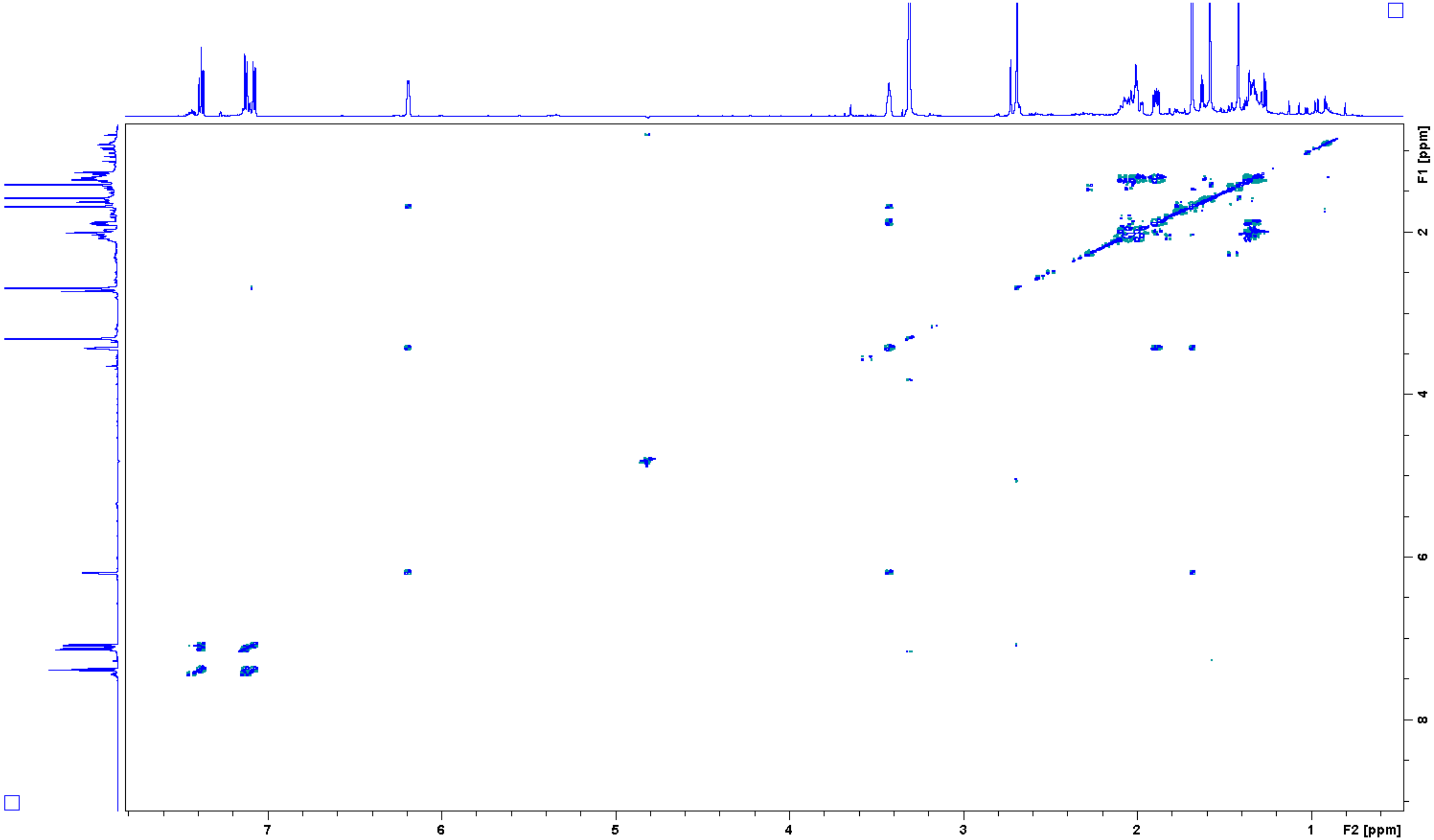

Figure S76. HMBC spectrum of **14** (600 MHz, methanol-d_4).

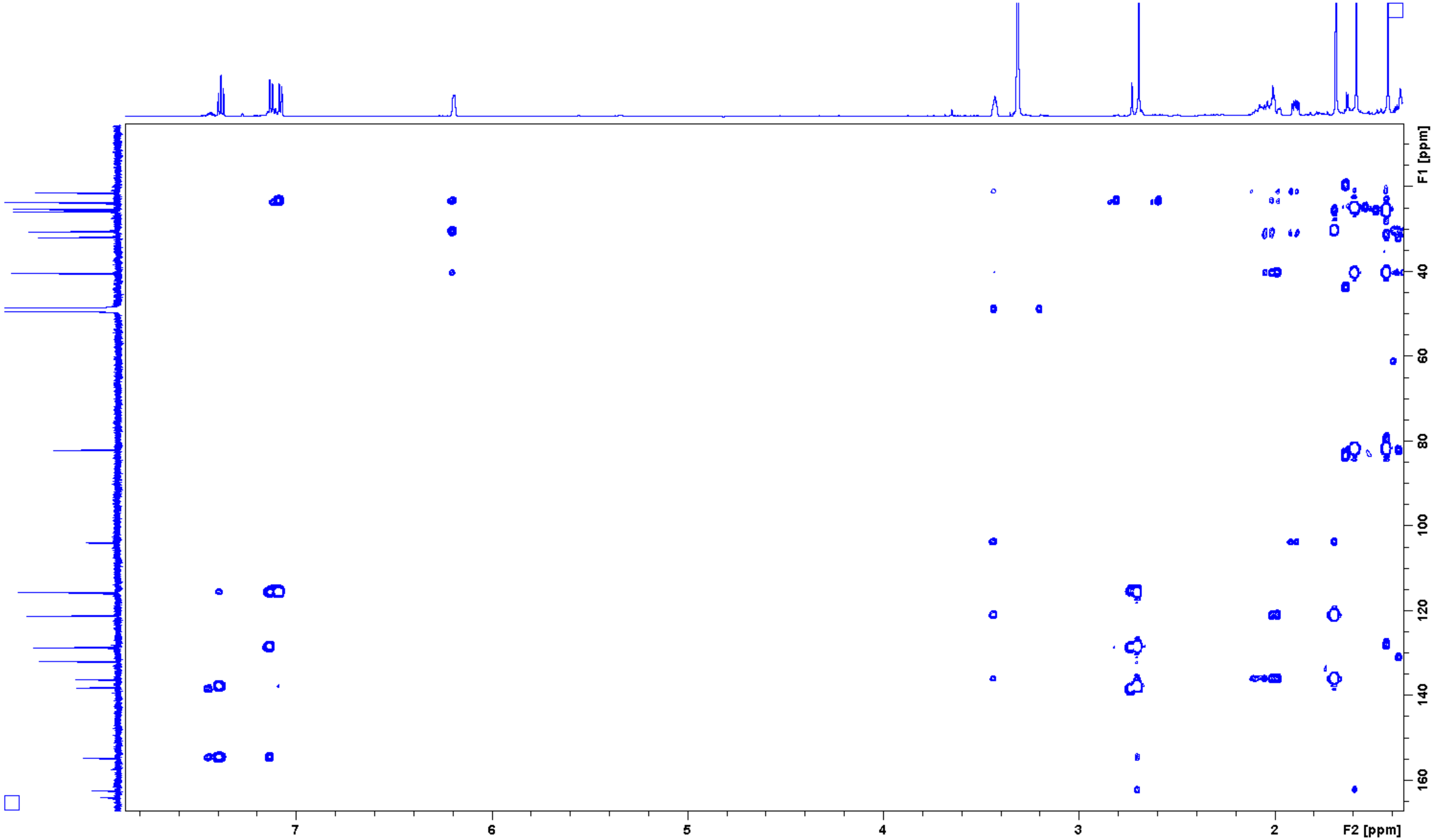

Figure S77. ROESY spectrum of **14** (600 MHz, methanol-d_4).

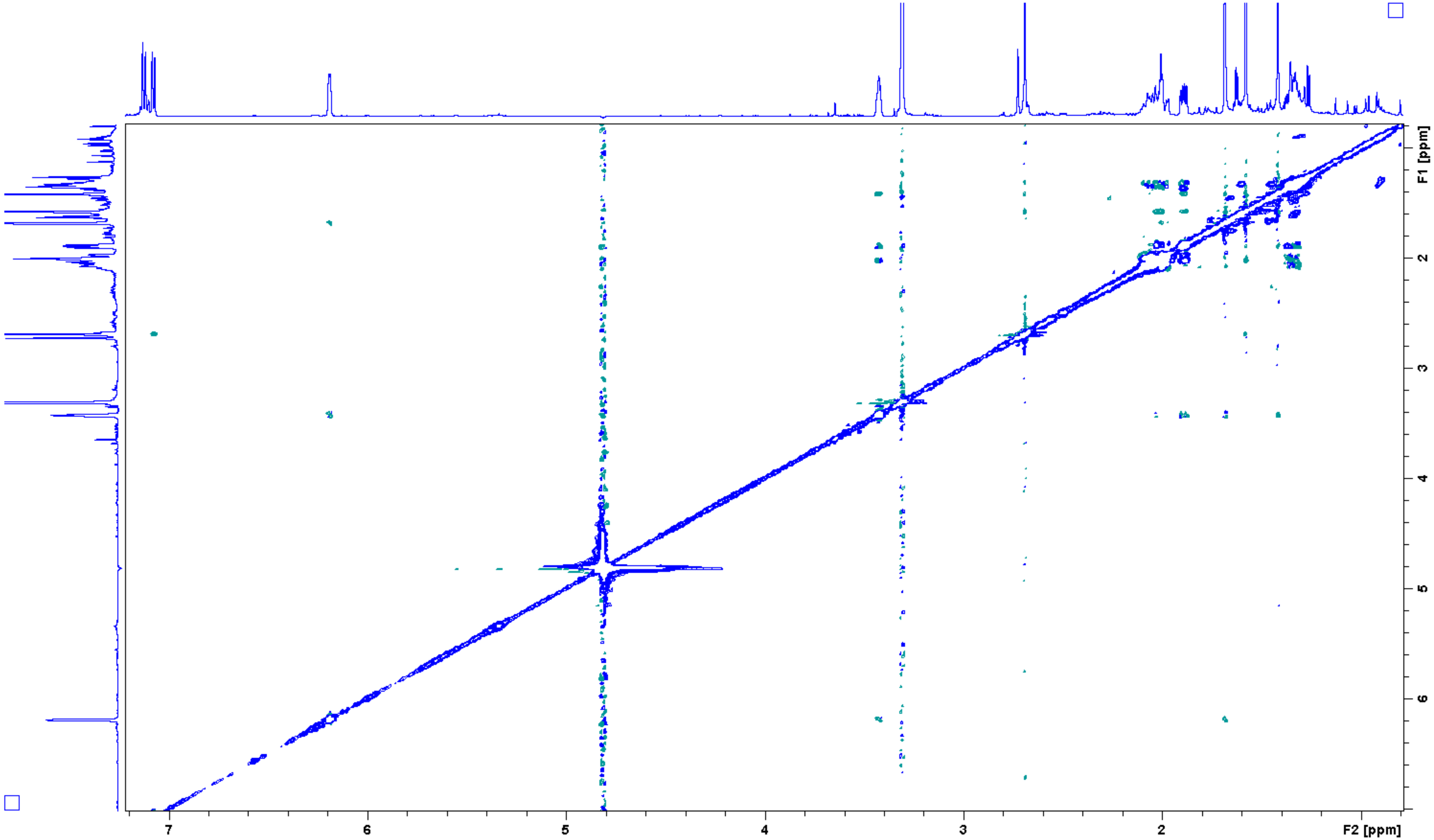

Figure S78. ^{1}H NMR spectrum of **15** (600 MHz, methanol-d_4).

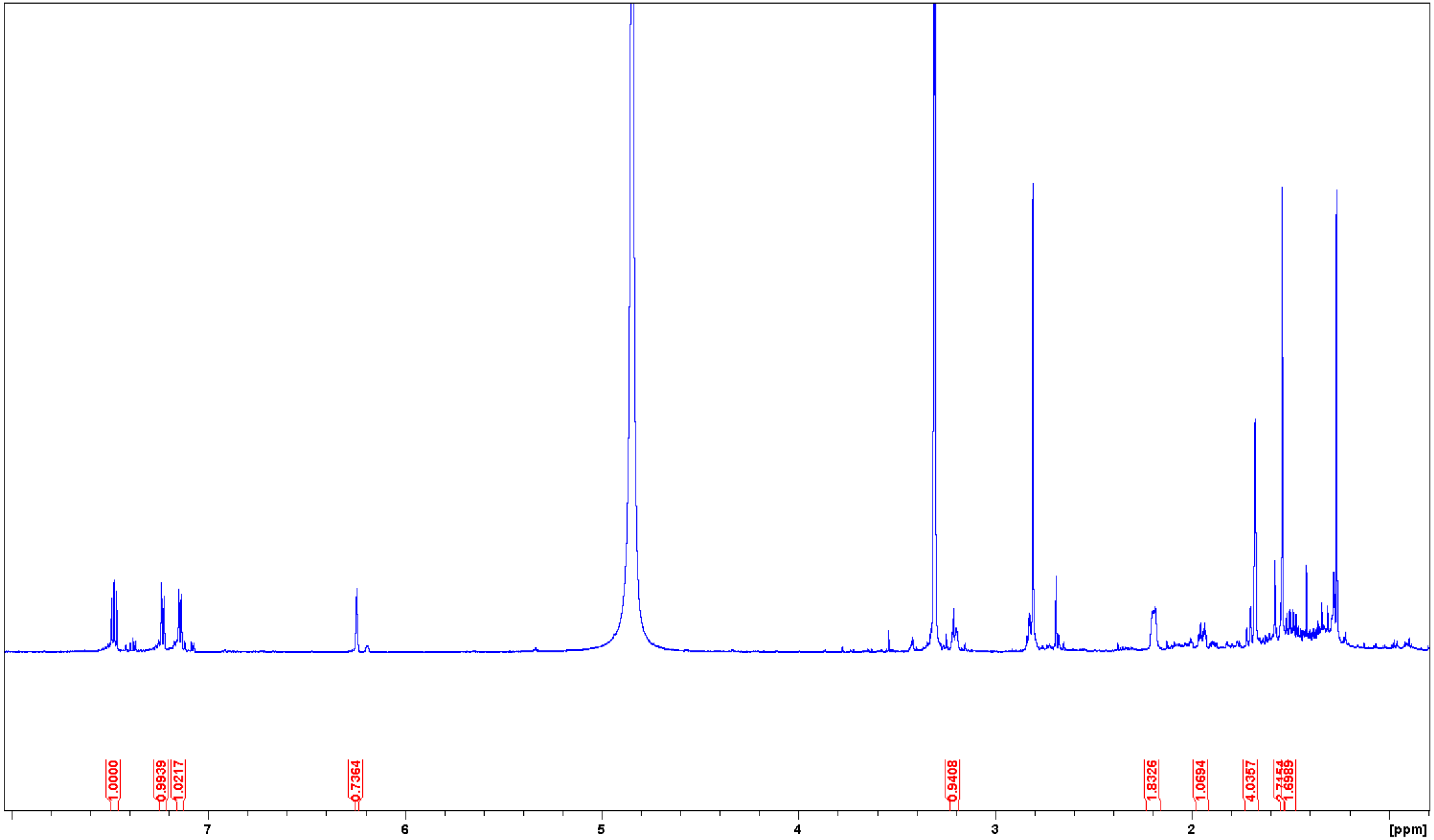

Figure S79. ^{13}C NMR spectrum of **15** (151 MHz, methanol-d_4).

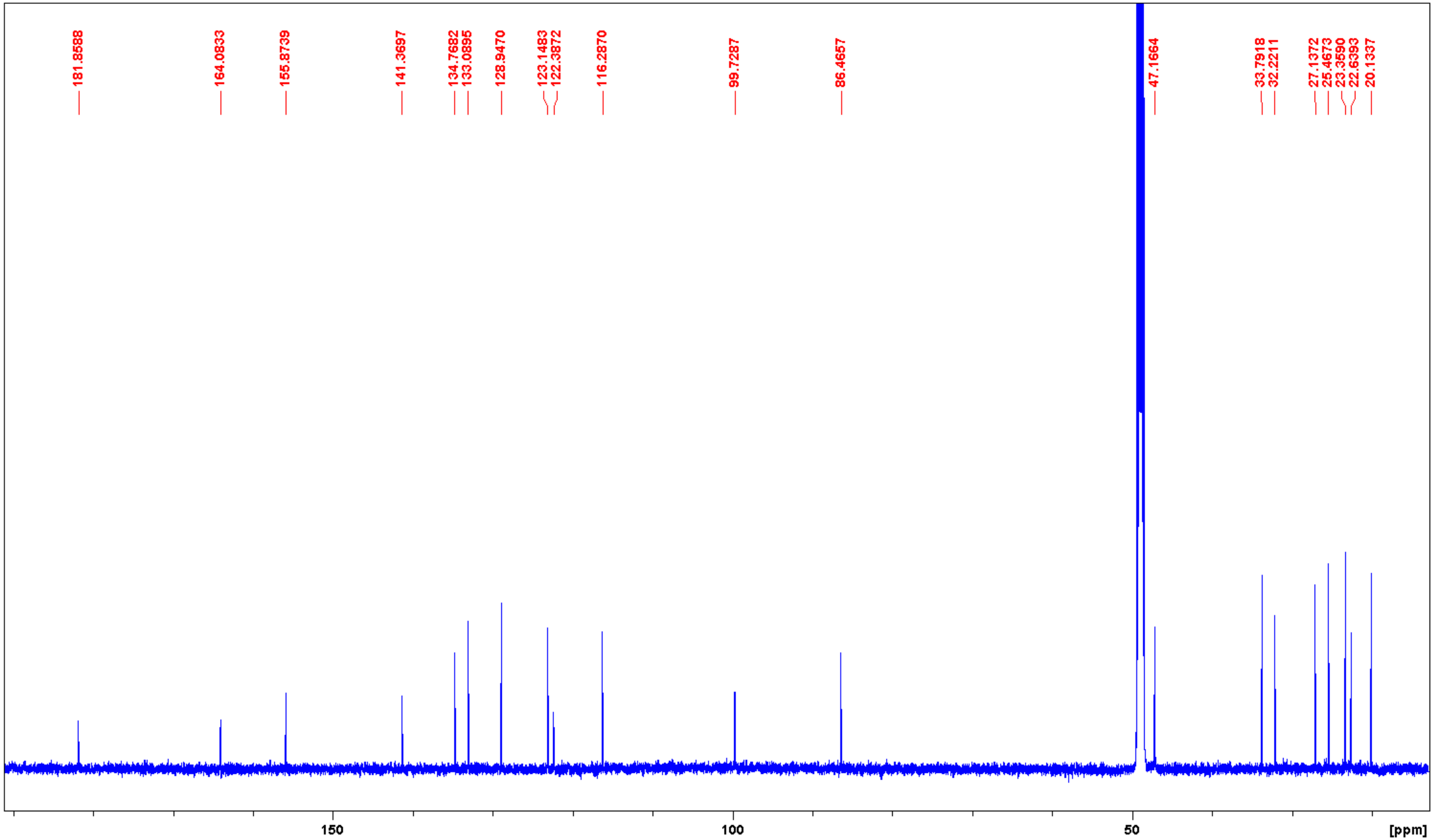

Figure S80. HSQC spectrum of **15** (600 MHz, methanol-d_4).

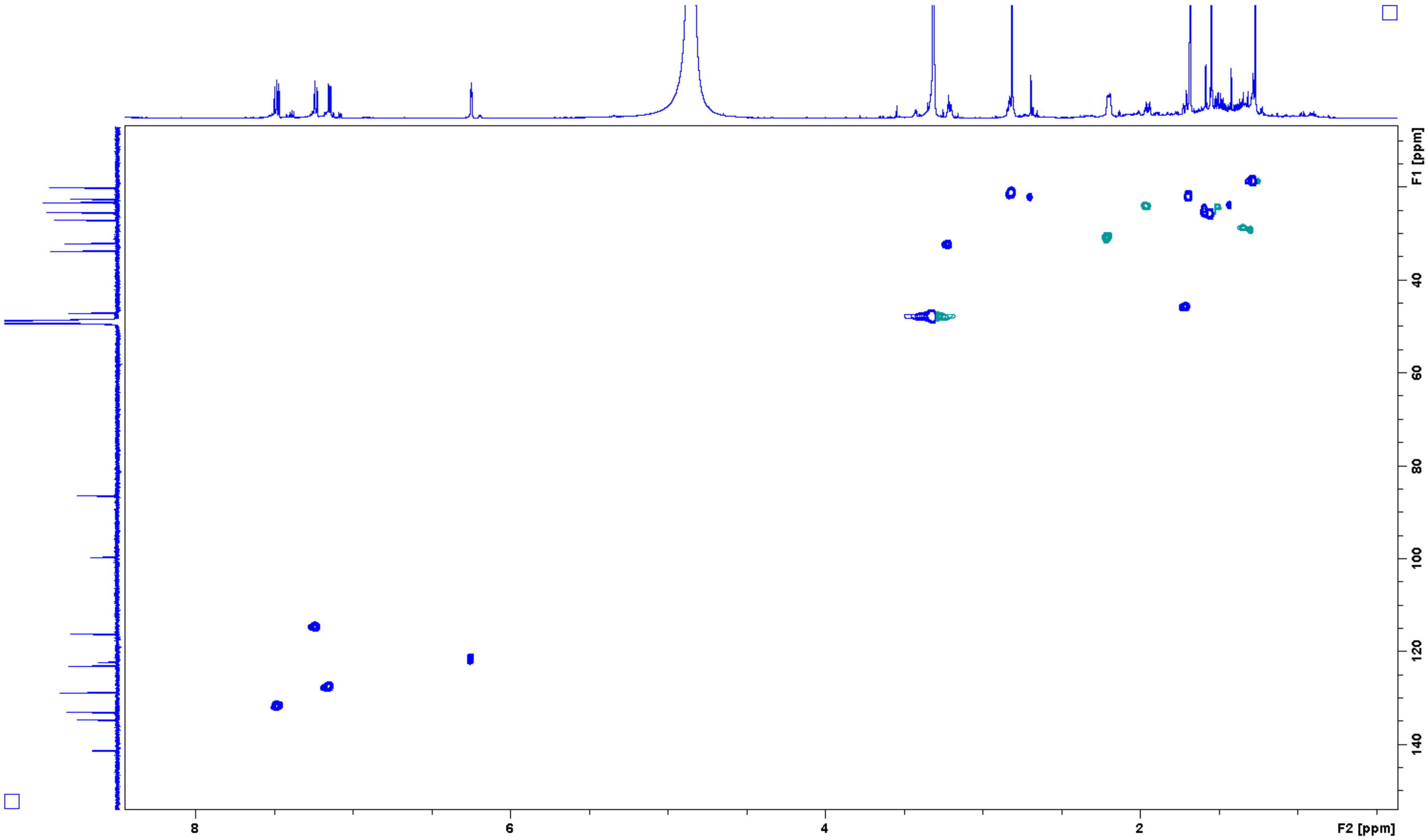

Figure S81. COSY spectrum of **15** (600 MHz, methanol-d_4).

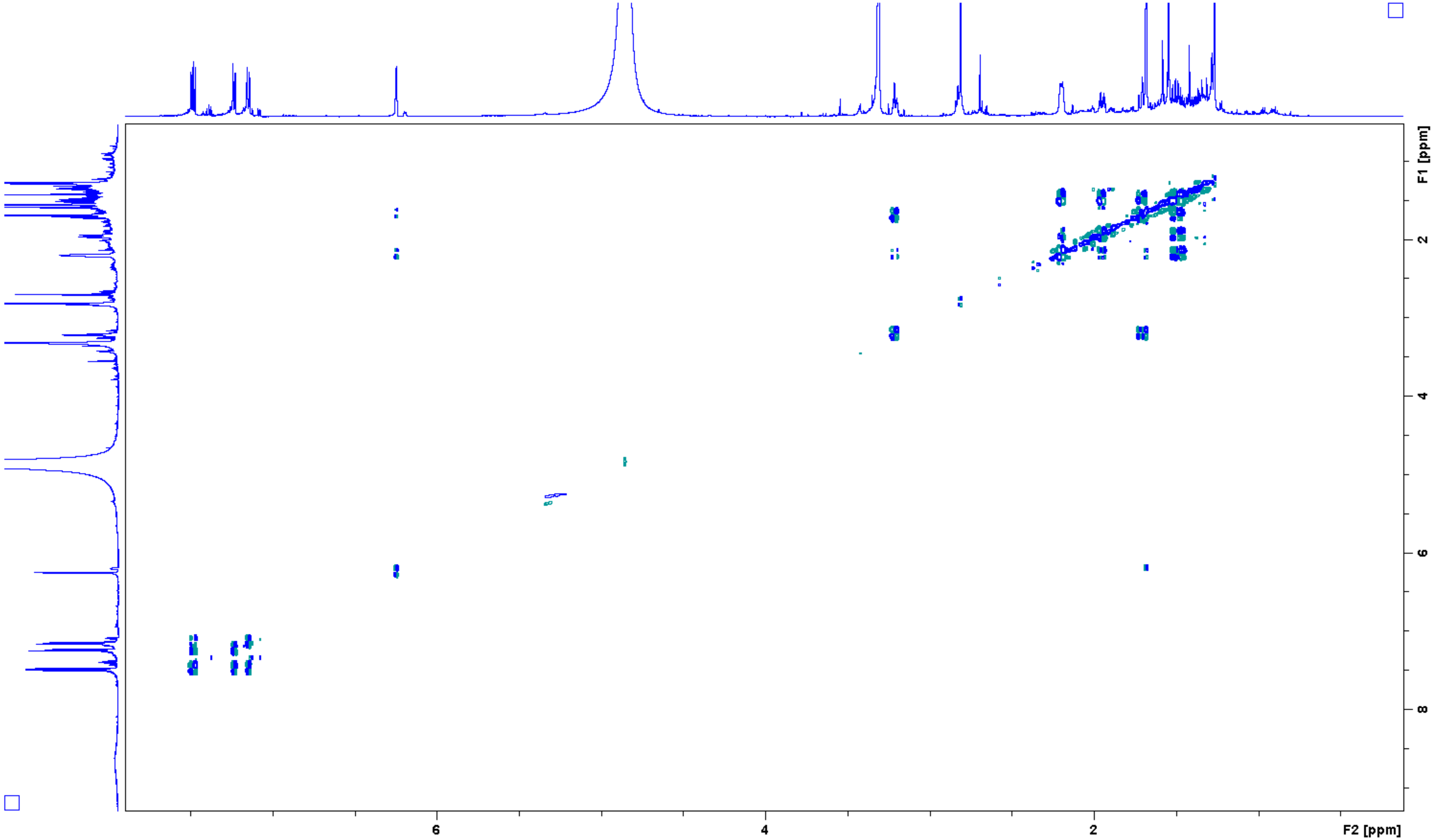

Figure S82. HMBC spectrum of **15** (600 MHz, methanol-d_4).

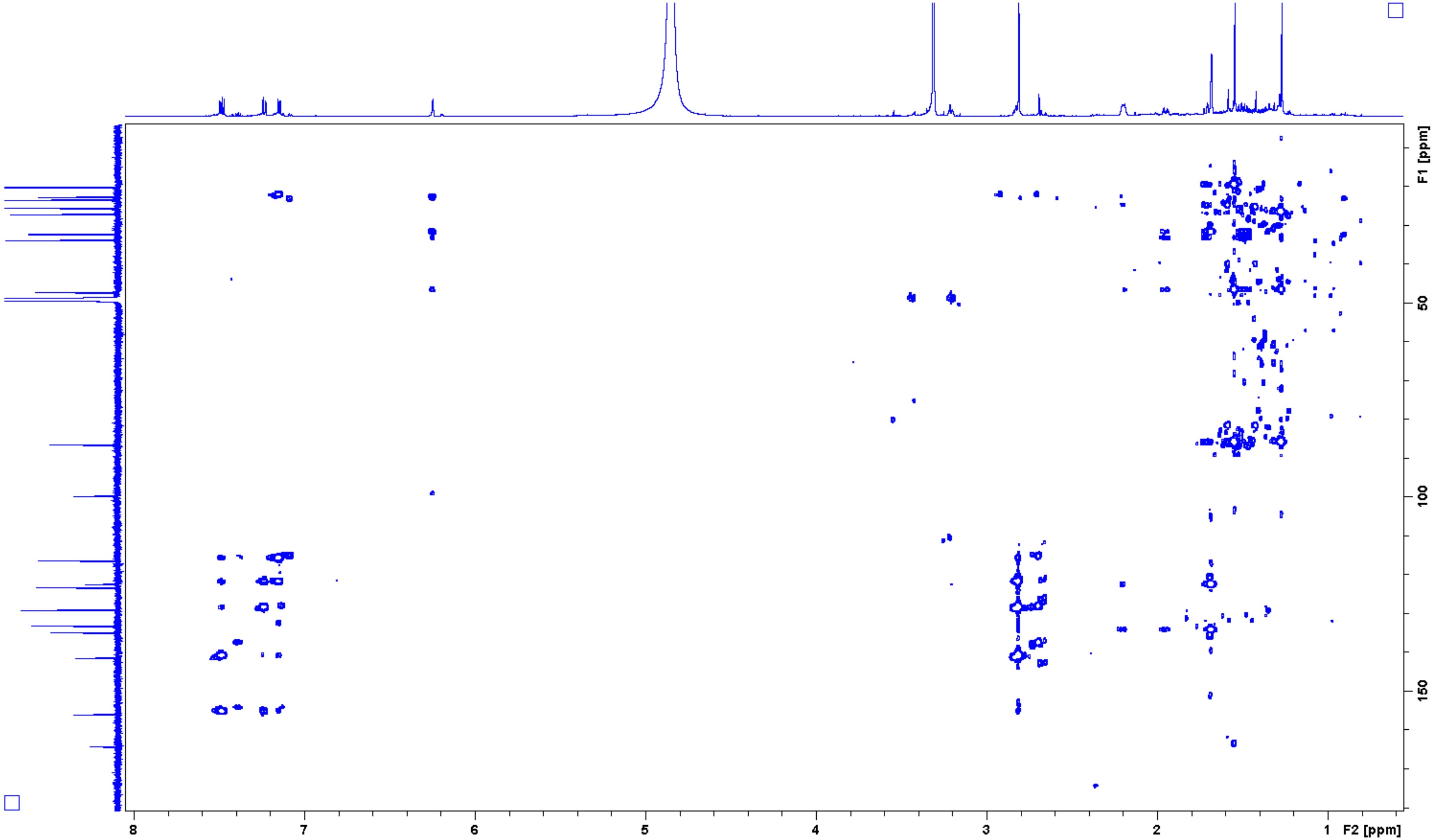

Figure S83. ROESY spectrum of **15** (600 MHz, methanol-d_4).

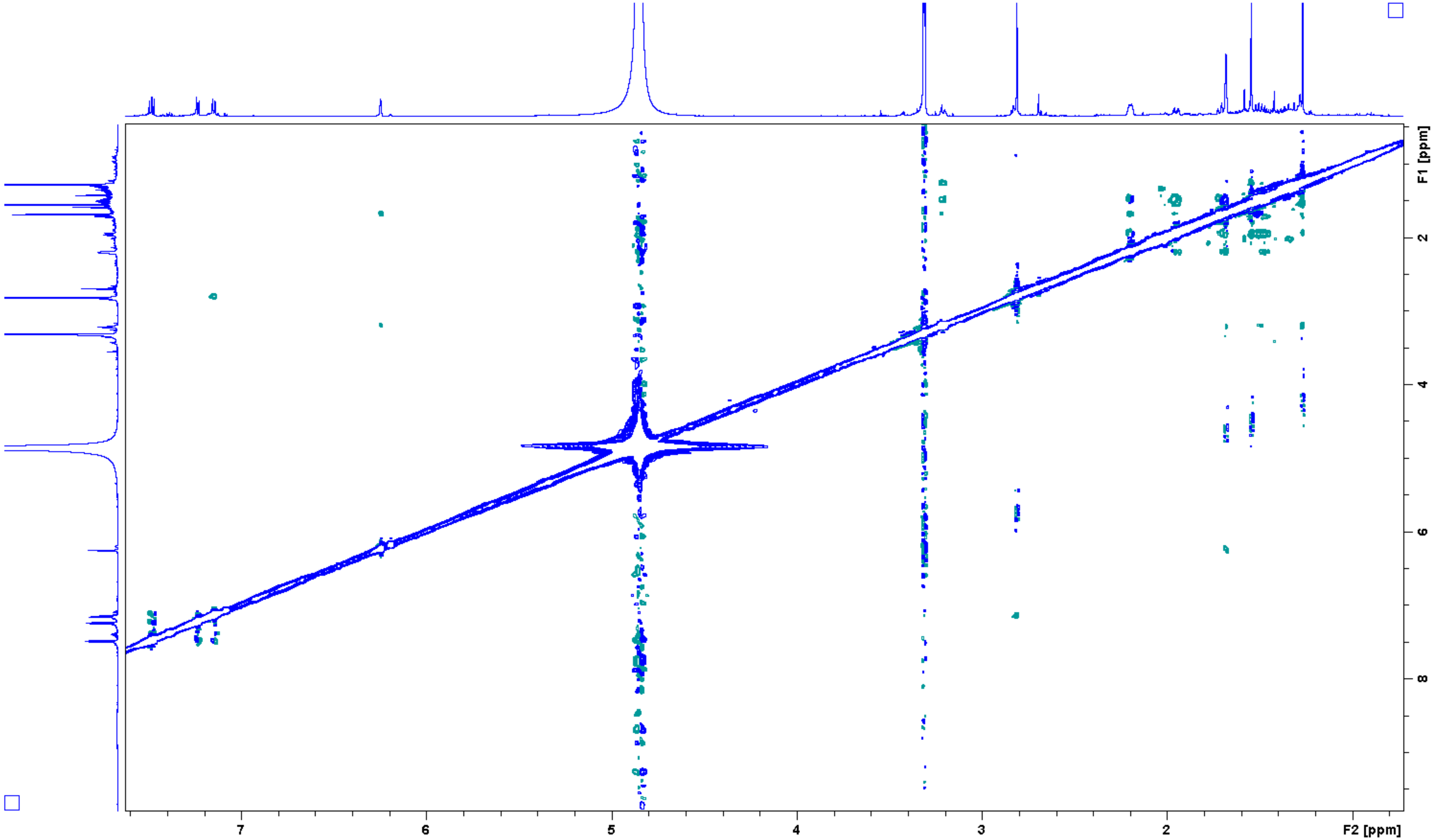

Figure S84. ^{1}H NMR spectrum of **16** (600 MHz, methanol-d_4).

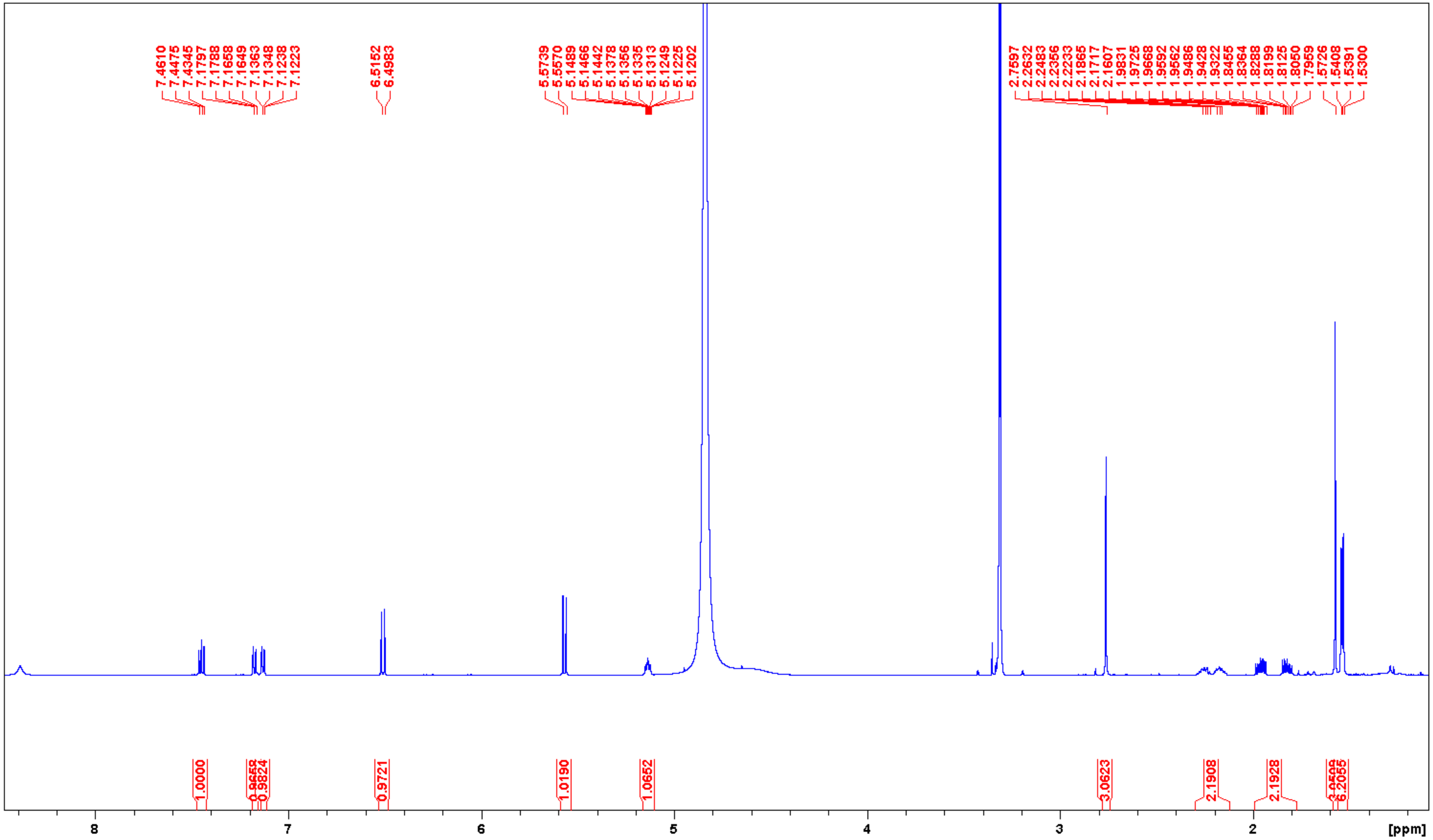

Figure S85. ^{13}C NMR spectrum of **16** (150 MHz, methanol-d_4).

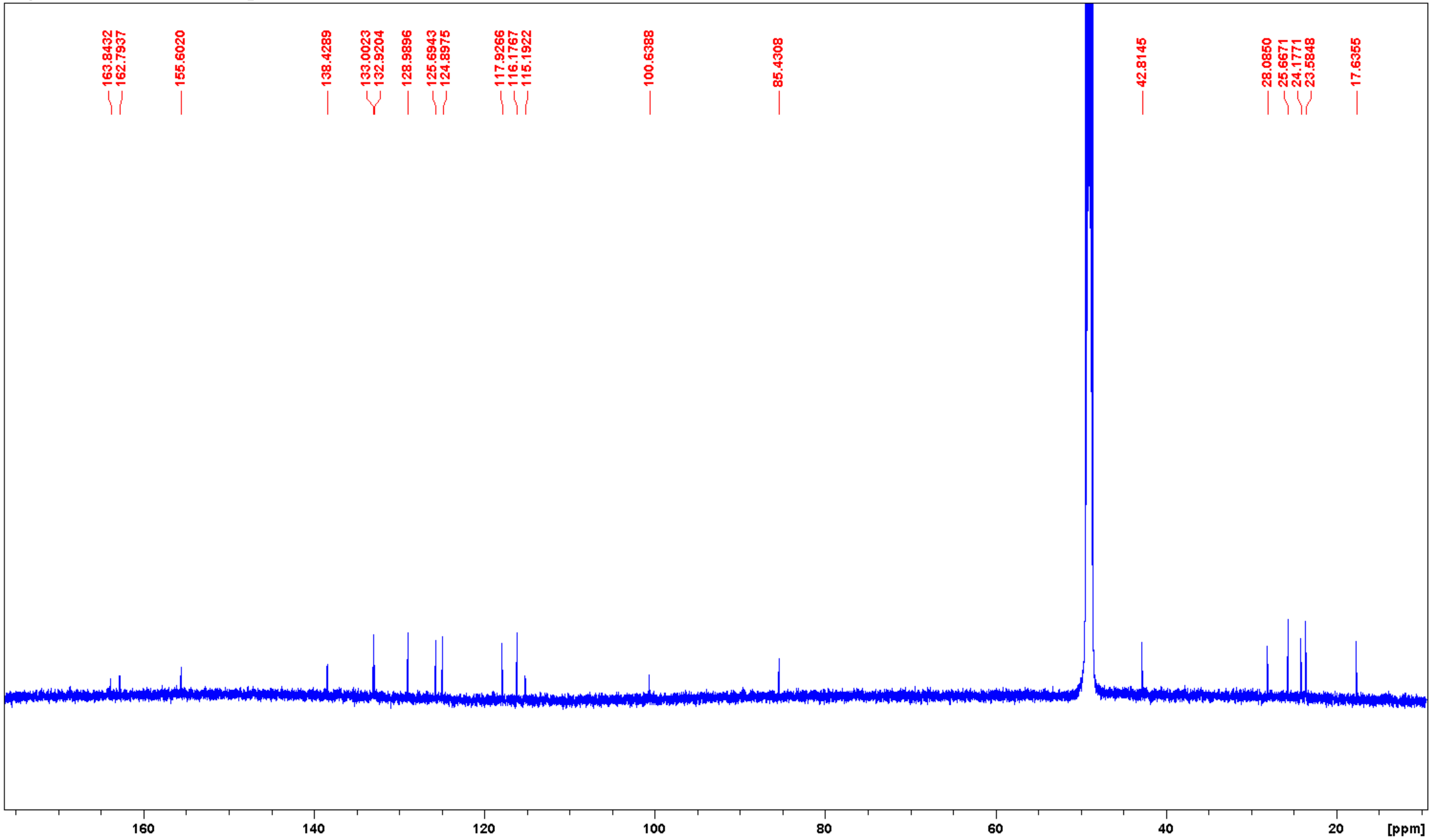

Figure S86. COSY spectrum of **16** (600 MHz, methanol-d_4).

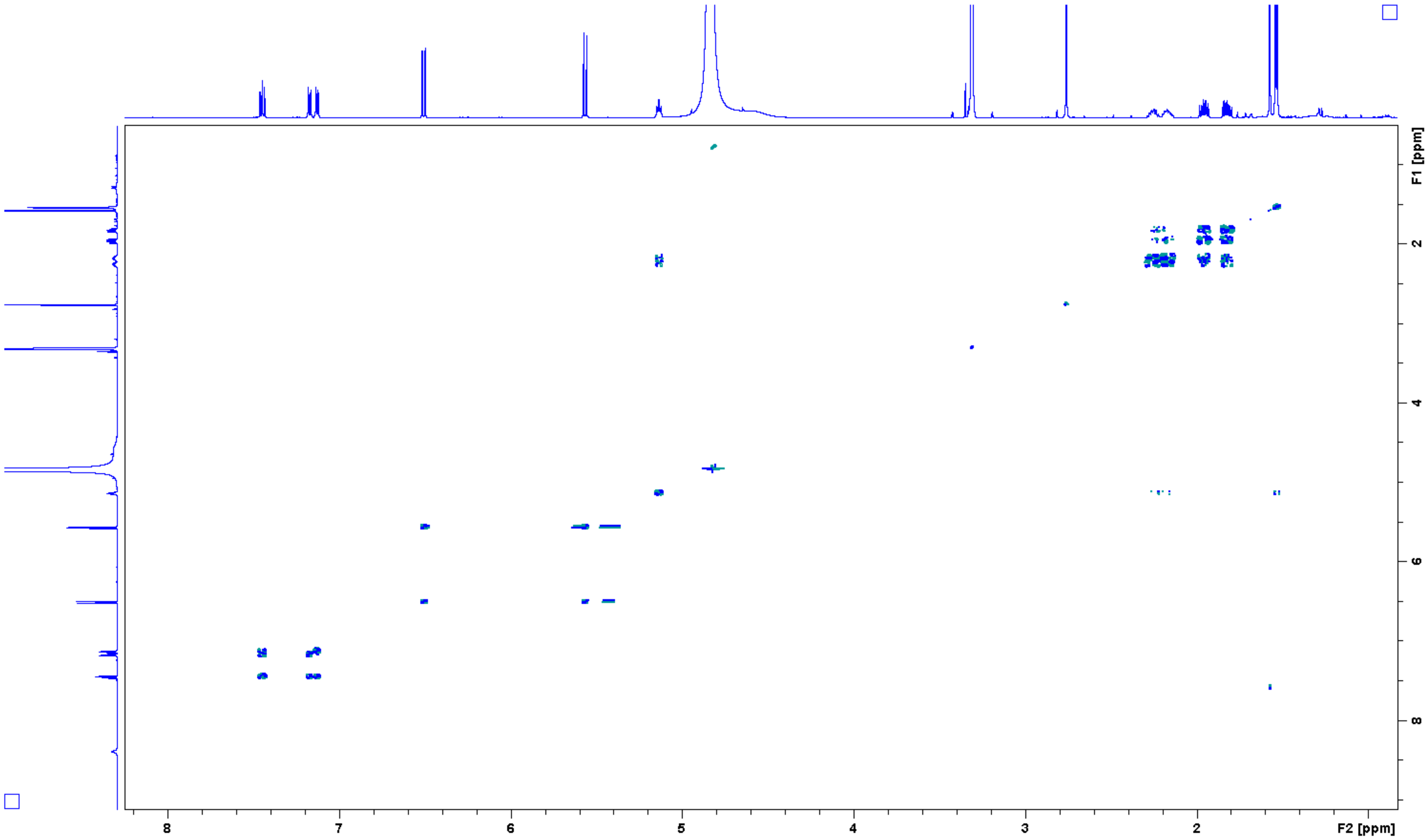

Figure S87. HSQC spectrum of **16** (600 MHz, methanol-d_4).

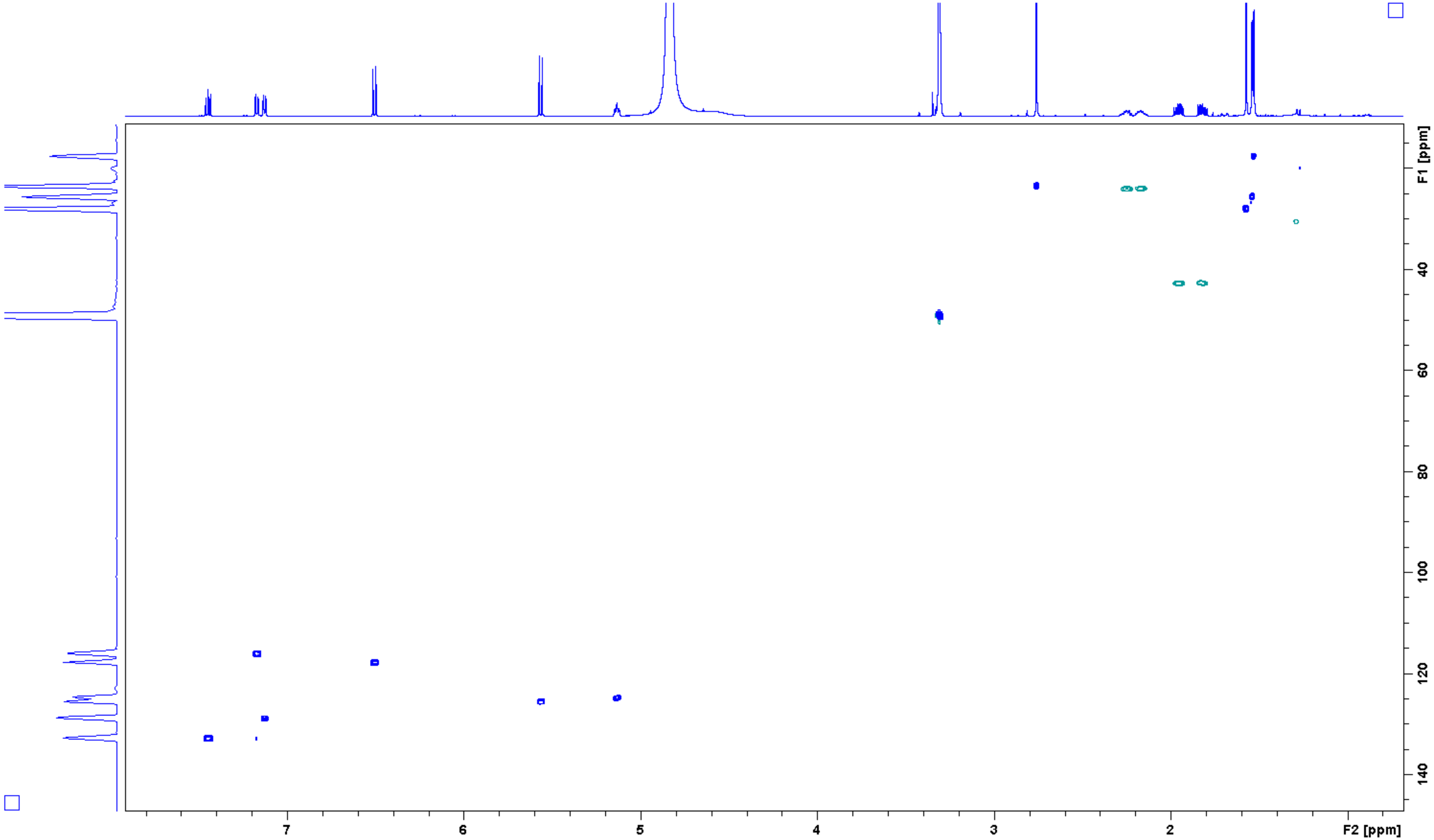

Figure S88. ^{1}H NMR spectrum of **17** (600 MHz, methanol-d_4).

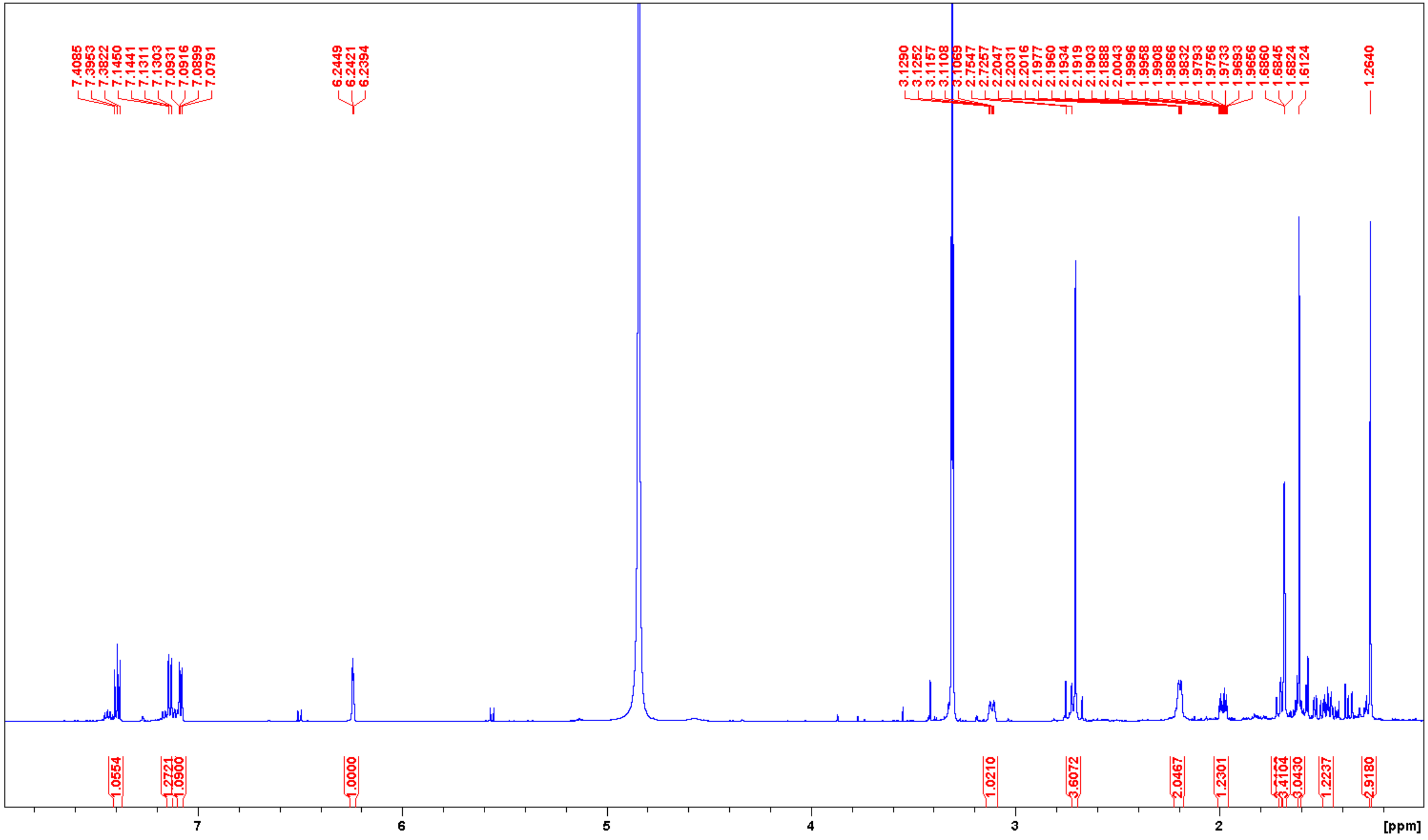

Figure S89. ^{13}C NMR spectrum of **17** (151 MHz, methanol-d_4).

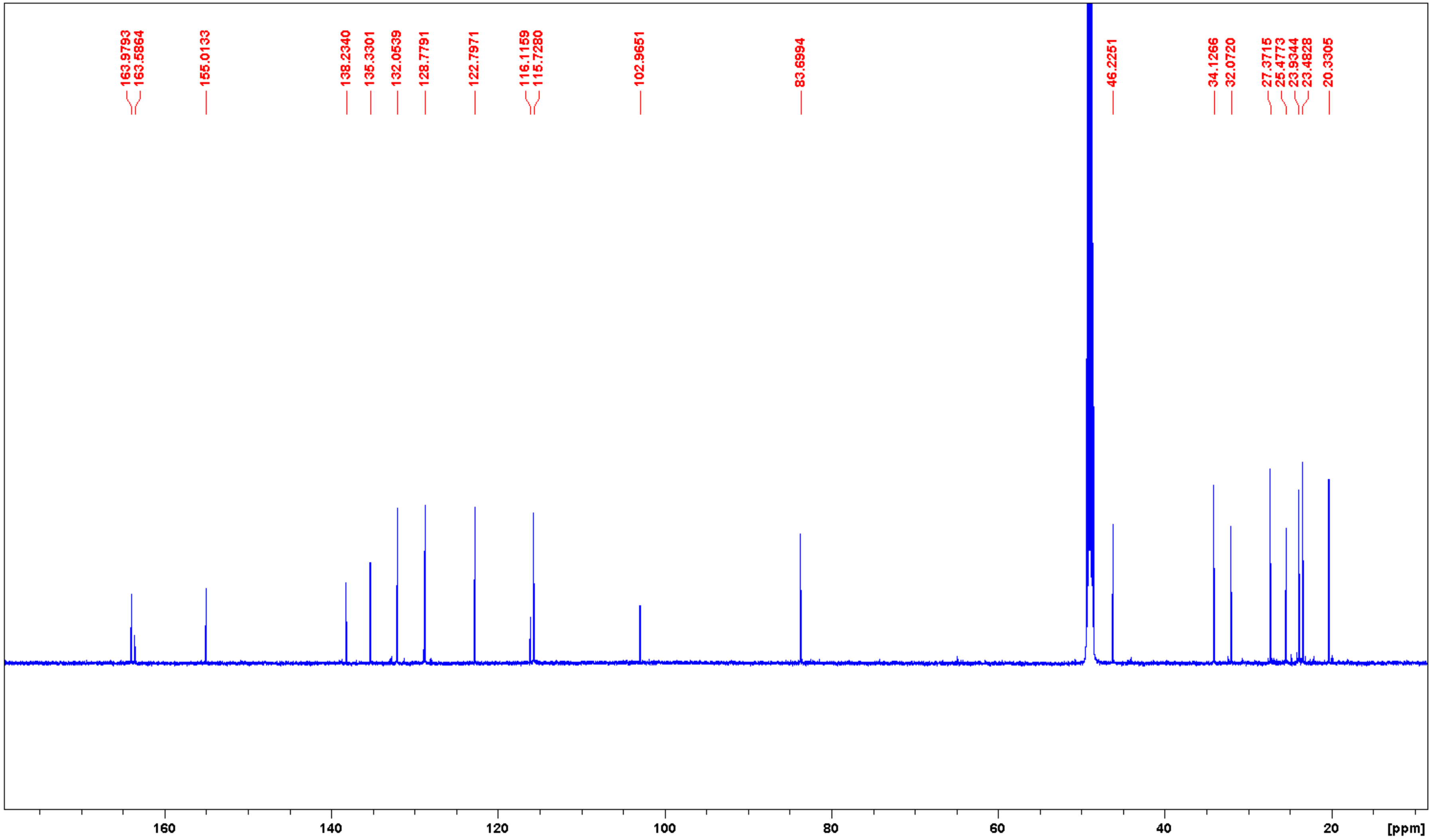

Figure S90. HSQC spectrum of **17** (600 MHz, methanol-d_4).

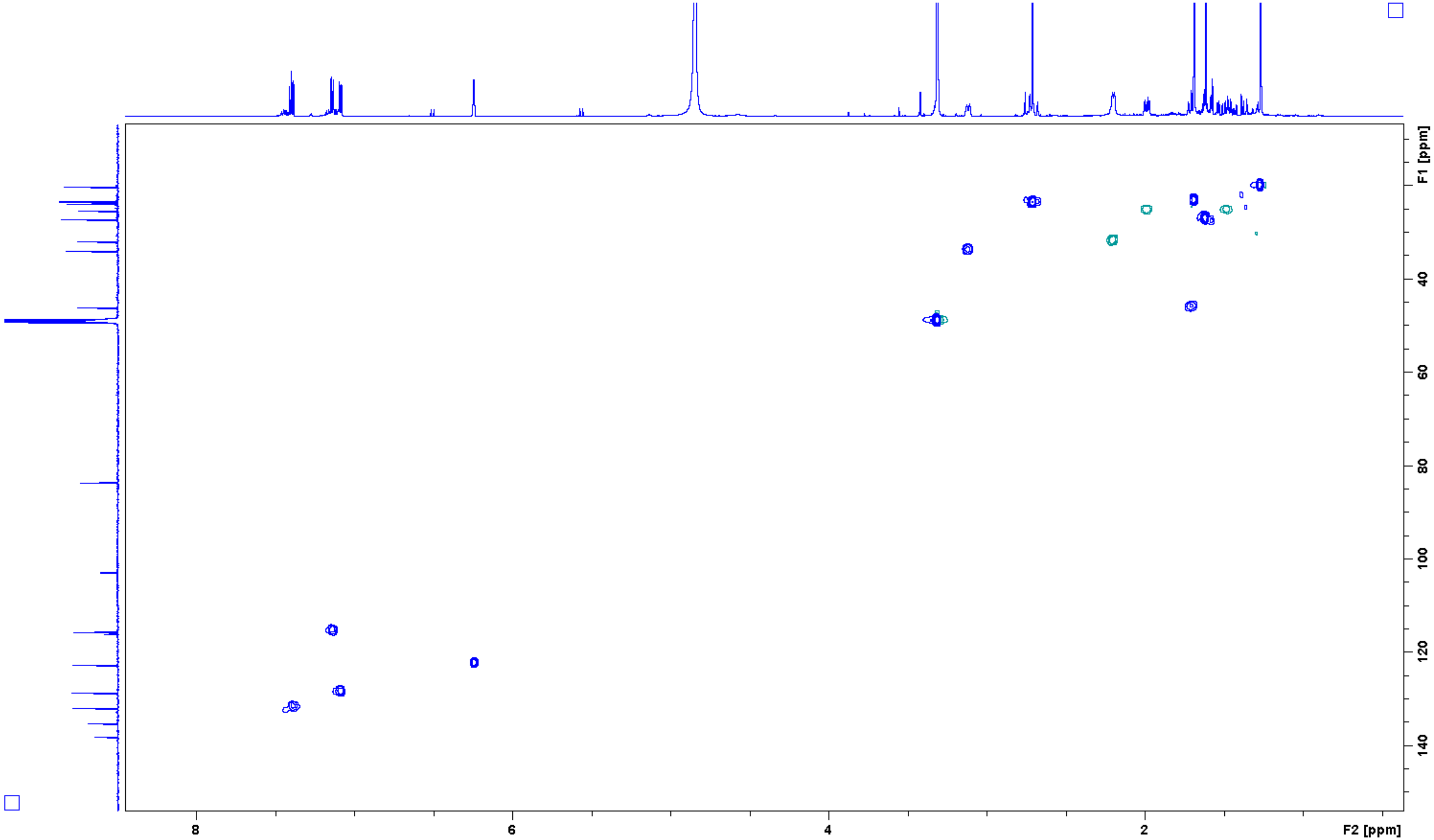

Figure S91. COSY spectrum of **17** (600 MHz, methanol-d_4).

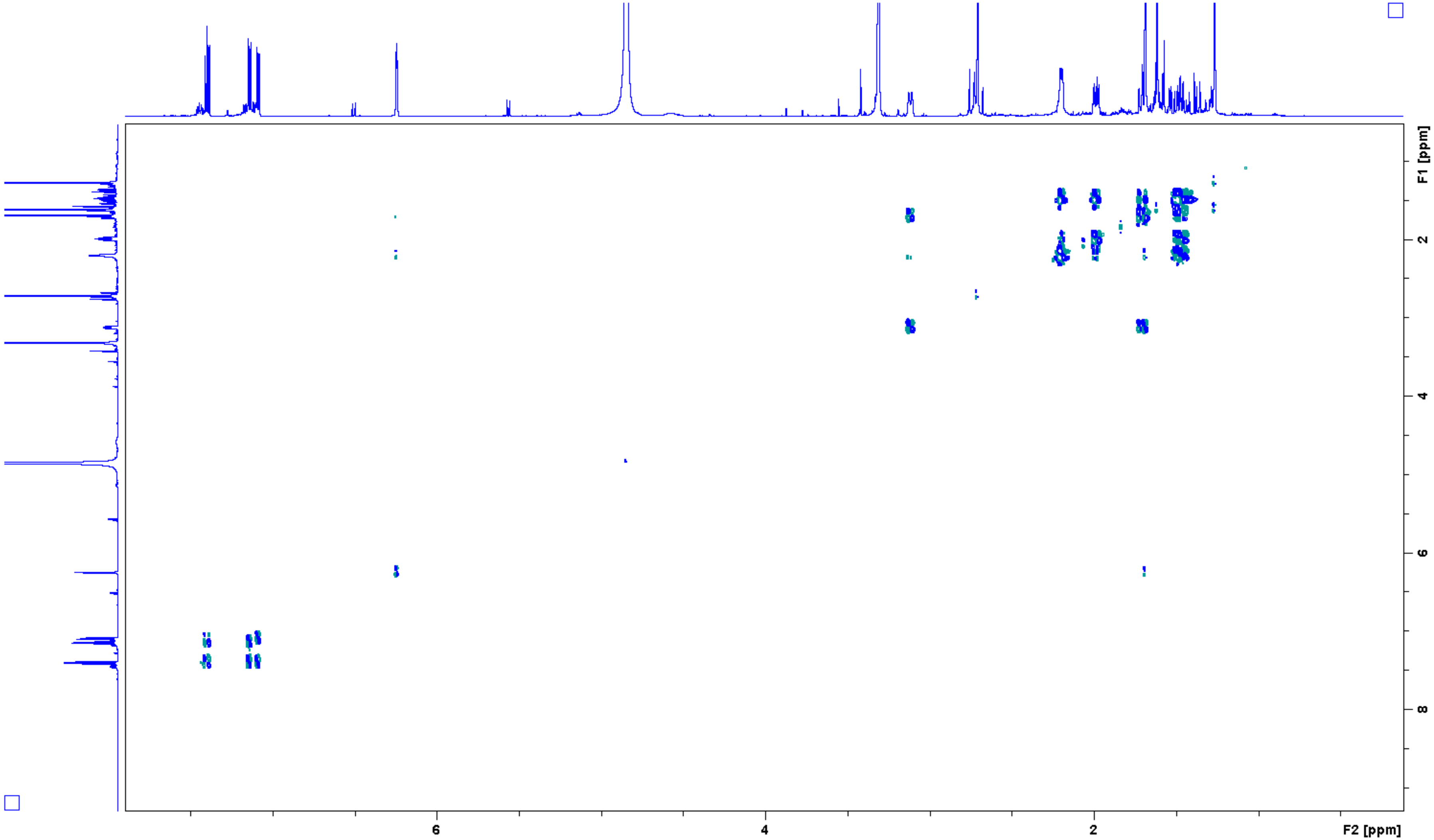

Figure S92. HMBC spectrum of **17** (600 MHz, methanol-d_4).

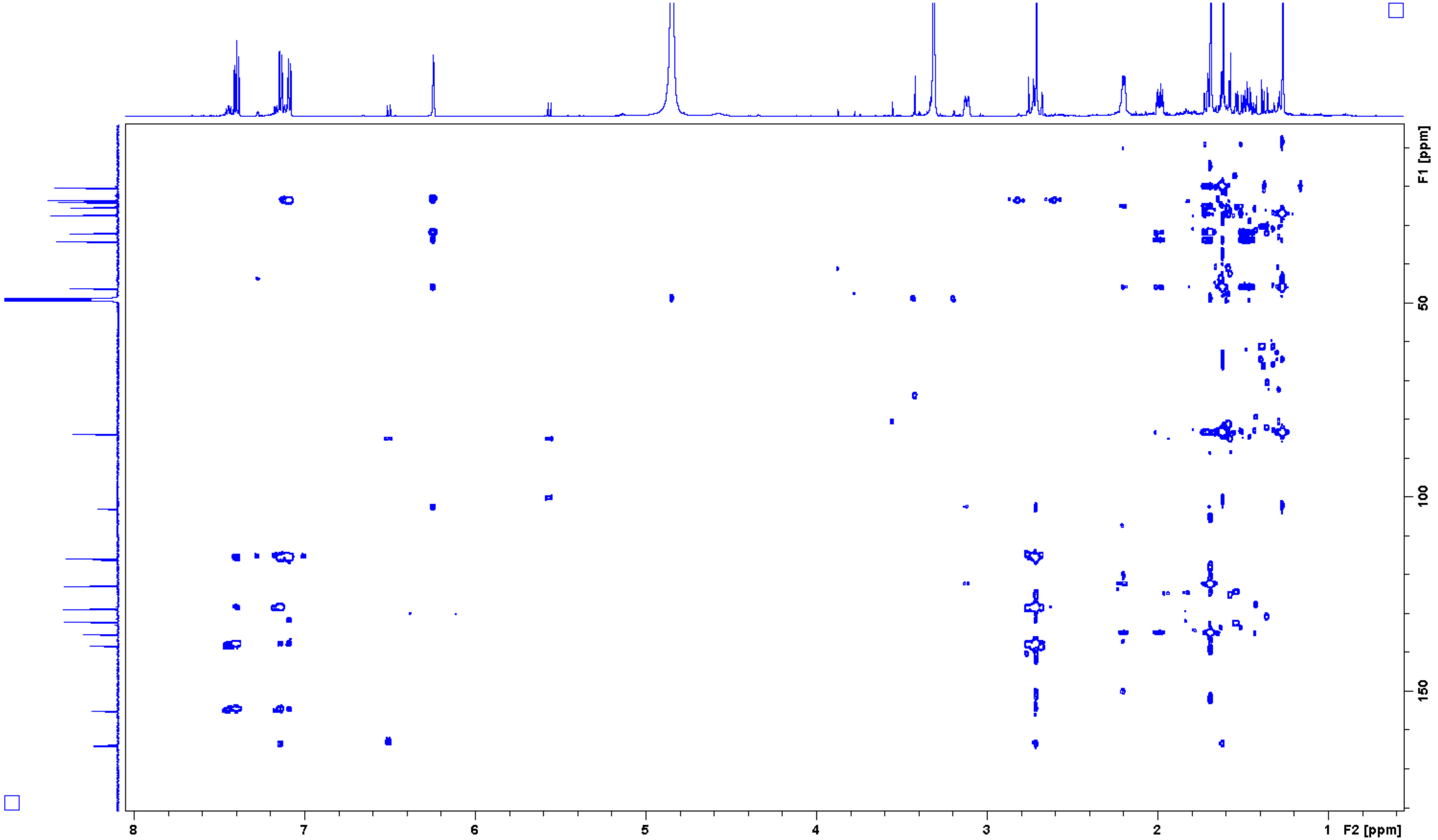

Figure S93. ROESY spectrum of **17** (600 MHz, methanol-d_4).

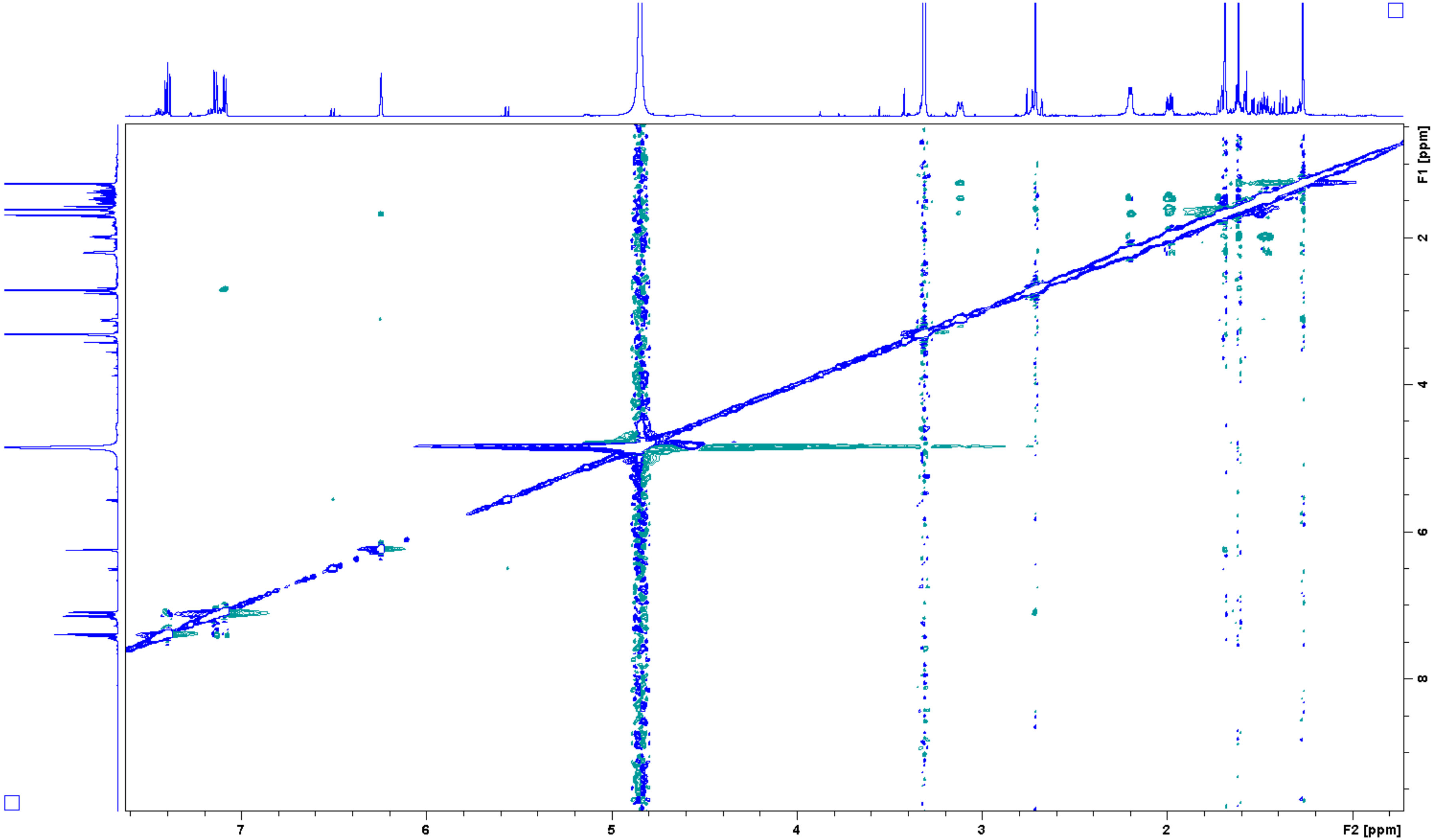

Figure S94. ^{1}H NMR spectrum of **18** (600 MHz, methanol-d_4).

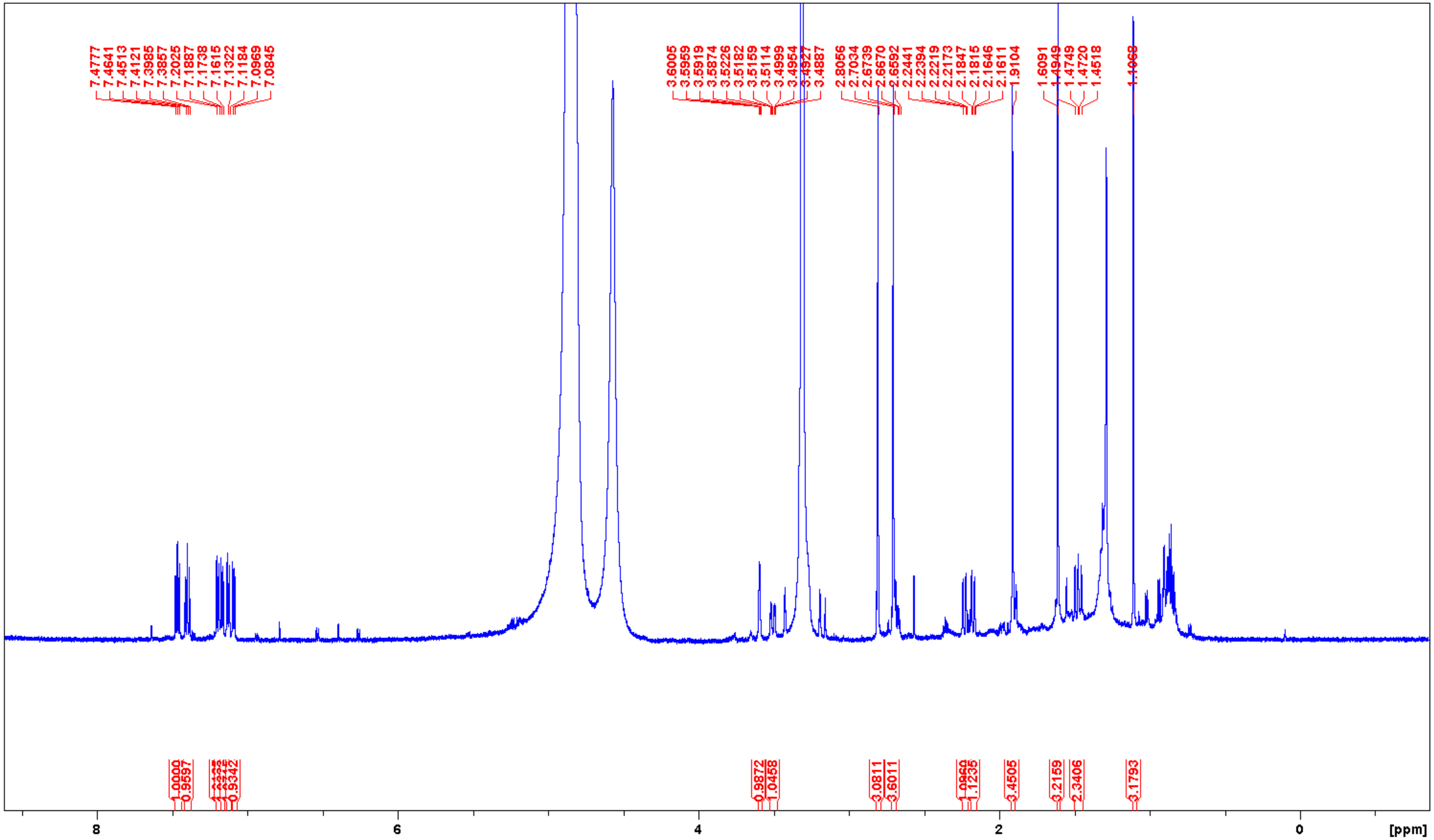

Figure S95. J-MOD NMR spectrum of **18** (600 MHz, methanol-d_4).

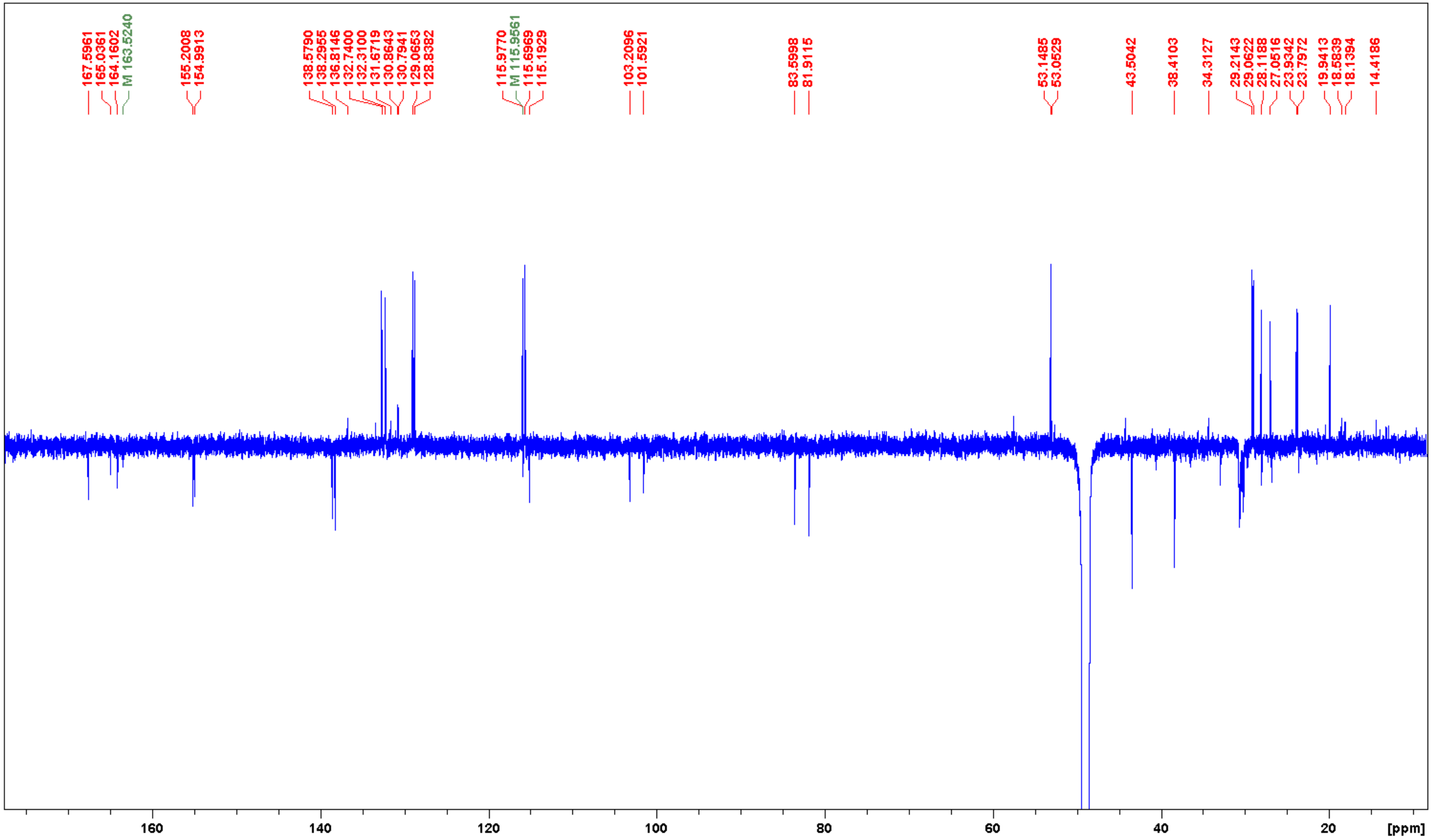

Figure S96. HSQC spectrum of **18** (600 MHz, methanol-d_4).

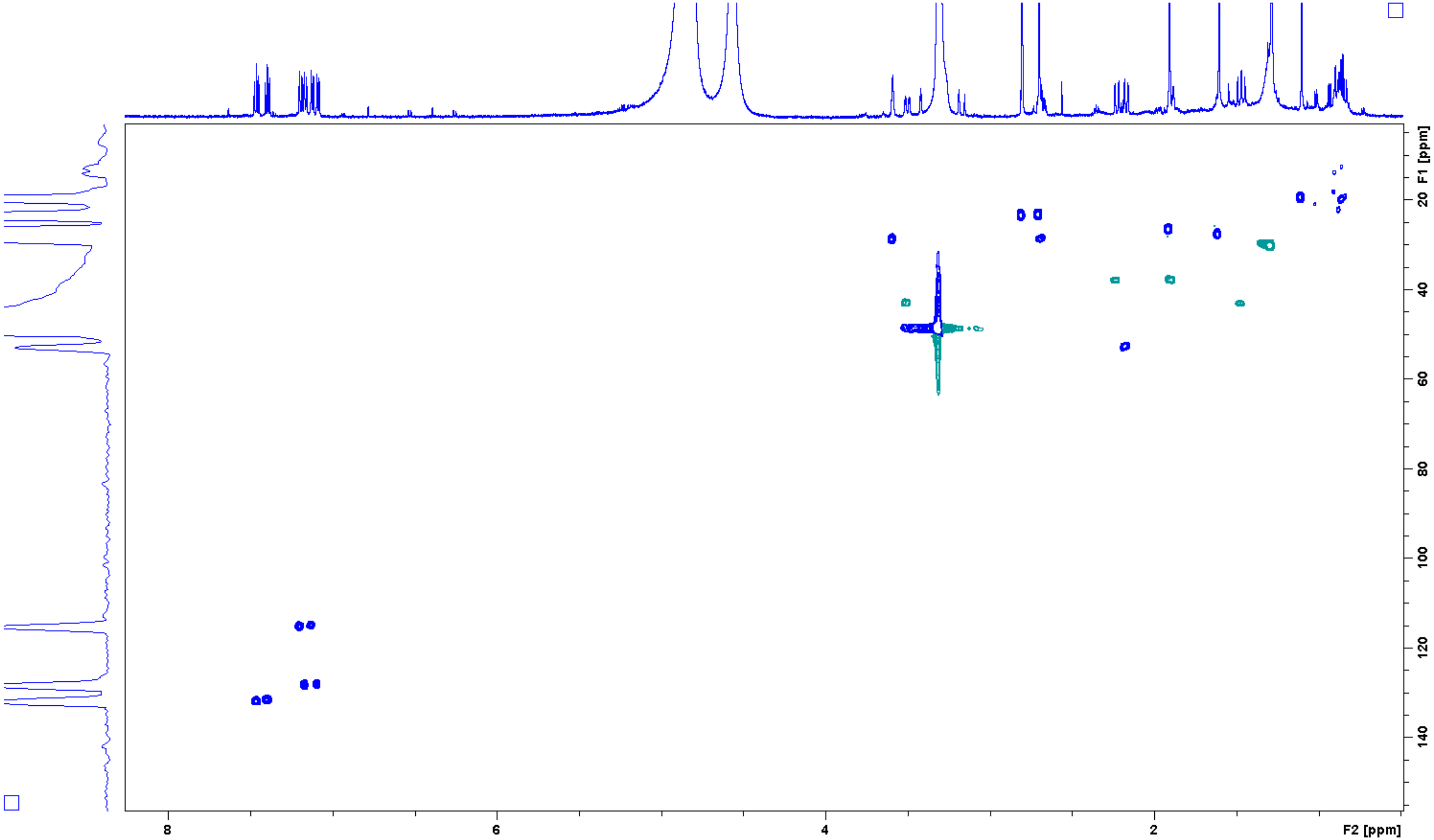

Figure S97. COSY spectrum of **18** (600 MHz, methanol-d_4).

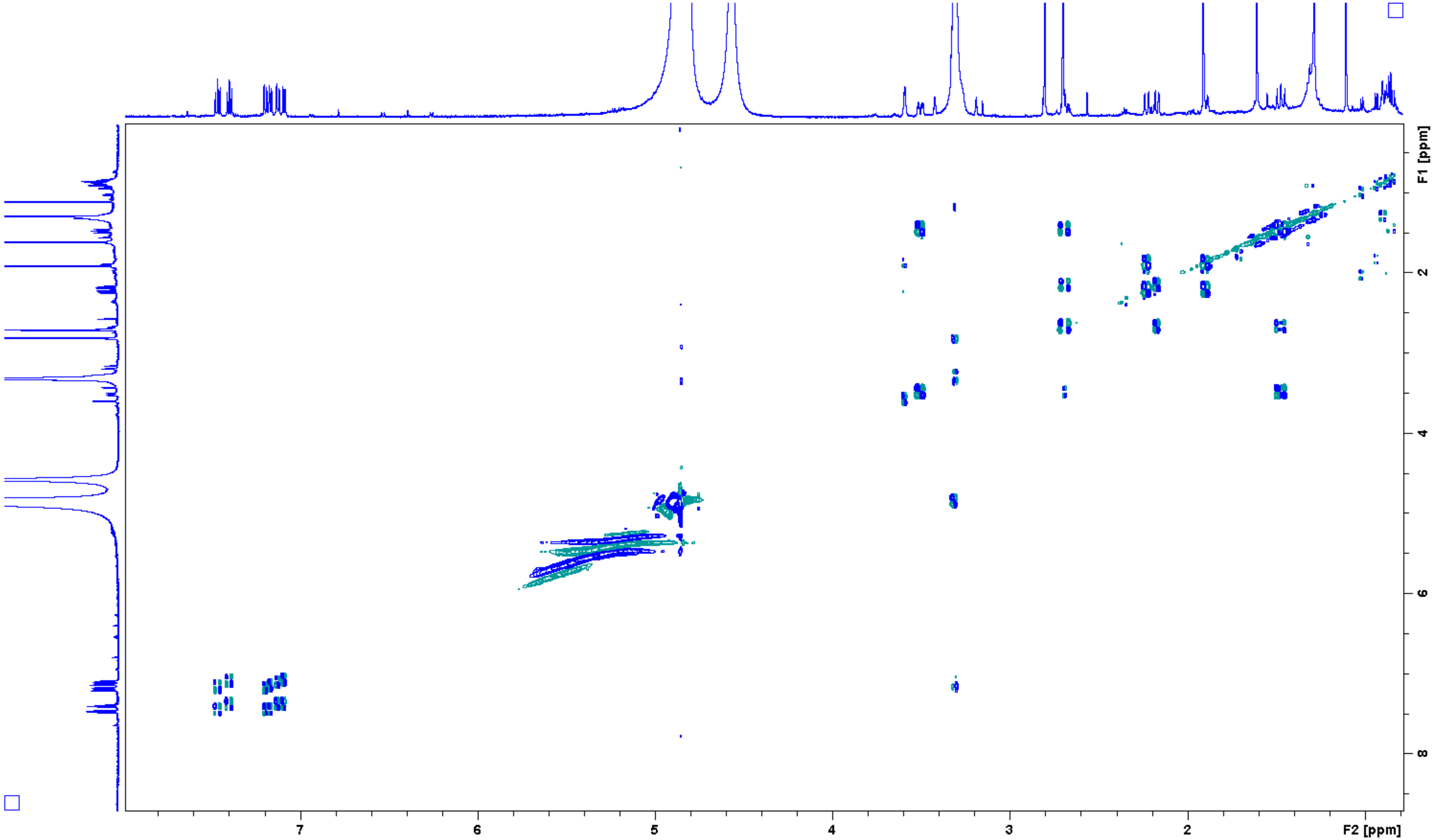

Figure S98. HMBC spectrum of **18** (600 MHz, methanol-d_4).

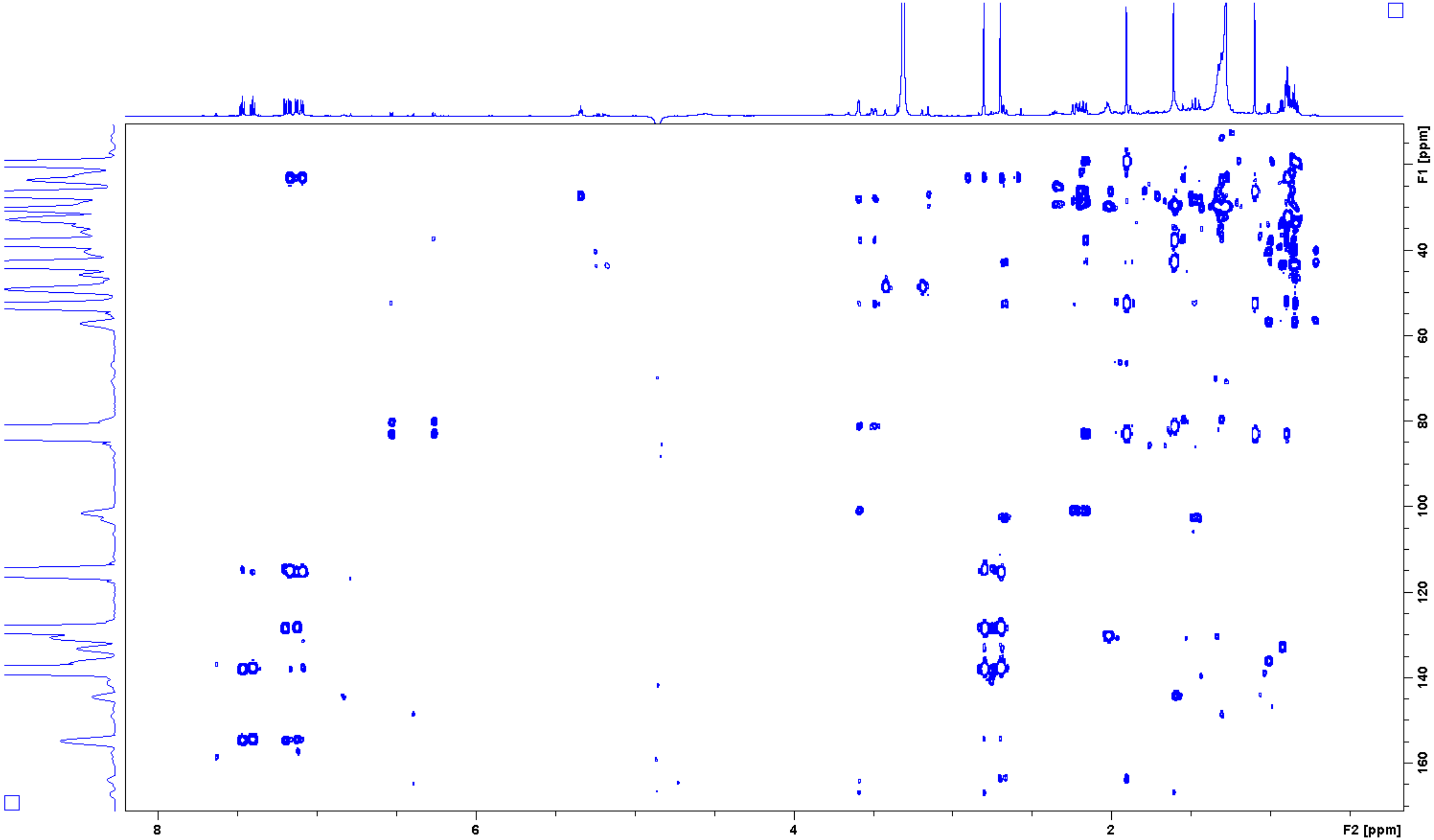

Figure S99. ROESY spectrum of **18** (600 MHz, methanol-d_4).

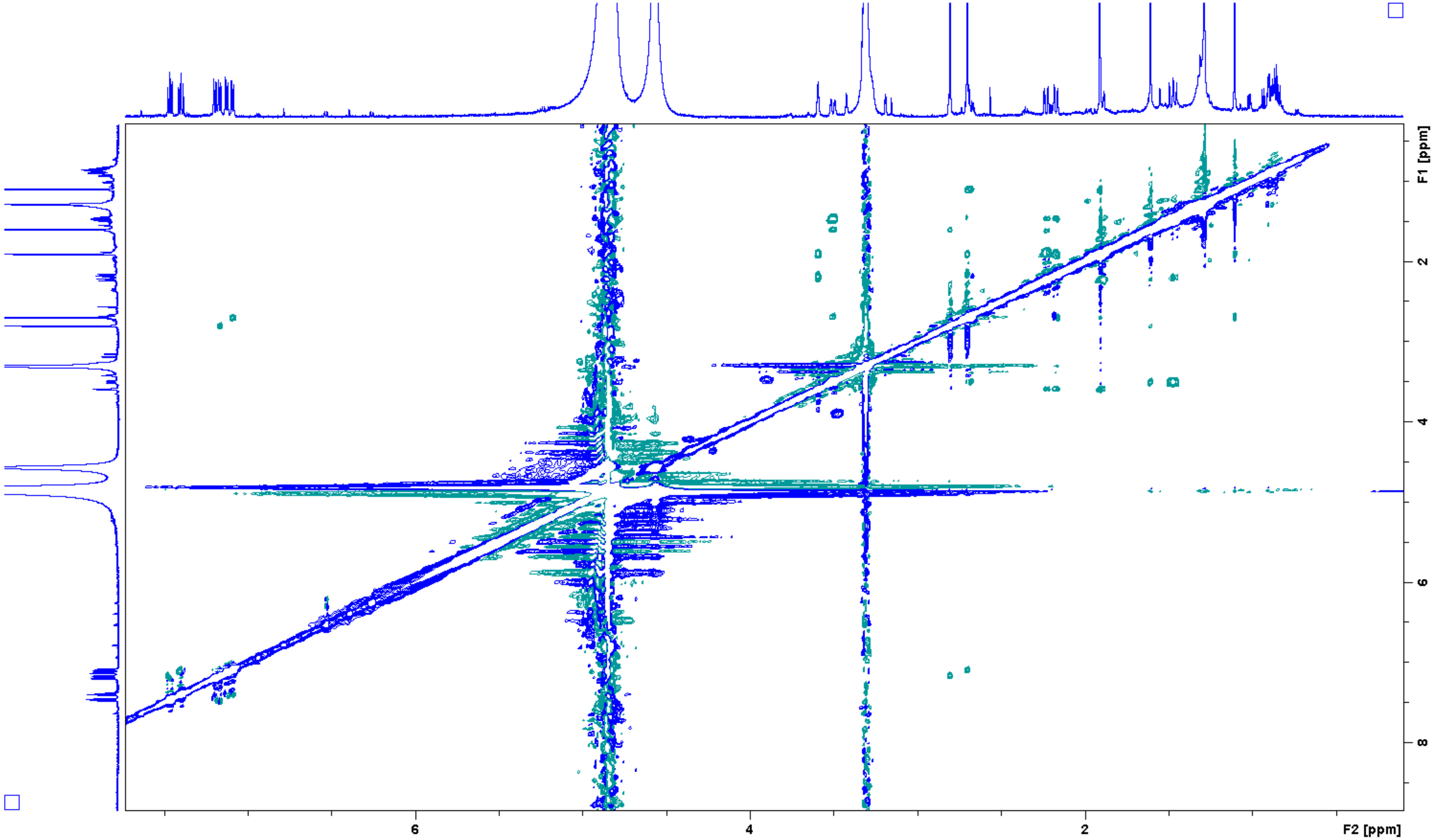

Figure S100. ^{1}H NMR spectrum of **19** (600 MHz, methanol-d_4).

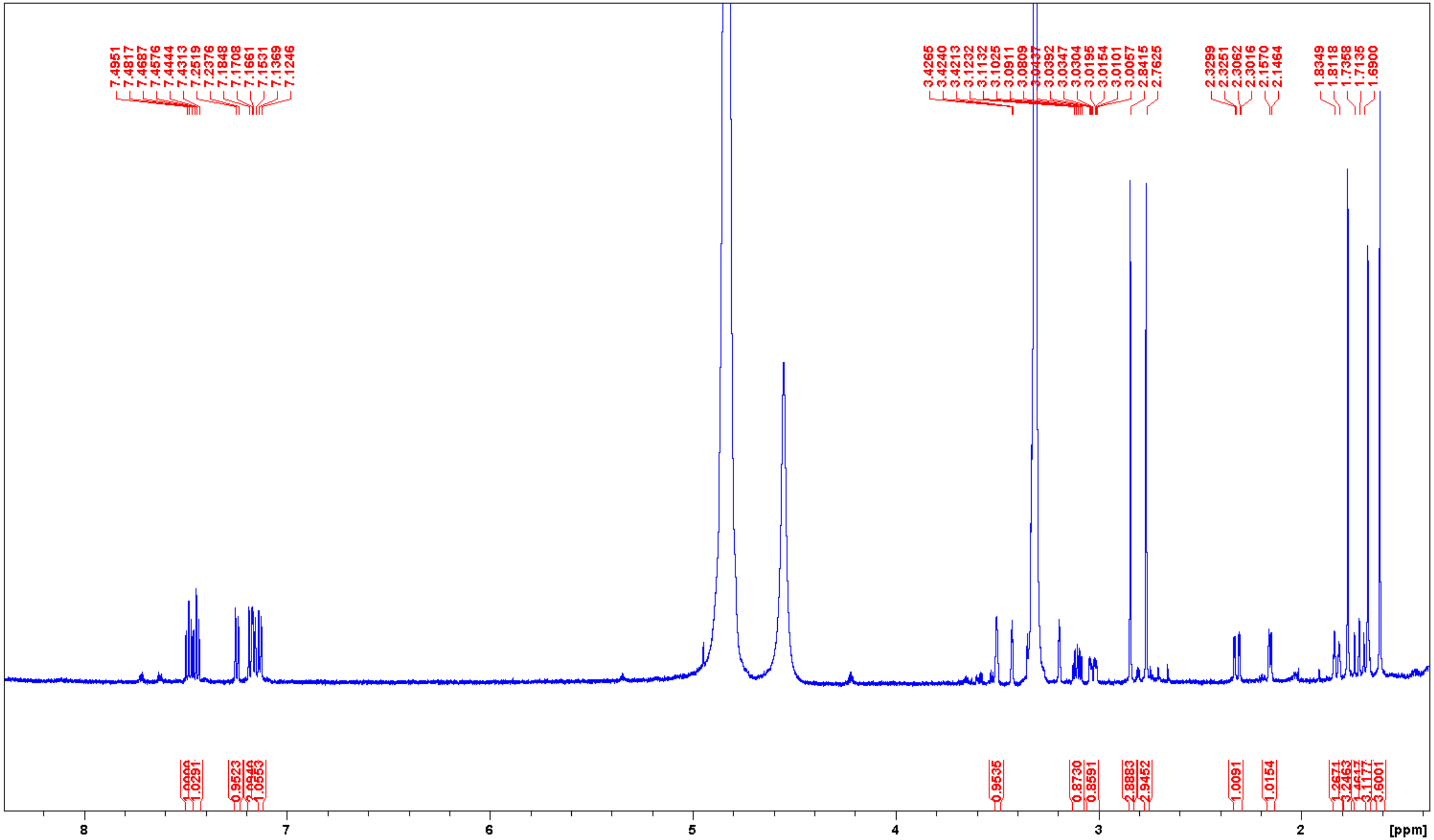

Figure S101. J-MOD NMR spectrum of **19** (151 MHz, methanol-d_4).

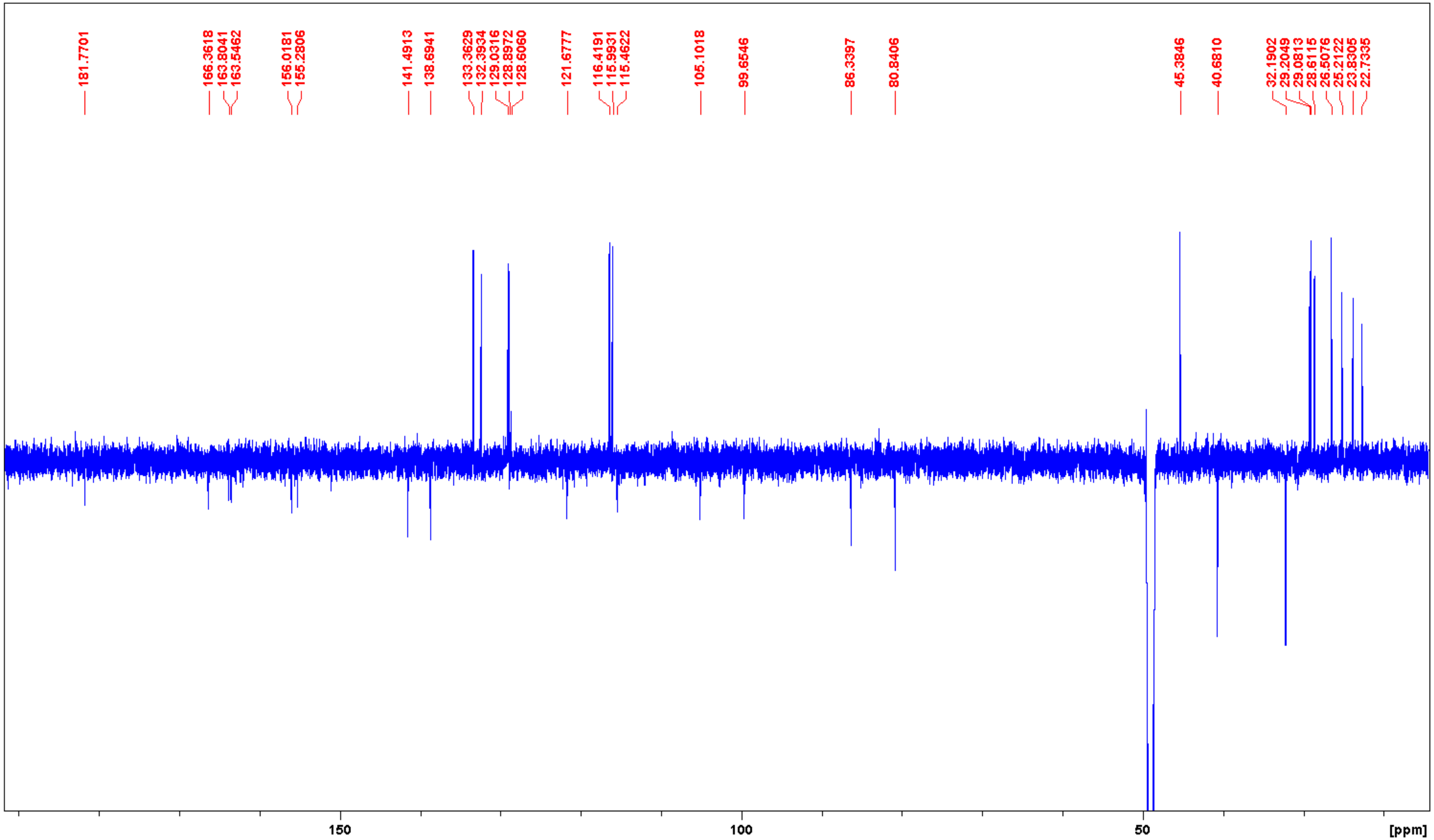

Figure S102. HSQC spectrum of **19** (600 MHz, methanol-d_4).

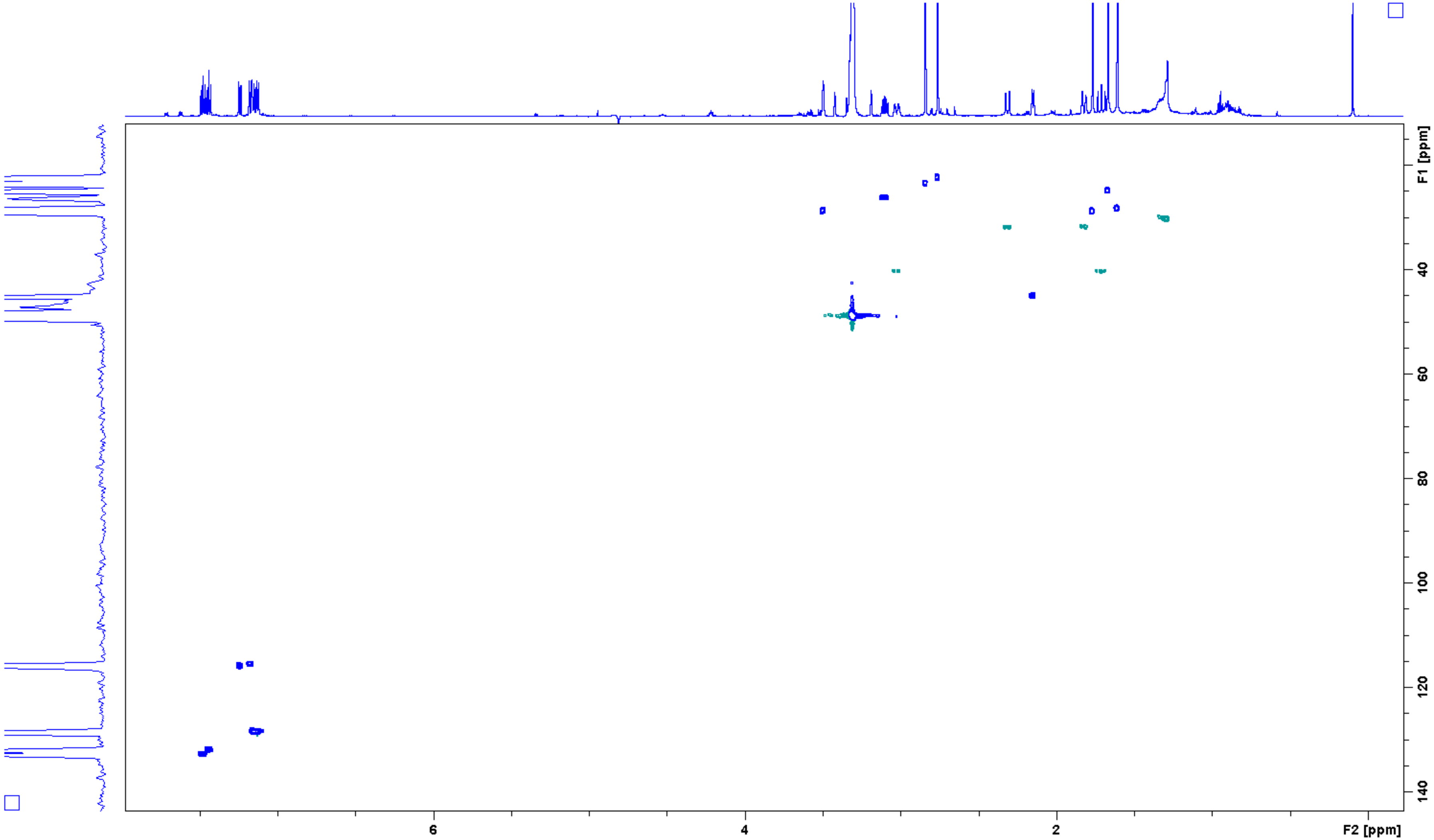

Figure S103. COSY spectrum of **19** (600 MHz, methanol-d_4).

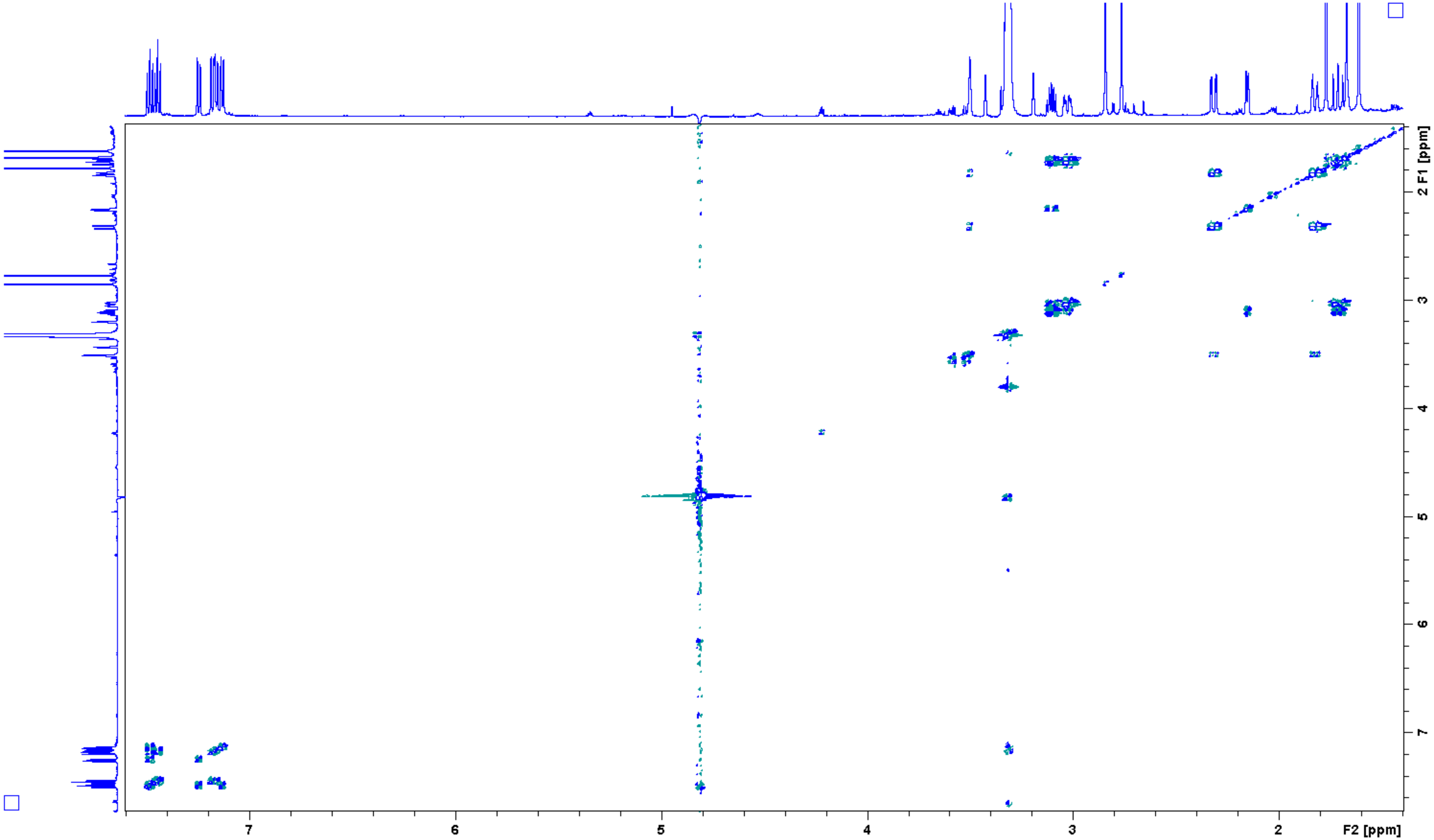

Figure S104. HMBC spectrum of **19** (600 MHz, methanol-d_4).

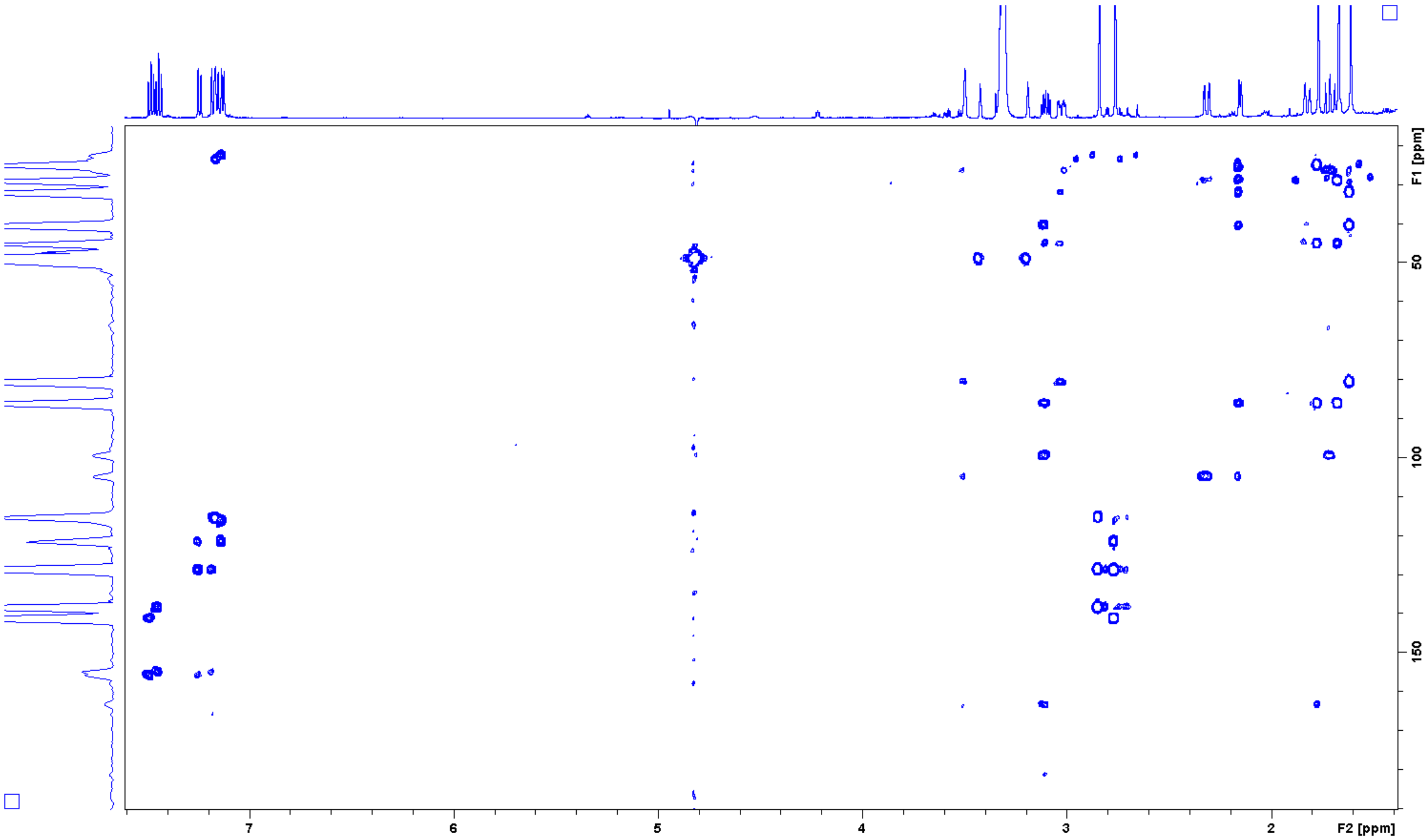

Figure S105. ROESY spectrum of **19** (600 MHz, methanol-d_4).

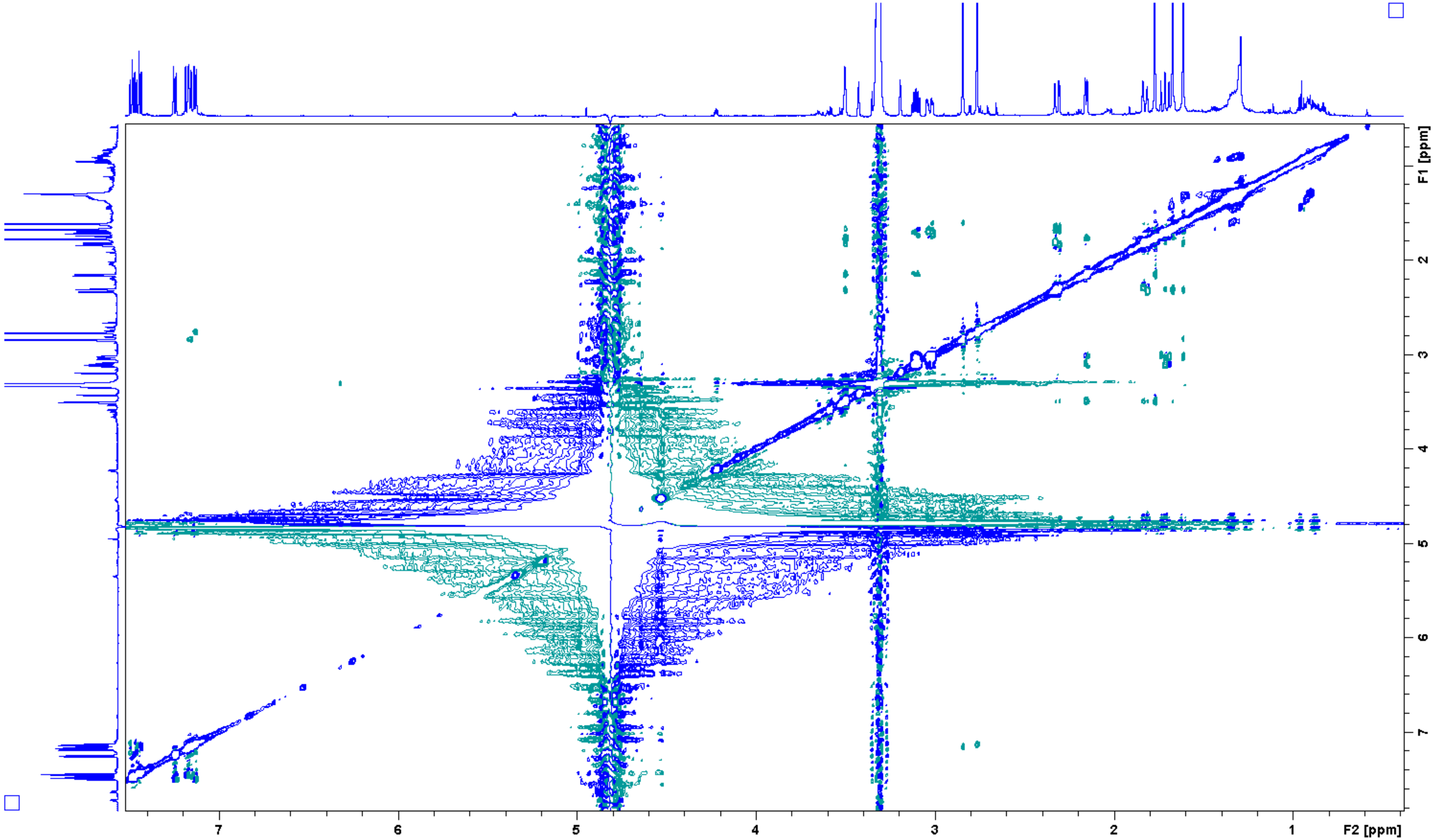

Figure S106. ^{1}H NMR spectrum of **23** (600 MHz, methanol-d_4).

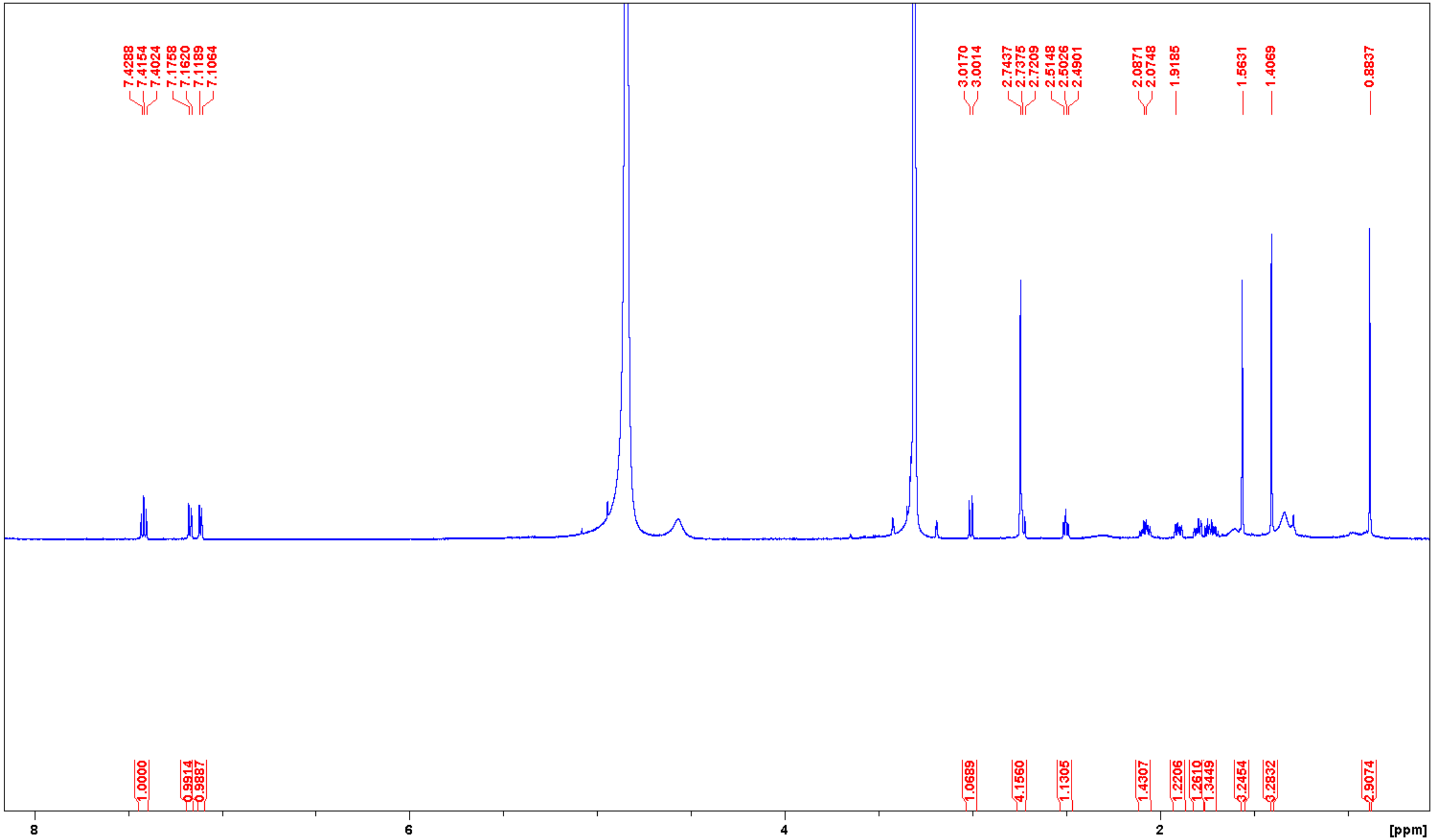

Figure S107. ^{13}C NMR spectrum of **23** (151 MHz, methanol-d_4).

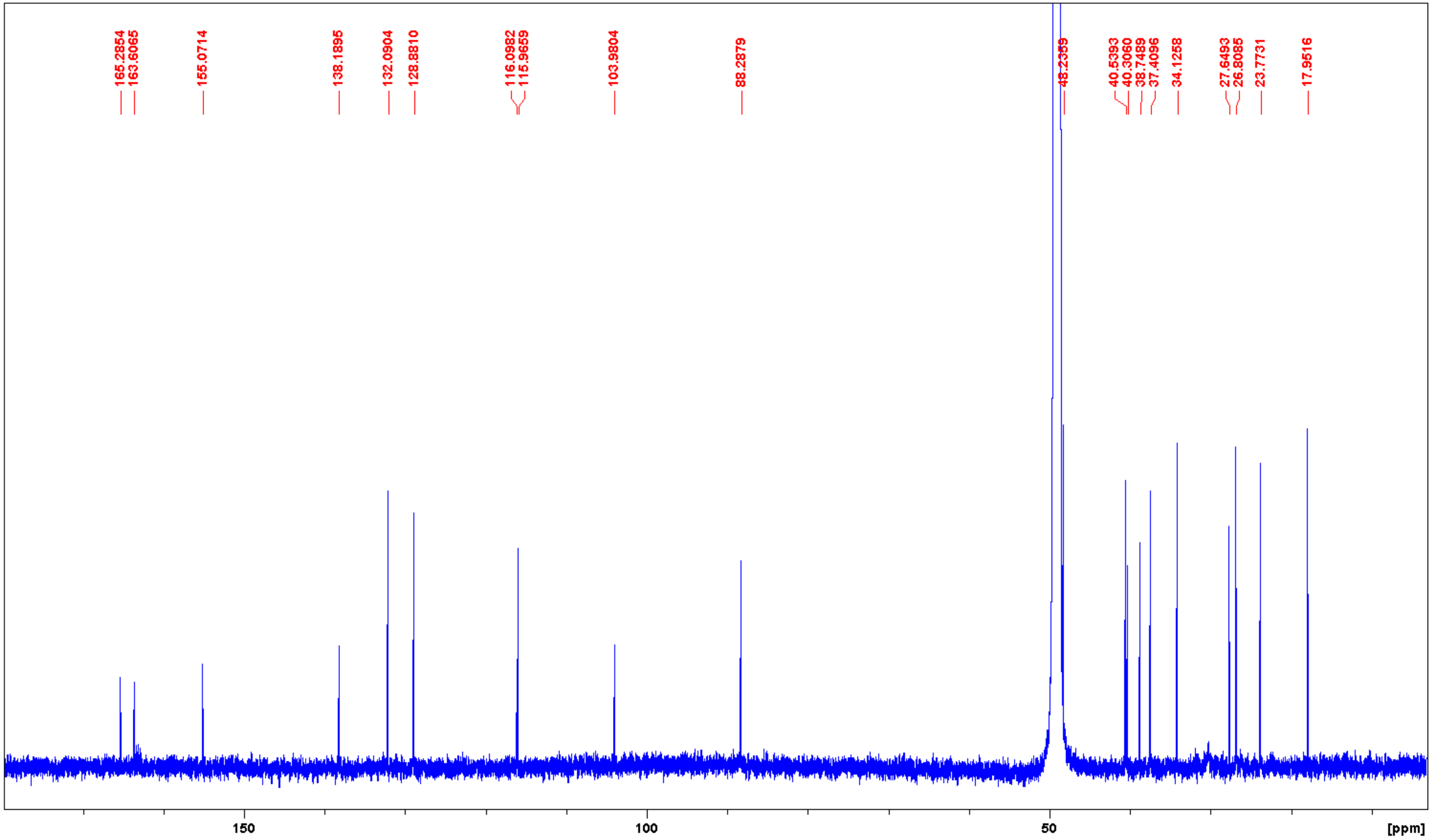

Figure S108. HSQC spectrum of **23** (600 MHz, methanol-d_4).

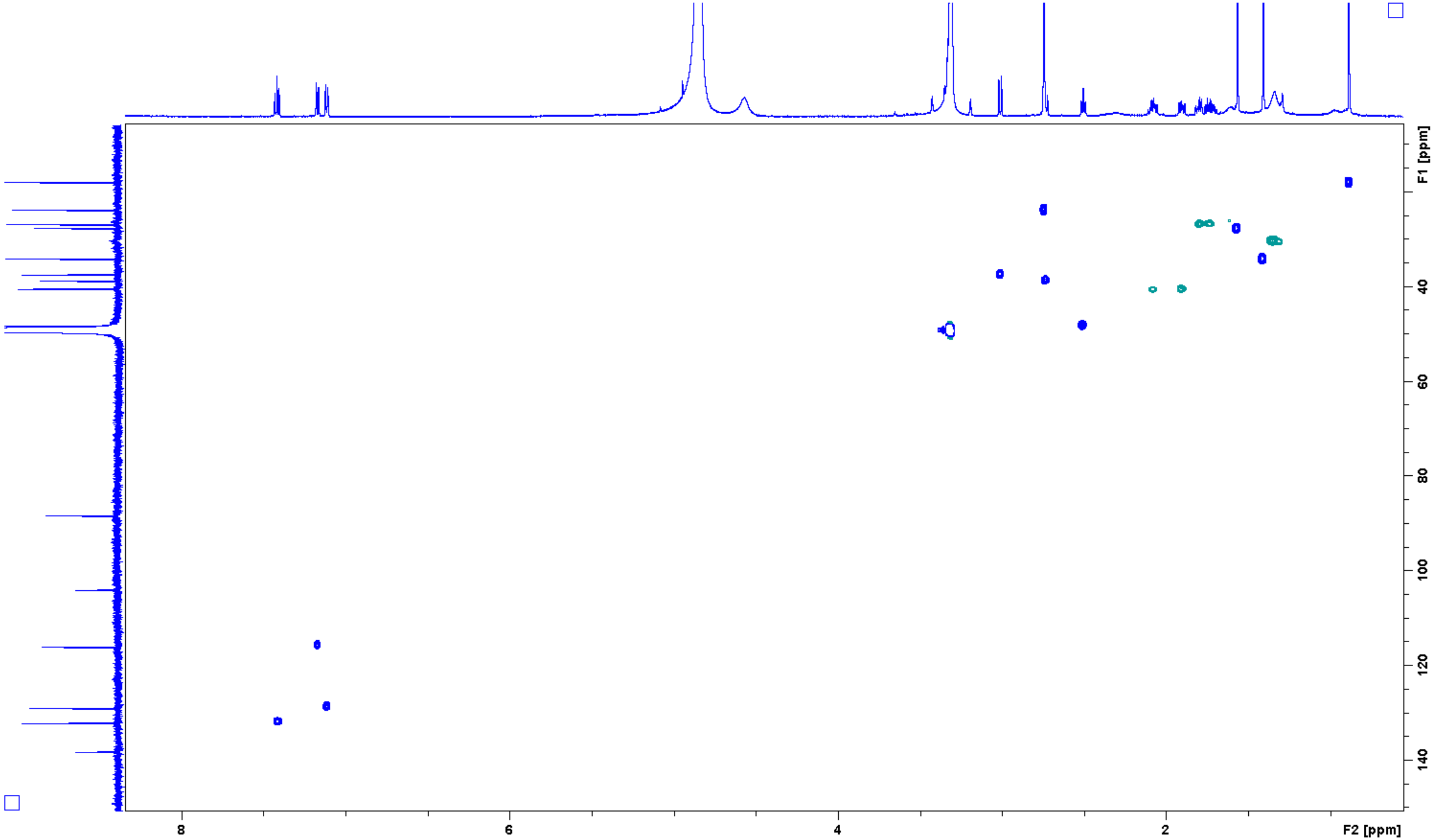

Figure S109. COSY spectrum of **23** (600 MHz, methanol-d_4).

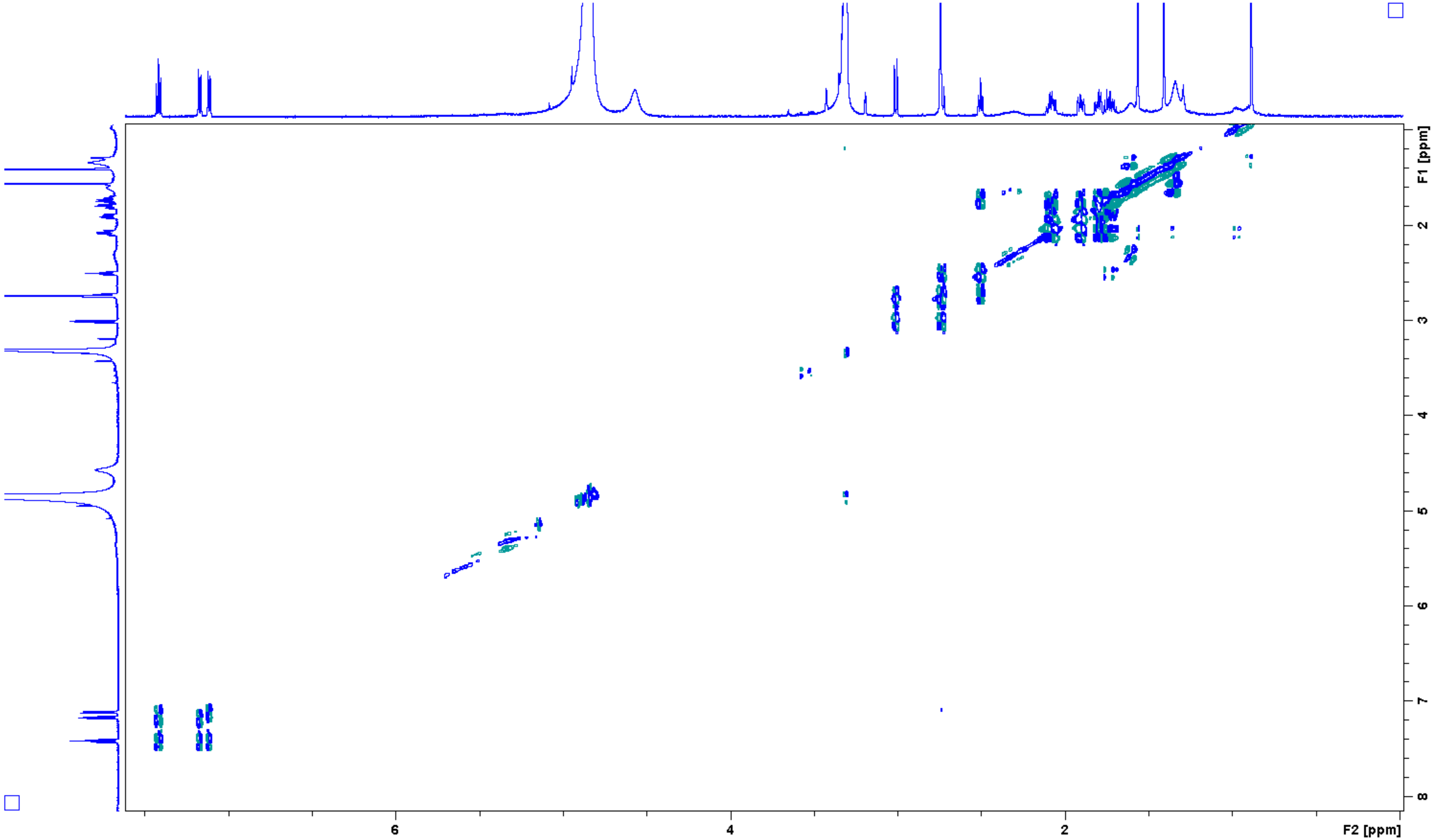

Figure S110. HMBC spectrum of **23** (600 MHz, methanol-d_4).

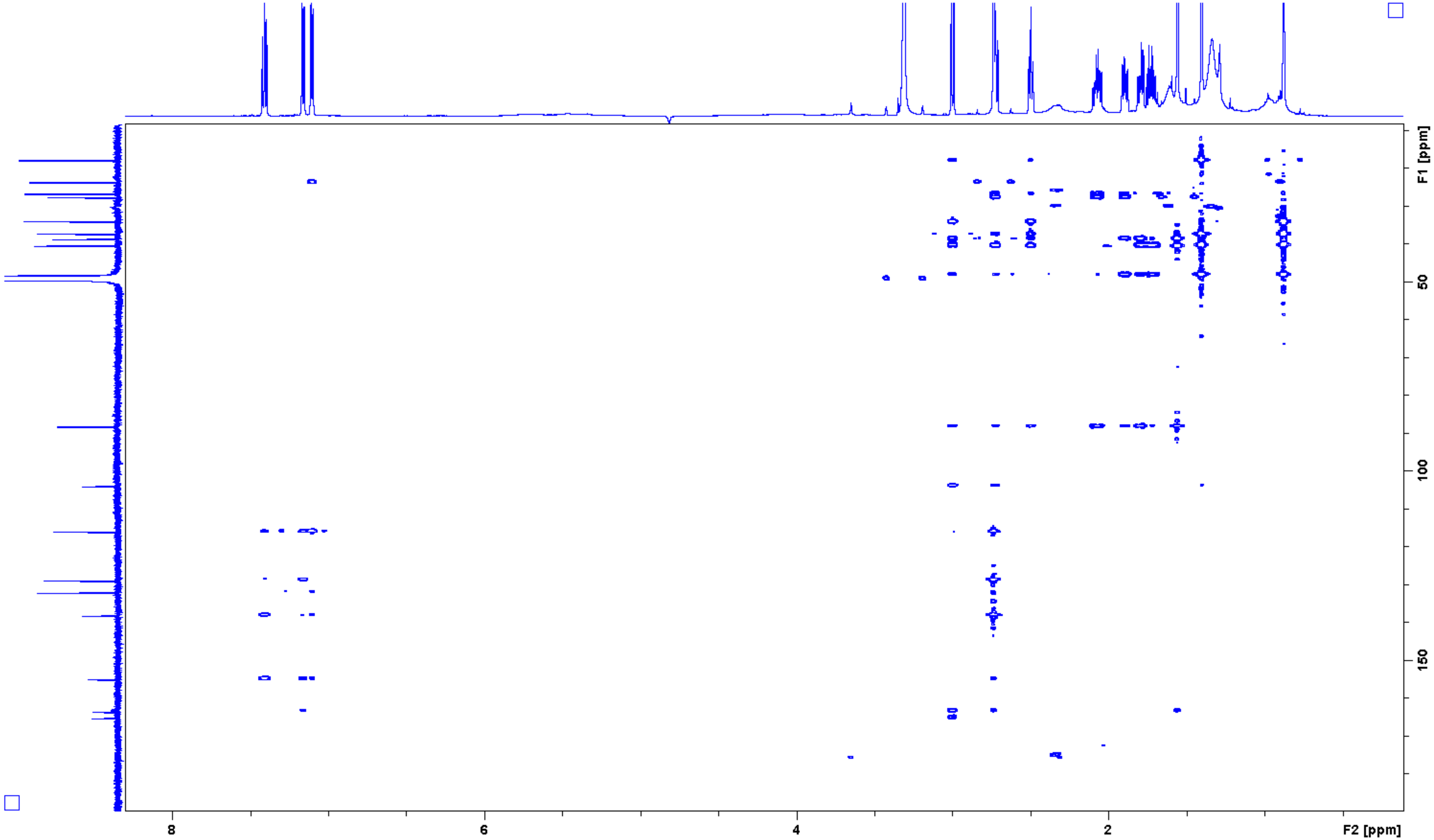

Figure S111. ROESY spectrum of **23** (600 MHz, methanol-d_4).

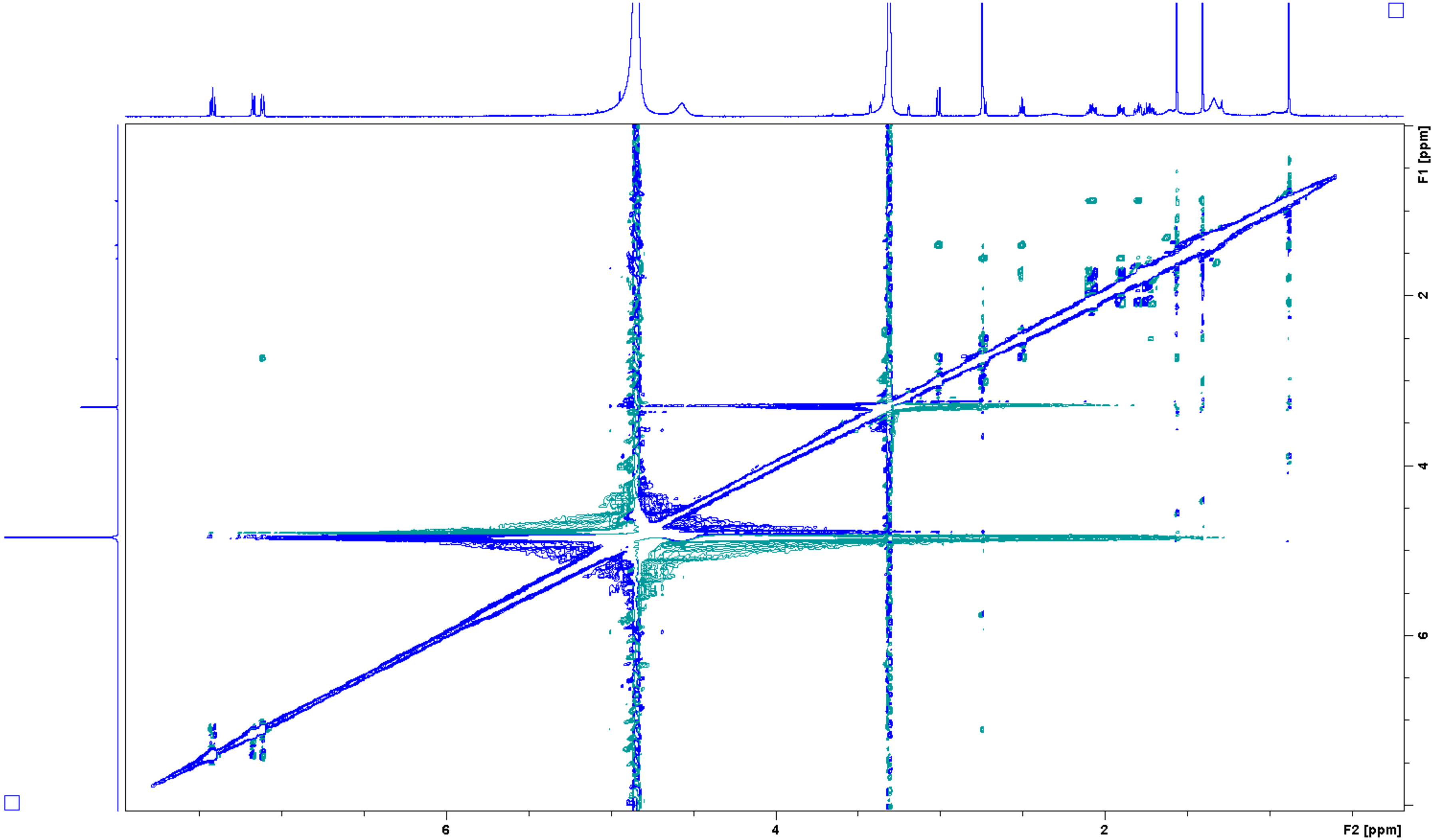

Figure S112. ^{1}H NMR spectrum of **24** (600 MHz, methanol-d_4).

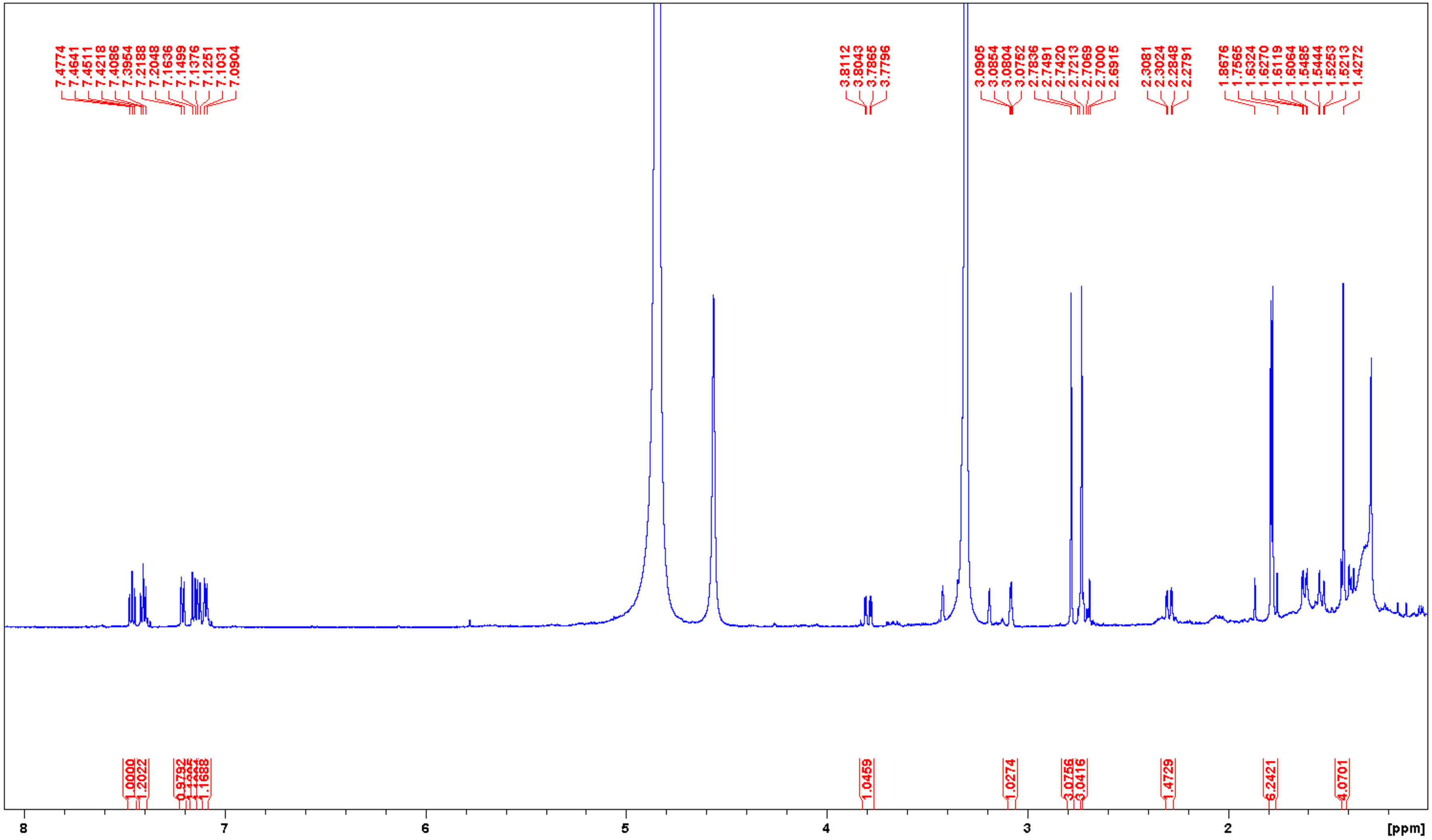

Figure S113. ^{13}C NMR spectrum of **24** (151 MHz, methanol-d_4).

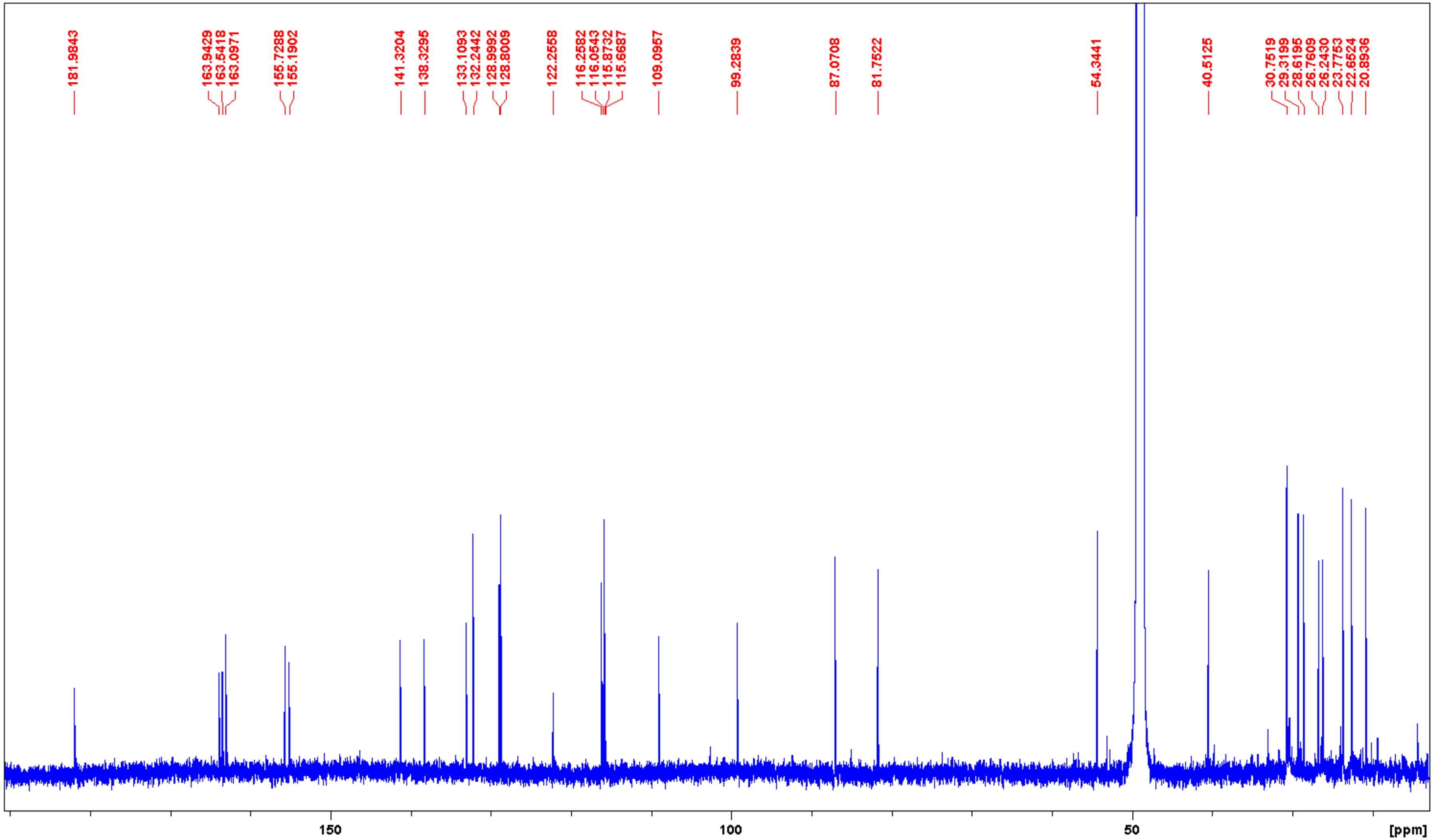

Figure S114. HSQC spectrum of **24** (600 MHz, methanol-d_4).

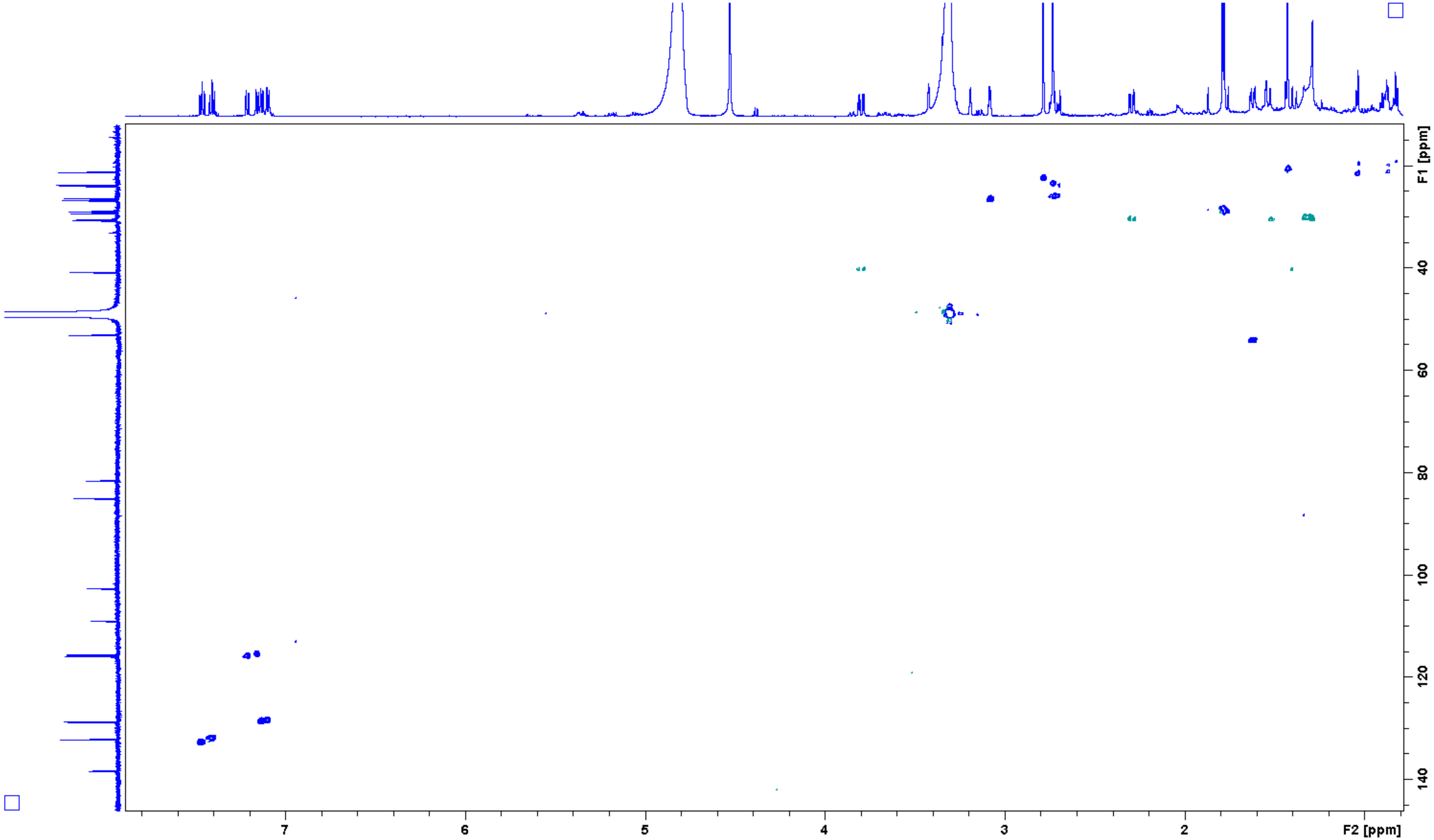

Figure S115. COSY spectrum of **24** (600 MHz, methanol-d_4).

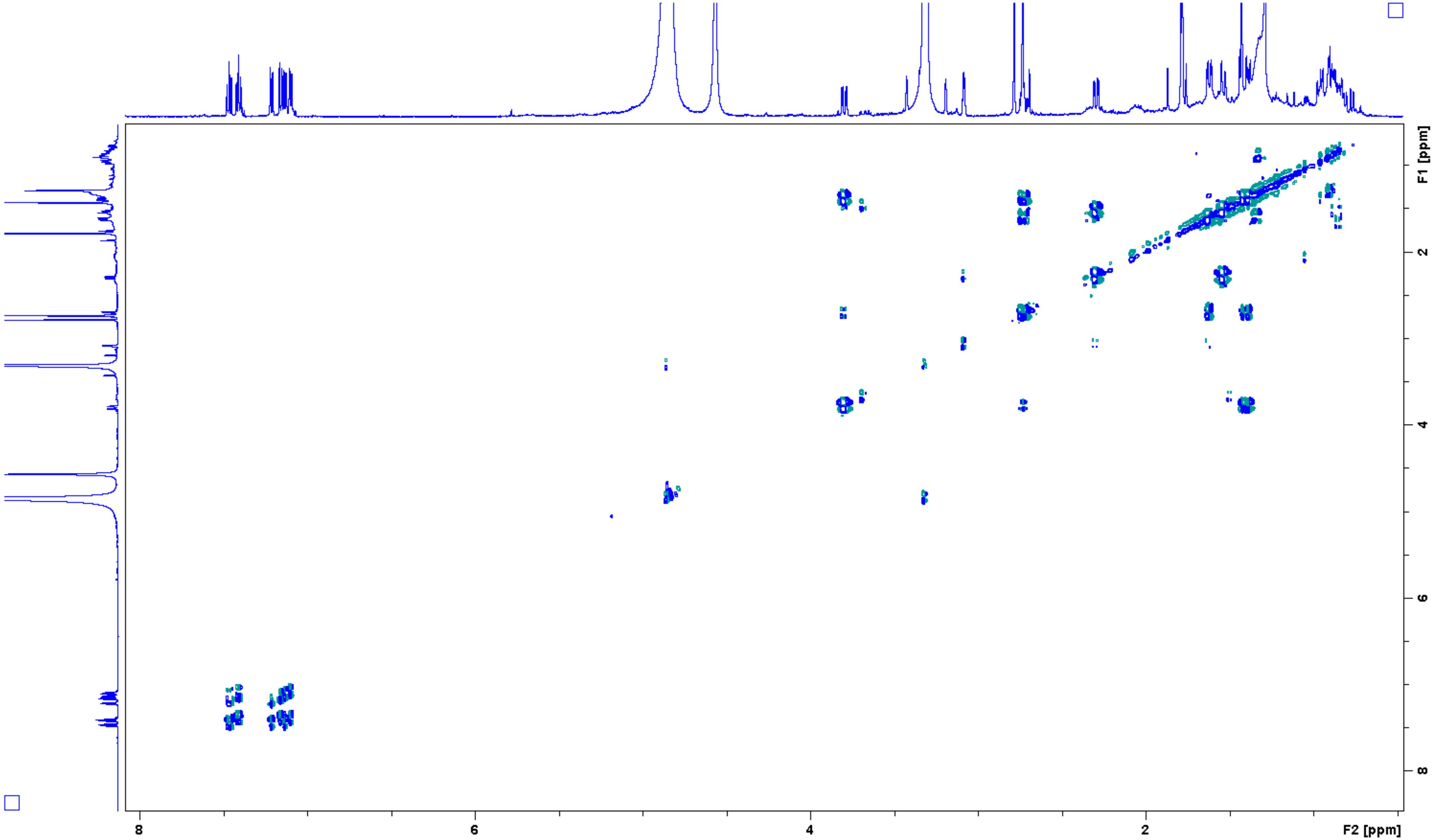

Figure S116. HMBC spectrum of **24** (600 MHz, methanol-d_4).

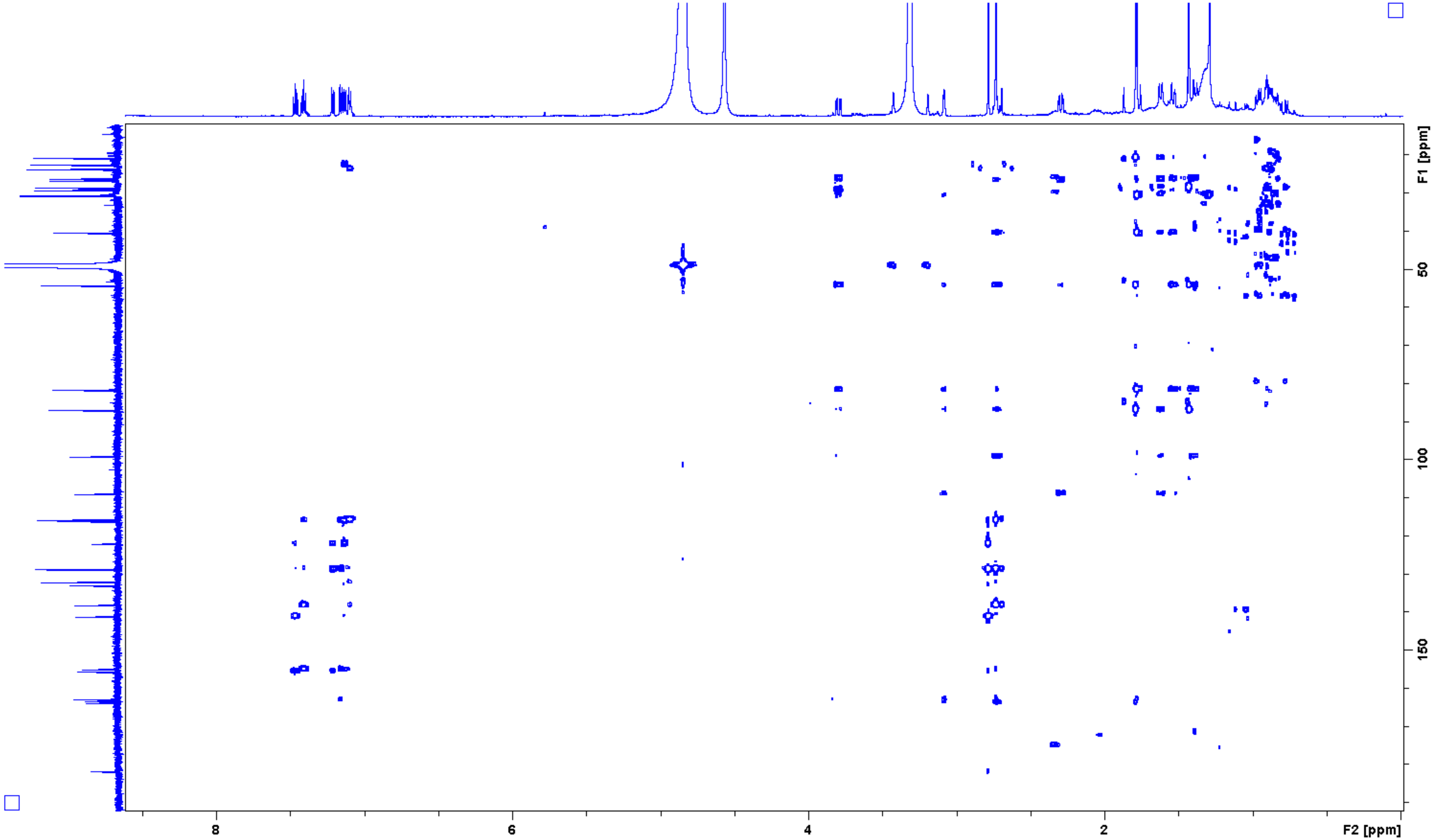

Figure S117. ROESY spectrum of **24** (600 MHz, methanol-d_4).

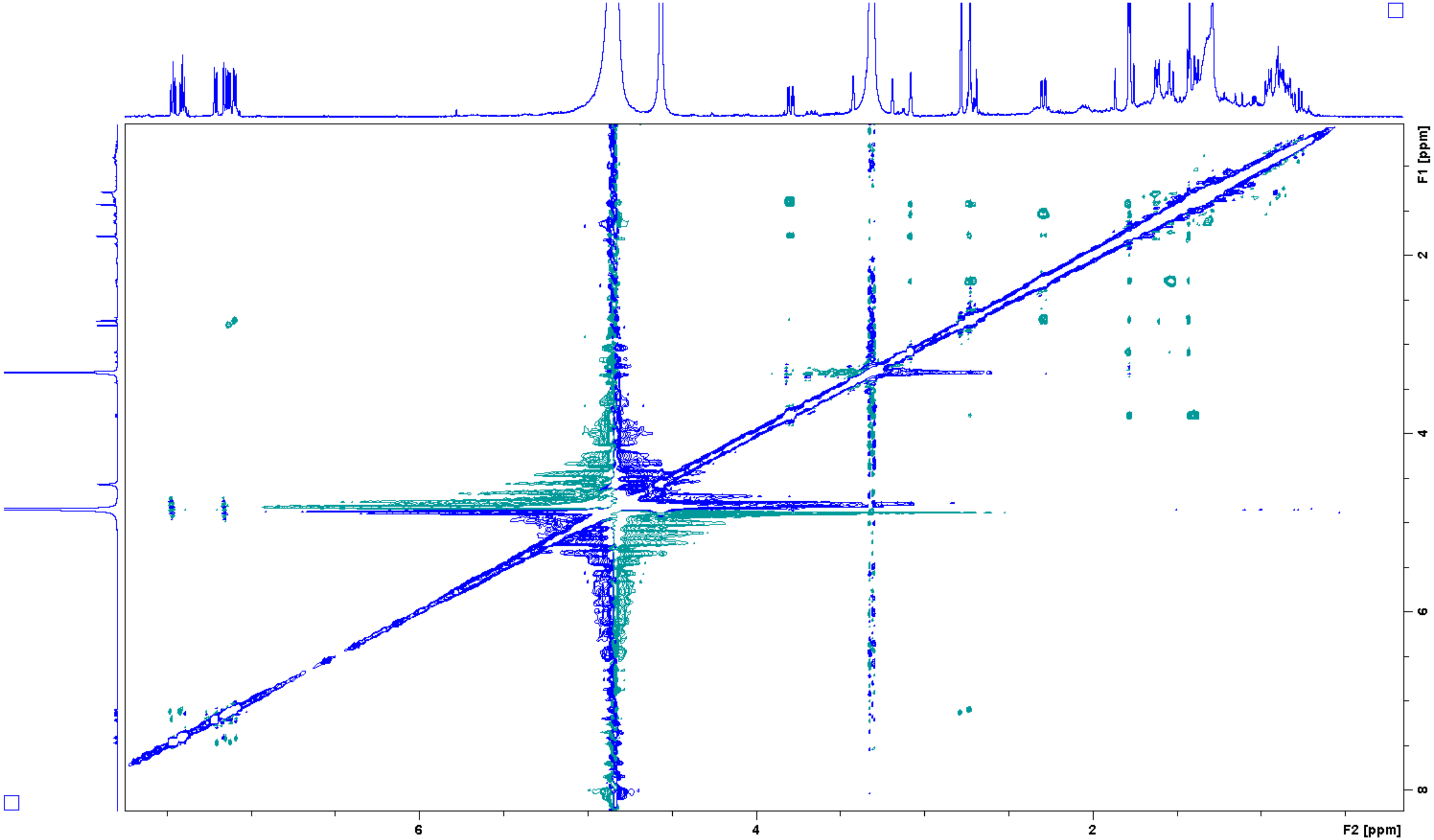

Figure S118. ^{1}H NMR spectrum of **25** (600 MHz, methanol-d_4).

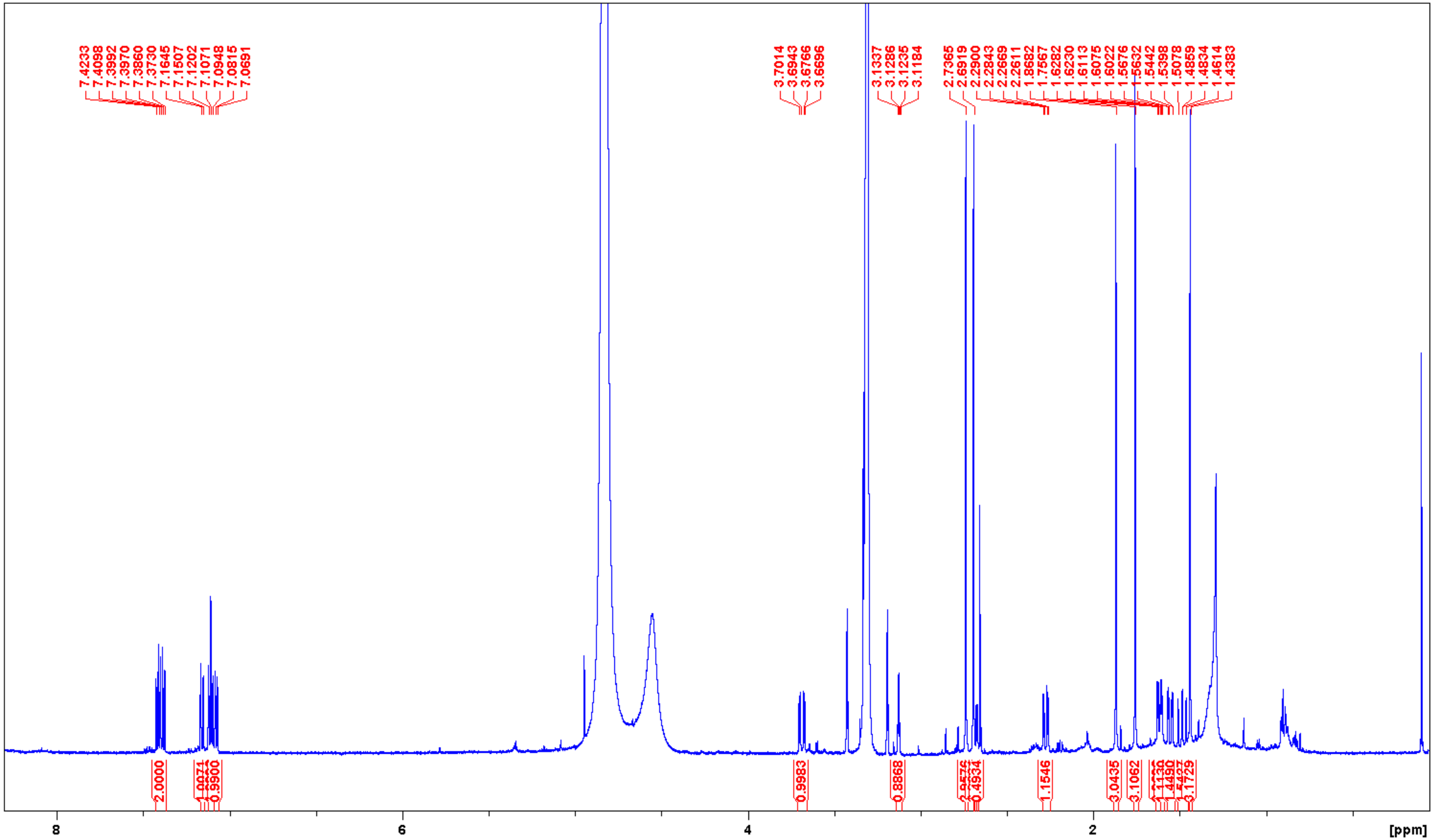

Figure S119. ^{13}C NMR spectrum of **25** (151 MHz, methanol-d_4).

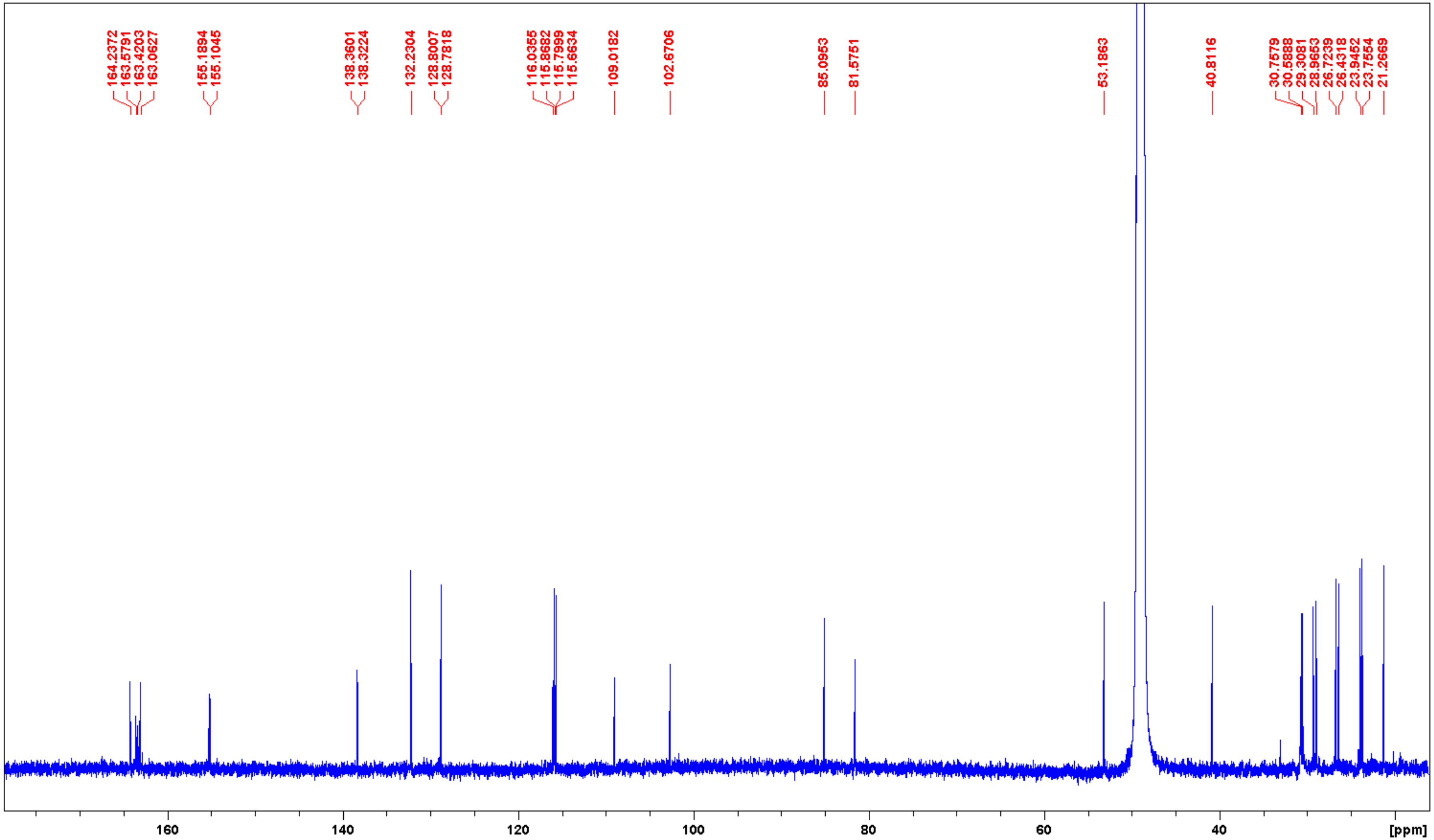

Figure S120. HSQC spectrum of **25** (600 MHz, methanol-d_4).

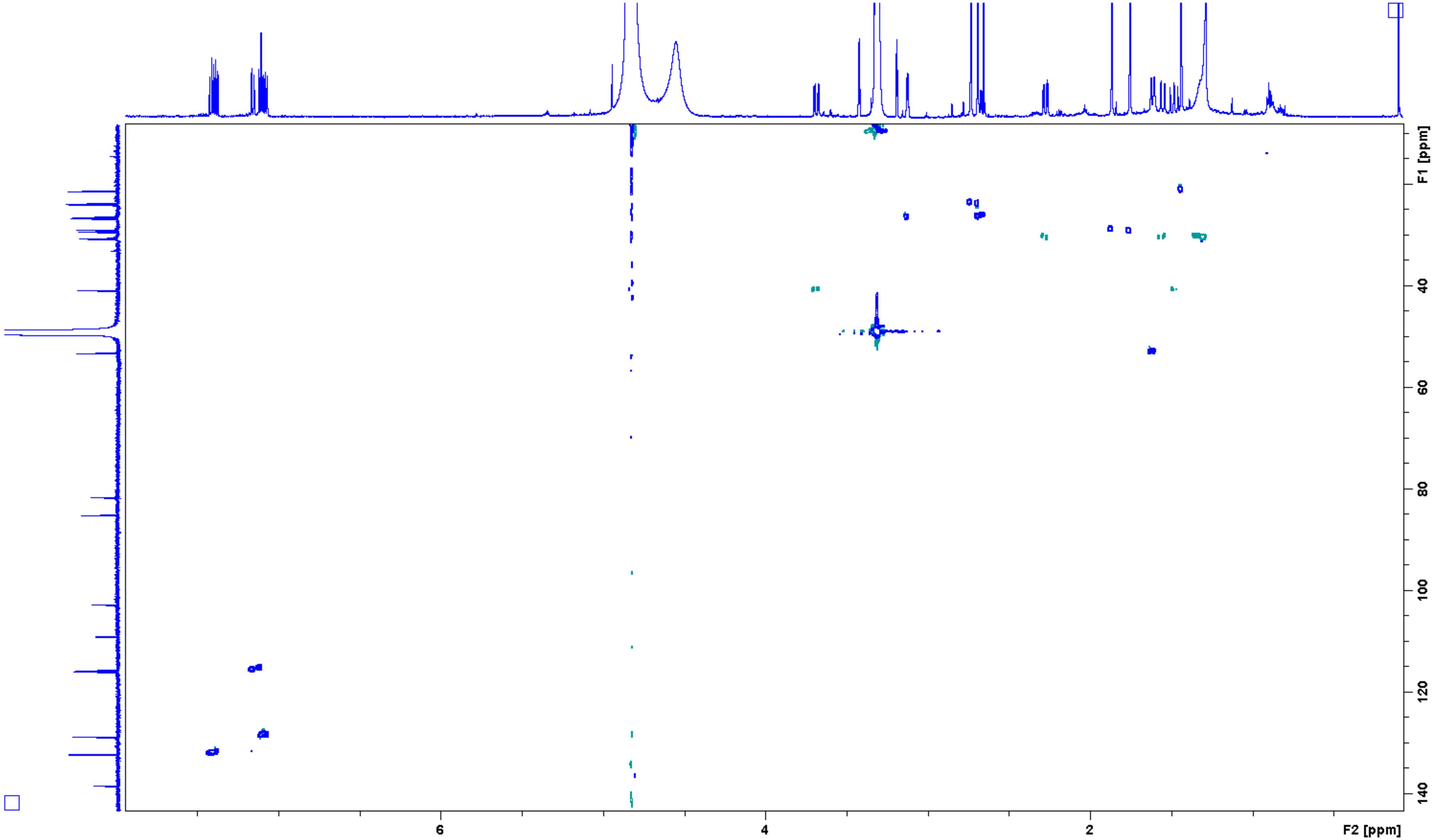

Figure S121. COSY spectrum of **25** (600 MHz, methanol-d_4).

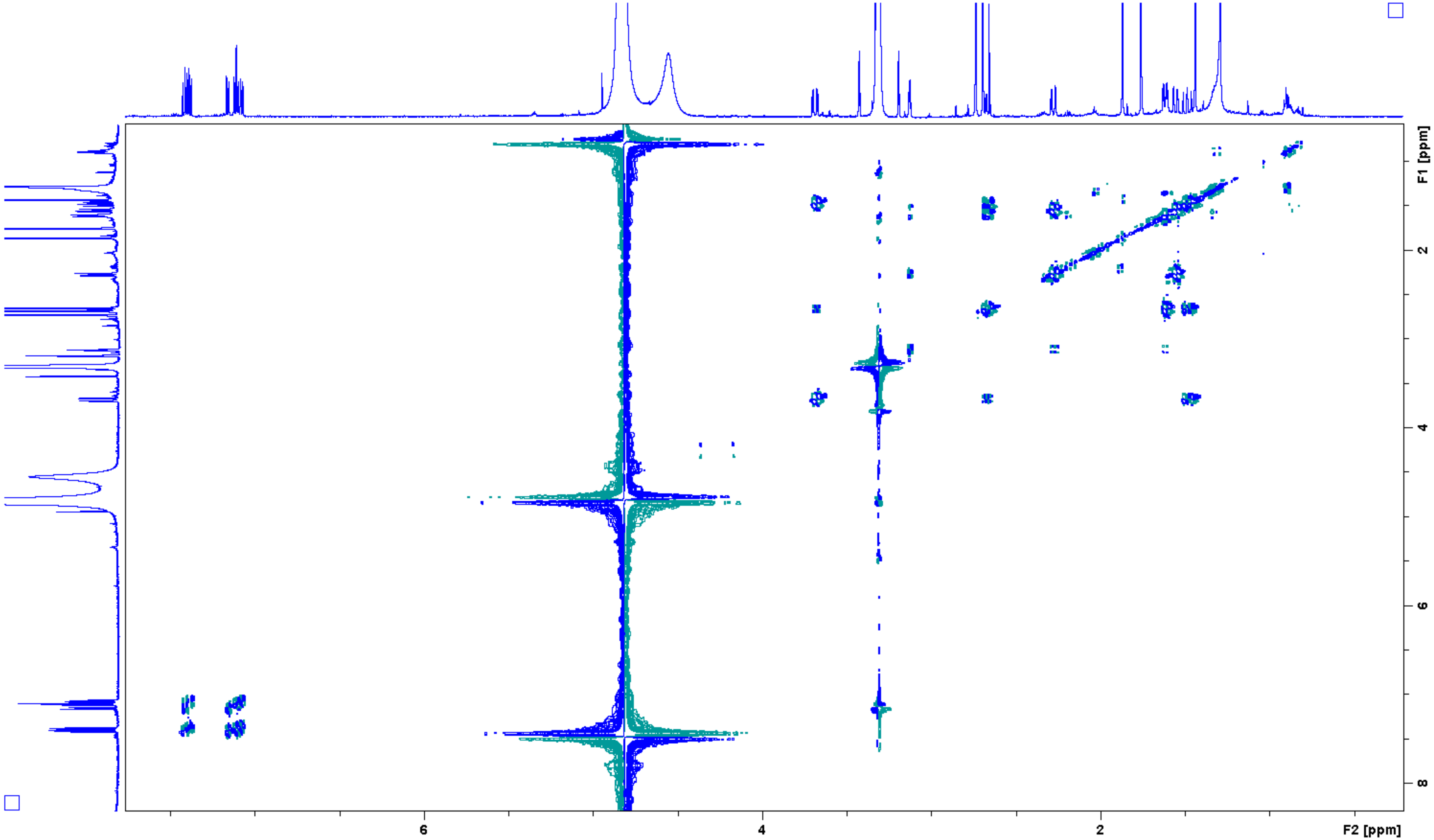

Figure S122. HMBC spectrum of **25** (600 MHz, methanol-d_4).

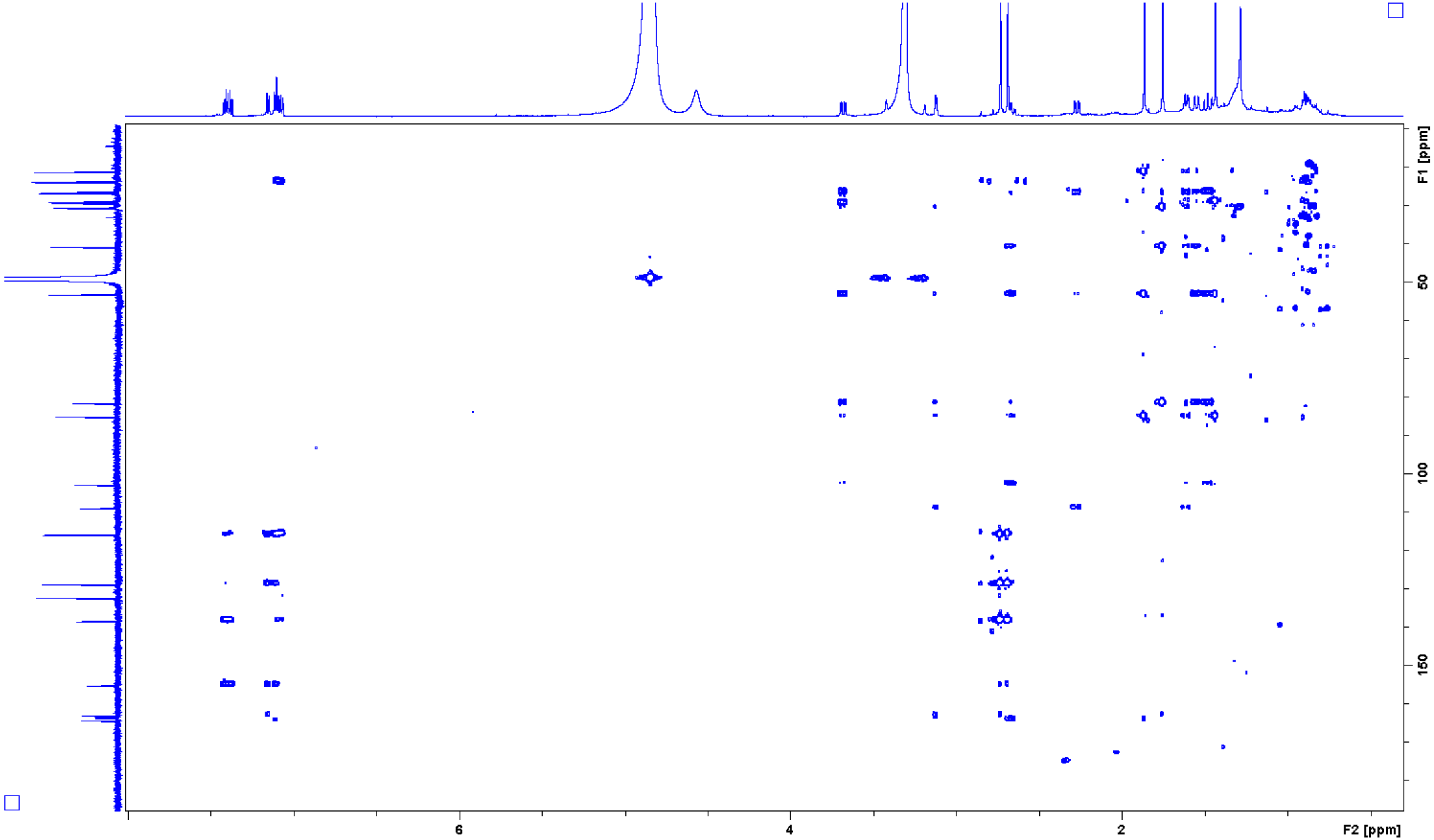

Figure S123. ROESY spectrum of **25** (600 MHz, methanol-d_4).

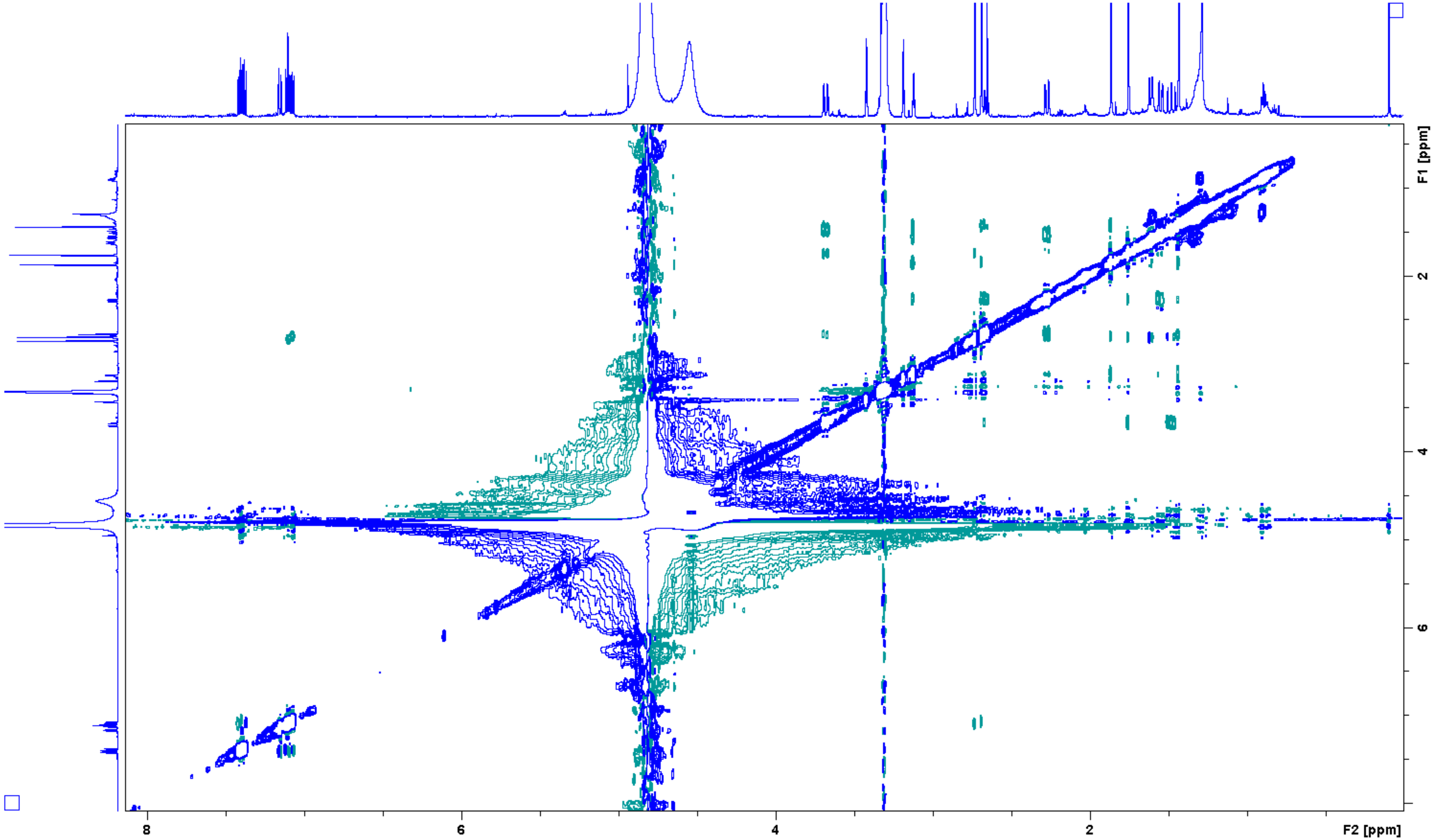

Table S16. Crystal data, data collection and structure refinement data of **19**

Parameter	Value
Formula:	$C_{30}H_{28}O_6$; 2 CD_3OD
Mw (g/mol)	556.7
Temperature, K	123(2)
Crystal class	monoclinic
Space group	*C*2/c
Cell parameters	a = 17.720(4) Å
	b = 15.753(3) Å
	c = 20.547(4) Å
	β = 107.81(3)°
V (Å^3), Z	5461(2) / 8
F(000)	2336
d_{calc} (g/cm3)	1.354
λ	1.54178 Å (Cu*K*α)
θ_{max} (°)	3.84 < θ < 70.17
Reflections total/unique	24636/5191
R_{merge}	0.0302
No. parameters	374
Reflections ($I > 2\ \sigma(I)$)	4591
R1/wR2 ($I > 2\ \sigma(I)$)	0.0357/0.0868
R1/wR2 (all reflections)	0.0416/0.0909
Goodness-of-fit on F^2	1.037
ρ_{max} / ρ_{min} (eÅ^{-3})	0.35 / -0.33
